# Crystalline nitrogen chain radical anions

**DOI:** 10.1038/s41557-025-02040-2

**Published:** 2026-02-10

**Authors:** Reece Lister-Roberts, Daniel Galano, Bono van IJzendoorn, George F. S. Whitehead, Adam Brookfield, Alice M. Bowen, Nikolas Kaltsoyannis, Meera Mehta

**Affiliations:** 1 Department of Chemistry, University of Manchester, Manchester, UK; 2 Department of Chemistry, University of Oxford, Oxford, UK; 3 The EPSRC National Research Facility for Electron Paramagnetic Resonance, Center for Quantum Science and Engineering, Photon Science Institute, University of Manchester, Manchester, UK

**Keywords:** Coordination chemistry, Chemical bonding

## Abstract

Long-chain nitrogen ions and radicals ([N_*n*_]^*x*+^/[N_*n*_]^*x*−^, *n* > 3) are naturally occurring under the intense radiative conditions of the Earth’s ionosphere and those of other planetary bodies. However, the strong thermodynamic driving force to lose N_2_ renders these types of molecule extremely reactive under ambient conditions such that they can typically be studied only under extreme conditions, for example, at ultrahigh pressures (10 GPa to >200 GPa). Here we report the isolation of a series of five molecules featuring metal unsupported {N_4_}^•−^ units under ambient conditions, with one derivative demonstrating remarkable multi-week long persistence in the solid state. Spectroscopic, crystallographic and computational studies provide insight into the bonding across the {N_4_}^•−^ chain. Reactivity studies reveal that the chain can cleave into N1 and N3 fragments, and can act as a source of nitrene radical anions, an observation that such molecules could act as storable nitrogen group transfer reagents.

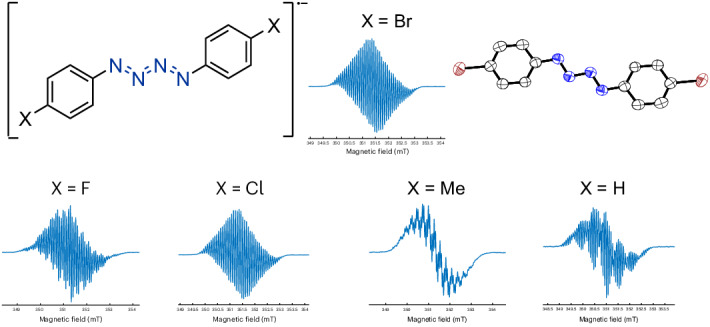

## Abstract (web summary)

The isolation of catenated nitrogen compounds is difficult, in part because these chains can readily lose nitrogen, creating a strong thermodynamic push towards decomposition. Now, a series of molecules containing radical anions of four-atom nitrogen chains have been synthesized and studied under ambient conditions; the chain can cleave into N1 and N3 fragments, and can act as a source of nitrene radical anion.

## Main

Carbon’s ability to form linear molecular chains is unmatched and central to how our biology and the materials in our world operate. By contrast, its neighbour nitrogen heavily disfavours chain formations, partially owing to the disproportionately strong N≡N triple bond when compared with N–N single and double bonds. This makes loss of dinitrogen (N_2_) gas an enormous thermodynamic driving force from catenated nitrogen^1^. This ability for nitrogen chains to readily release N_2_ renders them potent high-energy-density materials with applications as propellants, explosives and as gas generators^2,3^. Linear chains of N_*n*_ where *n* > 3 tend to be especially reactive and difficult to handle.

Nonetheless, nitrogen chains and their corresponding ions are of enormous interest. For example, sandwiched between the lower atmosphere and the magnetosphere, the ionosphere makes life on Earth possible by absorbing harmful radiation from the sun^4^. By absorbing this radiation, the ionosphere also increases the fidelity of radio communication and navigation. In this region of the upper atmosphere, where N_2_ is bombarded by solar radiation and cosmic rays, and under artificial plasma conditions, various nitrogen chain ions and radicals have been detected, including [N_4_]^+^, [N_5_]^+^ and [N_5_]^−^ (ref. ^5^). Similar ions are also thought to exist in the turbulent atmosphere of Titan^6,7^. These fleetingly stable molecules have been detected by mass spectrometric studies and trapped at very low temperatures in inert-gas matrices^8,9^. Furthermore, at ultrahigh pressures, the existence of different nitrogen phases has been demonstrated, and crystalline N_*n*_ (*n* > 3) chains identified in technologically relevant metal nitrides^10–20^. Yet, studying nitrogen chain ions under ambient conditions presents a formidable challenge, and a better understanding of their electronic structures should reveal a wealth of hitherto untapped chemical space.

**Fig. 1:**
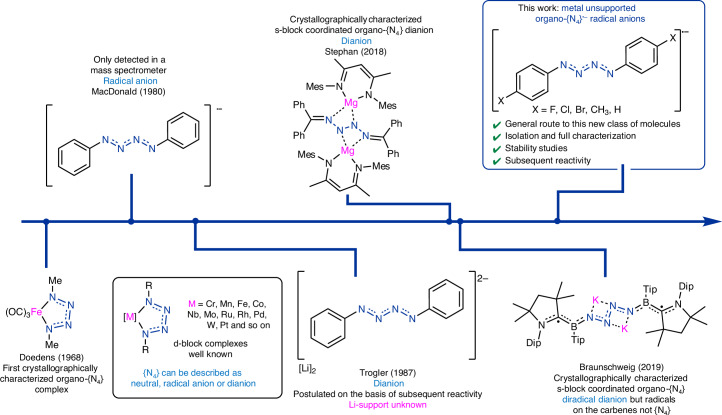
Timeline of key discoveries in organo-{N_4_} anion chemistry. Previous notable developments in organo-{N_4_} anion chemistry and this work. Mes, 1,3,5-trimethylphenyl; Dip, 2,6-diisopropylphenyl; Tip, 2,4,6-triisopropylphenyl.

Nitrogen chain anions substituted with organic groups are similarly under-investigated. An {N_4_} dianion flanked with organic groups was first reported in the form [Li]_2_[(Ph)_2_N_4_] (ref. ^21^), although this compound was not spectroscopically characterized (Fig. 1). The corresponding radical monoanion ([(Ph)_2_N_4_]^•−^) remains particularly elusive^22^, presumably owing to its radical nature in addition to the N chain structure. In 1980, McDonald detected [(Ph)_2_N_4_]^•−^ by mass spectroscopic studies and believed it to be generated from N_2_ loss from the azidobenzene (PhN_3_) to give the corresponding nitrene radical anion, which then coordinates a second equivalent of PhN_3_ (ref. ^23^). As with the unsubstituted ions, this extant body of literature suggests that organic compounds featuring {N_4_}^•−^ chains are fleetingly stable and accessible only under extreme conditions.

One common strategy to isolate highly reactive molecular fragments is to trap them in the coordination sphere of metals^24–26^. In the context of {N_4_} chemistry, numerous research groups have coordinated tetraazabutadiene (R–N=N–N=N–R) ligands to metals across the d-block, Mg and K^21,27–41^ (Fig. 1). When coordinated to d-block metals, the [R_2_N_4_] ligand can exhibit redox non-innocence and can be described as neutral, radical anionic or dianionic^29,30^. These groups also note that the naked [R_2_N_4_] ligand, that is, the corresponding neutral di-substituted tetraazadienes, could not be isolated and thus the {N_4_} unit is generated within the metal coordination sphere. Similar metal coordinated {N_6_} chains have also been reported by Jones, Stasch, Schulz and Holland^42–44^. Most relevant to this work, Braunschweig reported the compound [({cAAC}BTip)_2_(μ^2^-K)_2_N_4_] (cAAC = 1-(2,6-diisopropylphenyl)-3,3,5,5-tetramethylpyrrolidin-2-ylidene; Tip = 2,4,6-triisopropylphenyl), featuring a potassium supported {N_4_}^2−^ unit constructed directly from N_2_ gas^31^. However, in this approach, interactions between the metal and fragment, for example, *σ*-donation and *π*-backdonation, can perturb the electronic structure of said fragment and alter its geometry, raising questions as to whether the coordinated fragment accurately represents the unsupported species. Another common approach to enable isolation of reactive fragments is to invoke kinetic stabilization, and this has very recently been demonstrated by Hupf, Beckmann, Ye and Tan with the isolation of a triplet nitrene^45,46^. However, this tactic may not be the ideal solution to stabilizing ‘longer’ N_*n*_ chains, as the sterically encumbered substituents required are synthetically labour intensive and need to be large enough to span multiple atomic units. Thus, we consider delocalization of charge to stabilize the otherwise difficult-to-catch {N_4_}^•−^ unit.

Herein we report the synthesis of a robust metal unsupported nitrogen chain radical anion, [(4-BrC_6_H_4_)_2_N_4_]^•−^ ([**1**]^•−^). The electronic structure of compound [**1**]^•−^ is computationally and experimentally studied, and the radical character is confirmed to be primarily distributed across the {N_4_} chain. Further, we find that direct reduction of azides provides a general route to this class of molecules with a total of 5 derivatives reported (Fig. 1). The subsequent chemical reactivity of [**1**]^•−^ is surveyed and indicates the possibility that such a molecule can act as an N1 source, highly relevant in chemical synthesis.

## Results and discussion

### Synthesis and characterization

**Fig. 2:**
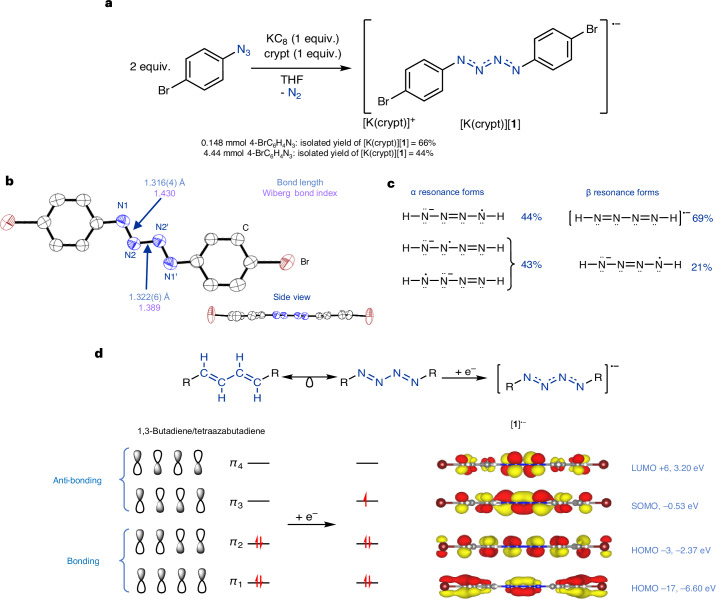
Synthesis and bonding analysis of [K(crypt)][**1**]. **a**, Synthesis of [K(crypt)][**1**]. **b**, Molecular structure of [**1**]^•−^ in [K(crypt)][**1**] showing anisotropic displacement ellipsoids at 50% probability. Counter cation and hydrogens omitted for clarity. ‘ denotes symmetry element: –X, +Y, 0.5–Z. Nitrogen, blue; carbon, white; bromine, brown. **c**, NRT calculated resonance forms of [HN_4_H]^•−^ (TPSS/def2-TZVP level of theory). **d**, Isolobal relationship between 1,3-butadiene and [RN_4_R], and selected Kohn–Sham molecular orbitals of [**1**]^•−^ (calculated at the TPSS/def2-TVZP level of theory) with simplified molecular orbital diagram focusing on the *π*-bonding of the {N_4_} unit.

First, 2 equiv. (0.148 mmol) of 1-azido-4-bromobenzene (4-BrC_6_H_4_N_3_) was reacted with 1 equiv. of potassium graphite (KC_8_) in the presence of 4,7,13,16,21,24-hexaoxa-1,10-diazabicyclo[8.8.8]hexacosane (crypt), resulting in a black solution (Fig. 2a). The reaction mixture was analysed by nuclear magnetic resonance (NMR) spectroscopy, and only the cation sequestering agent could be observed, consistent with it being diamagnetic (Supplementary Section 2.2). No other diamagnetic species were observed by the NMR studies. Dark yellow crystals, which can appear red-black, suitable for single-crystal X-ray diffraction (XRD) studies were obtained and authenticated the nitrogen-containing product to be the {N_4_} radical anion [**1**]^•−^ (Fig. 2b). The anion in [K(crypt)][**1**] was confirmed to be paramagnetic by electron paramagnetic resonance (EPR) spectroscopy, vide infra. Compound [K(crypt)][**1**] was isolated in 66% yield at this scale, but the reaction could be scaled 30× (4.44 mmol of 4-BrC_6_H_4_N_3_) with only a small decrease in yield. Bulk purity of [K(crypt)][**1**] was assessed by elemental analysis, powder XRD and spin counting using continuous wave EPR spectroscopy, and all methods confirmed high purity.

The structural data of [**1**]^•−^ reveal the terminal N1–N2 (which is the same as N1′–N2′) bond length to be 1.316(4) Å and the internal N2–N2′ bond length to be 1.322(6) Å, with the anion sitting on a crystallographically induced centre of inversion (Fig. 2b). These bond lengths are shorter than that of a hydrazine N–N single bond (1.45 Å) and longer than a *trans*-azobenzene N=N double bond (1.19 Å)^47,48^, consistent with partial multiple bond character between the N–N bonds of [**1**]^•−^. The ∠C1–N1–N2 of 111.8(3)° and ∠N1–N2–N2′ of 110.2(4)° are between the expected angles of an *sp*^2^ (120°) and *sp*^3^ (109.5°) hybridized nitrogen^49^. Density functional theory (DFT) calculations were conducted to further understand the structure and bonding of [**1**]^•−^, with all discussed data computed at the TPSS/def2-TZVP level of theory unless stated otherwise. Consistent with the slightly shorter N1–N2 crystallographic bond length, the Wiberg bond indices indicate that the N1–N2 bond has slightly higher bond order (N1–N2, 1.430; N2–N2′, 1.389). Natural localized molecular orbital calculations revealed the expected nitrogen hybridization between *sp*^2^ and *sp*^3^ (Supplementary Section 2.4). These data are consistent with the bonding across the {N_4_} unit as being both delocalized and to have partial multiple bond character between a single and double bond. This description is also supported by infrared spectroscopy, in which the stretches related to the {N_4_} unit are observed at 1,236 cm^−1^ (in good agreement with the calculated stretches at 1,272 cm^−1^), appearing between those reported for hydrazine (1,077 cm^−1^, N–N) and azobenzene (1,440 cm^−1^, N=N)^50,51^.

A range of partial atomic charge types were computed, and natural population analysis, Hirshfeld, Löwdin and Mulliken data all agree that the charge of [**1**]^•−^ is concentrated on the {N_4_} fragment with some delocalization onto the aromatic ring (Supplementary Section 2.5). Natural resonance theory (NRT) calculations of [**1**]^•−^ also support a highly delocalized electronic structure with 248 α spin resonances and 134 β spin resonances found, and with no structure contributing more than 2.6%. To simplify the picture and focus on the {N_4_} core, the model system [HN_4_H]^•−^ was investigated. NRT studies of [HN_4_H]^•−^ found 6 key α spin resonance forms with a total weight of 87% and 4 key β spin resonance forms with a total weight of 90%. Figure 2c shows the unique forms. The NRT data show that the majoirty of the radical and anion character is at the terminal (N1) and internal (N2) nitrogens, but which sites—terminal or internal—are prone to subsequent reactivity is unclear.

The Kohn–Sham molecular orbitals of [**1**]^•−^ were analysed, and as expected showed considerable delocalization over the whole molecule (Supplementary Section 2.6). Consistent with the isolobal relationship between 1,3-butadiene and the neutral [RN_4_R]^52^, and that addition of one electron to [RN_4_R] gives [RN_4_R]^•−^, we observed the expected molecular orbitals corresponding to the Hückel theory description of *π*-bonding^53^ (Fig. 2d). The singly occupied molecular orbital (SOMO) was found to be the *π*_3_ combination with anti-bonding character between N1–N2 and bonding character between N2–N2′.

**Fig. 3:**
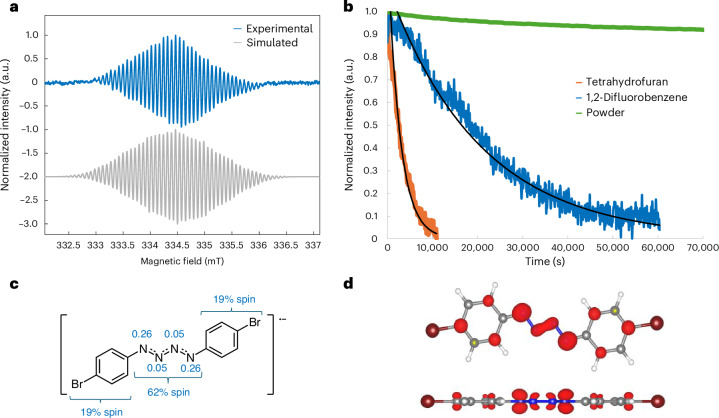
Spin density characterization and lifetime of [K(crypt)][**1**]. **a**, Experimental (blue) and simulated (grey, ×2 *A*_N_ = 14.61 MHz, ×2 *A*_N_ = 0.23 MHz, ×4 *A*_H_ = 8.51 MHz, ×4 *A*_H_ = 3.70 MHz, ×2 *A*_Br_ = 2.05 MHz, *g* = 2.006, lw = 0.04) continuous wave EPR spectra (298 K, 0.1 G, 2 scans, 9.390 GHz, sample sealed in a J Young tube under N_2_, in THF) of [K(crypt)][**1**]. **b**, Decay curves of [K(crypt)][**1**] determined by continuous wave EPR spectroscopy, where the integrated resonance intensity from a continuous wave EPR spectrum recorded every 90 s is reported (600 s for powder). **c**, Calculated Mulliken spin densities at the TPSS/def2-TZVP level of theory. **d**, Spin density plots from above and side-on with isovalue = 0.005.

EPR spectroscopy of [K(crypt)][**1**] confirmed the presence of an unpaired electron centred at *g* = 2.006 (Fig. 3a). The complexity of the spectrum is consistent with the delocalization of the radical. A single-point DFT calculation (computed at EPR-III (aug-cc-PVTZ for Br)/B3LYP/SMD(THF), where SMD means solvation model density and THF means tetrahydrofuran) gave an estimate of the isotropic hyperfine values and suggested that the electron has the largest coupling to the N1 atoms with *A*_N_ = 13.1 MHz. Refinement of the simulation gave a better fit to the experimental data (×2 *A*_N_ = 14.61 MHz, ×2 *A*_N_ = 0.23 MHz, ×4 *A*_H_ = 8.51 MHz, ×4 *A*_H_ = 3.70 MHz and ×2 *A*_Br_ = 2.05 MHz; Supplementary Section 2.7). The simulation finds that the electron is indeed most strongly coupled to one of the nitrogen environments (×2 *A*_N_ = 14.61 MHz) with smaller couplings from the rest of the molecule. The spin densities were also calculated using a variety of established methods: Mulliken, Löwdin and Hirshfeld (Supplementary Section 2.8). All these methods support delocalization of the radical across the structure with the {N_4_} unit having the largest portion of the spin density (62% from Mulliken; Fig. 3c). Further, these calculations revealed that the largest individual spin densities are on the terminal nitrogens (N1 and N1′; 26% each from Mulliken), followed by the *ortho*- (10% from Mulliken) and *para*- (11% from Mulliken) positions of the aromatic rings. These data provide insight into the resonance stabilization provided by the aromatic rings, where the terminal nitrogens can be regarded as benzylic-like showing similar delocalization effects as benzylic radicals^54^. Figure 3d shows the spin density plots; these plots and the Kohn–Sham SOMO show electron density above and below the plane of the molecule in a *π*-type orbital with 97% *p* character. The greater charge density at the *ortho*- than the *para*-positions is consistent with inductive effects also being considerable^55^. These findings suggest the terminal nitrogens as potential sites for subsequent reactivity.

The stability of [K(crypt)][**1**] was probed with half-life experiments using EPR spectroscopy (Fig. 3b). An EPR spectrum was periodically recorded and the peak intensities were used to generate decay curves, which revealed that [K(crypt)][**1**] is more stable in 1,2-difluorobenzene (*o*DFB) than in THF (*o*DFB, *t*_1/2_ = ~4 h; THF, t_1/2_ = ~42 min; Supplementary Section 2.10). In the solid state, [K(crypt)][**1**] was found to be remarkably stable, with only a negligible decrease in EPR resonance intensity observed after 2 days and the radical still present after 6 weeks when stored under anaerobic conditions.

**Fig. 4:**
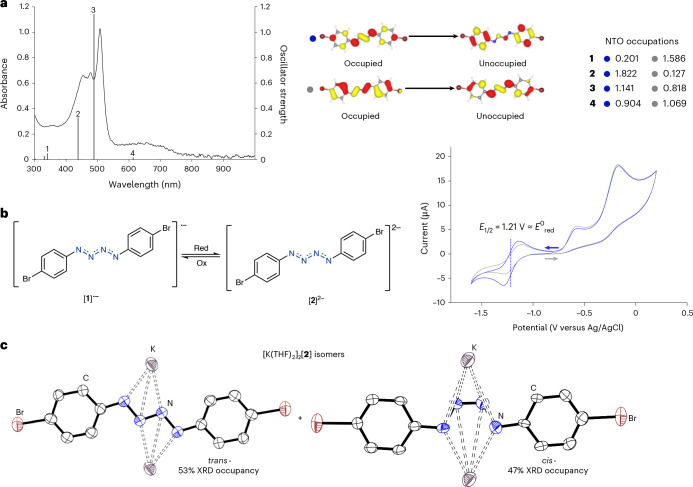
UV–vis and redox studies into [K(crypt)][**1**]. **a**, Experimental UV–vis spectrum (in *o*DFB solvent) and oscillator strengths from the calculated UV–vis spectrum of [**1**]^•−^. Key NTOs and their occupations with blue and grey dots in computed transitions 1–4. Oscillator strengths and NTOs were calculated at the TPSSh/def2-TZVP/SMD (cyclopentanone) level of theory. **b**, Left: reduction reaction of [**1**]^•−^ to [**2**]^2−^. Right: cyclic voltammogram of [K(crypt)][**1**] ([*n*Bu_4_N][PF_6_], 100 equiv.) 3 mM in THF at 0.1 V s^−1^ starting at −0.8 V and scanning independently in the positive direction first (grey trace) and the negative direction first (blue trace). Glassy carbon working electrode, platinum wire counter electrode and leak-proof Ag/AgCl reference electrode were used. Red, reduction; ox, oxidation. **c**, Molecular structures of the *cis*- and *trans*-isomers of [K(THF)_2_]_2_[**2**] showing anisotropic displacement ellipsoids at 50% probability with hydrogen atoms and coordinated solvent omitted for clarity. Nitrogen, blue; carbon, white; bromine, brown; potassium, violet.

Ultraviolet–visible (UV–vis) studies revealed a strong absorption of blue/green light as well as a weaker broad absorption tailing the whole of the visible region, consistent with the black appearance of [K(crypt)][**1**]. Time-dependent DFT (Supplementary Section 2.13.2) calculations were undertaken to better understand the nature of the electronic transitions resulting in this spectrum. Four key excitations were found and investigated further using natural transition orbitals (NTOs). The NTO data revealed that all four of the excitations are primarily underpinned by the same two orbital pairs with differing occupations, pictured in Fig. 4a. The transition identified with the blue dot can be described as a movement of electron density from the {N_4_} chain onto the aromatic rings, and the one with a grey dot with the opposite character.

[K(crypt)][**1**] was investigated by cyclic voltammetry (CV) to identify redox events (Supplementary Section 2.12). This CV data revealed a reversible single-electron reduction event at –1.21 V (versus Ag/AgCl). The one-electron reduction of [**1**]^•−^ would result in the formation of diamagnetic [**2**]^2−^ (Fig. 4b); the analogous phenyl-substituted lithium salt has been reported^21^. However, in this report, the dianionic lithium salt was not spectroscopically characterized, although a related Mg species has been isolated and the Mg cations were found to coordinate the {N_4_}^2−^ chain^28^. Similar reversible reduction of {N_4_}^•−^ to {N_4_}^2−^ has also been reported in the context of a tetrazene ligand coordinated to an iron centre^56^. Thus, efforts were made to chemically reduce [**1**]^•−^ using KC_8_ and cobaltocene. No reaction was observed in the case of cobaltocene, and with KC_8_ rapid gas evolution (believed to be N_2_ loss) was immediately observed and only decomposition products detected by NMR spectroscopy. However, when direct reduction of 1 equiv. of 4-BrC_6_H_4_N_3_ was tested with 1 equiv. of KC_8_ in the absence of crypt, single crystals obtained from the reaction mixture revealed the formation of the *cis*- and *trans*-isomers of potassium-coordinated [**2**]^2−^, with respective 47:53 relative freely refined XRD occupancies (Fig. 4c). Variable temperature NMR studies revealed that these isomers interconvert at room temperature, with the aromatic ^1^H resonances beginning to decoalesce at –80 °C (Supplementary Section 8.1). DFT calculations revealed that the *trans*-isomer is 3.8 kcal mol^−1^ more stable. However, [**2**]^2−^ was not characterized further as it was found to explode. Considering the molecular orbital diagram in Fig. 2d, the observed lower stability of [**2**]^2−^ is not entirely surprising. Addition of a second electron increases electron density in the highest occupied molecular orbital (HOMO), resulting in an increase in anti-bonding character between the N1 and N2 atoms and bonding character between the two internal nitrogens, which, in turn, promotes N_2_ elimination. Scanning the reversible reduction event at different scan rates (Supplementary Section 2.11) revealed that the redox event appears to be more reversible at lower scan rates, consistent with this event being chemically reversible on the CV timescale but demonstrating slow electron transfer, which could be the result of a structural change between the two states^57^.

**Fig. 5:**
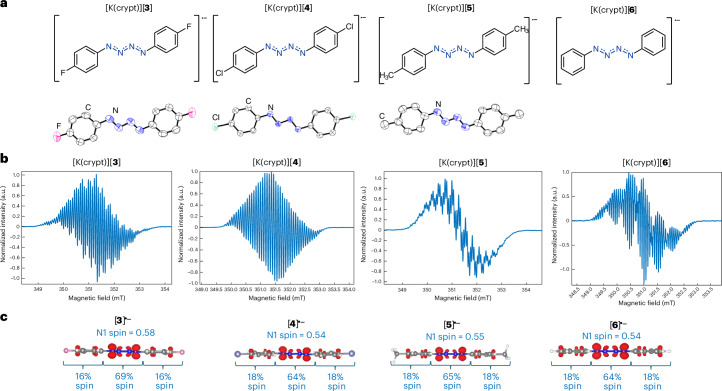
Structures and characterization of a series of molecules containing {N_4_}^•−^. **a**, Molecular structures of [**3**]^•−^, [**4**]^•−^, [**5**]^•−^ and [**6**]^•−^ in the [K(crypt)]^+^ salts, with XRD structures pictured with anisotropic displacement ellipsoids at 50% probability. Counter cation and hydrogens omitted for clarity. Nitrogen, blue; carbon, white; fluorine, pink; chlorine, dark green. **b**, Continuous wave EPR spectroscopy (298 K, 20 scans, X band, sample sealed in a J Young tube under N_2_) spectrum of [K(crypt)][**3**], [K(crypt)][**4**], [K(crypt)][**5**] and [K(crypt)][**6**] in THF (50/50 *o*DFB/Tol for [K(crypt)][**5**]). **c**, Spin density plots (isovalue = 0.005) of [**3**]^•−^, [**4**]^•−^, [**5**]^•−^ and [**6**]^•−^ with calculated total N1 spin density percentages provided (calculated at the TPSS/def2-TZVP level of theory).

In the case of benzylic radicals, spin delocalization is sensitive to aromatic ring substitution^54^. To probe spin density changes throughout [RN_4_R]^•−^-type molecules, radical anions [(4-FC_6_H_4_)_2_N_4_]^•−^ ([**3**]^•−^), [(4-ClC_6_H_4_)_2_N_4_]^•−^ ([**4**]^•−^), [(4-MeC_6_H_4_)_2_N_4_]^•−^ ([**5**]^•−^) and [(Ph)_2_N_4_]^•−^ ([**6**]^•−^; originally detected by McDonald^23^) were synthesized as the [K(crypt)]^+^ salts following a similar method to that used to prepare [K(crypt)][**1**] (Fig. 5). These compounds were characterized by single-crystal XRD (except for [K(crypt)][**6**]), EPR and UV–vis spectroscopy, CV, their spin densities calculated and decay curves collected in THF (Supplementary Section 3.7). The bonding across the {N_4_} unit for this family of radical anions ([**1**]^•−^ and [**3**]^•−^–[**6**]^•−^) is similar, as are the UV–vis data. Analysis of the cyclic voltammograms revealed that only [K(crypt)][**4**] exhibited a reversible reduction wave, similar to [K(crypt)][**1**], with the rest of the series having irreversible reductions with more negative reduction potentials. EPR studies revealed that the largest coupling remains at the terminal nitrogens (N1 positions) in all cases, which was also supported by the computed Mulliken spin densities, and that the spin density is sensitive to aromatic substitution (for example, highest Mulliken total N1 spin density = 58% for [**3**]^•−^ and lowest = 53% for [**1**]^•−^; largest *A*_N_ hyperfine coupling = 15.38 MHz for [**5**]^•−^ and lowest = 13.13 MHz for [**6**]^•−^). Interestingly, the half-life of the radicals was found to be [K(crypt)][**1**] > [K(crypt)][**4**] > [K(crypt)][**3**] > [K(crypt)][**6**] > [K(crypt)][**5**], with [K(crypt)][**5**] having the shortest *t*_1/2_ of 156 s (~2.6 min). Synthesis of these additional {N_4_}^•−^-containing molecules emphasizes the generality of the method developed in this work to access such previously elusive compounds. As the scaled synthesis of [K(crypt)][**1**] had already been established and as this compound demonstrated the greatest stability, [K(crypt)][**1**] was chosen as the focus of subsequent reactivity studies.

### Reactivity studies

**Fig. 6:**
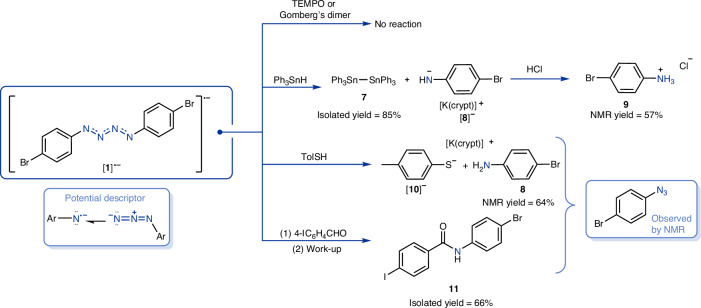
Reactivity studies of [K(crypt)][**1**]. No thiyl radical species or disulfide species is detected in the thiol chemistry, and the radical is believed to be quenched by H^•^ abstraction from either solvent or crypt^67^. Thiyl radicals are known to abstract H^•^ from ethers^68,69^. The crude reaction mixture from the reaction with 4-iodobenzaldehyde revealed unidentified decomposition products by ^1^H NMR spectroscopy, as is typical in nitrene C–H activation chemistry^63^.

Radical–radical recombination reactions have become useful tools to pinpoint the reactive site for paramagnetic materials^58^. For this reason, (2,2,6,6-tetramethylpiperidin-1-yl)oxyl (TEMPO) and 1,1′,1″-{[4-(diphenylmethylidene)cyclohexa-2,5-dien-1-yl]methanetriyl}tribenzene (Gomberg’s dimer) were independently reacted with [K(crypt)][**1**] (Fig. 6). In both reactions, these persistent radicals showed no reactivity towards [K(crypt)][**1**], further highlighting the high stability of [**1**]^•−^. Next, triphenyltin hydride was investigated as a source of H^•^ (ref. ^59^), a far less stable and less bulky radical, which did lead to radical quenching. Single crystals of hexaphenyldistannane (**7**) were obtained from the reaction mixture^60^, a known by-product from H^•^ elimination from tin hydrides^59^. ^1^H NMR studies of the crude reaction mixture revealed the formation of two doublets (both ^3^*J*_H–H_ = 8.2 Hz, 2H) in the aromatic region along with a broad singlet at 5.85 ppm, confirming the presence of the amide [**8**]^−^. After work-up, the product [**8**]^−^ was converted to 4-bromoanilinium chloride (**9**), confirmed by NMR spectroscopy. However, when 4 equiv. of 4-methylthiophenol (TolSH, **10**, a known source of H^•^ and H^+^)^61,62^ was investigated in place of the triphenyltin hydride, **8** and [K(crypt)][**10**] were formed with an NMR conversion of 68%. Crystals suitable for XRD analysis showed that **8** + [**10**]^−^ co-crystallized with the potassium cation sitting between them and the sulfur and nitrogen groups pointing towards one another. The absence of any residual electron density around sulfur and observed electron density in the difference map around nitrogen indicates that in the solid state, this product mixture exists as **8** + [K(crypt)][**10**]. Furthermore, the nitrogen-bound hydrogen atoms were freely refined by Hirshfeld atom refinement and confirmed them to be bound to nitrogen and not the sulfur. However, it is noteworthy that the diagnostic ^1^H NMR resonances corresponding to **8** + [K(crypt)][**10**] shift based on the relative concentrations of the two species, consistent with a proton from **8** shuttling to [**10**]^−^ and back again in solution. To better understand this reactivity, 1 equiv. of TolSH was reacted with [K(crypt)][**1**] and immediately inspected by ^1^H NMR spectroscopy. To our delight, the formation of 4-BrC_6_H_4_N_3_ was observed (Supplementary Section 8.3.1). Almost half a century ago, in McDonald’s work where [(Ph)_2_N_4_]^•−^ ([**6**]^•−^) was detected in a mass spectrometer, it was postulated that the {N_4_}^•−^ was generated by first ionizing 1 equiv. of azide to give the nitrene radical anion [PhN]^•−^, which subsequently forms an adduct with another equivalent of azide^23^ (pictorial description in Fig. 6). We computed the Gibbs reaction energy of the dissociation of this ‘adduct’ starting from [**1**]^•−^ to the corresponding azide and nitrene radical anion to be ~+19 kcal mol^−1^. To our knowledge this description of the bonding of such molecules has not previously been reported, in part because until now molecules featuring metal unsupported {N_4_}^•−^ units were not isolated. However, detection of 4-BrC_6_H_4_N_3_ in the thiol reaction demonstrates that [**1**]^•−^ can decompose in a manner consistent with this bonding description. Nitrene species are known to react with aldehydes to undergo carbonyl C–H activation^63^. In fact, in 1983, McDonald investigated the gas-phase reactivity of in situ-generated [PhN]^•−^ with aldehydes, which revealed the formation of the corresponding amide along with H^•^ (ref. ^64^). As [K(crypt)][**1**] may act as a source of nitrene radical anion, it was investigated with 4-iodobenzaldehyde (4-IC_6_H_4_CHO) and indeed the corresponding amide **11** could be isolated after work-up. Analysis of the crude reaction mixture by ^1^H NMR spectroscopy revealed the presence of expected 4-BrC_6_H_4_N_3_, as well as a singlet at 4.55 ppm corresponding to H_2_, suggesting the formation of H^•^ (refs. ^65,66^; Supplementary Section 8.4.1). It is also worth noting that 4-BrC_6_H_4_N_3_ does not react with 4-IC_6_H_4_CHO under the same conditions, and independently prepared [K(crypt)][**8**] reacts with 4-IC_6_H_4_CHO to give the corresponding imine not amide.

## Conclusion

Almost half a century after the first detection of a compound featuring an {N_4_}^•−^ chain in a mass spectrometer, we show that molecules featuring this moiety can be isolated under ambient conditions. Electronically stabilized by aromatic units, crystalline [(4-BrC_6_H_4_)_2_N_4_]^•−^ is isolated as a storable solid with multi-week stability under anaerobic conditions. The propensity of nitrogen catenates to rapidly degrade and release N_2_ has thus far been an impediment to fully understand the electronic structure of such chains, but the high stability of the metal unsupported [(4-BrC_6_H_4_)_2_N_4_]^•−^ has allowed this barrier to be overcome. Related derivatives [(4-FC_6_H_4_)_2_N_4_]^•−^, [(4-ClC_6_H_4_)_2_N_4_]^•−^, [(4-MeC_6_H_4_)_2_N_4_]^•−^ and [(Ph)_2_N_4_]^•−^ were also isolated and studied in a similar manner, and although changes in spin density across the {N_4_} chain and stability with aromatic substitution are observed, the computational and experimental electronic structure studies into the series are consistent with the {N_4_} chains having partial multiple bond character with substantial radical character on the terminal nitrogen atoms bonded to the aromatic units. These conclusions are consistent with the subsequent reactivity studies, which reveal that the {N_4_} chain of [(4-BrC_6_H_4_)_2_N_4_]^•−^ can decompose into N1 and N3 units, with reactivity consistent with the generation of a nitrene radical anion, exemplified by its reaction with an aldehyde where the carbonyl C–H bond is activated to give an amide. Efforts are now focused on exploring the additional reactivity patterns of {N_4_}^•−^-containing molecules, to enhance our understanding of their chemical properties and to fully unlock their potential as gram-scale storable nitrene synthons.

## Methods

### General considerations

NMR spectra were recorded on a Bruker AVIII 400 spectrometer at ambient temperatures. CV was carried out in the glovebox under inert conditions with EMStat4s. UV–vis electronic absorption spectra were recorded on a Mettler Toledo UV5Bio spectrophotometer using 10 mm path length quartz J Young cuvettes. EPR spectra were recorded at X band (9.4–9.8 GHz) with a Bruker EMXmicro spectrometer at 298 K. Spin counting was conducted on a Bruker Magnettech ESR5000 at X band (9.8 GHz), 298 K using the ESR Studio software’s incorporated spin counting function. Mass spectrometry samples were analysed using an electrospray ionization-equipped Waters RDa benchtop time-of-flight mass spectrometer. Attenuated total reflectance infrared (ATR-IR) spectra were recorded using a Bruker Alpha II under an inert atmosphere. X-ray diffraction data were collected for compounds [K(crypt)][**1**], [K(THF)_2_]_2_[**2**], [K(crypt)][**3**] and [K(crypt)][**4**] on a dual-source Rigaku XtaLAB Synergy-DW VHF equipped with a PhotonJet-R dual-wavelength rotating anode and HyPix-Arc 150° detector at 100 K. X-ray diffraction data were collected for [K(crypt)][**5**], **7** and **8** + [K(crypt)][**10**] on an Oxford Diffraction Supernova dual-source diffractometer at 150 K using Cu Kα (1.54184 Å) radiation equipped with a 135 mm Atlas CCD area detector.

### General procedure for preparation of [K(crypt)][RN_4_R] (R = *p*-XC_6_H_4_)

In the glovebox, KC_8_ (1 equiv.) and crypt (1 equiv.) were suspended in THF in a vial. Aryl azide (2 equiv.) was added and the vial shaken for 30 s. The solution was filtered and diethyl ether added to precipitate a black solid. The solid was filtered and washed with diethyl ether before drying under vacuum yielding [K(crypt)][RN_4_R] as a black crystalline solid. Single crystals were obtained by slow vapour diffusion of hexane into THF at –40 °C. Caution! Covalent azides are potentially hazardous and can decompose explosively under various conditions!

### Computational methodology

DFT calculations were carried out using the Gaussian 16 package, revision C.01 (ref. ^70^). Following extensive benchmarking (Supplementary Section 2.3), the TPSS functional was used^71^, together with the def2-TZVP basis set^72,73^. Geometry optimizations were performed, and all minima verified as true by harmonic vibrational frequency calculations. These frequencies were used in conjunction with Grimme’s quasi-harmonic approach for computation of the Gibbs energies^72,74^. The solvent environment was modelled using the SMD method^75^, with parameters appropriate to THF and cyclopentanone (a suitable model for *o*DFB). Natural bond orbital (NBO), natural localized molecular orbital and NRT calculations were carried out using NBO 7.0 (ref. ^76^). Time-dependent DFT and NTO single-point calculations were performed using the TPSSh functional^77^ and def2-TZVP basis set. EPR-related single-point calculations were carried out using ORCA 5.0.4 with the B3LYP functional^78–80^, with the aug-cc-pVTZ basis set used for F, Cl and Br^81^, and the EPR-III basis set for all other atoms^82^.

## Online content

Any methods, additional references, Nature Portfolio reporting summaries, source data, extended data, supplementary information, acknowledgements, peer review information; details of author contributions and competing interests; and statements of data and code availability are available at 10.1038/s41557-025-02040-2.

## Supplementary information

Supplementary Information Supplementary Figs. 1–83, Tables 1–40, discussion and crystallographic tables.

Supplementary Data 1 Raw data associated with [K(crypt)][**1**].

Supplementary Data 2 Raw data associated with [K(crypt)][**3**].

Supplementary Data 3 Raw data associated with [K(crypt)][**4**].

Supplementary Data 4 Raw data associated with [K(crypt)][**5**].

Supplementary Data 5 Raw data associated with [K(crypt)][**6**].

Supplementary Data 6 Raw data associated with [K(THF)2]2[**2**].

Supplementary Data 7 Raw data associated with decay curves and spin counting.

Supplementary Data 8 Raw data associated with all other crystals.

Supplementary Data 9 Coordinates from DFT.

## Data Availability

All data are available in the main text, Supplementary Information or Supplementary files. Cartesian coordinates of optimized structures are provided in the Supplementary files. Crystallographic data for structures reported in this article have been deposited at the Cambridge Crystallographic Data Centre, under deposition numbers 2423978 ([K(crypt)][**1**]), 2423981 ([K(THF)_2_]_2_[**2**]), 2481373 ([K(crypt)][**3**], 2481374 ([K(THF)_2_][**4**], 2481375 ([K(crypt)][**5**], 2423980 (**7**) and 2423979 (**8** + [K(crypt)][**10**]). These data can be obtained free of charge from The Cambridge Crystallographic Data Centre via www.ccdc.cam.ac.uk/data_request/cif. All data are also available from the corresponding authors upon request.

## References

[1] Greenwood, N. N. & Earnshaw, A. *Chemistry of the Elements* (Elsevier, 2012).

[2] Klapötke, T. M. *Chemistry of High-Energy Materials* (De Gruyter, 2022).

[3] O’Sullivan, O. T. & Zdilla, M. J. Properties and promise of catenated nitrogen systems as high-energy-density materials. *Chem. Rev.* **120**, 5682–5744 (2020). 32543838 10.1021/acs.chemrev.9b00804

[4] Heelis, R. A. & Maute, A. Challenges to understanding the Earth’s ionosphere and thermosphere. *J. Geophys. Res. Space Phys.* **125**, e2019JA027497 (2020).

[5] Pavlov, A. V. Photochemistry of ions at D-region altitudes of the ionosphere: a review. *Surv. Geophys.* **35**, 259–334 (2014).

[6] Molina-Cuberos, G. J., López-Moreno, J. J., Rodrigo, R. & Lara, L. M. Chemistry of the galactic cosmic ray induced ionosphere of Titan. *J. Geophys. Res. Planets* **104**, 21997–22024 (1999).

[7] Vuitton, V., Dutuit, O., Smith, M. & Balucani, N. in *Titan: Interior, Surface, Atmosphere, and Space Environment* (eds Müller-Wodarg, I. et al.) 224–284 (Cambridge Univ. Press, 2014).

[8] Cacace, F., de Petris, G. & Troiani, A. Experimental detection of tetranitrogen. *Science* **295**, 480–481 (2002). 11799238 10.1126/science.1067681

[9] Knight, L. B. Jr et al. ESR and ab initio theoretical studies of the cation radicals 14N+4 and 15N+4: the trapping of ion–neutral reaction products in neon matrices at 4 K. *J. Chem. Phys.* **87**, 885–897 (1987).

[10] Eremets, M. I., Gavriliuk, A. G., Trojan, I. A., Dzivenko, D. A. & Boehler, R. Single-bonded cubic form of nitrogen. *Nat. Mater.* **3**, 558–563 (2004). 15235595 10.1038/nmat1146

[11] Bykov, M. et al. Fe-N system at high pressure reveals a compound featuring polymeric nitrogen chains. *Nat. Commun.* **9**, 2756 (2018). 30013071 10.1038/s41467-018-05143-2 PMC6048061

[12] Laniel, D. et al. Synthesis of magnesium-nitrogen salts of polynitrogen anions. *Nat. Commun.* **10**, 4515 (2019). 31586062 10.1038/s41467-019-12530-w PMC6778147

[13] Gregoryanz, E. et al. Raman, infrared, and X-ray evidence for new phases of nitrogen at high pressures and temperatures. *Phys. Rev. B* **66**, 224108 (2002).

[14] Gregoryanz, E., Goncharov, A. F., Hemley, R. J. & Mao, H.-K. High-pressure amorphous nitrogen. *Phys. Rev. B* **64**, 052103 (2001).

[15] Goncharov, A. F., Gregoryanz, E., Mao, H.-k., Liu, Z. & Hemley, R. J. Optical evidence for a nonmolecular phase of nitrogen above 150 GPa. *Phys. Rev. Lett.* **85**, 1262–1265 (2000). 10991527 10.1103/PhysRevLett.85.1262

[16] Eremets, M. I., Hemley, R. J., Mao, H.-k. & Gregoryanz, E. Semiconducting non-molecular nitrogen up to 240 GPa and its low-pressure stability. *Nature* **411**, 170–174 (2001). 11346788 10.1038/35075531

[17] Yu, S. et al. Emergence of novel polynitrogen molecule-like species, covalent chains, and layers in magnesium–nitrogen Mg_*x*_N_*y*_ phases under high pressure. *J. Phys. Chem. C* **121**, 11037–11046 (2017).

[18] Zhu, S. et al. Stable calcium nitrides at ambient and high pressures. *Inorg. Chem.* **55**, 7550–7555 (2016). 27428707 10.1021/acs.inorgchem.6b00948

[19] Wei, S. et al. Alkaline-earth metal (Mg) polynitrides at high pressure as possible high-energy materials. *Phys. Chem. Chem. Phys.* **19**, 9246–9252 (2017). 28322368 10.1039/c6cp08771j

[20] Ding, C. et al. Single-bonded nitrogen chain and porous nitrogen layer via Ce–N compounds. *Mater. Adv.* **4**, 2162–2173 (2023).

[21] Lee, S. W., Miller, G. A., Campana, C. F., Maciejewski, M. L. & Trogler, W. C. Generation of mono- and dianions of 1,4-diphenyl-2-tetrazene by nonoxidative N-N bond formation. A novel route to a 2-tetrazene, a silacyclotetrazene, and the tetrazenide complex (1,4-diphenyltetrazenido)bis(triethylphosphine) palladium. *J. Am. Chem. Soc.* **109**, 5050–5051 (1987).

[22] Nguyen, M. T. Polynitrogen compounds. 1. Structure and stability of N_4_ and N_5_ systems. *Coord. Chem. Rev.* **244**, 93–113 (2003).

[23] McDonald, R. N. & Chowdhury, A. K. Hypovalent radicals. 7. Gas-phase generation of phenylnitrene anion radical and its reaction with phenyl azide. *J. Am. Chem. Soc.* **102**, 5118–5119 (1980).

[24] Johnson, M. J. A., Odom, A. L. & Cummins, C. C. Phosphorus monoxide as a terminal ligand. *Chem. Commun.* **1997**, 1523–1524 (1997).

[25] Braunschweig, H., Radacki, K. & Schneider, A. Oxoboryl complexes: boron–oxygen triple bonds stabilized in the coordination sphere of platinum. *Science* **328**, 345–347 (2010). 20395506 10.1126/science.1186028

[26] Sun, J. et al. A platinum(II) metallonitrene with a triplet ground state. *Nat. Chem.* **12**, 1054–1059 (2020). 32839602 10.1038/s41557-020-0522-4

[27] Vanicek, S. et al. Redox-rich metallocene tetrazene complexes: synthesis, structure, electrochemistry, and catalysis. *Organometallics* **38**, 1361–1371 (2019). 30930522 10.1021/acs.organomet.8b00681 PMC6437651

[28] Zhou, J., Liu, L. L., Cao, L. L. & Stephan, D. W. Reductive coupling and loss of N_2_ from magnesium diazomethane derivatives. *Chem. Eur. J.* **24**, 8589–8595 (2018). 29719075 10.1002/chem.201802138

[29] Vaddypally, S., McKendry, I. G., Tomlinson, W., Hooper, J. P. & Zdilla, M. J. Electronic structure of manganese complexes of the redox-non-innocent tetrazene ligand and evidence for the metal-azide/imido cycloaddition intermediate. *Chem. Eur. J.* **22**, 10548–10557 (2016). 27339316 10.1002/chem.201600531

[30] Gross, M. E., Trogler, W. C. & Ibers, J. A. Delocalized π bonding in tetraazadiene metallocycles. *J. Am. Chem. Soc.* **103**, 192–193 (1981).

[31] Légaré, M.-A. et al. The reductive coupling of dinitrogen. *Science* **363**, 1329–1332 (2019). 30898929 10.1126/science.aav9593

[32] Gärtner, A. et al. Achieving control over the reduction/coupling dichotomy of N_2_ by boron metallomimetics. *J. Am. Chem. Soc.* **145**, 8231–8241 (2023). 36977310 10.1021/jacs.3c01762

[33] Obenhuber, A. H., Gianetti, T. L., Berrebi, X., Bergman, R. G. & Arnold, J. Reaction of (bisimido)niobium(V) complexes with organic azides: [3 + 2] cycloaddition and reversible cleavage of β-diketiminato ligands involving nitrene transfer. *J. Am. Chem. Soc.* **136**, 2994–2997 (2014). 24524190 10.1021/ja413194z

[34] Cramer, S. A., Hernández Sánchez, R., Brakhage, D. F. & Jenkins, D. M. Probing the role of an FeIV tetrazene in catalytic aziridination. *Chem. Commun.* **50**, 13967–13970 (2014). 10.1039/c4cc05124f 25265968

[35] Geisenberger, J., Nagel, U., Sebald, A. & Beck, W. (2-Tetrazen-1,4-diyl)platin(IV)-Komplex: Struktur von(PhC≡C)2(Et3)2Pt[1,4-(4-NO2C6H4)2N4]. *Chem. Ber.* **116**, 911–916 (1983).

[36] Park, J. Y., Kim, Y., Bae, D. Y., Rhee, Y. H. & Park, J. Ruthenium bisammine complex and its reaction with aryl azides. *Organometallics* **36**, 3471–3476 (2017).

[37] Zhong, W. et al. Synthesis and reactivity of the imido-bridged metallothiocarboranes CpCo(S_2_C_2_B_10_H_10_)(NSO_2_R). *Organometallics* **31**, 6658–6668 (2012).

[38] Elpitiya, G. R., Malbrecht, B. J. & Jenkins, D. M. A chromium(II) tetracarbene complex allows unprecedented oxidative group transfer. *Inorg. Chem.* **56**, 14101–14110 (2017). 29116767 10.1021/acs.inorgchem.7b02253

[39] Lee, S. W. & Trogler, W. C. Synthesis, structure, and properties of dicarbonyl bis(phosphine) 1,4-diphenyltetraazabutadiene complexes of molybdenum and tungsten. *Organometallics* **9**, 1470–1478 (1990).

[40] Danopoulos, A. A., Wilkinson, G., Sweet, T. K. N. & Hursthouse, M. B. Reactions of imido complexes of iridium, rhodium and ruthenium. *Dalton Trans.* **1996**, 3771–3778 (1996).

[41] Doedens, R. J. Molecular configuration of (Me)_2_N_4_Fe(CO)_3_, a tetrazadiene–tricarbonyliron complex. *Chem. Commun.* **1968**, 1271–1272 (1968).

[42] Bonyhady, S. J., Green, S. P., Jones, C., Nembenna, S. & Stasch, A. A dimeric magnesium(I) compound as a facile two-center/two-electron reductant. *Angew. Chem. Int. Ed.* **48**, 2973–2977 (2009). 10.1002/anie.200900331 19294719

[43] Gondzik, S. et al. Reactions of a Zn(I) complex with group 14 azides—formation of zinc azide and zinc hexazene complexes. *Chem. Commun.* **50**, 927–929 (2014). 10.1039/c3cc47687a 24254029

[44] Cowley, R. E. et al. A bridging hexazene (RNNNNNNR) ligand from reductive coupling of azides. *J. Am. Chem. Soc.* **130**, 6074–6075 (2008). 18419120 10.1021/ja801375g

[45] Janssen, M. et al. Synthesis of a stable crystalline nitrene. *Science* **385**, 318–321 (2024). 38870274 10.1126/science.adp4963

[46] Wang, D. et al. Isolation and characterization of a triplet nitrene. *Nat. Chem.* **17**, 38–43 (2025). 39562811 10.1038/s41557-024-01669-9

[47] Collin, R. L. & Lipscomb, W. N. The crystal structure of hydrazine. *Acta Crystallogr.* **4**, 10–14 (1951).

[48] Harada, J., Ogawa, K. & Tomoda, S. Molecular motion and conformational interconversion of azobenzenes in crystals as studied by X-ray diffraction. *Acta Crystallogr. B* **53**, 662–672 (1997).

[49] Haynes, W. M. *CRC Handbook of Chemistry and Physics* (CRC Press, 2014).

[50] Gulaczyk, I., Kręglewski, M. & Valentin, A. The N–N stretching band of hydrazine. *J. Mol. Spectrosc.* **220**, 132–136 (2003).

[51] Fujino, T. & Tahara, T. Picosecond time-resolved Raman study of trans-azobenzene. *J. Phys. Chem. A* **104**, 4203–4210 (2000).

[52] Hoffmann, R. Building bridges between inorganic and organic chemistry (Nobel lecture). *Angew. Chem. Int. Ed. Eng.* **21**, 711–724 (1982).

[53] Roberts, J. D. & Caserio, M. C. *Basic Principles of Organic Chemistry* (Benjamin, 1977).

[54] Dust, J. M. & Arnold, D. R. Substituent effects on benzyl radical ESR hyperfine coupling constants. The σ_α_• scale based upon spin delocalization. *J. Am. Chem. Soc.* **105**, 1221–1227 (1983).

[55] Fehir, R. J. Jr & McCusker, J. K. Differential polarization of spin and charge density in substituted phenoxy radicals. *J. Phys. Chem. A* **113**, 9249–9260 (2009). 19606813 10.1021/jp905314h

[56] Cowley, R. E., Bill, E., Neese, F., Brennessel, W. W. & Holland, P. L. Iron(II) complexes with redox-active tetrazene (RNNNNR) ligands. *Inorg. Chem.* **48**, 4828–4836 (2009). 19397284 10.1021/ic900001y

[57] Yamada, H., Yoshii, K., Asahi, M., Chiku, M. & Kitazumi, Y. Cyclic voltammetry part 1: fundamentals. *Electrochemistry* **90**, 102005–102005 (2022).

[58] Buzzetti, L., Crisenza, G. E. M. & Melchiorre, P. Mechanistic studies in photocatalysis. *Angew. Chem. Int. Ed.* **58**, 3730–3747 (2019). 10.1002/anie.201809984 30339746

[59] Clive, D. L. J. Triphenylstannane. *Encyclopedia of Reagents for Organic Synthesis* (John Wiley & Sons, 2001).

[60] Dakternieks, D., Kuan, F. S., Duthie, A. & Tiekink, E. R. T. The crystal structure of the triclinic polymorph of hexaphenyldistannane. *Main Group Met. Chem.* **24**, 65–66 (2001).

[61] Loh, Y. K. et al. Isolation of a pentadienyl-type radical featuring a central secondary carbon. *Nat. Synth.* **3**, 727–731 (2024).

[62] Danehy, J. P. & Parameswaran, K. N. Acidic dissociation constants of thiols. *J. Chem. Eng. Data* **13**, 386–389 (1968).

[63] Schmidt-Räntsch, T. et al. Nitrogen atom transfer catalysis by metallonitrene C–H insertion: photocatalytic amidation of aldehydes. *Angew. Chem. Int. Ed.* **61**, e202115626 (2022). 10.1002/anie.202115626 PMC9305406 34905281

[64] McDonald, R. N. & Chowdhury, A. K. Hypovalent radicals. 13. Gas-phase nucleophilic reactivities of phenylnitrene (PhN-•) and sulfur anion radicals (S-•) at sp3 and carbonyl carbon. *J. Am. Chem. Soc.* **105**, 198–207 (1983).

[65] Dixon-Lewis, G., Sutton, M. M. & Williams, A. The kinetics of hydrogen atom recombination. *Disc. Faraday Soc.* **33**, 205–212 (1962).

[66] Wang, S., Dames, E. E., Davidson, D. F. & Hanson, R. K. Reaction rate constant of CH_2_O + H = HCO + H_2_ revisited: a combined study of direct shock tube measurement and transition state theory calculation. *J. Phys. Chem. A* **118**, 10201–10209 (2014). 25319141 10.1021/jp5085795

[67] Charles, S., Danis, J. A., Mattamana, S. P., Fettinger, J. C. & Eichhorn, B. W. Protonation and hydrogen atom abstraction reactions in the synthesis of the [HP_7_M(CO)_3_]^2–^ ions (M = Cr, W). *Z. Anorg. Allg. Chem.* **624**, 823–829 (1998).

[68] Schöneich, C., Bonifačić, M., Dillinger, U. & Asmus K.-D. in *Sulfur-Centered Reactive Intermediates in Chemistry and Biology* (eds Chatgilialoglu, C. & Asmus, K.-D.) 367–376 (Springer, 1990).

[69] Dénès, F., Pichowicz, M., Povie, G. & Renaud, P. Thiyl radicals in organic synthesis. *Chem. Rev.* **114**, 2587–2693 (2014). 24383397 10.1021/cr400441m

[70] Frisch, M. J. et al. Gaussian 16 Rev. C.01 (2016).

[71] Tao, J., Perdew, J. P., Staroverov, V. N. & Scuseria, G. E. Climbing the density functional ladder: nonempirical meta–generalized gradient approximation designed for molecules and solids. *Phys. Rev. Lett.* **91**, 146401 (2003). 14611541 10.1103/PhysRevLett.91.146401

[72] Grimme, S. Supramolecular binding thermodynamics by dispersion-corrected density functional theory. *Chem. Eur. J.* **18**, 9955–9964 (2012). 22782805 10.1002/chem.201200497

[73] Weigend, F. & Ahlrichs, R. Balanced basis sets of split valence, triple zeta valence and quadruple zeta valence quality for H to Rn: design and assessment of accuracy. *Phys. Chem. Chem. Phys.* **7**, 3297–3305 (2005). 16240044 10.1039/b508541a

[74] Luchini, G., Alegre-Requena, J., Funes-Ardoiz, I. & Paton, R. GoodVibes: automated thermochemistry for heterogeneous computational chemistry data. *F1000Research* **9**, 291 (2020).

[75] Marenich, A. V., Cramer, C. J. & Truhlar, D. G. Universal solvation model based on solute electron density and on a continuum model of the solvent defined by the bulk dielectric constant and atomic surface tensions. *J. Phys. Chem. B* **113**, 6378–6396 (2009). 19366259 10.1021/jp810292n

[76] Glendening, E. D. et al. NBO 7.0 (Theoretical Chemistry Institute, Univ. of Wisconsin, 2018).

[77] Staroverov, V. N., Scuseria, G. E., Tao, J. & Perdew, J. P. Comparative assessment of a new nonempirical density functional: molecules and hydrogen-bonded complexes. *J. Chem. Phys.* **119**, 12129–12137 (2003). 10.1063/1.4971853 28010100

[78] Neese, F. The ORCA program system. *WIREs Comput. Mol. Sci.* **2**, 73–78 (2012).

[79] Becke, A. D. Density-functional thermochemistry. III. The role of exact exchange. *J. Chem. Phys.* **98**, 5648–5652 (1993).

[80] Lee, C., Yang, W. & Parr, R. G. Development of the Colle-Salvetti correlation-energy formula into a functional of the electron density. *Phys. Rev. B* **37**, 785–789 (1988). 10.1103/physrevb.37.785 9944570

[81] Kendall, R. A., Dunning, T. H. Jr & Harrison, R. J. Electron affinities of the first-row atoms revisited. Systematic basis sets and wave functions. *J. Chem. Phys.* **96**, 6796–6806 (1992).

[82] Barone, V. in *Recent Advances in Density Functional Methods* 287–334 (World Scientific Publishing, 1995).

