# Supplementary material for: Crystalline nitrogen chain radical anions
Source: Nat Chem. 2026 Feb 10;18(4):686–94. doi: 10.1038/s41557-025-02040-2 (PMC13061614; doi:10.1038/s41557-025-02040-2)
Supplement: Supplementary file 1 — Supplementary Figs. 1–83, Tables 1–40, discussion and crystallographic tables. [file 41557_2025_2040_MOESM1_ESM.pdf]

nature chemistry

Supplementary information https://doi.org/10.1038/s41557-025-02040-2

Crystalline nitrogen chain radical anions

In the format provided by the authors and unedited

Table of Contents

1. Methods and Materials 4

1.1 Experimental Considerations 4

1.2 Analytical Considerations 4

1.3 General Computational Considerations: 7

2. Synthesis and Characterization of [K(crypt)][1] 9

2.1 Synthesis of literature known compounds 9

2.1.1 Preparation of Potassium Graphite (KC_8)[2] 9

2.1.2 Preparation of Gomberg's Dimer[1] 9

2.2. Synthesis and Characterization Data of [K(crypt)][1] 11

2.3 Functional benchmarking against geometric structure 13

2.4 Calculated Bond Metric Data 14

2.5 Charge Delocalization Data 16

2.6 Molecular Orbital Diagram 19

2.7 EPR of [K(crypt)][1] 20

2.7.1 EPR of [K(crypt)][1] stacked with a simulation of $[1]^{•-}$ using calculated hyperfine values. 20

2.7.2 EPR of [K(crypt)][1] stacked with an optimized simulation 22

2.8 Calculated Spin Densities of $[1]^{•-}$ 24

2.9 Spin Counting of [K(crypt)][1] 26

2.10 Assessing Stability of [K(crypt)][1] 27

2.11 Cyclic Voltammetry of [K(crypt)][1] 29

2.12 Infrared Spectroscopy of [K(crypt)][1] 30

2.13 Ultraviolet-Visible Spectroscopy of [K(crypt)][1] 33

2.13.1 Observed UV-Vis Spectra of [K(crypt)][1] 33

2.13.2 Calculated UV-Vis Transitions 33

3. Synthesis and Characterization of [K(crypt)][3] 36

3.1 Synthesis of [K(crypt)][3] 36

3.2 Charge and Spin Delocalization Data of $[3]^{•-}$ 37

3.3 EPR Data of [K(crypt)][3] 41
3.4 Cyclic Voltammetry Data of [K(crypt)][3] 45
3.5 UV-Vis Spectrum and NTOs of [K(crypt)][3] 46
4. Synthesis and Characterization of [K(crypt)][4] 48
4.1 Synthesis of [K(crypt)][4] 48
4.2 Charge and Spin Delocalization data $[4]^{•-}$ 50
4.3 EPR data of [K(crypt)][4] 54
4.4 Cyclic Voltammetry Data of [K(crypt)][4] 58
4.5 UV-Vis spectrum and NTOs of [K(crypt)][4] 59
5. Synthesis and Characterization of [K(crypt)][5] 61
5.1 Synthesis of [K(crypt)][5] 61
5.2 Charge and Spin Delocalization of $[5]^{•-}$ 62
5.3 EPR data of [K(crypt)][5] 66
5.4 Cyclic Voltammetry Data of [K(crypt)][5] 70
5.5 UV-Vis spectrum and NTOs of [K(crypt)][5] 71
6. Synthesis and Characterization of [K(crypt)][6] 73
6.1 Synthesis of [K(crypt)][6] 73
6.2 Charge and Spin Densities of $[6]^{•-}$ 74
6.3 EPR data of [K(crypt)][6] 78
6.4 Cyclic Voltammetry Data of [K(crypt)][6] 82
6.5 UV-Vis spectrum and NTOs of [K(crypt)][6] 83
7. Comparison of Derivatives 85
8. Reactivity Studies 88
8.1 $[K(THF)_2]_2[2]$ 88
8.1.1 Synthesis of $[K(THF)_2]_2[2]$ 88
8.1.2 Investigation of *cis*- and *trans*- isomer interconversion 90
8.2 Addition of Ph_3SnH to [K(crypt)][1] 92
8.2.1 Addition of Ph_3SnH to [K(crypt)][1] with crude NMR 92
8.2.2 Addition of Ph_3SnH to [K(crypt)][1] with aqueous workup 93

8.3. Addition of TolSH to [K(crypt)][1] 95
8.3.1. Addition of 1 equivalent of TolSH 95
8.3.2 Addition of 4 equivalents of TolSH 97
8.3.3 Independent preparation of 1:2 mixture of 8 + [K(crypt)][10]. 98
8.4 Reaction with 4-IC_6H_4CHO 99
8.4.1. Reaction in THF-d_8 and crude NMR spectra 99
8.4.2. Reaction in *o*DFB and isolation of 11 100
8.4.3. Control reaction of 4-BrPhNHK with 4-IC_6H_4CHO 102
8.5 Calculated Energy of Azide Loss 104
9. Crystallography Tables 105
10. References 109

1. Methods and Materials

1.1 Experimental Considerations

All manipulations were performed under an inert atmosphere (unless specifically stated) using standard Schlenk line, and glovebox techniques. Glassware was flame dried prior to use.

Dry tetrahydrofuran (THF), diethyl ether (ether), toluene (Tol), dimethylformamide (DMF), acetonitrile (MeCN), hexane and pentane were obtained using Innovative Technologies anhydrous engineering solvent purification systems and subsequently degassed. 1,2-Difluorobenzene (*o*DFB) was dried over 3 Å molecular sieves. THF-d_8 and DMF-d_7 were dried over 3 Å sieves, $CDCl_3$ (containing TMS 1% v/v) and MeOD-D_4 were used without purification. All dry solvents were stored over activated 3 Å molecular sieves.

The following compounds were purchased from commercial suppliers and used without further purification. 1-Azido-4-bromobenzene (4-$BrC_6H_4N_3$, Fluorochem), 4-methylbenzenethiol (TolSH, Sigma-Aldrich), triphenyltin hydride (Ph_3SnH, Sigma-Aldrich), 4-iodobenzaldehyde (4-IC_6H_4CHO, Apollo Scientific), 2.2.2-cryptand (crypt, Sigma-Aldrich), TEMPO (Sigma-Aldrich), KCl (Sigma-Aldrich), KH (Thermo Fisher Scientific), magnesium sulfate ($MgSO_4$, Sigma-Aldrich) ^{t}butyl nitrite (Sigma-Aldrich), trimethylsilyl azide (TMS-N_3, Sigma-Aldrich), 4-chloroaniline (Sigma-Aldrich), 4-fluoroaniline (Sigma-Aldrich), 4-methylaniline (Sigma-Aldrich) and cobaltocene (Sigma-Aldrich).

Aniline was purchased from Sigma-Aldrich and distilled at 130°C under dynamic vacuum to yield a colourless oil.

KC_8, Gomberg's dimer and aromatic azides were synthesized using literature procedures.[1, 2, 3]

1.2 Analytical Considerations

Nuclear Magnetic Resonance. ^1H, $^{13}C\{^1H\}$ and ^{119}Sn NMR were recorded on a Bruker AVIII 400 spectrometer using operating frequencies 400.17 MHz, 100.55 MHz and 149.24 MHz respectively. ^1H and $^{13}C\{^1H\}$ NMR chemical shifts were internally referenced to the residual solvent resonances (THF-d_8 (tetrahydrofuran-d_8): ^1H δ = 3.58, 1.73 ppm, $^{13}C\{^1H\}$ δ = 67.57, 25.37 ppm, ($CDCl_3$ (chloroform-d), MeOD-d_4 (methanol-d_4): ^1H δ = 4.78, 3.31 ppm, $^{13}C\{^1H\}$ δ = 49.15 ppm, ^1H δ = 7.26 ppm), DMF-d_7 (dimethylformamide-d_7): ^1H δ = 8.03, 2.92 ppm. ^{119}Sn chemical shifts were referenced externally to Me_4Sn. Solution phase NMR samples were prepared in 5 mm J Young NMR tubes (under an inert atmosphere) where stated. All NMR were analyzed using MestReNova V15.0.0 software

Ultraviolet–visible spectroscopy. Ultraviolet-visible (UV-Vis) electronic absorption spectra were recorded on a Mettler Toledo UV5Bio spectrophotometer using 10 mm path length quartz J Young cuvettes.

Electron Paramagnetic Resonance. Electron paramagnetic resonance (EPR) spectra were recorded at X band (9.4 – 9.8 GHz) with a Bruker EMXmicro spectrometer at 298 K. Spin counting was conducted on a Bruker Magnettech ESR5000 at X band (9.8 GHz), 298 K and analyzed using the ESR Studio software's incorporated spin counting function. All EPR spectra were plotted using MATLAB R2024a and simulations conducted using easyspin-6.0.2 plugin.[4]

Cyclic Voltammetry. Cyclic voltammetry (CV) was carried out in the glovebox under inert conditions with EMStat4s. Electrodes: Working – glassy carbon. Counter – platinum wire. Pseudo reference – silver wire. Reference – Ag /AgCl (leak proof).

Mass spectrometry. Mass spectrometry samples were analyzed by the mass spectrometry service at the University of Oxford using an electrospray ionization (ESI) equipped Waters RDa bench-top time of flight mass spectrometer. Samples were prepared under a nitrogen atmosphere and directly injected into the ionization source of the mass spectrometer.

Infrared spectroscopy. ATR-IR spectra were recorded on microcrystalline powders using a Bruker Alpha II under an inert atmosphere.

Elemental analysis. Elemental analysis was carried out by the microanalysis service of the University of Manchester using a Flash 2000 elemental analyser. The sample was prepared under a nitrogen atmosphere.

X-ray diffraction studies. X-ray diffraction data was collected for compounds [K(crypt)][**1**], $[K(THF)_2]_2$[**2**], [K(crypt)][**3**], and [K(crypt)][**4**] on a dual source Rigaku XtaLAB Synergy-DW VHF equipped with a PhotonJet-R dual wavelength rotating anode and HyPix-Arc 150° detector at 100K. X-ray diffraction data was collected for [K(crypt)][**5**], **7**, and **8** + [K(crypt)][**10**] on an Oxford Diffraction Supernova dual-source diffractometer at 150K using Cu Kα (1.54184 Å) radiation equipped with a 135 mm Atlas CCD area detector. X-ray data was collected using CrysAlisPro software.[5]

Crystal structure determination and refinements: X-ray data was processed and reduced using CrysAlisPro. Absorption correction was performed using empirical methods (SCALE3 ABSPACK) based upon symmetry-equivalent reflections combined with measurements at different azimuthal angles. The crystal structure was solved and refined against all F2 values using the SHELX and Olex2 suite of programmes.[6, 7] All atoms were refined anisotropically.

Hydrogen atoms were placed in calculated positions and refined using idealized geometries and assigned fixed isotropic displacement parameters.

In $[K(THF)_2]_2$[**2**] positional and atomic displacement parameters were restrained using SHELX SIMU, RIGU, and SADI commands. Aryl rings were treated with the SHELX FLAT command. The occupancies of the *cis*- and *trans*- isomers were allowed to freely refine.

The structure of **8** + [K(crypt)][**10**] was first solved as described above. The NoSpherA2 implementation of Hirshfeld atom refinement (HAR) within Olex2 was then employed to confirm the accurate characterization of protic H atoms in our identification of **8**.[8] The quantum chemistry calculations were performed with ORCA 6.0.1.[9] All C-bound H atoms were placed at calculated positions and treated with the riding model as described above and were not refined by HAR. Two maxima were clearly identified in the Fourier difference map near the N atom and were assigned as H atoms. The positional and thermal parameters of the N-bound H atoms were allowed to refine freely and isotropically throughout the HAR procedure. An initial refinement was performed with the 3-21G basis set and the r2SCAN functional with low integration accuracy, sloppy SCF threshold, and slow convergence of the SCF.[10, 11] A second refinement was then performed with the Def2-SVP basis set and the r2SCAN functional with low integration accuracy, NoSpherA2 SCF threshold, and slow convergence of the SCF. A final refinement strategy was then performed iteratively with the def2-TZVP basis set and the r2SCAN with normal integration accuracy, strong SCF threshold, and slow convergence of the SCF.[12] The iterative procedure converged after 6 cycles. The N–H bond distances afforded by free refinement were unreasonably short. The N-bound H atoms were thus treated with a chemically reasonable SHELX DFIX command (d=1.0 Å). Similarity and rigid bond restraints were applied to the aryl sulfide C atoms. The CIF file for the NoSpherA2 solution has been included but not deposited to the CCDC as the overall solution is of a lower quality.

Crystallographic data have been deposited with the CCDC (CCDC 2423978-2423981, 2481373-2481375).

Powder X-ray diffraction: Data collection. Microcrystalline sample of [K(crypt)][**1**] was sealed inside a 0.5 mm outer diameter capillary and data were collected using a Rigaku FR-X rotating anode single crystal X-ray diffractometer using Cu Kα radiation (λ = 1.5418 Å) with a Hypix-6000HE detector and an Oxford Cryosystems nitrogen flow gas system. Data were collected between 3–50° 2θ with a detector distance of 150 mm and a beam divergence of 0.5 mRad.[13] X-ray data were collected using CrysAlisPro software.[5]

Microcrystalline sample of [K(crypt)][**4**] was sequestered from ambient air using home-made air-tight sample holders with mylar windows and collection conducted in-house using a Bruker D8 Advance Eco diffractometer. This is a high intensity instrument operating in Bragg-

Brentano geometry with CuK_a radiation (l ~ 1.5418 Å). The sample position is fixed and mounted on a flat plate whereas the X-ray source and LYNXEYE XE-T detector are rotated throughout the measurement. The detector has an energy threshold which allows for filtering of k_b radiation.

Powder X-ray diffraction was attempted on [K(crypt)][**3**], [K(crypt)][**5**], and [K(crypt)][**6**] but they did not diffract strongly enough to obtain data with sufficient resolution.

Data processing. The instrument was calibrated using the collected data, with the instrument model refined using diffraction peak positions measured at multiple detector angles. X-ray data were reduced and integrated using CrysAlisPro software.[5] Peak hunting and unit cell indexing was performed using TOPAS software.[14] Le Bail profile analysis was performed using JANA2020 software.[15]

1.3 General Computational Considerations:

DFT geometry optimizations and frequency calculations were carried out using the Gaussian 16 package, revision C.01.[16] Following benchmarking studies (see Section 2.3) the Tao–Perdew–Staroverov–Scuseria (TPSS) functional was used. Geometry optimizations were performed, with default settings, starting from crystallographic co-ordinates. Analysis of the harmonic vibrational frequencies confirmed the optimized geometries as energetic minima. IR spectra were plotted using the calculated vibrational frequencies (Supplementary Figure 18 and S19). Grimme's quasi-harmonic correction was employed to obtain the Gibbs energies, using the python-based code GoodVibes.[17, 18, 19] The Ahlrichs Def2 basis set of polarized triple-ζ quality (def2-TZVP) was used for all atoms.[20] Solvent environment was modelled using the smd method, with parameters appropriate to THF and cyclopentanone (a suitable model for *o*DFB).[21] Natural bond orbital (NBO), natural localized molecular orbital (NLMO) and natural resonance theory (NRT) calculations were carried out using NBO 7.0 program.[22, 23] Mulliken and Hirshfeld charges and spin densities were obtained from the output of the Gaussian 16 calculations.

50 state, full TDDFT calculations and subsequent NTO calculations were performed using the Gaussian 16 package with the meta-hybrid TPSS (TPSSh) functional (following benchmarking, see Section 2.13.2).[24] The def2-TZVP basis set was used for all atoms. The coordinate were taken from the TPSS/def2-TZVP/SMD(THF) calculation and not re-optimized.

EPR calculations were carried out using ORCA 5.0.4 with the B3LYP functional.[25, 26, 27] Dunning's correlation consistent basis set of polarized triple-ζ quality with diffuse functions (aug-cc-pVTZ) was used for F, Cl and Br, with Barone's EPR-III basis set for all other atoms.[28, 29] The coordinates for the EPR calculation were taken from the TPSS/def2-TZVP/SMD(THF) calculation and not re-optimized. Löwdin charges and spin densities were also obtained from

a single point calculation carried out within ORCA 5.0.4 at the TPSS/def2-TZVP level of theory. Solvent environment was modelled using the SMD method, with parameters appropriate to THF.

Plots of the spin density, Kohn-Sham orbitals and Natural Transition Orbitals were obtained from the Gaussian fchk file. Cube files for these were generated from the fchk file in Multiwfn.[30] These files were imported into VESTA to generate the corresponding figures.[31]

2. Synthesis and Characterization of [K(crypt)][1]

2.1 Synthesis of literature known compounds

2.1.1 Preparation of Potassium Graphite (KC_8)[2]

Freshly cut potassium (1 g, 25.6 mmol, 1 equiv.) and graphite (2.46 g, 204.8 mmol, 8 equiv.) were added to an ampoule in the glovebox and then heated under static vacuum at 130 °C for three hours with regular shaking. Potassium graphite was formed as a golden-brown powder in quantitative yield and used without further purification.

Isolated Yield: 3.46 g (>99%)

2.1.2 Preparation of Gomberg's Dimer[1]

In an ampoule wrapped in tin foil, zinc powder (1.7 g, 26.0 mmol, 5.2 equiv.) was added to a solution of triphenylmethyl chloride (1.4 g, 5.0 mmol, 1 equiv.) in toluene and stirred for five days at room temperature. The yellow solution was filtered and solvent removed under reduced pressure to yield a light-yellow solid. The light-yellow solid was dried under vacuum for two days.

Isolated Yield: 0.94 g (70%)

^{1}H NMR (400 MHz, 298 K, THF-d_8): δ= 6.90-7.56 (m, 25H, aryl CH), 6.23 (m, 2H vinylic), 5.98 (m, 2H, vinylic), 5.21 (m, 1H, allylic) ppm.

2.1.3 General procedure for preparation of *p*-$X(C_6H_4)N_3$[3]

In a round bottom flask, *p*-$X(C_6H_4)NH_2$ (1 equiv., 4 mmol; X = F, Cl, Me, H) was dissolved in MeCN and cooled to 0 °C. ^{t}Butyl nitrite (1.5 equiv. 6 mmol) was added dropwise followed by trismethylsilyl azide (1.5 equiv. 6 mmol) and the reaction warmed to room temperature and stirred for 1 hour. The solvent was removed in vacuo before passing through a silica plug using hexane. The solution was concentrated, dried using $MgSO_4$, filtered and solvent removed in vacuo yielding the corresponding azide in quantitative yield. *Caution! Covalent azides are potentially hazardous and can decompose explosively under various conditions!*

Azidobenzene:

^{1}H NMR (400 MHz, 298 K, $CDCl_3$): δ= 6.96 (dd, $^3J_{H\text{-}H}$ = 7.6 Hz, $^4J_{H\text{-}H}$ = 0.7 Hz, 2H), 7.06 (td, $^3J_{H\text{-}H}$ = 7.6, $^4J_{H\text{-}H}$ = 0.7 Hz, 1H), 7.27 (t, $^3J_{H\text{-}H}$ = 7.6 Hz, 2H) ppm.

1-azido-4-fluorobenzene:

^{1}H NMR (400 MHz, 298 K, $CDCl_3$): δ= 7.05-6.96 (m, 2H), 6.96-6.89 (m, 2H) ppm.

1-azido-4-chlorobenzene:

^{1}H NMR (400 MHz, 298 K, $CDCl_3$): δ= 7.30-7.23 (m, 2H), 6.94-6.87 (m, 2H) ppm.

1-azido-4-methylbenzene:

^{1}H NMR (400 MHz, 298 K, $CDCl_3$): δ= 7.15 (m, 2H), 6.92 (m, 2H), 2.32 (s, 3H) ppm.

2.2. Synthesis and Characterization Data of [K(crypt)][1]

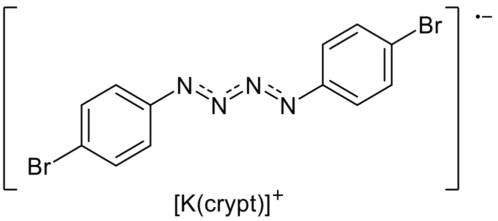

In the glovebox, KC_8 (10 mg, 0.074 mmol, 1 equiv.) and 2.2.2-cryptand (crypt; 28 mg, 0.074 mmol, 1 equiv.) were suspended in THF in a vial. 4-$BrC_6H_4N_3$ (19 uL, 0.148 mmol, 2 equiv.) was added and the vial shaken for 30 seconds. The solution was filtered and diethyl ether added to precipitate a black solid. The solid was filtered and washed with diethyl ether before drying under vacuum yielding [K(crypt)][**1**] as a black crystalline solid. Single crystals were obtained by slow vapour diffusion of hexane into THF at –40 °C. A mortar and pestle were used to grind the crystalline powder to a fine powder in the glovebox for use in powder X-ray diffraction studies.

Isolated Yield: 38.3 mg, 66%

At 15 x scale (2.22 mmol) Isolated Yield = 57%

At 30 x scale (4.44 mmol) Isolated Yield = 44%

Mass Spectrometry[32]: [[**1**]+H]$^-$ Found 366.9208 Calculated 366.9199

Elemental Analysis for $C_{34}H_{54}Br_2KN_6O_7$ [K(crypt)][1]•Et_2O: Expected: 47.61, 6.35, 9.80 Found: 47.44, 6.10, 9.68

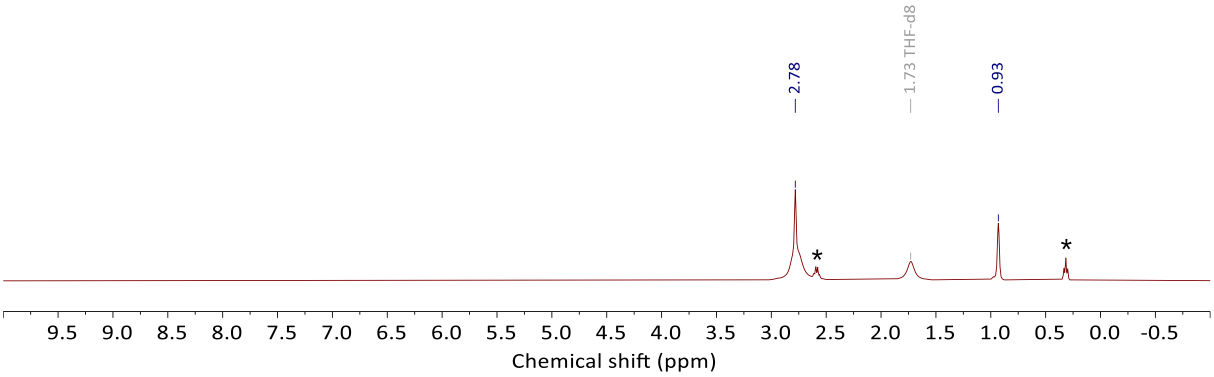

Supplementary Figure 1: ^{1}H NMR spectrum (400 MHz, THF-d_8) of [K(crypt)][**1**]. Broadened K(crypt) signals due to presence of paramagnetic [**1**]$^{•-}$. Residual diethyl ether solvent identified with *.

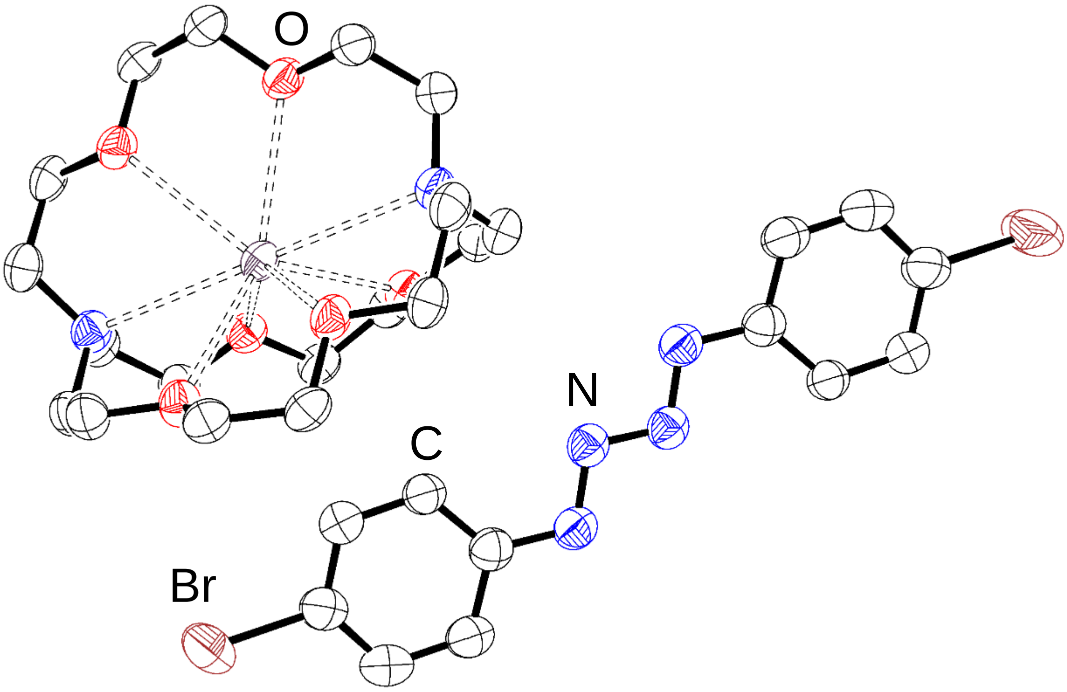

Supplementary Figure 2: Molecular structure of [K(crypt)][**1**] showing anisotropic displacement ellipsoids at 50% probability. Hydrogen atoms omitted for clarity. Nitrogen: blue; carbon: white; bromine: brown; potassium: violet; oxygen: red.

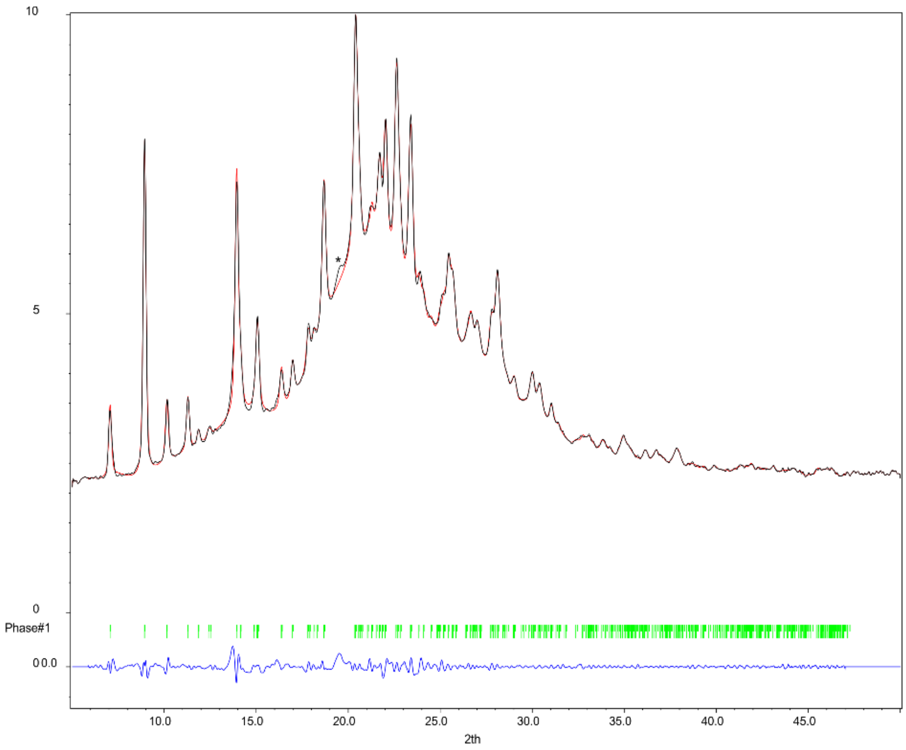

Supplementary Figure 3: Experimental (black) and simulated (red) powder x-ray diffraction (PXRD) patterns of [K(crypt)][**1**] (minor impurity highlighted with *).

2.3 Functional benchmarking against geometric structure

Supplementary Table 1: Selected experimental and calculated bond lengths for [**1**]$^{\bullet-}$ for a variety of functionals using def2-TZVP basis set.[33, 34, 35]

**Method**	**N1–N2 (Å)**	**N2–N2' (Å)**
Experimental	1.316(4)	1.322(6)
PBE	1.314	1.329
PBE0	1.297	1.307
TPSS	1.318	1.329
TPSSh	1.310	1.319
ωB97XD	1.297	1.308

2.4 Calculated Bond Metric Data

Supplementary Table 2: Selected experimental and calculated bond lengths and bond angles of [**1**]$^{\bullet-}$ with varying solvents/counterions at TPSS/def2-TZVP level of theory.

**Structure/Solvent**	**N1–N2 (Å)**	**N2–N2' (Å)**	**∠C1–N1–N2 (°)**	**∠N1–N2–N2' (°)**
Experimental	1.316(4)	1.322(6)	111.8(3)	110.2(4)
[**1**]$^{\bullet-}$/Gas Phase	1.318	1.329	112.5	109.9
[**1**]$^{\bullet-}$ /THF	1.319	1.329	113.2	110.1
[K(crypt)][**1**] /THF	1.318	1.330	113.3	110.2
[**1**]$^{\bullet-}$ /cyclopentanone	1.319	1.330	113.4	110.1
[K(crypt)][**1**] /cyclopentanone	1.318	1.330	113.4	110.2

Supplementary Table 3: Calculated Wiberg Bond Index (WBI) for [**1**]$^{\bullet-}$ (no counterion) at TPSS/def2-TZVP /SMD level of theory.

**Solvent**	**N1–N2**	**N2–N2'**	**N1'–N2'**
THF	1.430	1.386	1.430
Cyclopentanone	1.428	1.386	1.428

It was found that the addition of the counter ion [K(crypt)]$^{+}$ and choice of solvent had no significant impact on the optimized geometry. Thus, all subsequent DFT data in the manuscript and supplementary information were collected on structures optimized without the counter ion, and using whatever solvent best matched experiment (solvent choice is provided alongside the level of theory, where applicable)

Supplementary Table 4: Select results from NLMO analysis of [K(crypt)][**1**] at TPSS/def2-TZVP level of theory.

NLMO (R1–R2)	% R1[a]	% R2[b]	R1 %s[c]	R1 %p[d]	R1 hybridization[e]	R2 %s[f]	R2 %p[g]	R2 hybridization[h]
N1 – C1 (σ)	56.0	43.1	30.7	69.0	sp2.24	30.6	69.3	sp2.27
N1 – C1 (π)	65.3	23.2	0	99.8	p	0	97.4	p
N1 – N2 (σ)	49.5	49.7	27.0	72.9	sp2.70	27.3	72.5	sp2.66
N1 (LP)	97.0	NA	43.8	56.1	sp1.28	NA	NA	NA
N2 – N2' (σ)	49.6	49.6	26.5	73.3	sp2.77	26.5	73.3	sp2.77
N2 (LP)	98.3	NA	48.9	51.0	sp1.04	NA	NA	NA

[a] R1-atom contribution to the specified NLMO. [b] R2-atom contribution to the specified NLMO. [c] %s character of the R1-atom contribution to the NLMO. [d] %p character of the R1-atom contribution to the NLMO. [e] Hybridization of R1-atom in specified bond NLMO. [f] %s character of the R2-atom contribution to the NLMO. [g] %p character of the R2-atom contribution to the NLMO. [h] Hybridization of R2-atom in specified bond NLMO.

2.5 Charge Delocalization Data

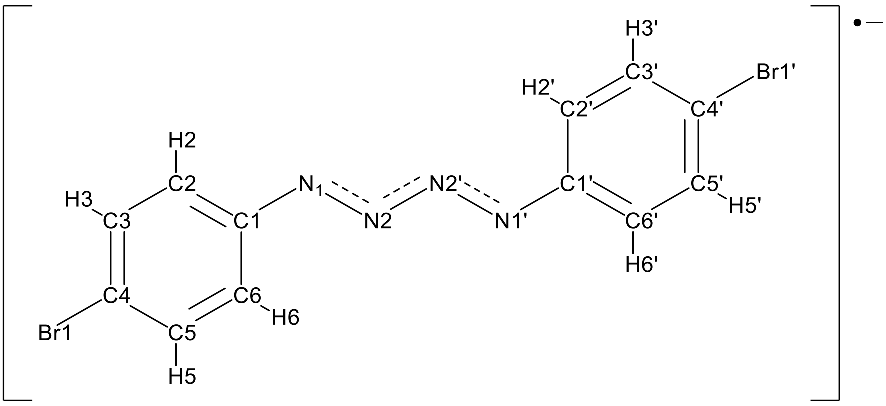

Supplementary Table 5: Calculated NPA, Hirshfeld, Löwdin, and Mulliken charge distributions for [**1**]$^{\bullet-}$ at TPSS/def2-TZVP level of theory.

Atom	Charge Distribution			
	NPA	Hirshfeld	Löwdin	Mulliken
Br1	0.000	–0.087	0.281	–0.172
N1	–0.287	–0.199	–0.035	–0.088
N2	–0.107	–0.127	–0.110	–0.167
C2	–0.166	–0.060	–0.127	–0.228
H2	0.117	0.030	0.144	0.096
C3	–0.100	–0.054	–0.160	–0.191
H3	0.112	0.045	0.150	0.135
C4	–0.103	–0.019	–0.385	0.176
C5	–0.106	–0.057	–0.162	–0.177
H5	0.113	0.045	0.151	0.132
C6	–0.143	–0.056	–0.118	–0.229
H6	0.113	0.039	0.149	0.095
C1	0.057	0.000	–0.278	0.112
Br1’	0.000	–0.087	0.281	–0.171
N1’	–0.287	–0.199	–0.035	–0.087
N2’	–0.107	–0.127	–0.110	–0.155
C2’	–0.166	–0.060	–0.127	–0.219
H2’	0.117	0.030	0.144	0.094
C3’	–0.100	–0.054	–0.160	–0.193
H3’	0.112	0.045	0.150	0.134
C4’	–0.103	–0.019	–0.385	0.176

C5’	–0.106	–0.057	–0.162	–0.175
H5’	0.113	0.045	0.151	0.132
C6’	–0.143	–0.056	–0.118	–0.238
H6’	0.113	0.039	0.149	0.095
C1’	0.057	0.000	–0.278	0.114
Total	–1.000	–1.000	–1.000	–1.000

Supplementary Table 6: Calculated NPA, Hirshfeld, and Mulliken charge distributions for neutral $(4\text{-}BrC_6H_4)_2N_4$ at TPSS/def2-TZVP level of theory.

Atom	Charge Distribution		
	**NPA**	**Hirshfeld**	**Mulliken**
Br1	0.068	–0.030	–0.107
N1	–0.169	–0.083	0.012
N2	–0.097	–0.059	–0.105
C2	–0.165	–0.020	–0.173
H2	0.243	0.053	0.126
C3	–0.232	–0.030	–0.167
H3	0.241	0.059	0.153
C4	–0.045	0.015	0.187
C5	–0.240	–0.033	–0.158
H5	0.242	0.060	0.151
C6	–0.141	–0.015	–0.173
H6	0.239	0.061	0.128
C1	0.057	0.022	0.125
Br1’	0.068	–0.030	–0.107
N1’	–0.169	–0.083	0.012
N2’	–0.097	–0.059	–0.105
C2’	–0.165	–0.020	–0.173
H2’	0.243	0.053	0.126
C3’	–0.232	–0.030	–0.167
H3’	0.241	0.059	0.153
C4’	–0.045	0.015	0.187
C5’	–0.240	–0.033	–0.158
H5’	0.242	0.060	0.151
C6’	–0.141	–0.015	–0.173
H6’	0.239	0.061	0.128
C1’	0.057	0.022	0.125
Total	0.000	0.000	0.000

2.6 Molecular Orbital Diagram

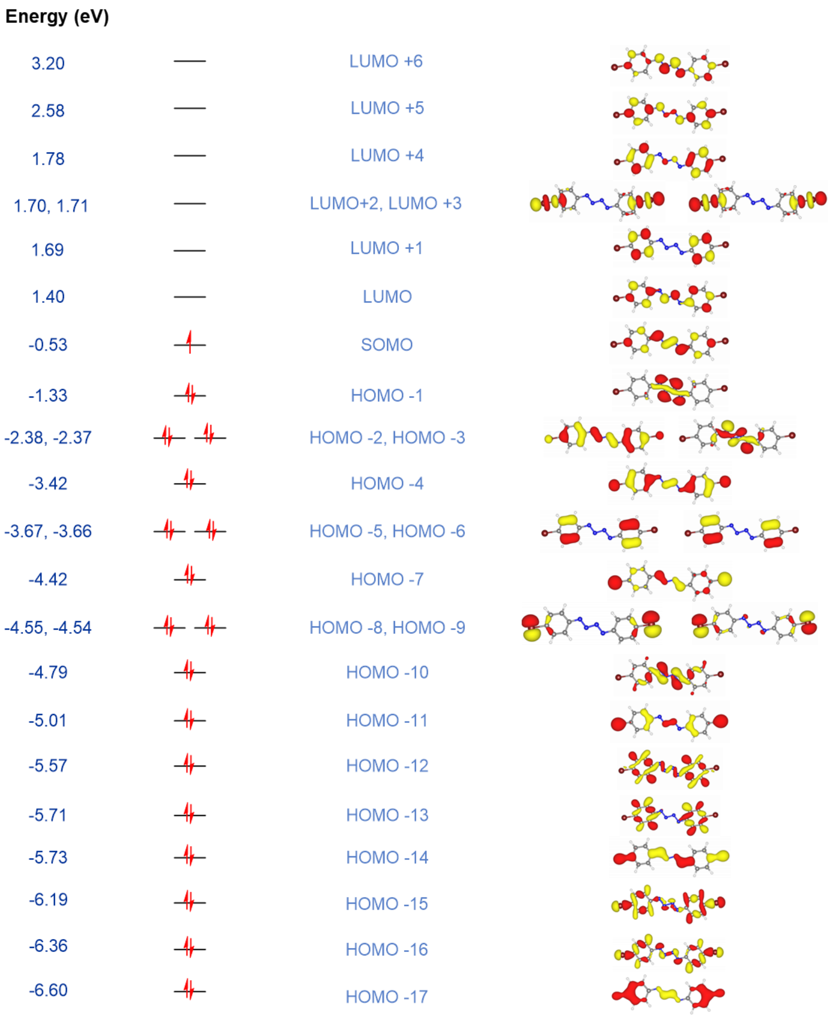

Supplementary Figure 4: Molecular orbital diagram of $[\mathbf{1}]^{\bullet-}$ from HOMO-17 to LUMO+6 with isovalue of 0.04. Kohn-Sham orbitals were calculated at the Def2-TVZP/TPSS level of theory.

2.7 EPR of [K(crypt)][1]

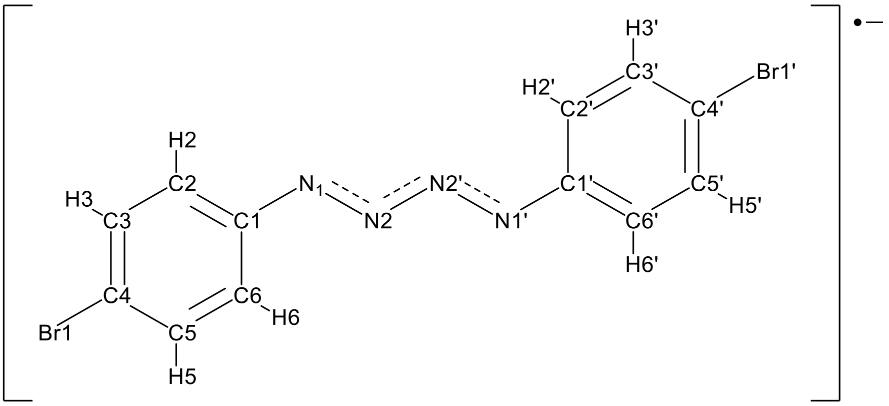

2.7.1 EPR of [K(crypt)][1] stacked with a simulation of $[\mathbf{1}]^{\bullet-}$ using calculated hyperfine values.

Supplementary Table 7: Calculated A_{iso} values of $[\mathbf{1}]^{\bullet-}$ at EPR-III (aug-cc-PVTZ for Br)/B3LYP/SMD(THF) level of theory.

Atom	A_{iso} (MHz)	Atom	A_{iso} (MHz)
N1	13.0996	N1’	13.1001
N2	0.0948	N2’	0.0953
H2	–7.0883	H2’	–7.0882
H3	3.2410	H3’	3.2407
H5	3.3310	H5’	3.3308
H6	–8.2026	H6’	–8.2018
Br1	1.4442	Br1’	1.4444

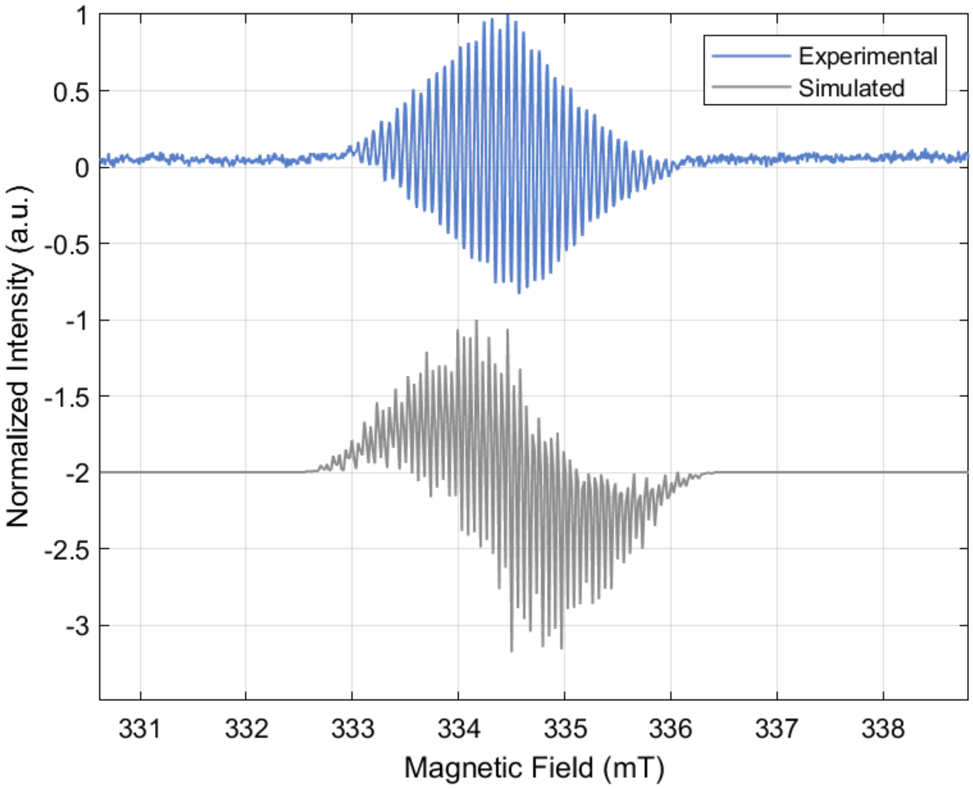

Supplementary Figure 5: Continuous wave EPR spectrum of [K(crypt)[**1**] stacked above the simulated spectrum of [**1**]$^{•-}$ using the above calculated hyperfine values from Supplementary Table 7. Experimental parameters: solvent: THF, frequency: 9.390 GHz, temperature: 298 K, modulation amplitude: 0.1 G, scans: 2, gain: 30 dB.

2.7.2 EPR of [K(crypt)][1] stacked with an optimized simulation

Hyperfine values from the DFT were optimized to get a more accurate simulation. Inclusion of the smaller nitrogen hyperfine makes no visible change as it is within the linewidth of the spectrum, but the optimized value was still included. These parameters closely reproduce the experimentally measured spectrum indicating that these are the most important interactions (See Supplementary Figure 6 and Supplementary Figure 7).It should be noted that due to overlap in the hyperfine lines it is possible to reasonably simulate the spectrum using more than one set of hyperfine values.

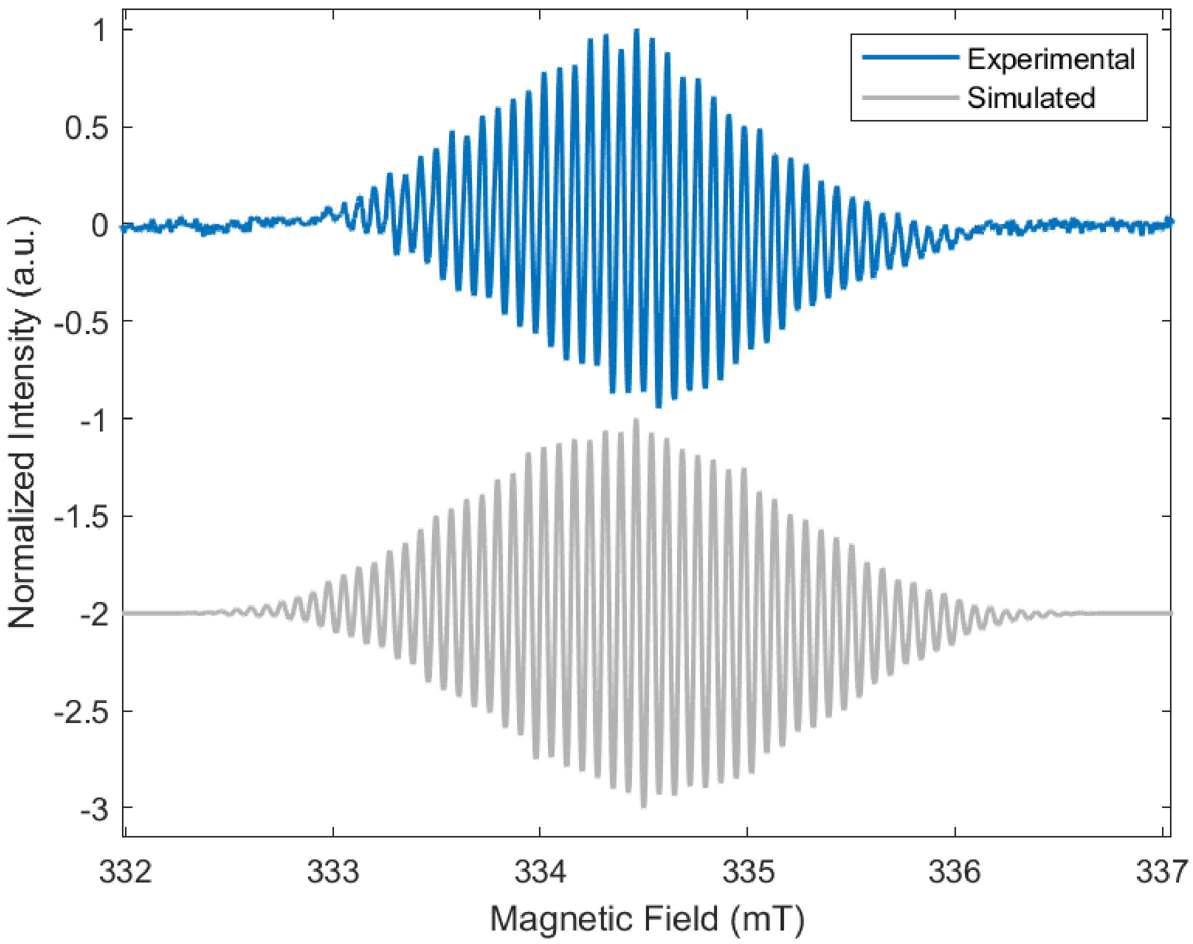

Supplementary Figure 6: Continuous wave EPR spectrum of [K(crypt)][**1**] (blue) stacked above the simulated spectrum (grey, ×2 A_N = 14.61 MHz, ×2 A_N = 0.23 MHz, ×4 A_H = 8.51 MHz, ×4 A_H = 3.70 MHz, ×2 A_{Br} = 2.05 MHz, g = 2.006, lw = 0.04). Experimental parameters: solvent: THF, frequency: 9.390 GHz, temperature: 298 K, modulation amplitude: 0.1 G, scans: 2, gain: 30 dB.

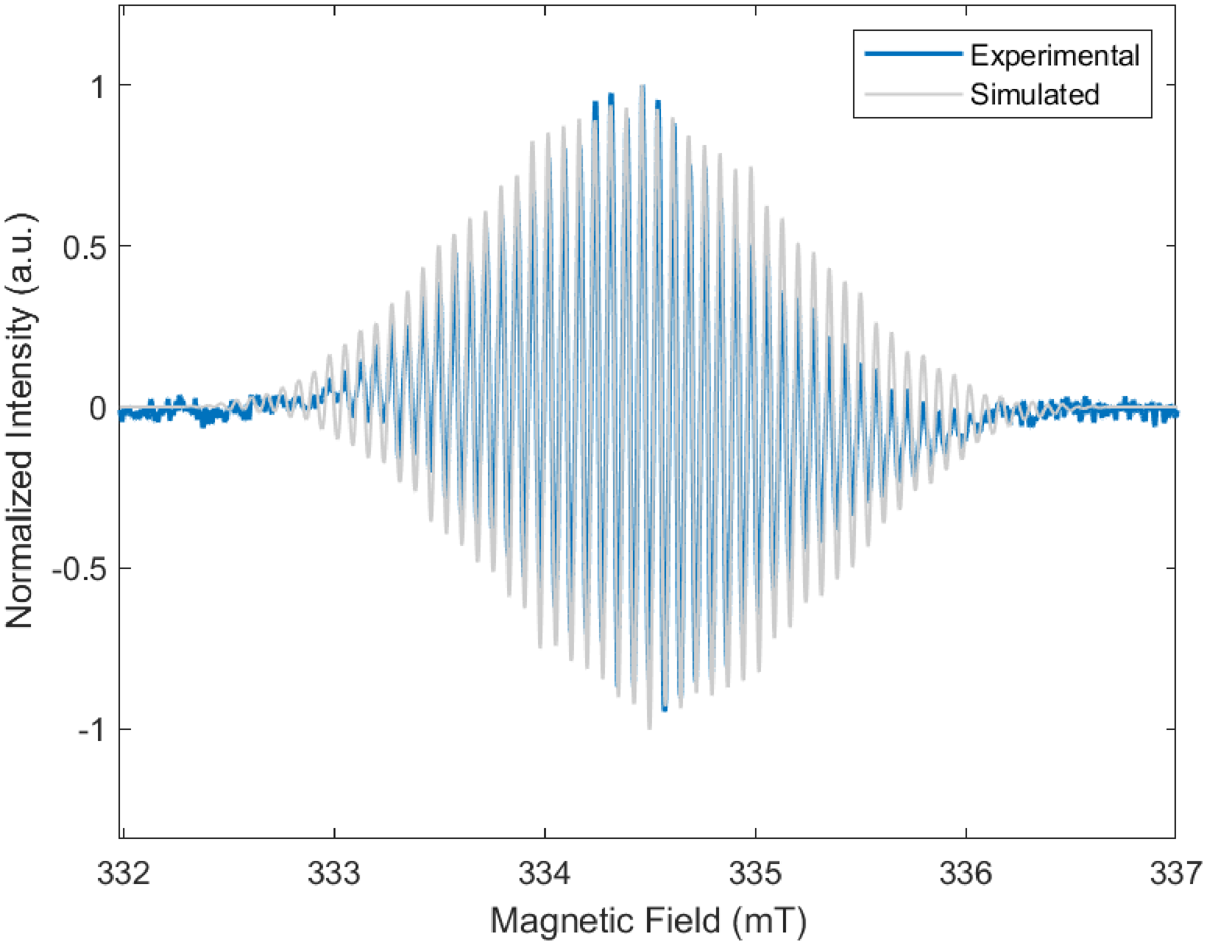

Supplementary Figure 7: Continuous wave EPR spectrum of [K(crypt)][**1**] (blue) overlayed with the simulated spectrum (grey, ×2 A_N = 14.61 MHz, ×2 A_N = 0.23 MHz, ×4 A_H = 8.51 MHz, ×4 A_H = 3.70 MHz, ×2 A_{Br} = 2.05 MHz, g = 2.006, lw = 0.04). Experimental parameters: solvent: THF, frequency: 9.390 GHz, temperature: 298 K, modulation amplitude: 0.1 G, scans: 2, gain: 30 dB.

2.8 Calculated Spin Densities of [1]$^{•−}$

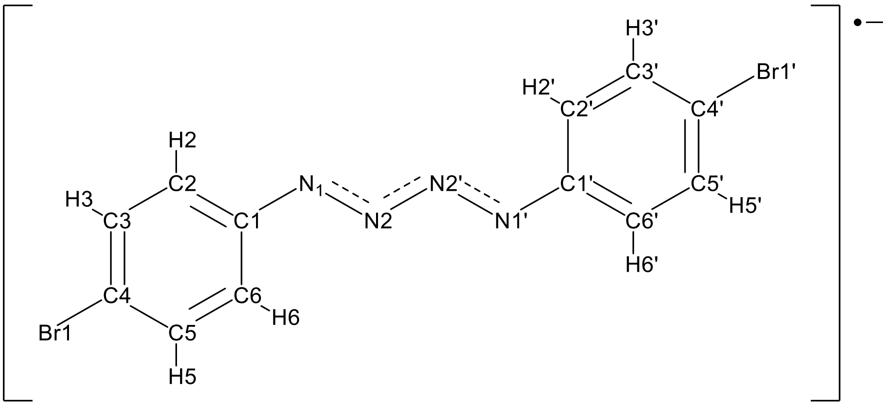

Supplementary Table 8: Calculated Hirshfeld, Löwdin, and Mulliken spin densities for [**1**]$^{•−}$ at TPSS/def2-TZVP level of theory.

Atom	Spin Number		
	Hirshfeld	Löwdin	Mulliken
Br1	0.018	0.016	0.009
N1	0.226	0.228	0.263
N2	0.066	0.063	0.049
C2	0.067	0.068	0.114
H2	0.005	0.000	–0.007
C3	–0.011	–0.009	–0.047
H3	–0.001	0.000	0.002
C4	0.075	0.074	0.113
C5	–0.010	–0.008	–0.042
H5	–0.001	0.000	0.002
C6	0.058	0.056	0.091
H6	0.003	0.000	–0.004
C1	0.005	0.011	–0.040
Br1’	0.018	0.016	0.009
N1’	0.226	0.228	0.262
N2’	0.066	0.063	0.048
C2’	0.067	0.068	0.111
H2’	0.005	0.000	–0.006
C3’	–0.011	–0.009	–0.046
H3’	–0.001	0.000	0.002
C4’	0.075	0.074	0.111

C5’	–0.010	–0.008	–0.042
H5’	–0.001	0.000	0.002
C6’	0.058	0.056	0.090
H6’	0.003	0.000	–0.004
C1’	0.005	0.011	–0.040
Total	1.000	1.000	1.000

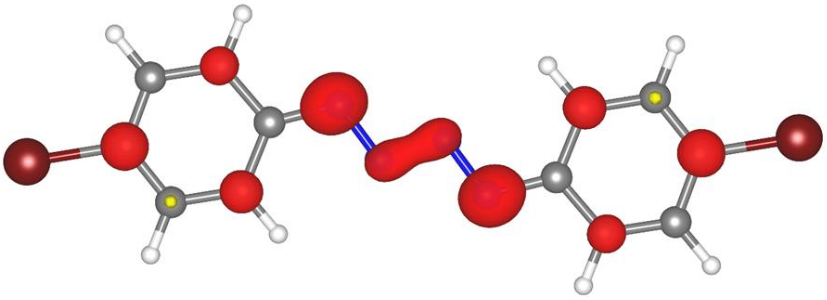

Supplementary Figure 8: Spin density plot (front-on) of [**1**]$^{\bullet-}$ with isovalue = 0.005.

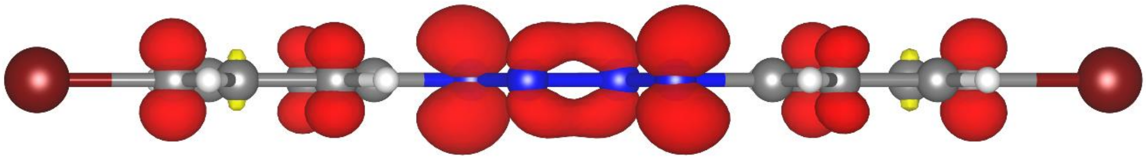

Supplementary Figure 9: Spin density plot (side-on) of [**1**]$^{\bullet-}$ with isovalue = 0.005.

2.9 Spin Counting of [K(crypt)][1]

To further confirm purity, a spin counting experiment was done.

3.7mg of [K(crypt)][**1**] was dissolved in 2 mL 50:50 mixture of *o*DFB : toluene. A 0.5 mL aliquot was taken and diluted with 1.5 mL of 50:50 *o*DFB : toluene to make a 0.59 mM sample. 0.3 mL was added to a J Young EPR tube in order to fill the whole resonator and immediately frozen in liquid nitrogen until it was ready to be measured. A spin number of 3 x 10^{16} was calculated indicating a radical concentration of 0.586 mM using the equation from the calibration curve trendline which was measured using a standard sample of TEMPO diluted to different concentrations. This result shows that there is near complete conversion and retention of the radical form [K(crypt)][**1**].

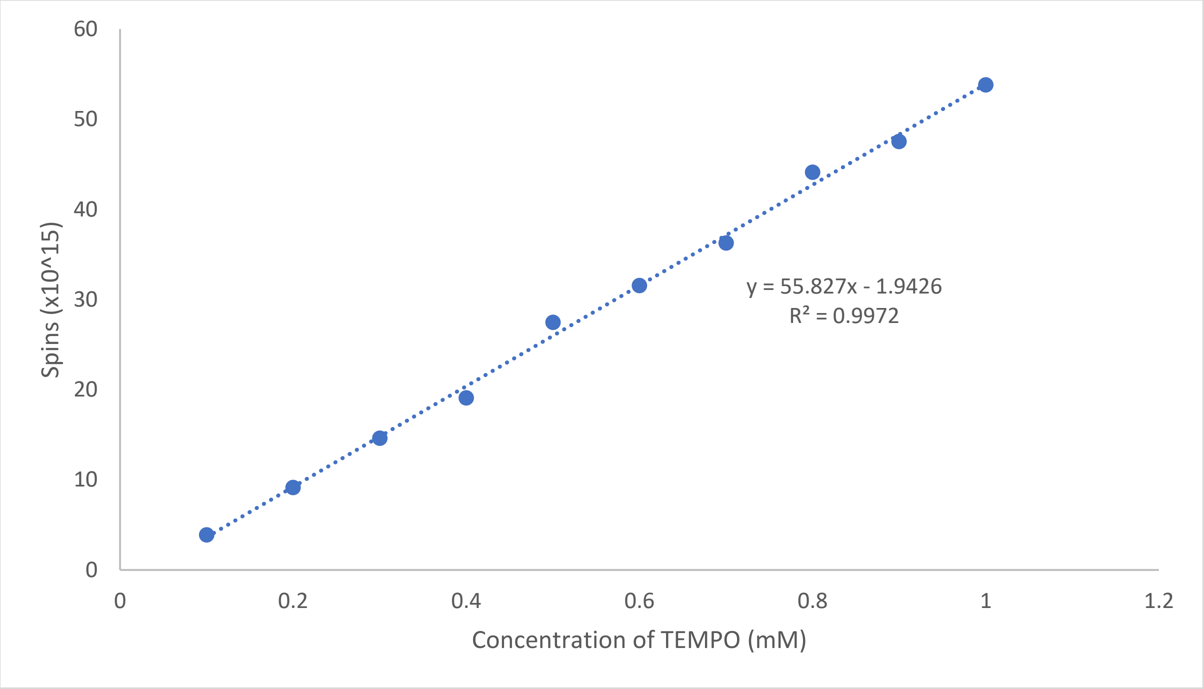

Supplementary Figure 10: Calibration curve for spin counting with a varying TEMPO concentration; equation of the straight line used to calculate spins in [K(crypt)][**1**] is shown.

2.10 Assessing Stability of [K(crypt)][1]

To assess the effect of solvents on the half-life of [K(crypt)][**1**], a 2D continuous wave EPR technique was used where a spectrum is collected in consistent intervals and the max peak intensity of spectra can be tracked over time and plotted as a decay curve.

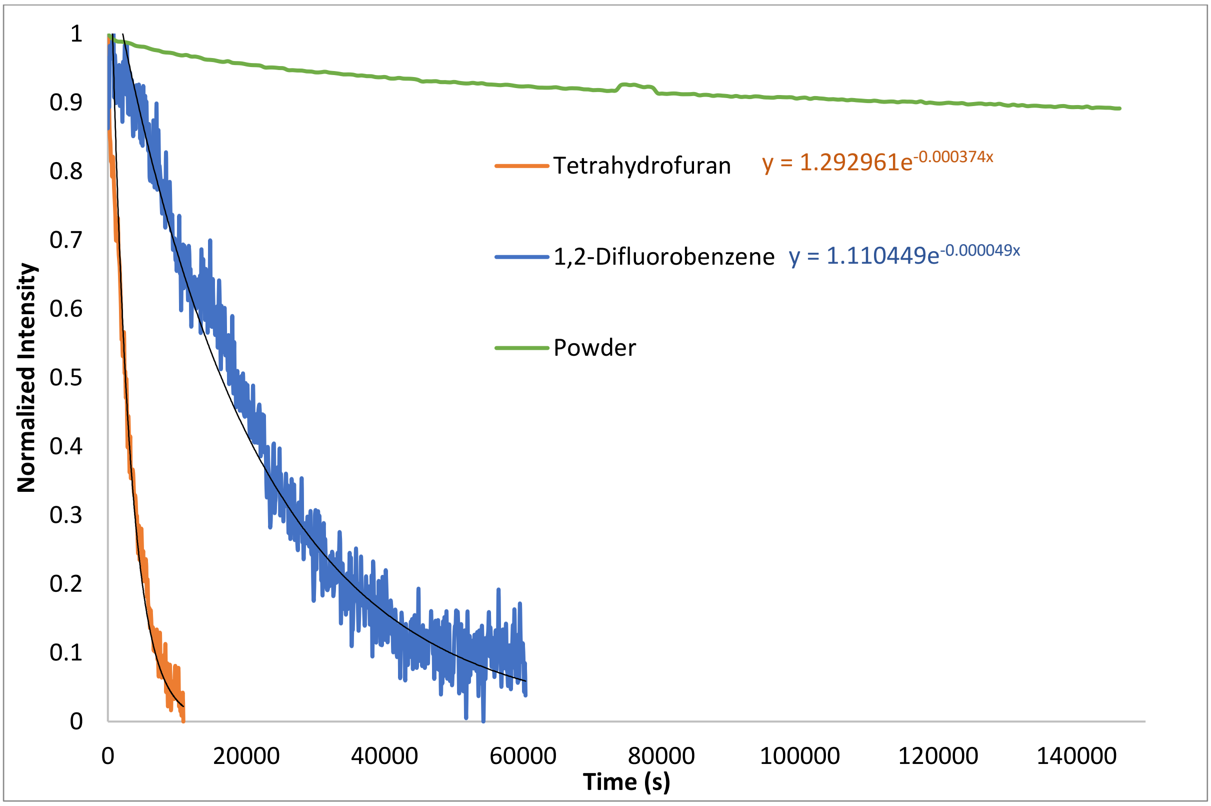

Supplementary Figure 11: Decay curve of the EPR signal intensity for [K(crypt)][**1**] in THF (orange), *o*DFB (blue) and powder (green) with the exponential equation shown beside the legend.

Supplementary Table 9: Calculated half-life of [K(crypt)][**1**] in THF and *o*DFB using exponential decay curve equation.

**Solvent**	**Half-life (s)**
THF	2540.3 (42.3 mins)
*o*DFB	16283.9 (271.4 mins)

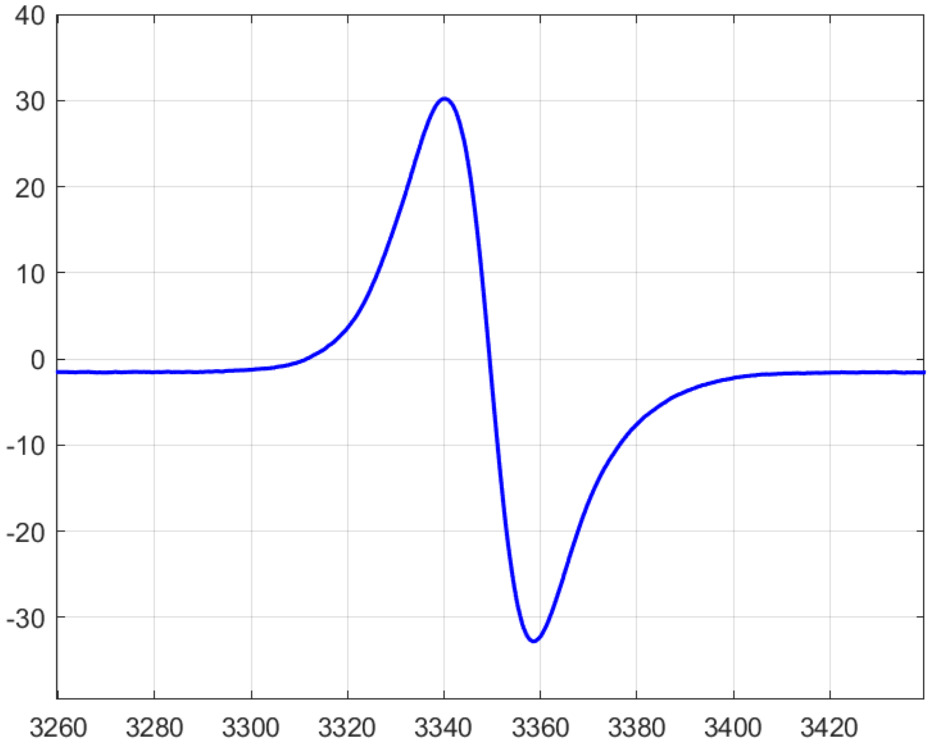

Supplementary Figure 12: Continuous wave EPR spectrum (solid state) of [K(crypt)][**1**] after 6 weeks under inert atmosphere at room temperature.

2.11 Cyclic Voltammetry of [K(crypt)][1]

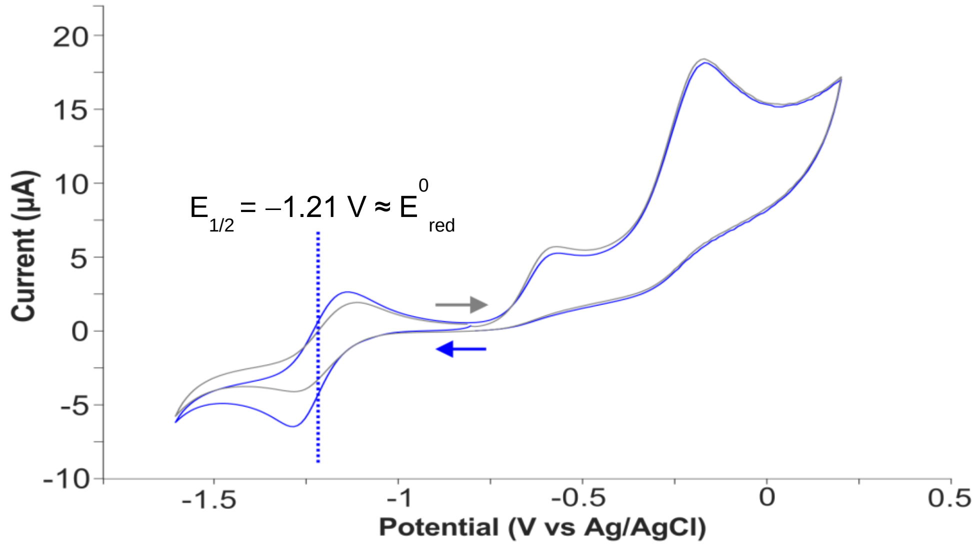

Supplementary Figure 13: Cyclic Voltammetry of [K(crypt)][**1**] 3 mM in THF with [nBu_4N][PF_6] electrolyte at 0.1 V/s starting at –0.8 V and scanning independently in the positive direction first (grey trace) and the negative direction first (blue trace). Glassy carbon working electrode, platinum wire counter electrode and leak-proof Ag/AgCl reference electrode were used.

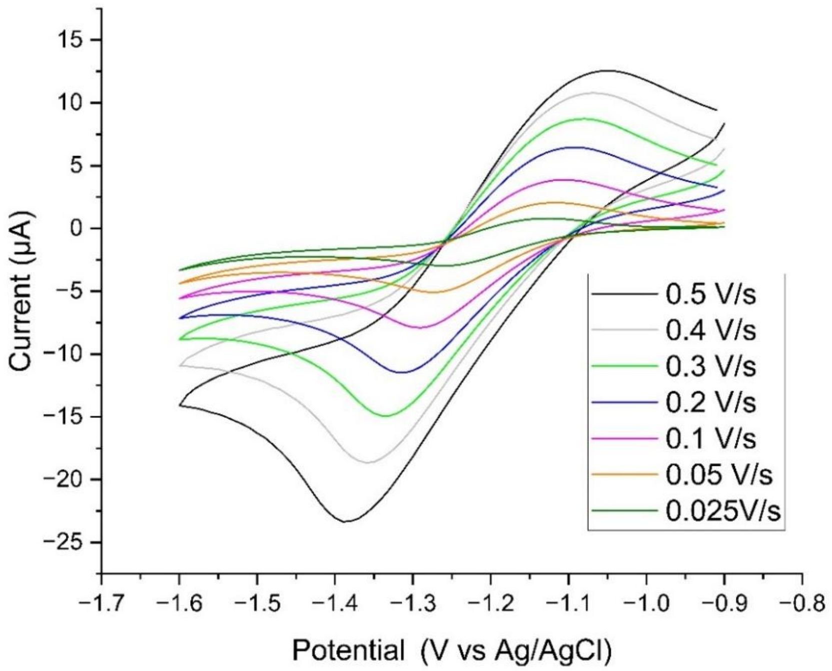

Supplementary Figure 14: Cyclic Voltammetry of [K(crypt)][**1**] 3 mM in THF with [nBu_4N][PF_6] electrolyte at varying scan rates on first reduction wave using glassy carbon working electrode, platinum wire counter electrode and leak-proof Ag/AgCl reference electrode.

2.12 Infrared Spectroscopy of [K(crypt)][1]

When comparing the IR of [K(crypt)][**1**] to [K(crypt)][Cl] (Supplementary Figure 17, zoomed in), we can see a major new peak at 1236 cm^{-1} which is between the stretching frequencies of hydrazine (1077 cm^{-1}) and azobenzene (1439 cm^{-1}).[36, 37] This is believed to be associated with N–N bond stretches, and is in good agreement with the calculated IR stretches (1272 cm^{-1}, TPSS/def2-TZVP level of theory, Supplementary Figure 18 and S19).

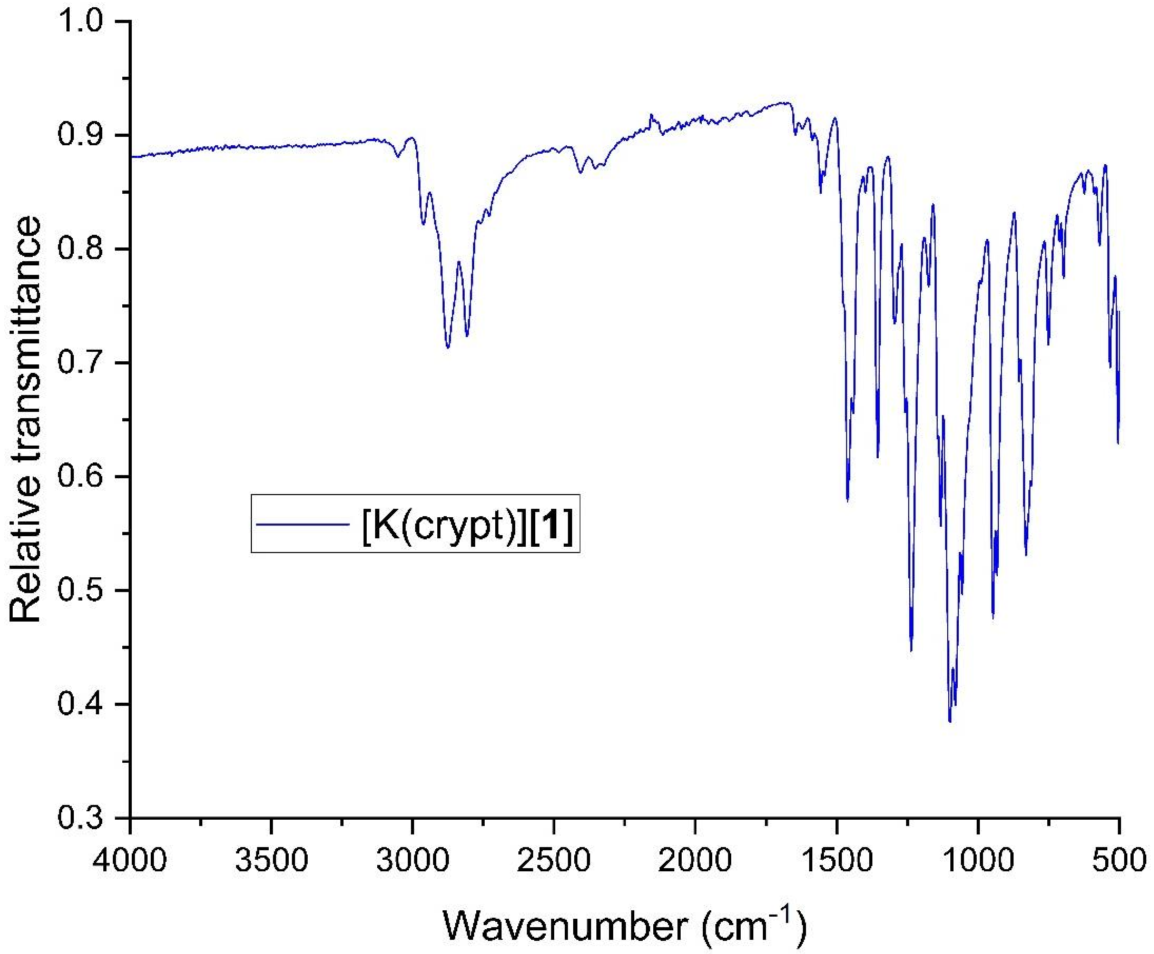

Supplementary Figure 15: Full solid state IR spectrum of [K(crypt)][**1**]

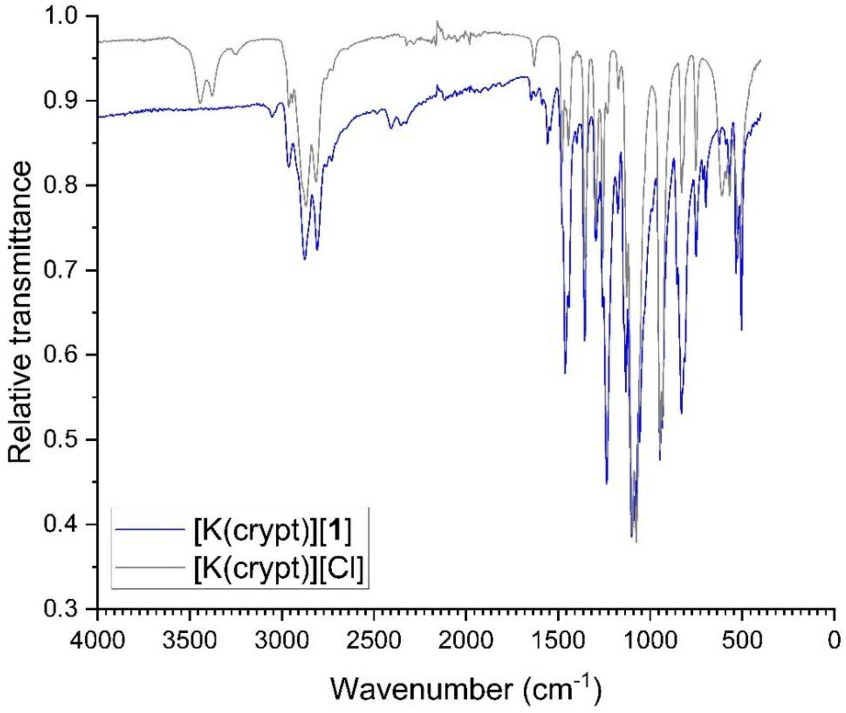

Supplementary Figure 16: Solid state IR spectrum of [K(crypt)][**1**] overlayed with independently prepared [K(crypt)][Cl].

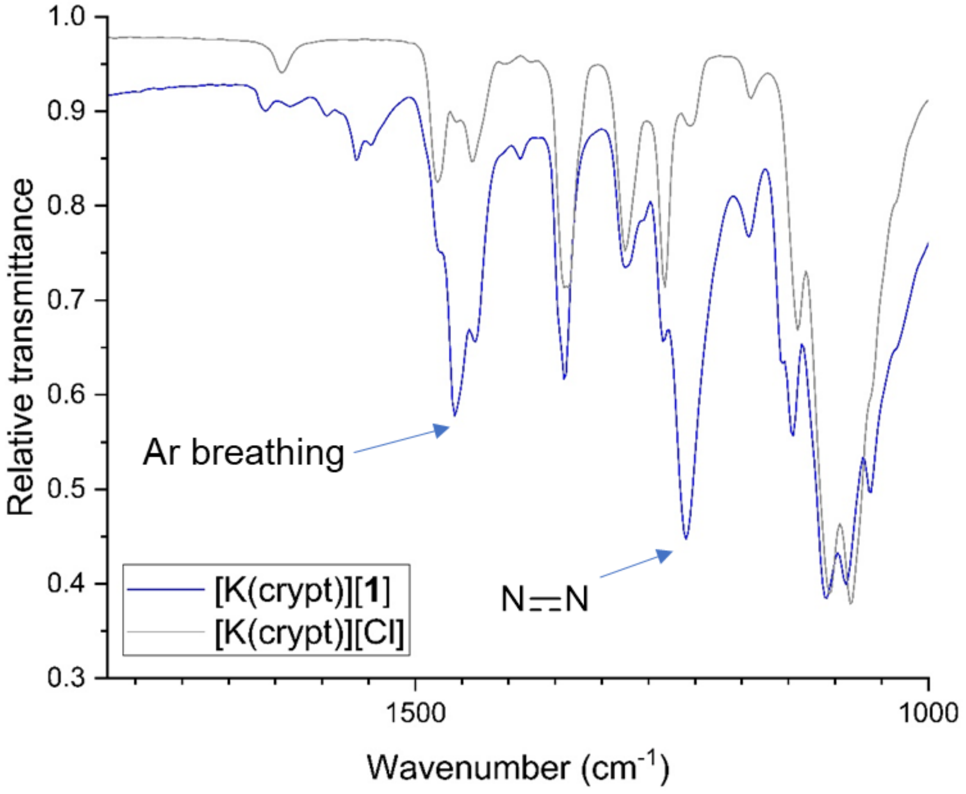

Supplementary Figure 17: Zoomed in solid state IR spectrum of [K(crypt)][**1**] overlayed with independently prepared [K(crypt)][Cl] with key peaks from [**1**]$^{\bullet-}$ highlighted.

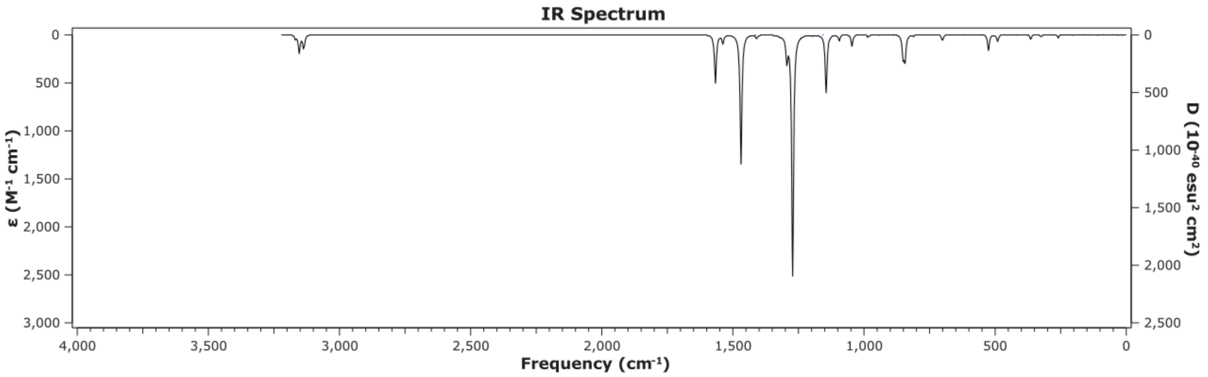

Supplementary Figure 18: Calculated IR spectrum of [**1**]$^{\bullet-}$ at TPSS/def2-TZVP level of theory.

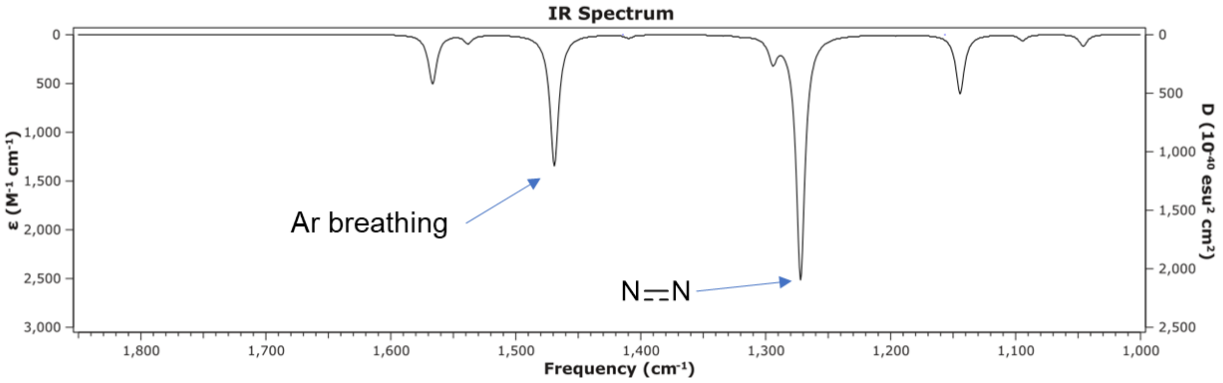

Supplementary Figure 19: Zoomed in calculated IR spectrum of [**1**]$^{\bullet-}$, with key peaks highlighted, at TPSS/def2-TZVP level of theory.

2.13 Ultraviolet-Visible Spectroscopy of [K(crypt)][1]

2.13.1 Observed UV-Vis Spectra of [K(crypt)][1]

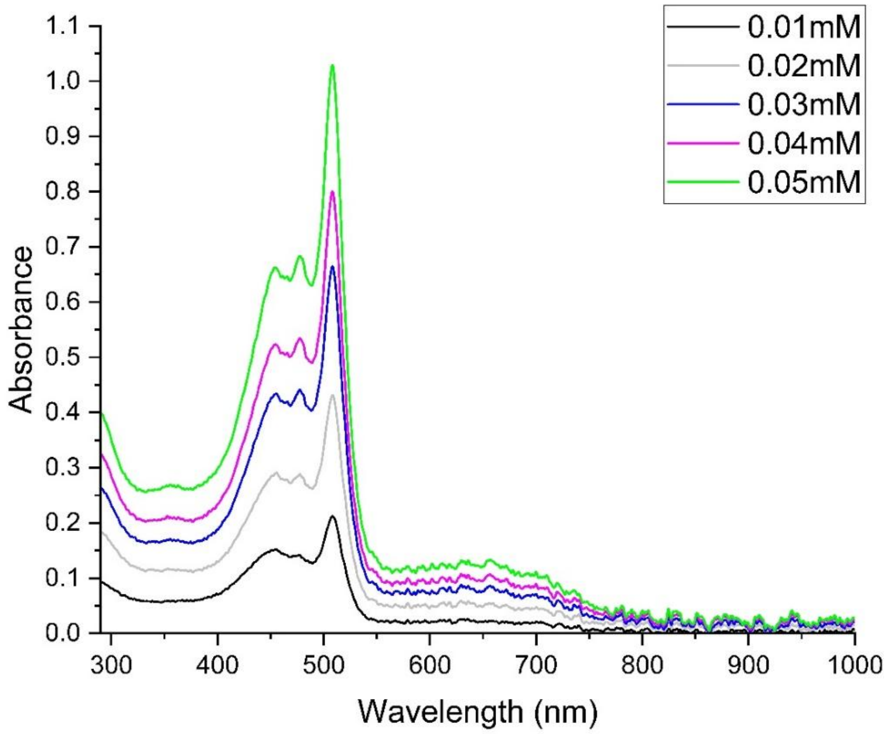

Supplementary Figure 20: UV-Vis spectra of [K(crypt)][**1**] at a range of concentrations from 0.01 mM to 0.05 mM in *o*DFB.

2.13.2 Calculated UV-Vis Transitions

To characterize the UV-Vis absorption spectrum of $[\mathbf{1}]^{\bullet-}$, TD-DFT calculations were performed on the geometry previously optimized at TPSS/def2-TZVP/SMD(THF), using a variety of functionals (Supplementary Table 10 below). The key transitions found experimentally at 355 nm, 454 nm, 508 nm and 612 nm were best reproduced by the TPSSh functional. The orbital make-up of the transitions is complicated at the Kohn-Sham level, so to better understand the origin of the computed transitions, the Natural Transition Orbitals (NTOs) associated with each transition were calculated, revealing that all four of the excited states were underpinned by transitions 90α → 91α (blue dot) and 89β → 90β (grey dot), with different occupations (Supplementary Table 11).

Supplementary Table 10: Experimental UV-Vis wavelengths and calculated oscillator strengths for transitions to selected excited states for $[\mathbf{1}]^{\bullet-}$ for a variety of functionals and solvents (experimental absorbance is compared with computed oscillator strengths, calculated at def2-TZVP level of theory).

**Method/ Solvent**	**Excited State 1**		**Excited State 2**		**Excited State 3**		**Excited State 4**	
	**Wavelength (nm)**	**Oscillator Strength**	**Wavelength (nm)**	**Oscillator Strength**	**Wavelength (nm)**	**Oscillator Strength**	**Wavelength (nm)**	**Oscillator Strength**
**Experimental/ *o*DFB**	~355	~0.27	~454	~0.66	~508	~1.03	~612	~0.13
**PBE/THF**	378.66	0.019	487.62	1.25	538.76	0.316	663.19	0.001
**PBE0/THF**	314.45	0.017	406.30	0.195	474.87	1.379	579.45	0.034
**TPSS/THF**	366.43	0.023	473.70	1.05	515.92	0.538	645.59	0.004
**TPSSh/THF**	337.60	0.049	441.72	0.437	487.76	1.167	612.00	0.013
**B3LYP/THF**	329.14	0.067	423.99	0.250	484.42	1.320	598.67	0.013
**CAM-B3LYP/THF**[38]	282.68	0.011	360.88	0.112	466.01	1.357	540.96	0.072
**B3PW91/THF**[26]	328.16	0.063	421.27	0.249	480.28	1.338	593.67	0.019
**M06-2X/THF**[39]	299.75	0.068	363.24	0.122	453.07	1.481	527.85	0.003
**ωB97XD/THF**	278.78	0.020	355.24	0.100	460.52	1.399	533.90	0.057
**TPSS/cyclopentanone**	369.58	0.025	472.16	0.892	513.84	0.693	646.76	0.007
**TPSSh/cyclopentanone**	339.86	0.049	437.56	0.358	488.29	1.237	612.89	0.017
**B3LYP/cyclopentanone**	329.98	0.074	419.47	0.217	485.66	1.343	599.64	0.017

Supplementary Table 11: TD-DFT calculated UV wavelengths, oscillator strengths, natural transition orbitals (NTOs) and their occupations with blue and grey dots for selected excited states of $[\mathbf{1}]^{\bullet-}$ (calculated at TPSSh/def2-TZVP/SMD(cyclopentanone) level of theory).

Excited State	Wavelength	Oscillator Strength	NTO Occupation Number		
1	339.86	0.049	0.201	1.586	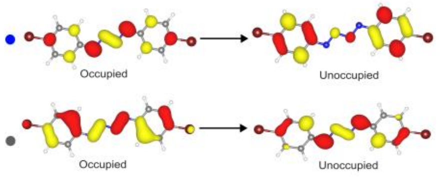
2	437.56	0.359	1.822	0.127	
3	488.28	1.237	1.141	0.818	
4	612.89	0.0170	0.904	1.069	

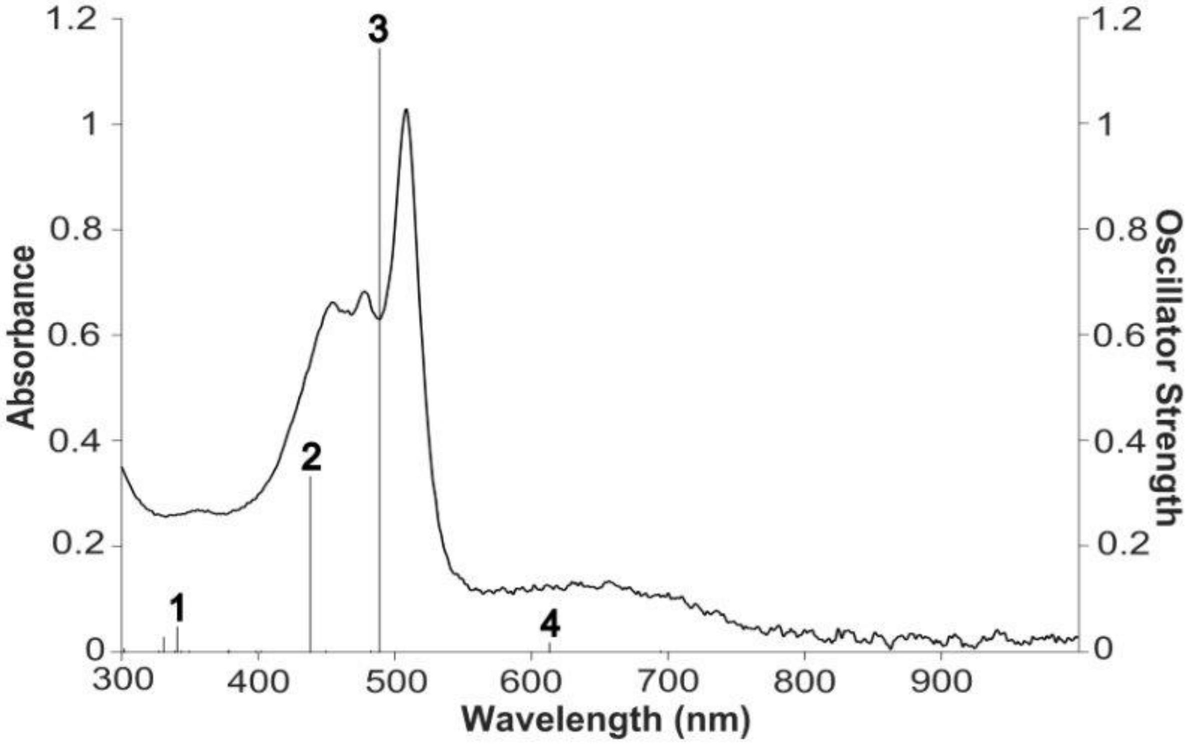

Supplementary Figure 21: Overlayed UV-Vis spectrum and oscillator strengths of the calculated spectrum of $[\mathbf{1}]^{\bullet-}$ at TPSSh/def2-TZVP/SMD(cyclopentanone) level of theory.

3. Synthesis and Characterization of [K(crypt)][3]

3.1 Synthesis of [K(crypt)][3]

In the glovebox, KC_8 (100 mg, 0.74 mmol, 1 equiv.) and 2.2.2-cryptand (280 mg, 0.74 mmol, 1 equiv.) were suspended in THF in a J-young ampoule. 1-azido-4-fluorobenzene (203 mg, 1.48 mmol, 2 equiv.) was added to the reaction mixture, and the reaction mixture was stirred for 10 minutes. Then, the solution was filtered and diethyl ether added to precipitate a black solid. The solid was filtered and washed with additional diethyl ether (3 x10 mL) before drying under vacuum yielding [K(crypt)][**3**] as a black crystalline solid. Single crystals were obtained by slow vapor diffusion of hexane into a solution of [K(crypt)][**3**] in THF at –40 °C.

Isolated Yield: 180 mg, 36%

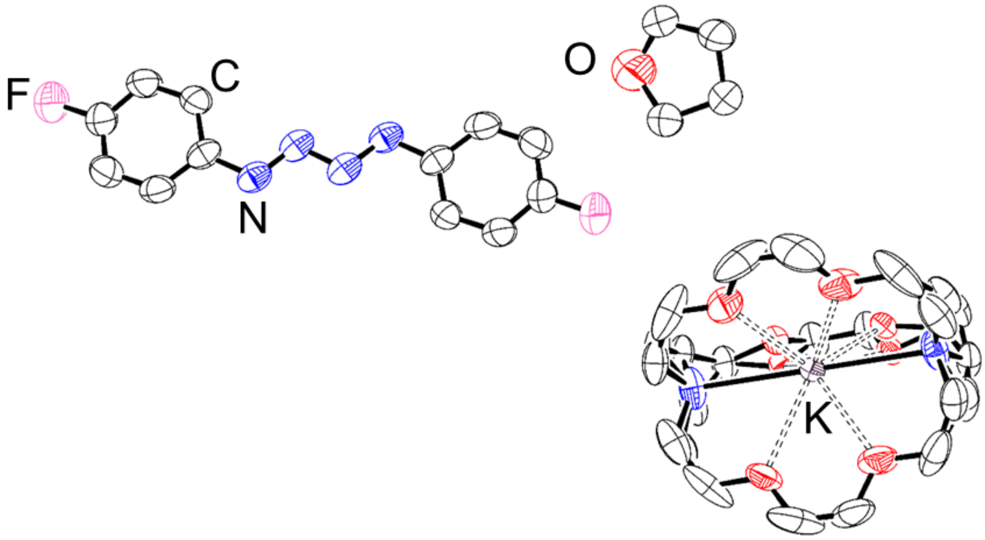

Supplementary Figure 22: Molecular structure of [K(crypt)][**3**] showing anisotropic displacement ellipsoids at 50% probability. Hydrogen atoms omitted for clarity. Nitrogen: blue; carbon: white; fluorine: pink; potassium: violet; oxygen: red.

Supplementary Table 12: Selected experimental and calculated bond lengths, bond angles, and Wiberg bond indices of [**3**]$^{\bullet-}$ calculated at TPSS/def2-TZVP/SMD(THF) level of theory.

	**N1–N2 bond length (Å)**	**N2–N2' bond length (Å)**	**∠C1–N1–N2 (°)**	**∠N1–N2–N2' (°)**	**WBI N1–N2**	**WBI N2–N2'**
**Experimental**	1.308(5)	1.339(6)	111.6(3)	109.6(4)		
**Calculated**	1.319	1.330	113.28	110.18	1.4297	1.3845

3.2 Charge and Spin Delocalization Data of [3]$^{•–}$

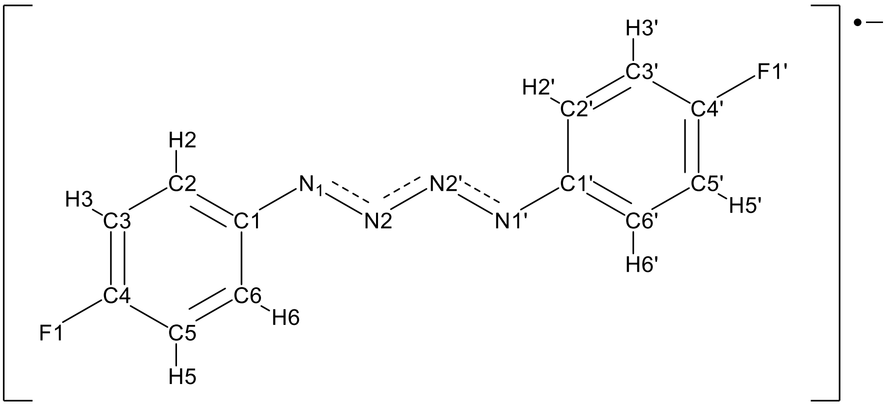

Supplementary Table 13: Calculated charge distributions of [**3**]$^{•–}$ at TPSS/def2-TZVP/SMD(THF) level of theory.

Atom	Charge Distribution		
	**NPA**	**Hirshfeld**	**Mulliken**
F1	–0.330	–0.135	–0.229
N1	–0.315	–0.205	–0.082
N2	–0.167	–0.133	–0.160
C1	0.061	–0.007	0.121
C2	–0.230	–0.062	–0.241
H2	0.228	0.028	0.072
C3	–0.277	–0.063	–0.208
H3	0.230	0.052	0.137
C4	0.334	0.059	0.312
C5	–0.287	–0.066	–0.207
H5	0.232	0.052	0.134
C6	–0.201	–0.058	–0.235
H6	0.220	0.038	0.087
F1’	–0.330	–0.135	–0.229
N1’	–0.315	–0.205	–0.082
N2’	–0.167	–0.133	–0.160
C1’	0.061	–0.007	0.121
C2’	–0.230	–0.062	–0.241
H2’	0.228	0.028	0.072
C3’	–0.277	–0.063	–0.208
H3’	0.230	0.052	0.137

C4’	0.334	0.059	0.312
C5’	–0.287	–0.066	–0.207
H5’	0.232	0.052	0.134
C6’	–0.201	–0.058	–0.235
H6’	0.220	0.038	0.087
Total	–1.000	–1.000	–1.000

Supplementary Table 14: Calculated spin densities of [**3**]$^{•-}$ at TPSS/def2-TZVP/SMD(THF) level of theory.

Atom	Spin Densities		
	**NPA**	**Hirshfeld**	**Mulliken**
F1	0.010	0.015	0.007
N1	0.275	0.234	0.288
N2	0.052	0.066	0.055
C1	–0.045	0.004	–0.036
C2	0.104	0.069	0.100
H2	–0.003	0.005	–0.006
C3	–0.038	–0.010	–0.037
C4	0.099	0.069	0.085
H3	0.001	–0.001	0.001
C5	–0.037	–0.011	–0.036
H5	0.001	–0.001	0.001
C6	0.084	0.059	0.081
H6	–0.003	0.003	–0.004
F1’	0.010	0.015	0.007
N1’	0.275	0.234	0.288
N2’	0.052	0.066	0.055
C1’	–0.045	0.004	–0.036
C2’	0.104	0.069	0.100
H2’	–0.003	0.005	–0.006
C3’	–0.038	–0.010	–0.037
C4’	0.099	0.069	0.085
H3’	0.001	–0.001	0.001
C5’	–0.037	–0.011	–0.036
H5’	0.001	–0.001	0.001
C6’	0.084	0.059	0.081
H6’	–0.003	0.003	–0.004
Total	1.000	1.000	1.000

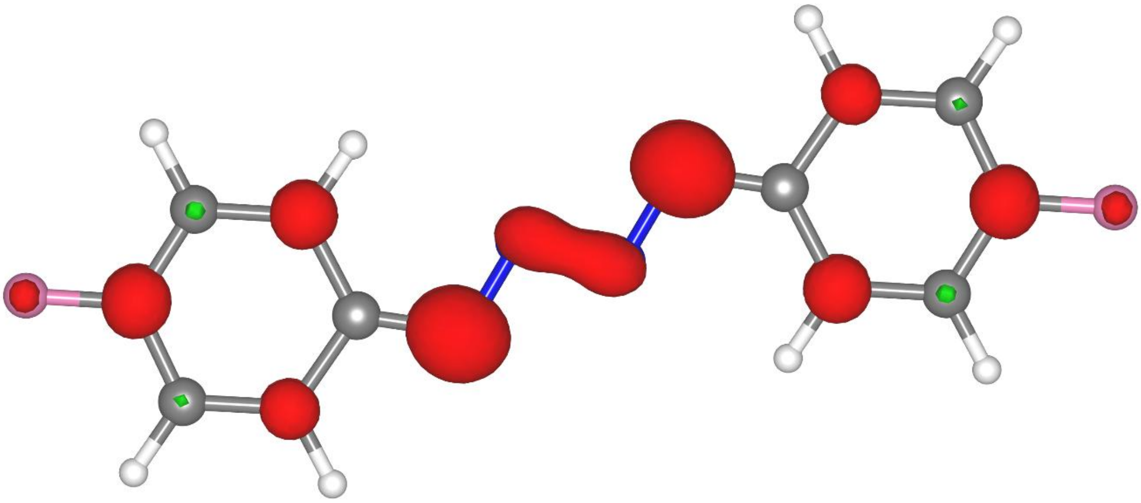

Supplementary Figure 23: Spin density plot (front-on) of [**3**]$^{\bullet-}$ with isovalue = 0.005.

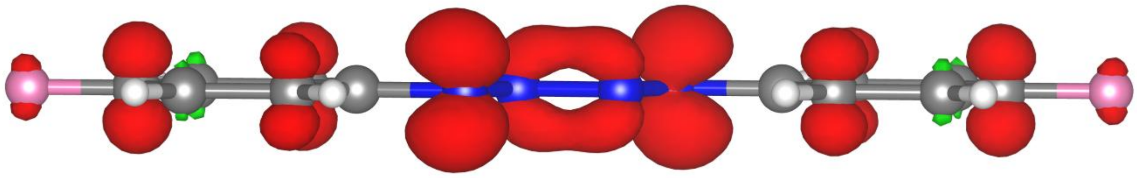

Supplementary Figure 24: Spin density plot (side-on) of [**3**]$^{\bullet-}$ with isovalue = 0.005.

3.3 EPR Data of [K(crypt)][3]

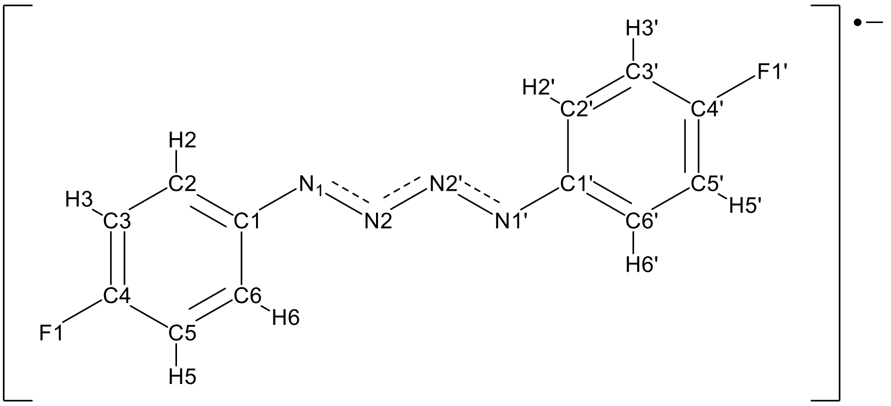

Supplementary Table 15: Calculated A_{iso} values of [**3**]$^{•-}$ at EPR-III (aug-cc-PVTZ for F)/B3LYP/SMD(THF) level of theory.

**Atom**	**A_{iso} (MHz)**	**Atom**	**A_{iso} (MHz)**
N1	13.676618	N1’	13.676676
N2	–0.071868	N2’	–0.071938
H2	–7.240625	H2’	–7.240689
H3	3.255201	H3’	3.255167
H5	3.114033	H5’	3.114019
H6	–8.498838	H6’	–8.499187
F1	11.791171	F1’	11.781406

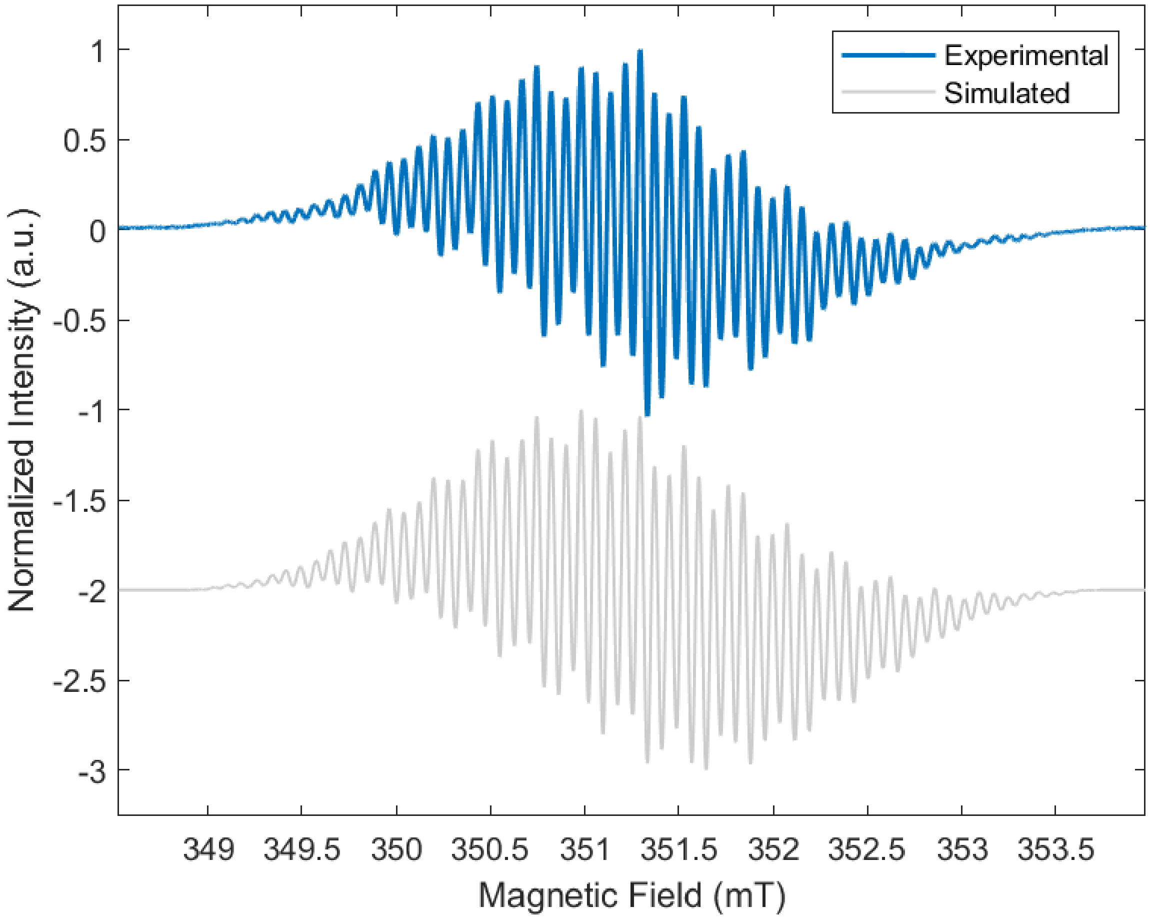

Supplementary Figure 25: Continuous wave EPR spectrum of [K(crypt)][**3**] (blue) stacked above the simulated spectrum (grey, ×2 A_N = 15.33, MHz, ×2 A_N = 1.70 MHz, ×4 A_H = 6.75 MHz, ×4 A_H = 2.27 MHz, ×2 A_F = 13.25 MHz, g = 2.004, lw = 0.06). Experimental parameters: solvent: THF, frequency: 9.854 GHz, temperature: 298 K, modulation amplitude: 0.2 G, scans: 20, gain: 30 dB.

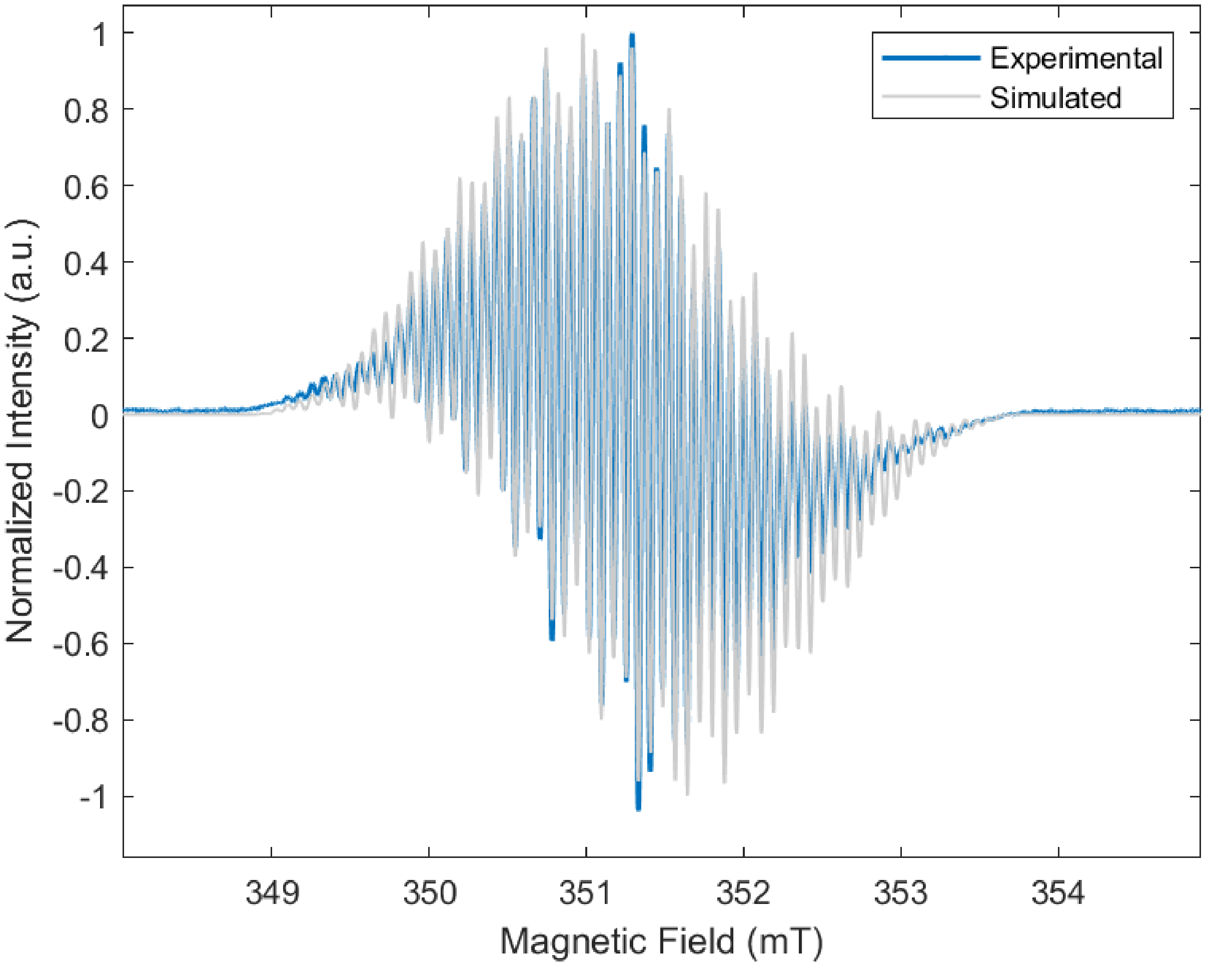

Supplementary Figure 26: Continuous wave EPR spectrum of [K(crypt)][**3**] (blue) overlayed with the simulated spectrum (grey, ×2 A_N = 15.33, MHz, ×2 A_N = 1.70 MHz, ×4 A_H = 6.75 MHz, ×4 A_H = 2.27 MHz, ×2 A_F = 13.25 MHz, g = 2.004, lw = 0.06). Experimental parameters: solvent: THF, frequency: 9.854 GHz, temperature: 298 K, modulation amplitude: 0.2 G, scans: 20, gain: 30 dB.

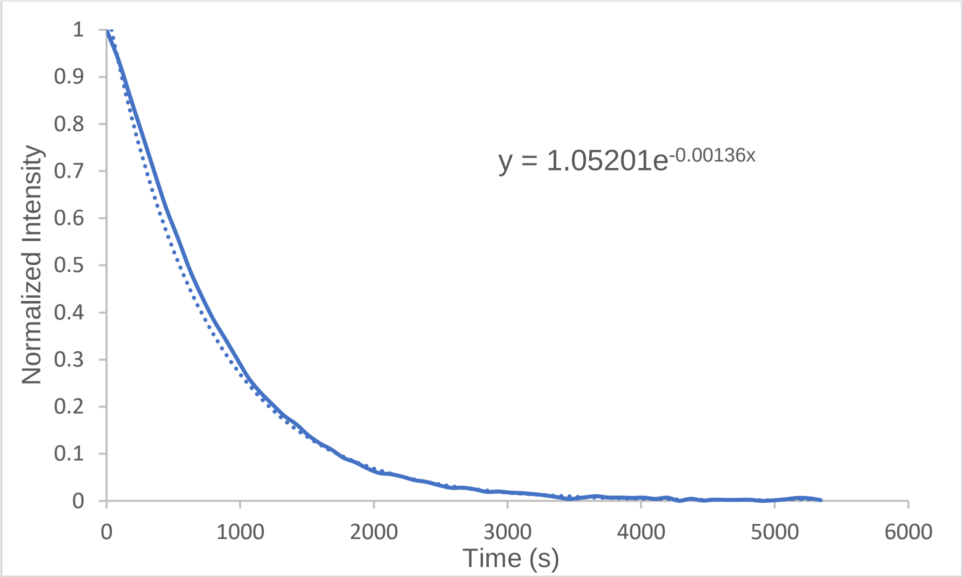

Supplementary Figure 27: Decay curve of the EPR signal intensity for [K(crypt)][**3**] in THF with the exponential equation shown.

Supplementary Table 16: Decay curve equation and corresponding half-life of [K(crypt)][**3**]

Compound	Equation	Half-life (s)
[K(crypt)][**3**]	$y = 1.05201e^{-0.00136x}$	546.9 (9.12 mins)

Supplementary Table 17: Spin counting for compound [K(crypt)][**3**]

Compound	Actual Concentration (mM)	Number of Spins ($x10^{15}$)	Measured Concentration (mM)
[K(crypt)][**3**]	0.43	20.95	0.41

3.4 Cyclic Voltammetry Data of [K(crypt)][3]

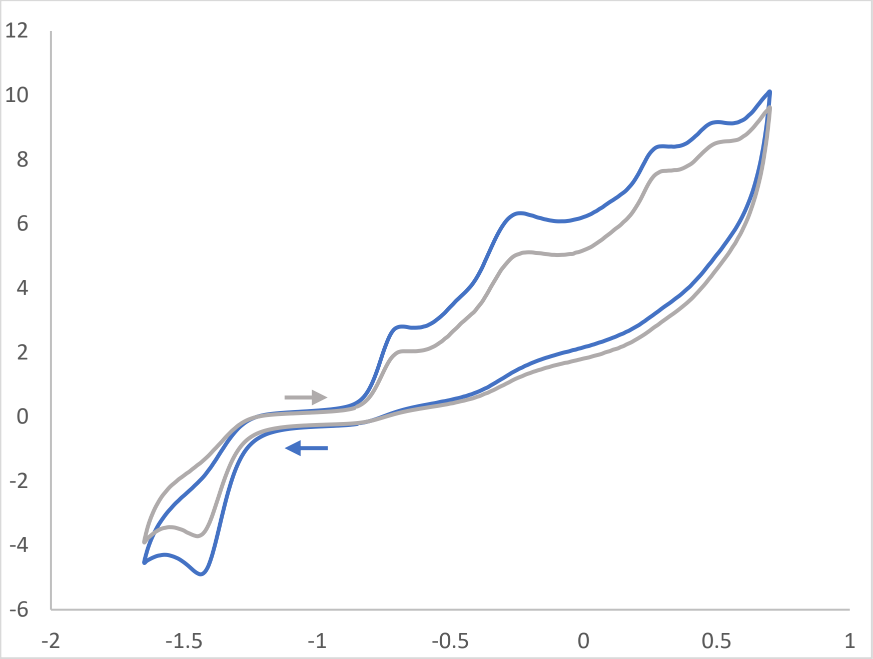

Supplementary Figure 28: Cyclic Voltammetry of [K(crypt)][**3**] 3 mM in THF with [nBu_4N][PF_6] electrolyte at 0.1 V/s starting at –0.8 V and scanning independently in the positive direction first (grey trace) and the negative direction first (blue trace). Glassy carbon working electrode, platinum wire counter electrode, and leak-proof Ag/AgCl reference electrode were used.

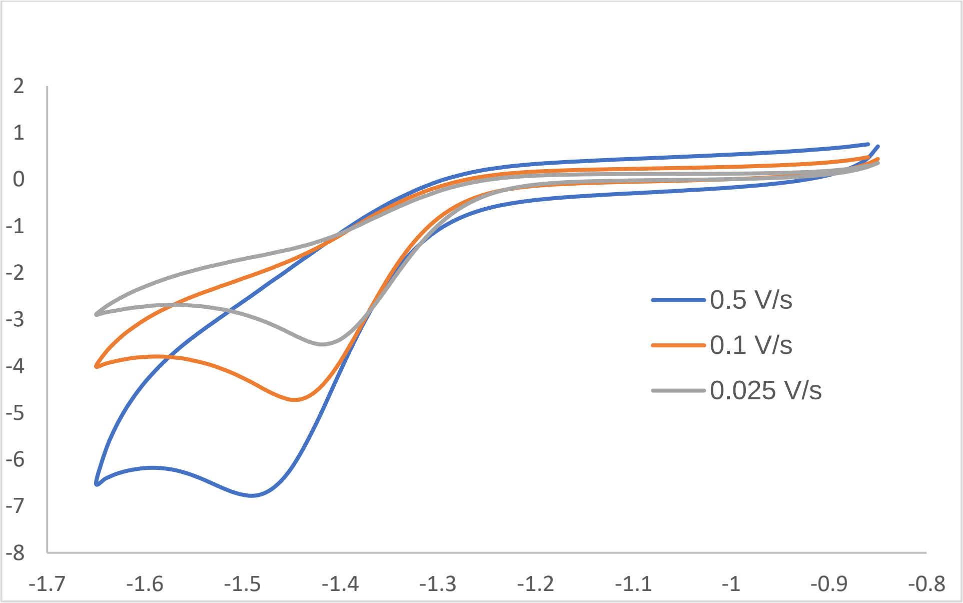

Supplementary Figure 29: Cyclic Voltammetry of [K(crypt)][**3**] 0.3 M in THF with [nBu_4N][PF_6] electrolyte at varying scan rates on first reduction wave using glassy carbon working electrode, platinum wire counter electrode, and leak-proof Ag/AgCl reference electrode.

3.5 UV-Vis Spectrum and NTOs of [K(crypt)][3]

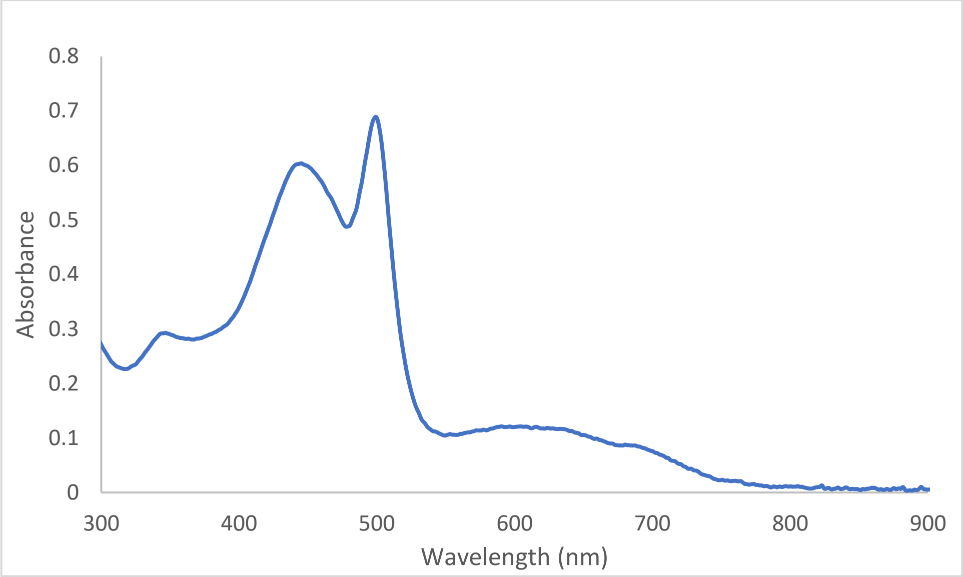

Supplementary Figure 30: UV-Vis spectrum of [K(crypt)][**3**] at an initial concentration of 0.05 mM in *o*DFB.

Supplementary Table 18: TD-DFT calculated UV wavelengths, oscillator strengths, natural transition orbitals (NTOs), and their occupations with blue and grey dots for selected excited states of [**3**]$^{\bullet-}$ (calculated at TPSSh/def2-TZVP/SMD(cyclopentanone) level of theory).

Excited State	Wavelength	Oscillator Strength	NTO Occupation Number		
1	329.45	0.0610	0.498	1.072	
2	441.68	0.6490	1.685	0.271	
3	489.27	0.7105	1.142	0.814	
4	601.85	0.0049	1.052	0.932	

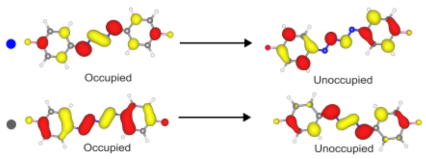

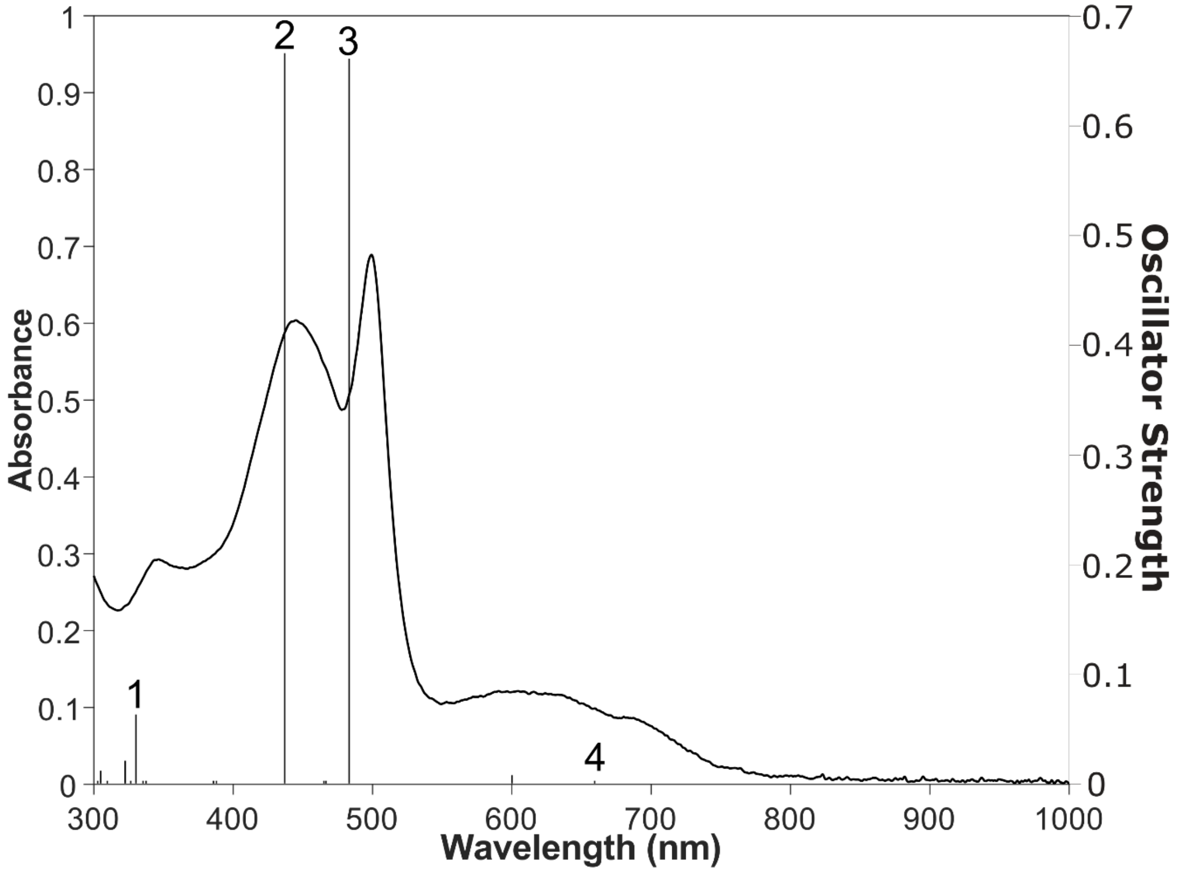

Supplementary Figure 31: Overlayed experimental UV-Vis spectrum of [K(crypt)][**3**] and calculated oscillator strengths of [**3**]$^{\bullet-}$ at TPSSh/def2-TZVP/SMD(cyclopentanone) level of theory.

4. Synthesis and Characterization of [K(crypt)][4]

4.1 Synthesis of [K(crypt)][4]

In the glovebox, KC_8 (100 mg, 0.74 mmol, 1 equiv.) and 2.2.2-cryptand (280 mg, 0.74 mmol, 1 equiv.) were suspended in THF in a J-young ampoule. 1-azido-4-chlorobenzene (227 mg, 1.48 mmol, 2 equiv.) was added to the reaction mixture, and the reaction mixture was stirred for 10 minutes. Then, the solution was filtered and diethyl ether added to precipitate a black solid. The solid was filtered and washed with additional diethyl ether (3 x10 mL) before drying under vacuum yielding [K(crypt)][**4**] as a black crystalline solid. Single crystals were obtained by slow vapor diffusion of hexane into a solution of [K(crypt)][**4**] in THF at –40 °C.

Isolated Yield: 321 mg, 62%

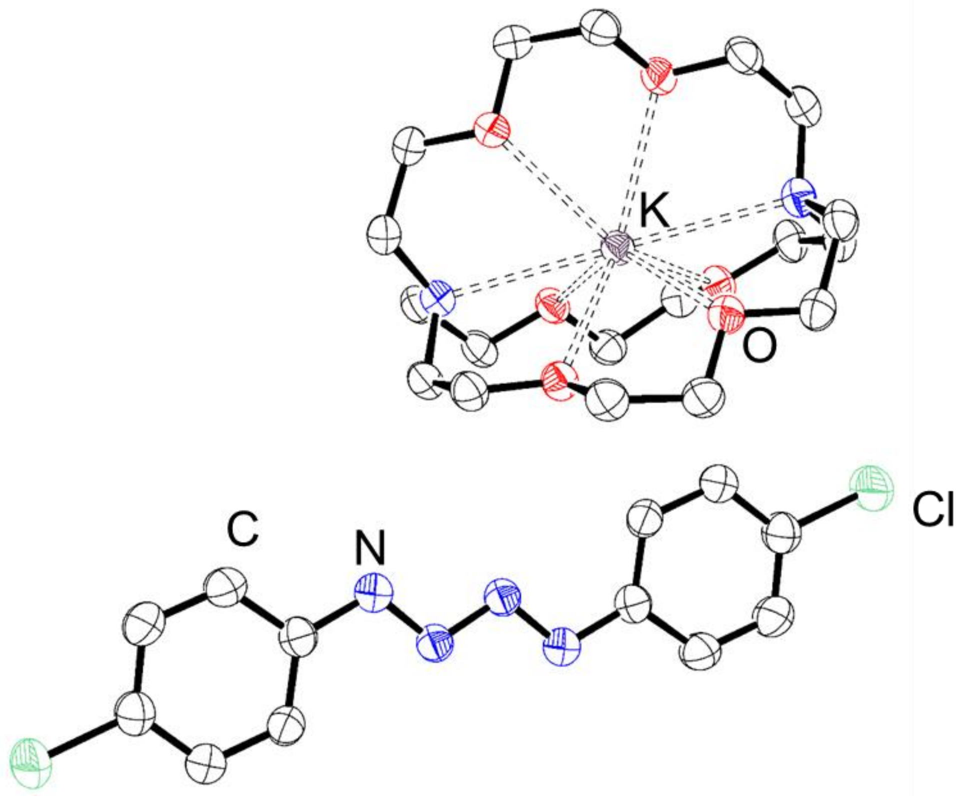

Supplementary Figure 32: Molecular structure of [K(crypt)][**4**] showing anisotropic displacement ellipsoids at 50% probability. Hydrogen atoms omitted for clarity. Nitrogen: blue; carbon: white; chlorine: green; potassium: violet; oxygen: red.

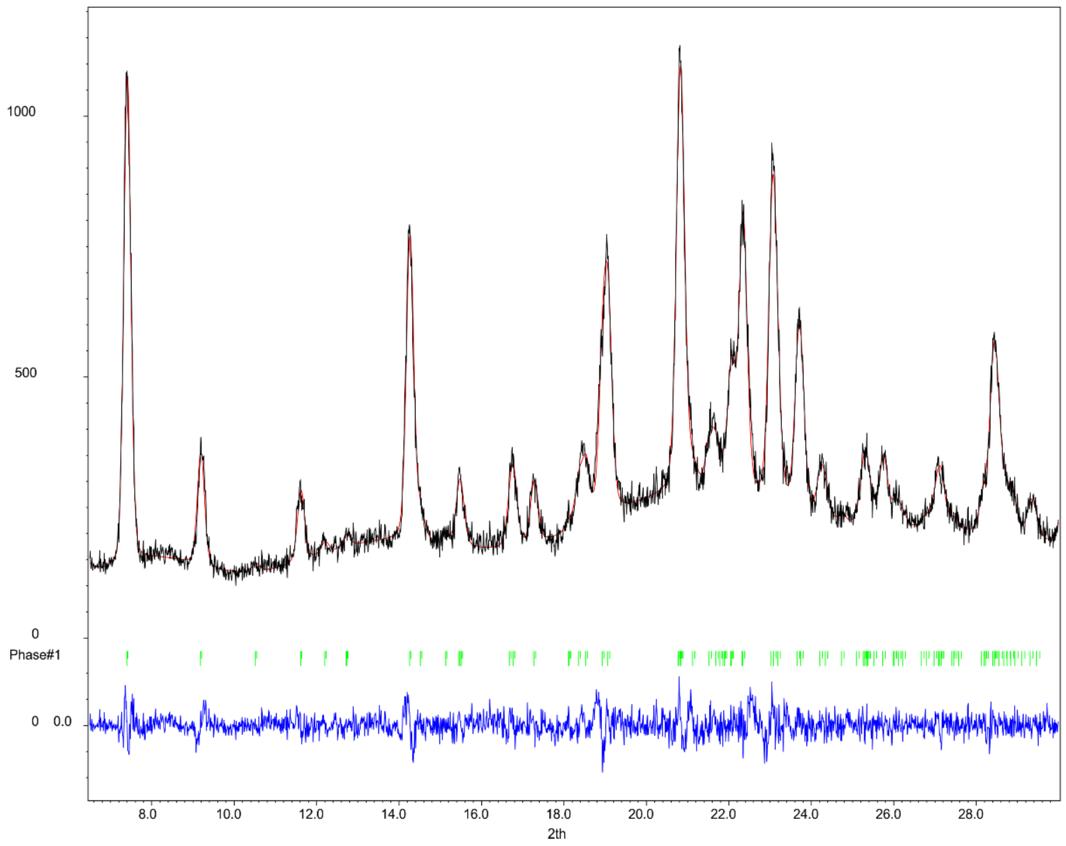

Supplementary Figure 33: Experimental (black) and simulated (red) powder X-ray diffraction (PXRD) patterns of [K(crypt)][**4**].

Supplementary Table 19: Selected experimental and calculated bond lengths, bond angles, and Wiberg bond indices of [**4**]$^{\bullet-}$ calculated at TPSS/def2-TZVP/SMD(THF) level of theory.

	**N1–N2 bond length (Å)**	**N2–N2' bond length (Å)**	**∠C1–N1–N2 (°)**	**∠N1–N2–N2' (°)**	**WBI N1–N2**	**WBI N2–N2'**
**Experimental**	1.314(2)	1.334(3)	112.45(17)	109.6(2)		
**Calculated**	1.318	1.330	113.35	110.03	1.4279	1.3859

4.2 Charge and Spin Delocalization data $[4]^{\bullet-}$

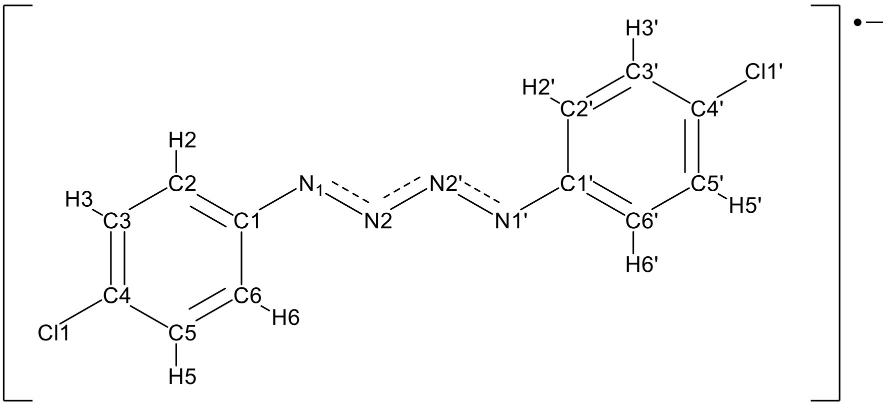

Supplementary Table 20: Calculated charge distributions of [**4**]$^{\bullet-}$ at TPSS/def2-TZVP/SMD(THF) level of theory.

Atom	Charge Distribution		
	NPA	Hirshfeld	Mulliken
Cl1	–0.039	–0.104	–0.187
N1	–0.308	–0.198	–0.078
N2	–0.159	–0.124	–0.157
C1	0.072	–0.001	0.130
C2	–0.203	–0.057	–0.239
H2	0.221	0.038	0.087
C3	–0.242	–0.058	–0.180
H3	0.229	0.047	0.133
C4	–0.062	–0.002	0.200
C5	–0.233	–0.055	–0.192
H5	0.227	0.047	0.136
C6	–0.232	–0.061	–0.220
H6	0.230	0.028	0.069
Cl1’	–0.039	–0.104	–0.187
N1’	–0.308	–0.198	–0.078
N2’	–0.159	–0.124	–0.157
C1’	0.072	–0.001	0.130
C2’	–0.203	–0.057	–0.239
H2’	0.221	0.038	0.087
C3’	–0.242	–0.058	–0.180
H3’	0.229	0.047	0.133

C4’	–0.062	–0.002	0.200
C5’	–0.233	–0.055	–0.192
H5’	0.227	0.047	0.136
C6’	–0.232	–0.061	–0.220
H6’	0.230	0.028	0.069
Total	–1.000	–1.000	–1.000

Supplementary Table 21: Calculated spin densities of [**4**]$^{\bullet-}$ at TPSS/def2-TZVP/SMD(THF) level of theory.

Atom	Spin Densities		
	**NPA**	**Hirshfeld**	**Mulliken**
Cl1	0.012	0.020	0.011
N1	0.264	0.225	0.268
N2	0.054	0.066	0.052
C1	–0.043	0.004	–0.037
C2	0.084	0.059	0.083
H2	–0.003	0.003	–0.004
C3	–0.036	–0.010	–0.038
H3	0.001	–0.001	0.002
C4	0.106	0.074	0.103
C5	–0.039	–0.011	–0.043
H5	0.001	–0.001	0.002
C6	0.102	0.067	0.109
H6	–0.003	0.005	–0.007
Cl1’	0.012	0.020	0.011
N1’	0.264	0.225	0.268
N2’	0.054	0.066	0.052
C1’	–0.043	0.004	–0.037
C2’	0.084	0.059	0.083
H2’	–0.003	0.003	–0.004
C3’	–0.036	–0.010	–0.038
H3’	0.001	–0.001	0.002
C4’	0.106	0.074	0.103
C5’	–0.039	–0.011	–0.043
H5’	0.001	–0.001	0.002
C6’	0.102	0.067	0.109
H6’	–0.003	0.005	–0.007
Total	1.000	1.000	1.000

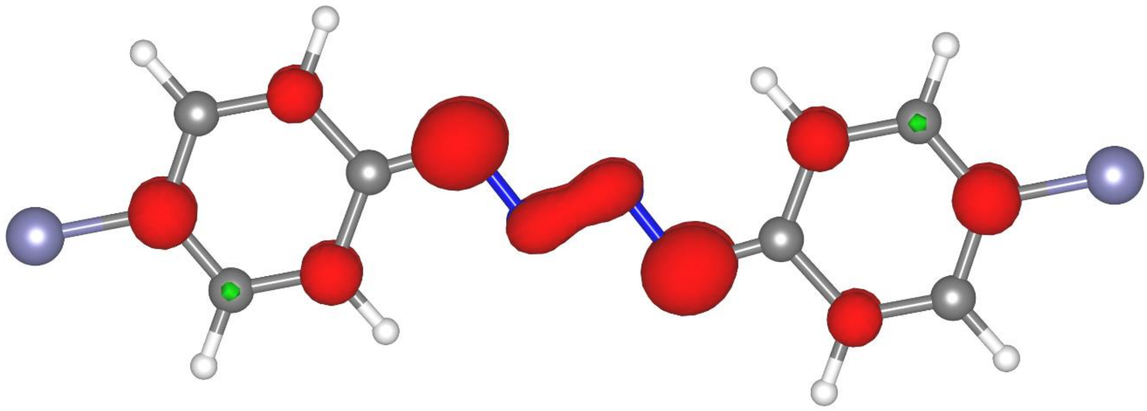

Supplementary Figure 34: Spin density plot (front-on) of [**4**]$^{\bullet-}$ with isovalue = 0.005.

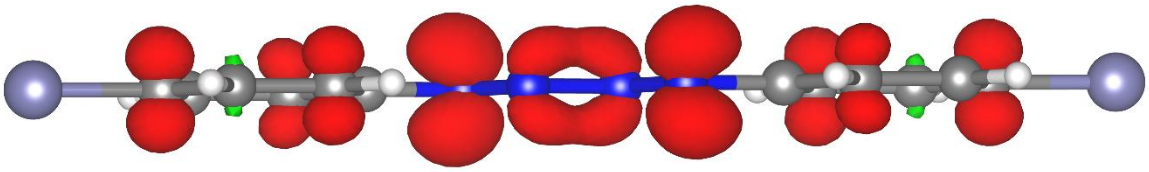

Supplementary Figure 35: Spin density plot (side-on) of [**4**]$^{\bullet-}$ with isovalue = 0.005.

4.3 EPR data of [K(crypt)][4]

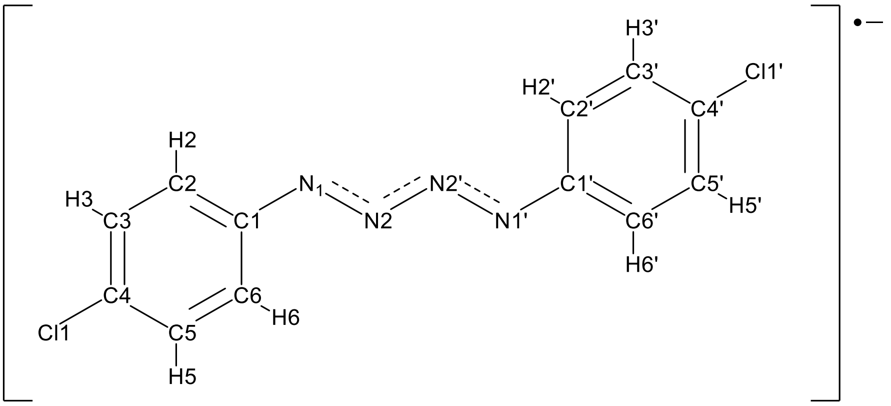

Supplementary Table 22: Calculated A_{iso} values for $[\mathbf{4}]^{\bullet-}$ at EPR-III(aug-cc-PVTZ for Cl)/B3LYP/SMD(THF) level of theory.

**Atom**	**A_{iso} (MHz)**	**Atom**	**A_{iso} (MHz)**
N1	13.109549	N1’	13.109553
N2	0.089931	N2’	0.089931
H2	–7.118952	H2’	–7.118952
H3	3.257564	H3’	3.257587
H5	3.327698	H5’	3.327720
H6	–8.263466	H6’	–8.263481
Cl1	1.062301	Cl1’	1.062252

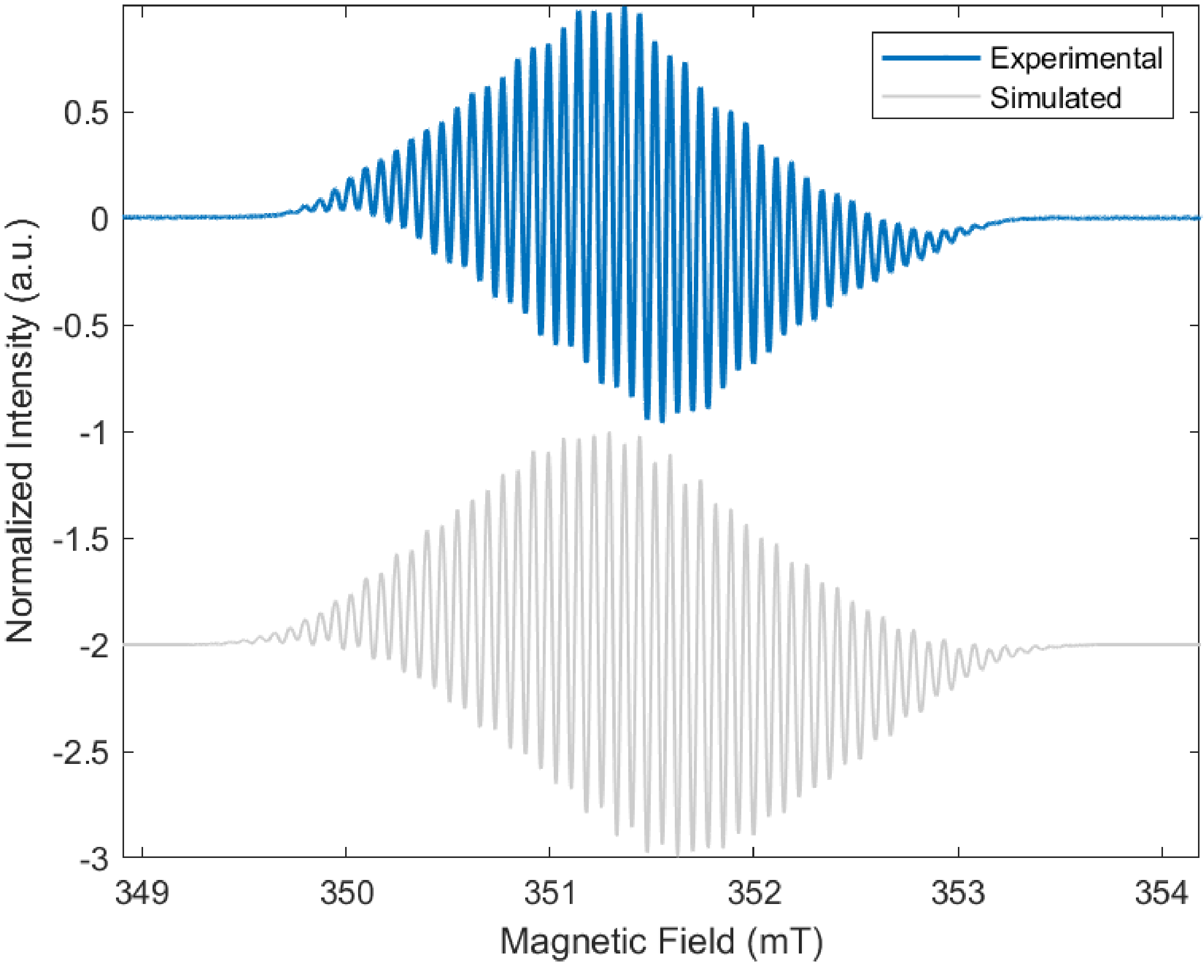

Supplementary Figure 36: Continuous wave EPR spectrum of [K(crypt)][**4**] (blue) stacked above the simulated spectrum (grey, ×2 A_N = 14.64, MHz, ×2 A_N = 2.09 MHz, ×4 A_H = 8.38 MHz, ×4 A_H = 4.13 MHz, ×2 A_{Cl} = 0.83 MHz, g = 2.004, lw = 0.04). Experimental parameters: solvent: THF, frequency: 9.859 GHz, temperature: 298 K, modulation amplitude: 0.2 G, scans: 20, gain: 30 dB.

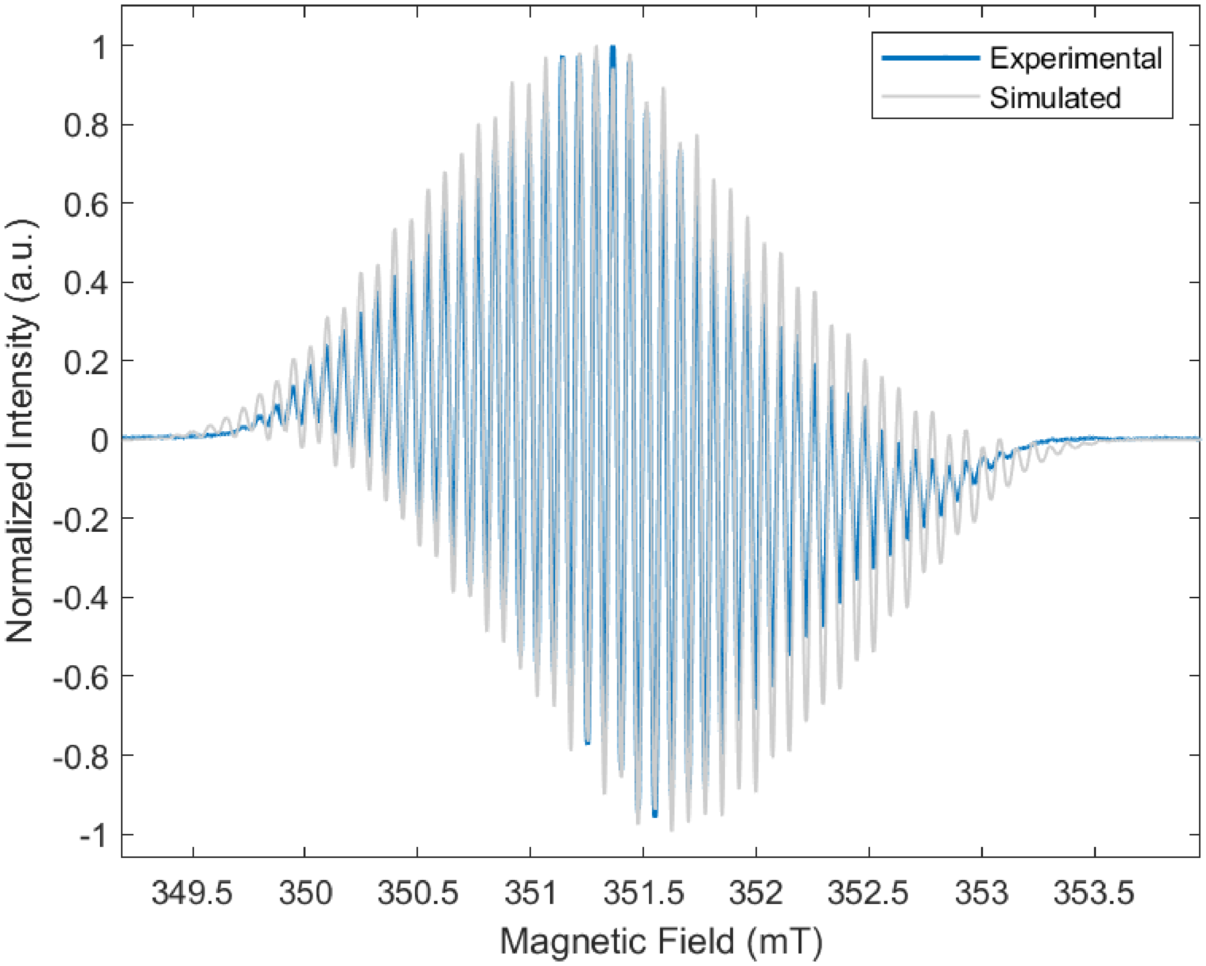

Supplementary Figure 37: Continuous wave EPR spectrum of [K(crypt)][**4**] (blue) overlayed with the simulated spectrum (grey, ×2 A_N = 14.64, MHz, ×2 A_N = 2.09 MHz, ×4 A_H = 8.38 MHz, ×4 A_H = 4.13 MHz, ×2 A_{Cl} = 0.83 MHz, g = 2.004, lw = 0.04). Experimental parameters: solvent: THF, frequency: 9.859 GHz, temperature: 298 K, modulation amplitude: 0.2 G, scans: 20, gain: 30 dB.

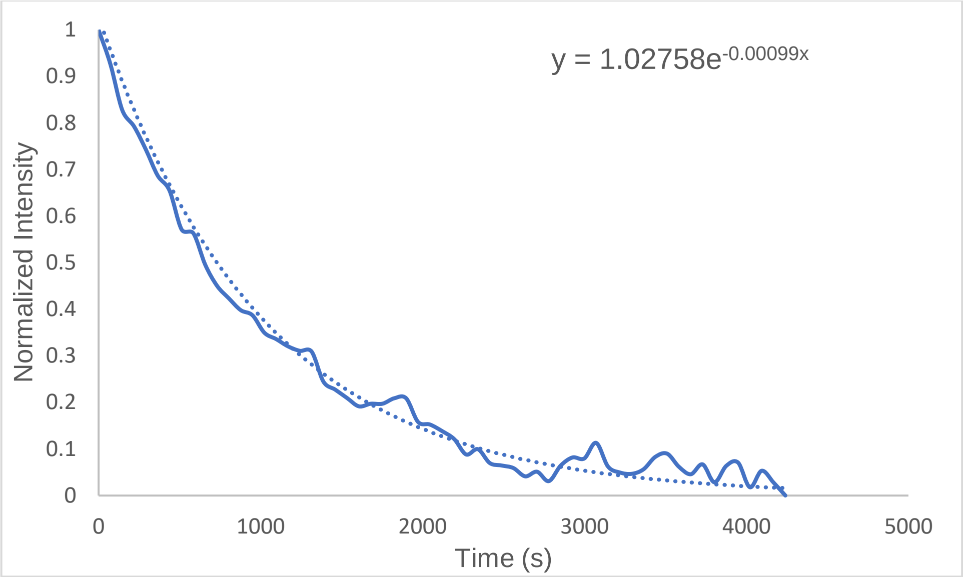

Supplementary Figure 38: Decay curve of the EPR signal intensity for [K(crypt)][**4**] in THF with the exponential equation shown.

Supplementary Table 23: Decay curve equation and corresponding half-life of [K(crypt)][**4**]

Compound	Equation	Half-life (s)
[K(crypt)][**4**]	$y = 1.02758e^{-0.00099x}$	727.6 (12.13 mins)

Supplementary Table 24: Spin counting for compound [K(crypt)][**4**]

Compound	Actual Concentration (mM)	Number of Spins ($x10^{15}$)	Measured Concentration (mM)
[K(crypt)][**4**]	0.85	43.51	0.82

4.4 Cyclic Voltammetry Data of [K(crypt)][4]

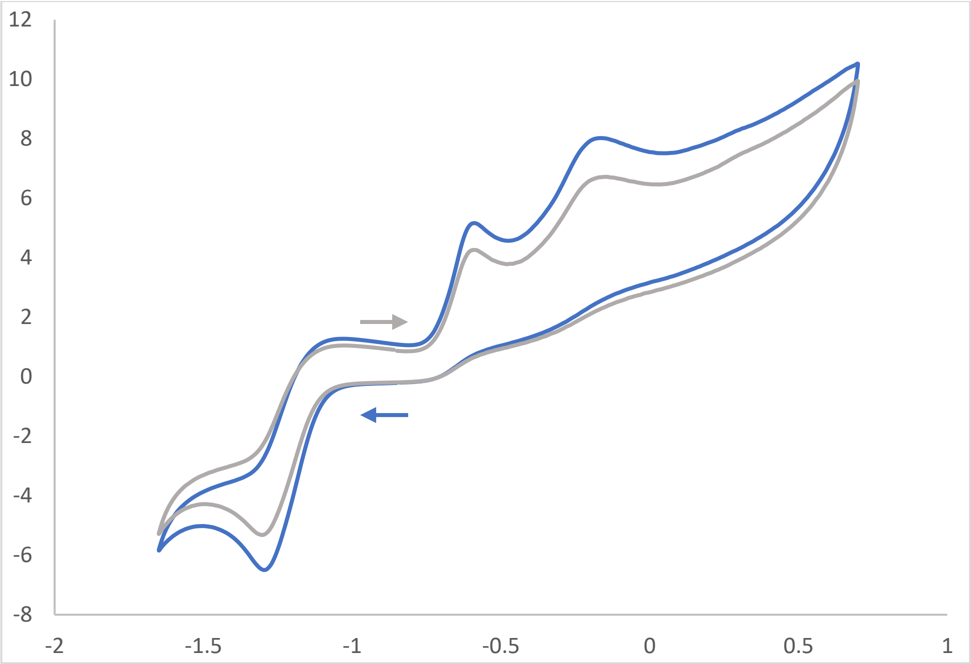

Supplementary Figure 39: Cyclic Voltammetry of [K(crypt)][**4**] 3 mM in THF with [nBu_4N][PF_6] electrolyte at 0.1 V/s starting at –0.8 V and scanning independently in the positive direction first (grey trace) and the negative direction first (blue trace). Glassy carbon working electrode, platinum wire counter electrode, and leak-proof Ag/AgCl reference electrode were used.

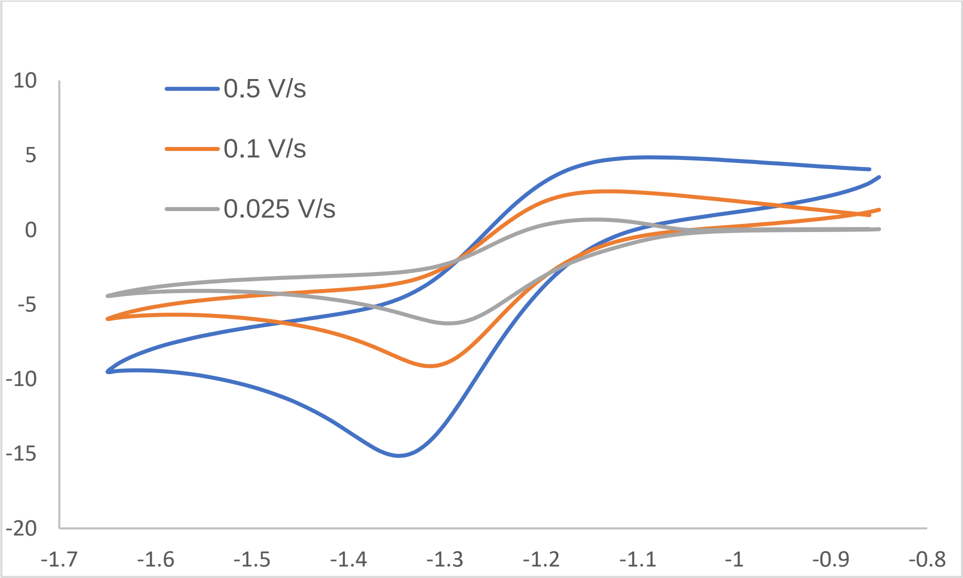

Supplementary Figure 40: Cyclic Voltammetry of [K(crypt)][**4**] 0.3 M in THF with [nBu_4N][PF_6] electrolyte at varying scan rates on first reduction wave using glassy carbon working electrode, platinum wire counter electrode, and leak-proof Ag/AgCl reference electrode.

4.5 UV-Vis spectrum and NTOs of [K(crypt)][4]

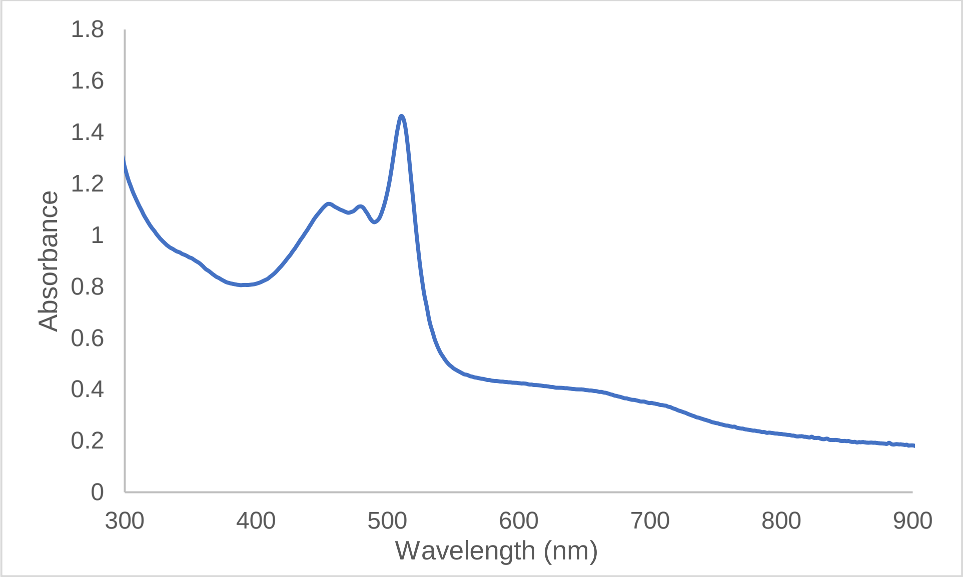

Supplementary Figure 41: UV-Vis spectrum of [K(crypt)][**4**] at an initial concentration of 0.05 mM in *o*DFB.

Supplementary Table 25: TD-DFT calculated UV wavelengths, oscillator strengths, natural transition orbitals (NTOs), and their occupations with blue and grey dots for selected excited states of [**4**]$^{\bullet-}$ (calculated at TPSSh/def2-TZVP/SMD(cyclopentanone) level of theory).

**Excited State**	**Wavelength**	**Oscillator Strength**	**NTO Occupation Number**		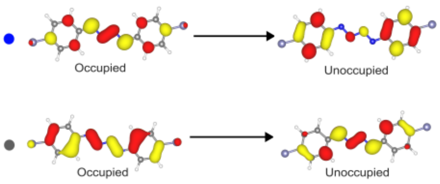
1	338.69	0.0504	0.246	1.516	
2	438.23	0.3817	1.811	0.140	
3	487.29	1.1622	1.127	0.832	
4	612.37	0.0124	0.933	1.041	

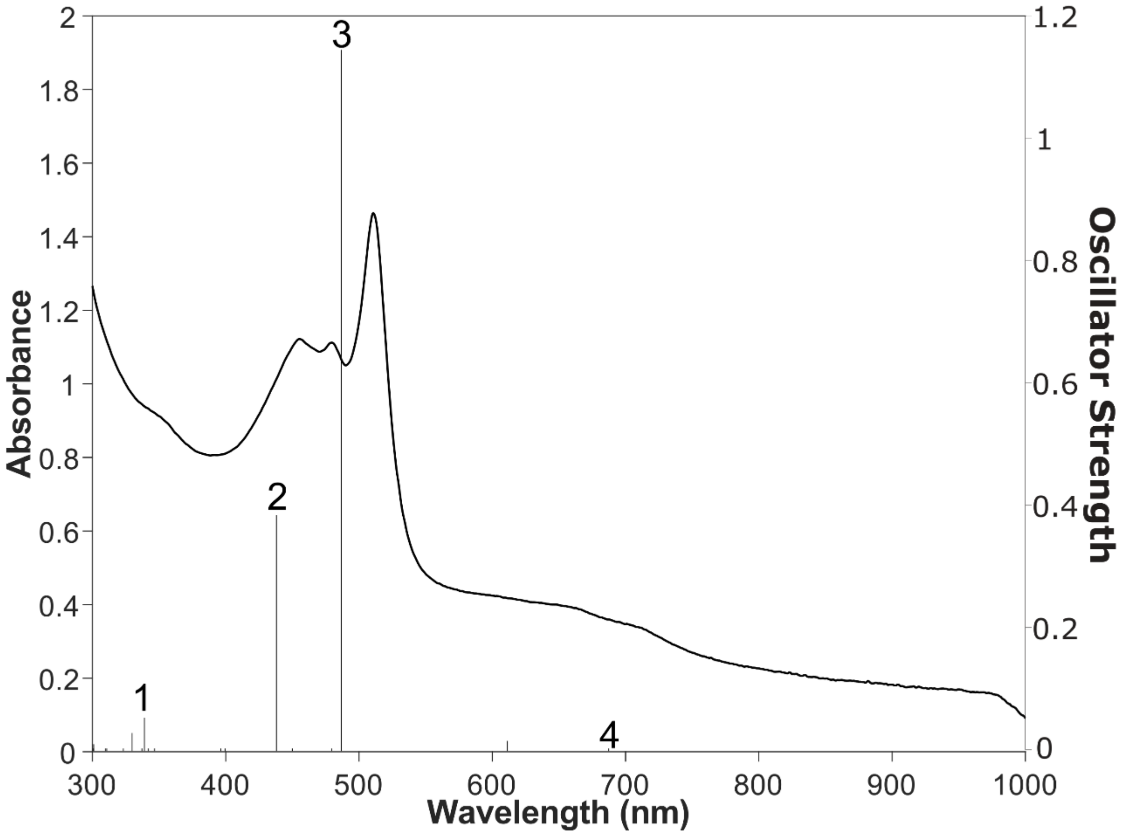

Supplementary Figure 42: Overlayed experimental UV-Vis spectrum of [K(crypt)][**4**] and calculated oscillator strengths of [**4**]$^{\bullet-}$ at TPSSh/def2-TZVP/SMD(cyclopentanone) level of theory.

5. Synthesis and Characterization of [K(crypt)][5]

5.1 Synthesis of [K(crypt)][5]

In the glovebox, KC_8 (100 mg, 0.74 mmol, 1 equiv.) and 2.2.2-cryptand (280 mg, 0.74 mmol, 1 equiv.) were suspended in THF in a J-young ampoule. 1-azido-4-methylbenzene (197 mg, 1.48 mmol, 2 equiv.) was added to the reaction mixture, and the reaction mixture was stirred for 10 minutes. Then, the solution was filtered and diethyl ether added to precipitate a black solid. The solid was filtered and washed with additional diethyl ether (3 x10 mL) before drying under vacuum yielding [K(crypt)][**5**] as a black crystalline solid. Single crystals were obtained by slow vapor diffusion of hexane into a solution of [K(crypt)][**5**] in THF at –40 °C.

Isolated Yield: 202 mg, 42%

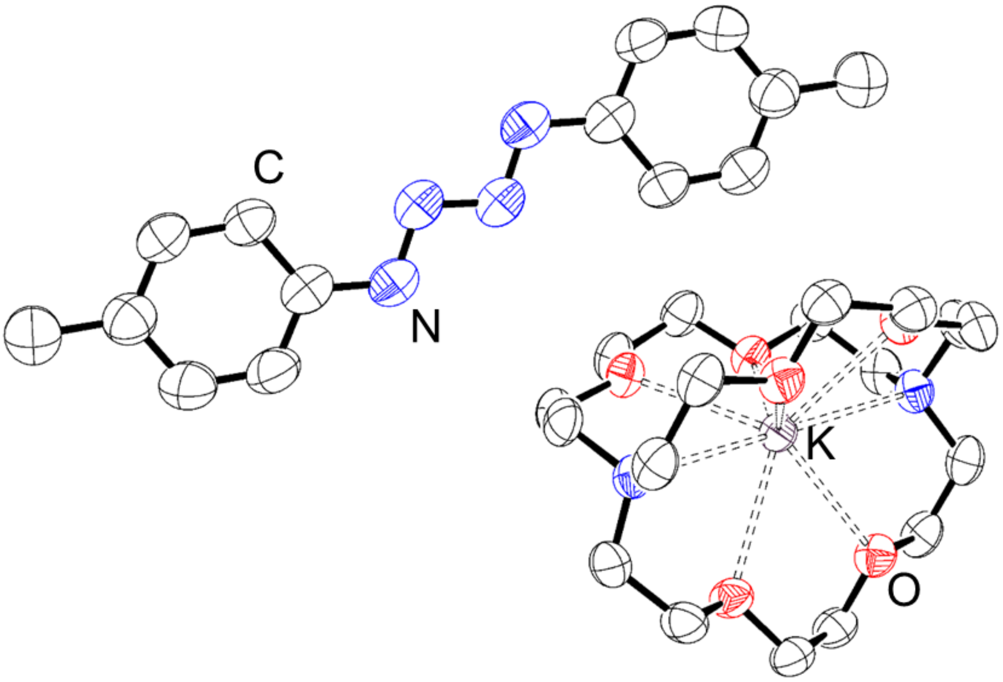

Supplementary Figure 43: Molecular structure of [K(crypt)][**5**] showing anisotropic displacement ellipsoids at 50% probability. Hydrogen atoms omitted for clarity. Nitrogen: blue; carbon: white; potassium: violet; oxygen: red.

Supplementary Table 26: Selected experimental and calculated bond lengths, bond angles and Wiberg bond indices of [**5**]$^{\bullet-}$ calculated at TPSS/def2-TZVP/SMD(THF) level of theory.

	N1–N2 bond length (Å)	N2–N2' bond length (Å)	∠C1–N1–N2 (°)	∠N1–N2–N2' (°)	WBI N1-N2	WBI N2-N2'
**Experimental**	1.311(3)	1.316(4)	112.80(18)	110.4(2)		
**Calculated**	1.319	1.330	113.49	110.13	1.4286	1.3844

5.2 Charge and Spin Delocalization of [5] $^{•–}$

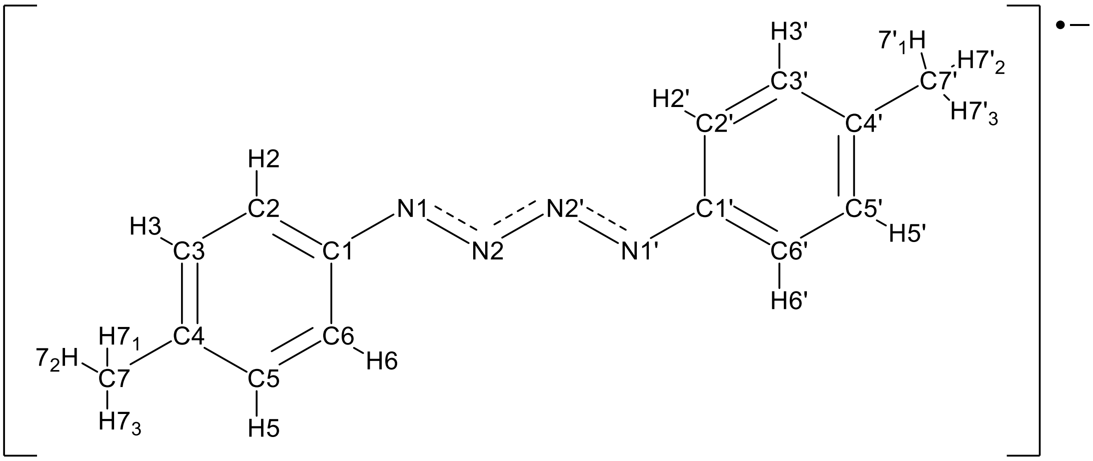

Supplementary Table 27: Calculated charge distributions of [**5**]$^{•–}$ at TPSS/def2-TZVP/SMD(THF) level of theory.

Atom	Charge Distribution		
	NPA	Hirshfeld	Mulliken
N1	–0.319	–0.209	–0.077
N2	–0.168	–0.136	–0.158
C1	0.064	–0.010	0.116
C2	–0.214	–0.070	–0.243
H2	0.213	0.030	0.072
C3	–0.223	–0.063	–0.227
H3	0.207	0.037	0.118
C4	–0.049	–0.018	0.139
C5	–0.215	–0.061	–0.223
H5	0.206	0.036	0.121
C6	–0.247	–0.075	–0.230
H6	0.221	0.021	0.055
C7	–0.606	–0.082	–0.364
H7	0.211	0.033	0.134
H8	0.211	0.034	0.133
H9	0.208	0.034	0.133
N1’	–0.319	–0.209	–0.077
N2’	–0.168	–0.136	–0.158
C1’	0.064	–0.010	0.116
C2’	–0.214	–0.070	–0.243

H2’	0.213	0.030	0.072
C3’	–0.223	–0.063	–0.227
H3’	0.207	0.037	0.118
C4’	–0.049	–0.018	0.139
C5’	–0.215	–0.061	–0.223
H5’	0.206	0.036	0.121
C6’	–0.247	–0.075	–0.230
H6’	0.221	0.021	0.055
C7’	–0.606	–0.082	–0.364
H7’	0.211	0.033	0.134
H8’	0.211	0.034	0.133
H9’	0.208	0.034	0.133
Total	–1.000	–1.000	–1.000

Supplementary Table 28: Calculated spin densities of [**5**]$^{•−}$ at TPSS/def2-TZVP/SMD(THF) level of theory.

Atom	Spin Distribution		
	NPA	Hirshfeld	Mulliken
N1	0.272	0.231	0.277
N2	0.052	0.065	0.046
C1	–0.045	0.003	–0.040
C2	0.081	0.056	0.083
H2	–0.003	0.003	–0.004
C3	–0.037	–0.011	–0.041
H3	0.001	–0.001	0.002
C4	0.109	0.075	0.114
C5	–0.038	–0.010	–0.043
H5	0.001	–0.001	0.002
C6	0.105	0.069	0.113
H6	–0.003	0.005	–0.007
C7	–0.006	0.005	–0.009
H7	0.007	0.005	0.004
H8	0.005	0.004	0.003
H9	0.000	0.000	0.000
N1’	0.272	0.231	0.277
N2’	0.052	0.065	0.046
C1’	–0.045	0.003	–0.040
C2’	0.081	0.056	0.083
H2’	–0.003	0.003	–0.004
C3’	–0.037	–0.011	–0.041
H3’	0.001	–0.001	0.002
C4’	0.109	0.075	0.114
C5’	–0.038	–0.010	–0.043
H5’	0.001	–0.001	0.002
C6’	0.105	0.069	0.113
H6’	–0.003	0.005	–0.007
C7’	–0.006	0.005	–0.009
H7’	0.007	0.005	0.004
H8’	0.005	0.004	0.003

H9’	0.000	0.000	0.000
Total	1.000	1.000	1.000

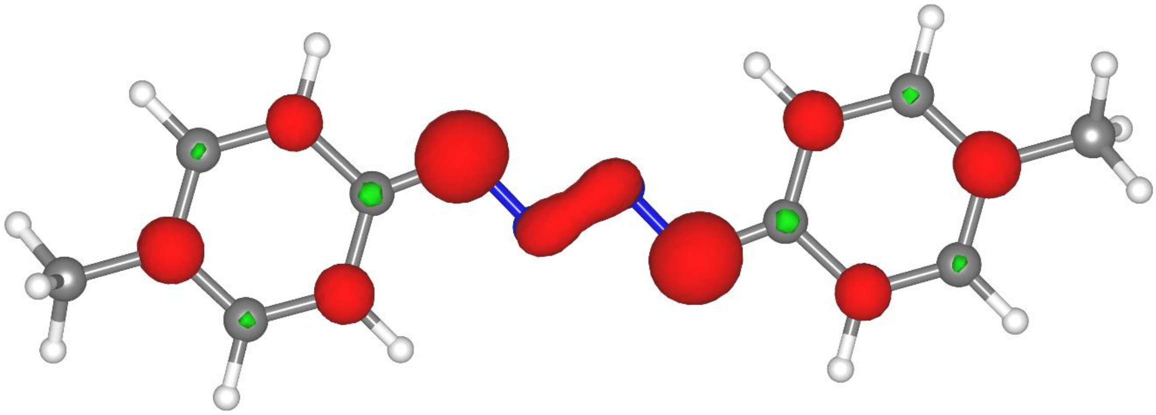

Supplementary Figure 44: Spin density plot (front-on) of [**5**]$^{•–}$ with isovalue = 0.005.

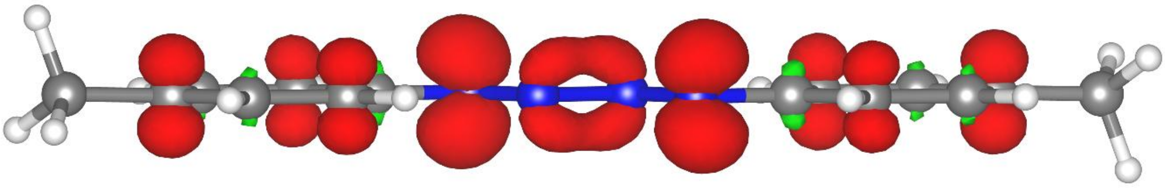

Supplementary Figure 45: Spin density plot (side-on) of [**5**]$^{•–}$ with isovalue = 0.005.

5.3 EPR data of [K(crypt)][5]

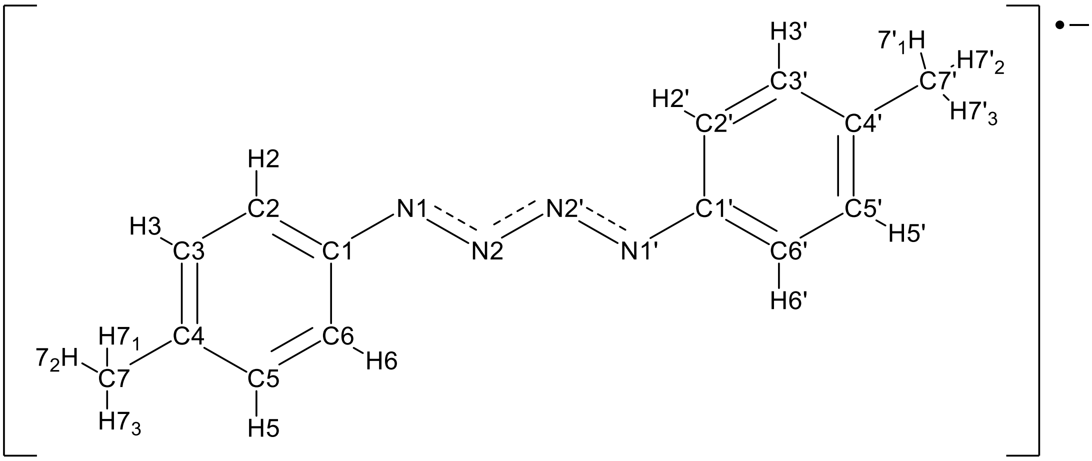

Supplementary Table 29: Calculated A_{iso} values of $[\mathbf{5}]^{\bullet-}$ at EPR-III /B3LYP/SMD(THF) level of theory.

**Atom**	**A_{iso} (MHz)**	**Atom**	**A_{iso} (MHz)**
N1	13.567867	N1'	13.567846
N2	–0.091616	N2'	0.091593
H2	–6.848467	H2'	–6.848441
H3	3.221258	H3'	3.221279
H5	3.075408	H5'	3.075450
H6	–8.386685	H6'	–8.386726
$H7_1$	12.180462	$H7'_1$	15.945540
$H7_2$	15.945599	$H7'_2$	12.180506
$H7_3$	0.668246	$H7'_3$	0.668258

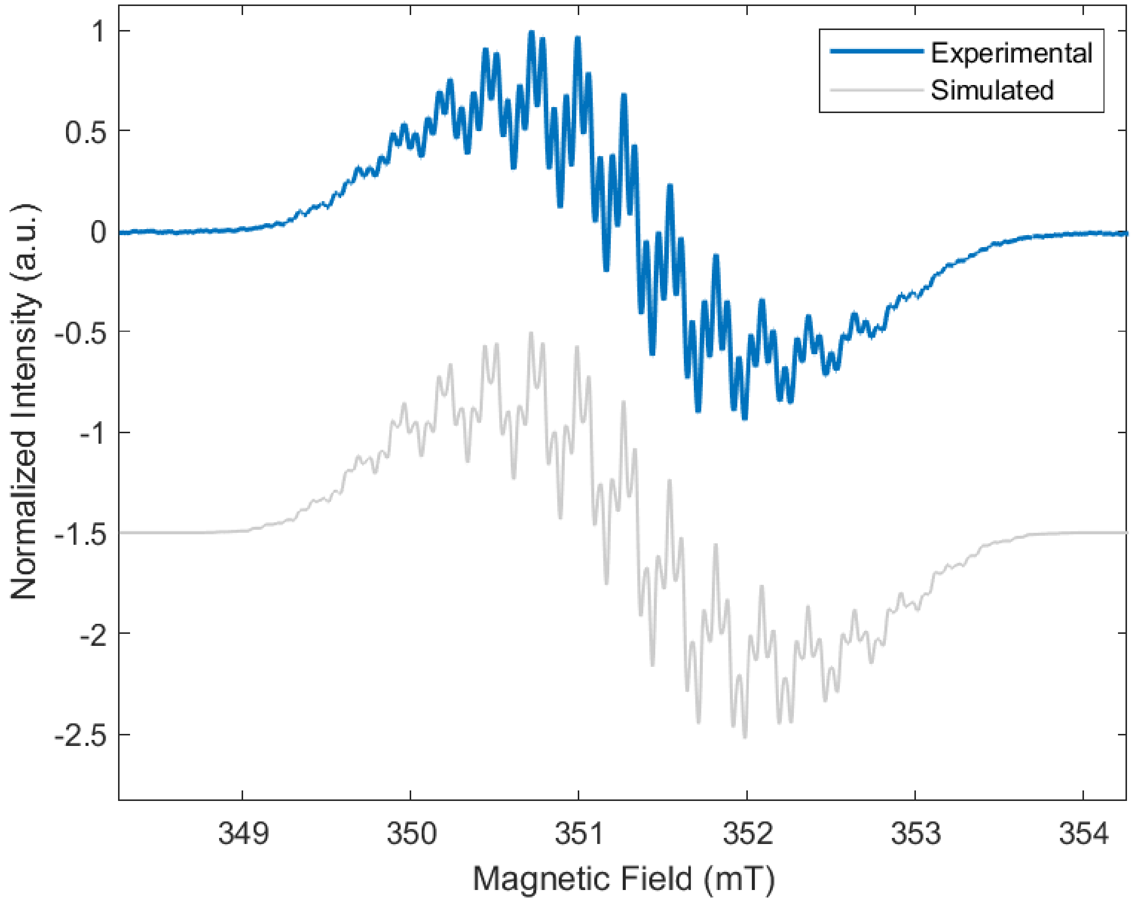

Supplementary Figure 46: Continuous wave EPR spectrum of [K(crypt)][**5**] (blue) stacked above the simulated spectrum (grey, ×2 A_N = 15.38 MHz, ×2 A_N = 0.87 MHz, ×4 A_H = 5.32 MHz, ×4 A_H = 2.29 MHz, ×6 A_H = 7.92 MHz, g = 2.003, lw = 0.05). Experimental parameters: solvent: *o*DFB/Tol, frequency: 9.852 GHz, temperature: 298 K, modulation amplitude: 0.5 G, scans: 20, gain: 30 dB.

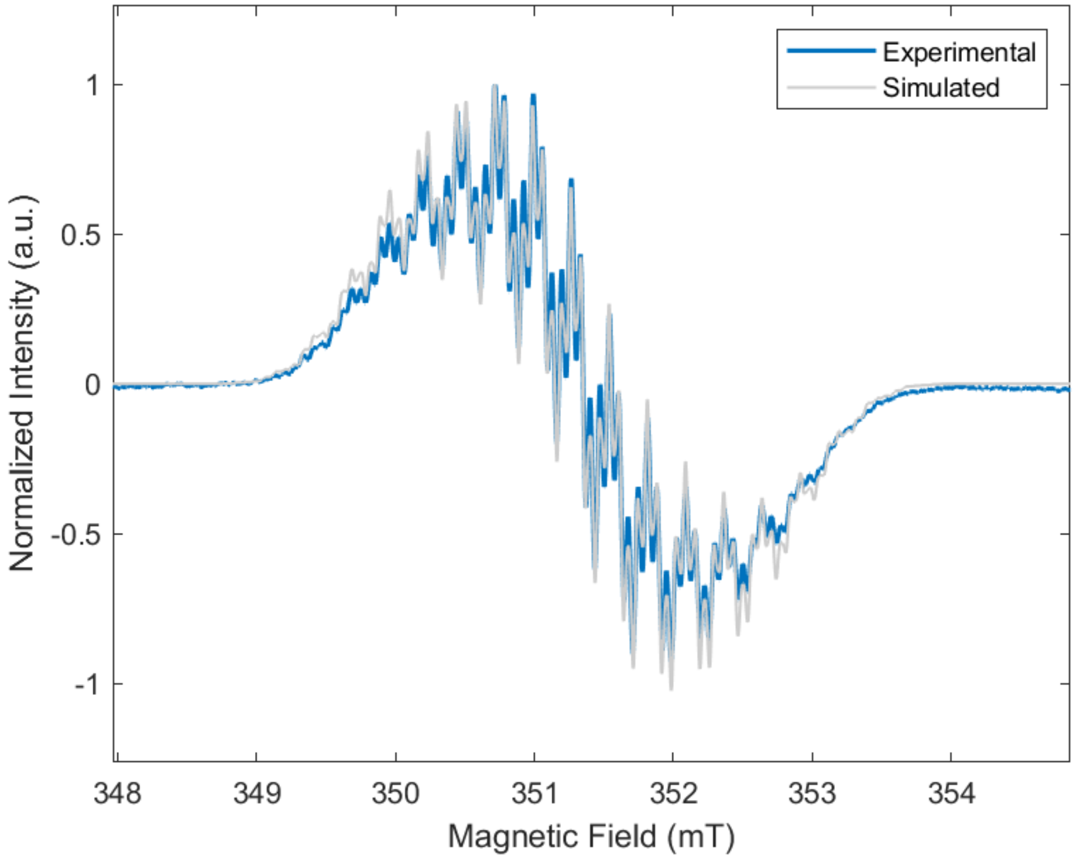

Supplementary Figure 47: Continuous wave EPR spectrum of [K(crypt)][**5**] (blue) overlayed with the simulated spectrum (grey, ×2 A_N = 15.38 MHz, ×2 A_N = 0.87 MHz, ×4 A_H = 5.32 MHz, ×4 A_H = 2.29 MHz, ×6 A_H = 7.92 MHz, g = 2.003, lw = 0.05). Experimental parameters: solvent: *o*DFB/Tol, frequency: 9.852 GHz, temperature: 298 K, modulation amplitude: 0.5 G, scans: 20, gain: 30 dB.

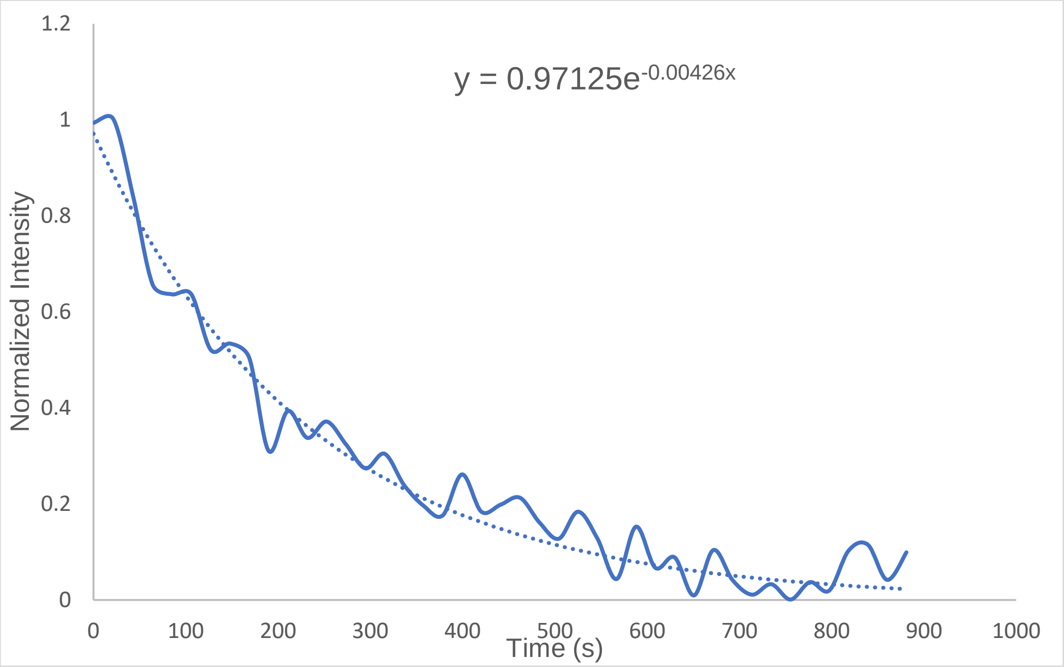

Supplementary Figure 48: Decay curve of the EPR signal intensity for [K(crypt)][**5**] in THF with the exponential equation shown.

Supplementary Table 30: Decay curve equation and corresponding half-life of [K(crypt)][**5**]

Compound	Equation	Half-life (s)
[K(crypt)][**5**]	$y = 0.97125e^{-0.00426x}$	155.9 (2.60 mins)

5.4 Cyclic Voltammetry Data of [K(crypt)][5]

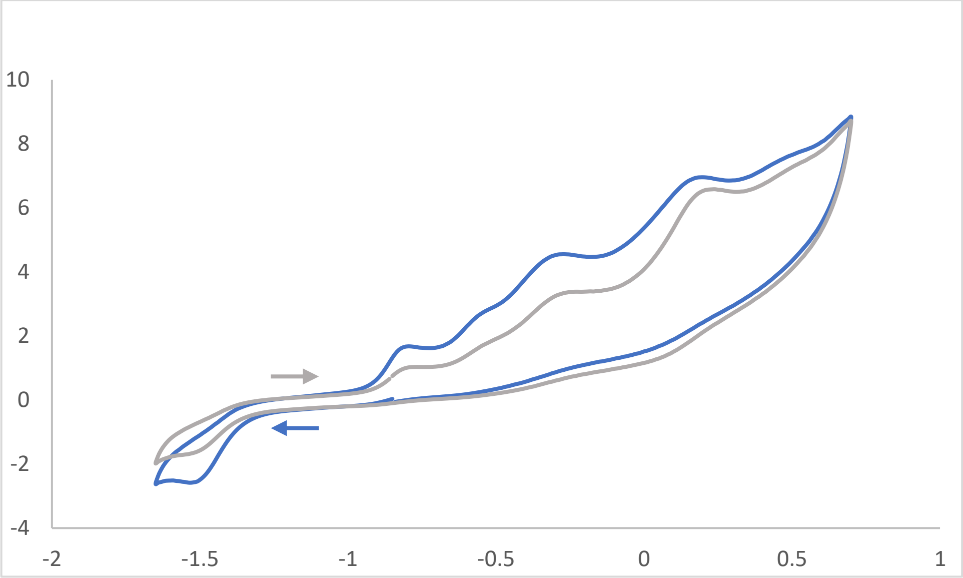

Supplementary Figure 49: Cyclic Voltammetry of [K(crypt)][**5**] 3 mM in THF with $[nBu_4N][PF_6]$ electrolyte at 0.1 V/s starting at –0.8 V and scanning independently in the positive direction first (grey trace) and the negative direction first (blue trace). Glassy carbon working electrode, platinum wire counter electrode, and leak-proof Ag/AgCl reference electrode were used.

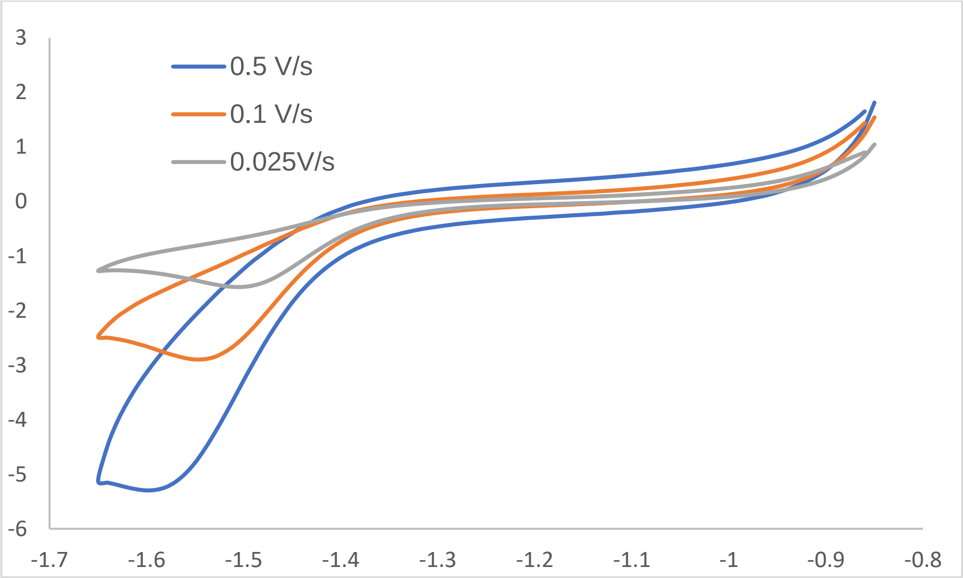

Supplementary Figure 50: Cyclic Voltammetry of [K(crypt)][**5**] 0.3 M in THF with $[nBu_4N][PF_6]$ electrolyte at varying scan rates on first reduction wave using glassy carbon working electrode, platinum wire counter electrode, and leak-proof Ag/AgCl reference electrode.

5.5 UV-Vis spectrum and NTOs of [K(crypt)][5]

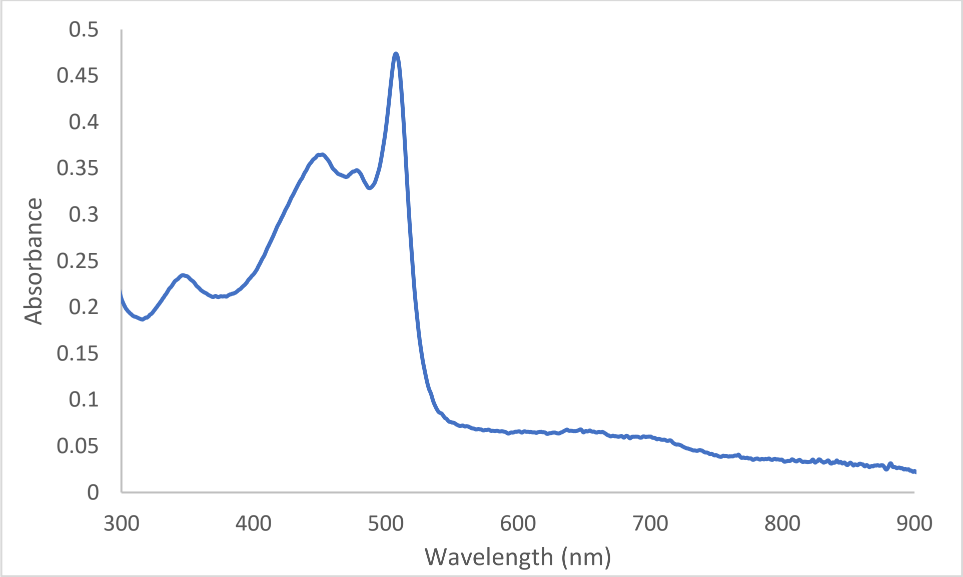

Supplementary Figure 51: UV-Vis spectrum of [K(crypt)][**5**] at an initial concentration of 0.05 mM in *o*DFB.

Supplementary Table 31: TD-DFT calculated UV wavelengths, oscillator strengths, natural transition orbitals (NTOs), and their occupations with blue and grey dots for selected excited states of $[\mathbf{5}]^{\bullet-}$ (calculated at TPSSh/def2-TZVP/SMD(cyclopentanone) level of theory).

Excited State	Wavelength	Oscillator Strength	NTO Occupation Number		
1	344.81	0.0559	0.136	1.705	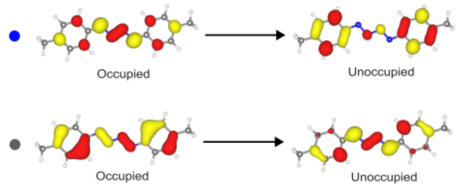
2	423.06	0.2764	1.811	0.125	
3	485.11	1.1991	1.081	0.879	
4	611.95	0.0115	0.948	1.026	

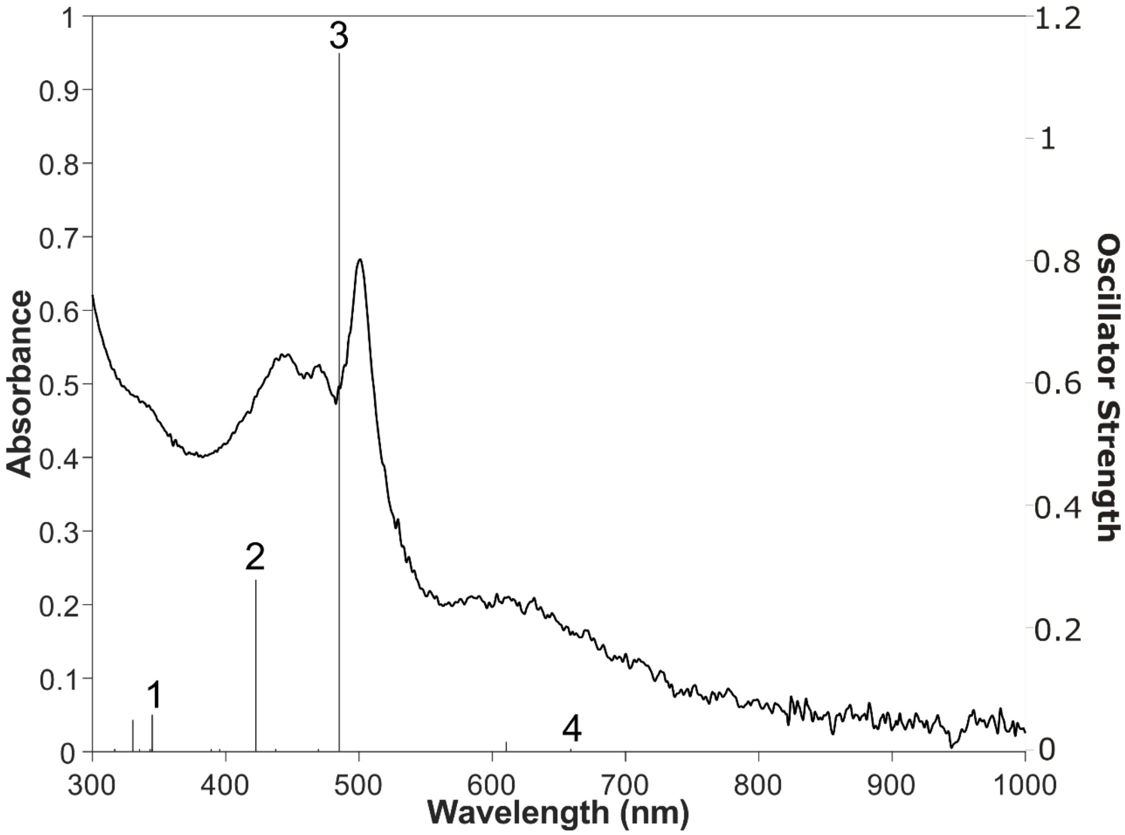

Supplementary Figure 52: Overlayed experimental UV-Vis spectrum of [K(crypt)][**5**] and calculated oscillator strengths of [**5**]$^{\bullet-}$ at TPSSh/def2-TZVP/SMD(cyclopentanone) level of theory.

6. Synthesis and Characterization of [K(crypt)][6]

6.1 Synthesis of [K(crypt)][6]

In the glovebox, KC_8 (100 mg, 0.74 mmol, 1 equiv.) and 2.2.2-cryptand (280 mg, 0.74 mmol, 1 equiv.) were suspended in THF in a J-young ampoule. Azidobenzene (176 mg, 1.48 mmol, 2 equiv.) was added to the reaction mixture, and the reaction mixture was stirred for 10 minutes. Then, the solution was filtered and diethyl ether added to precipitate a black solid. The solid was filtered and washed with additional diethyl ether (3 x10 mL) before drying under vacuum yielding [K(crypt)][**6**] as a black crystalline solid.

Isolated Yield: 200 mg, 43%

6.2 Charge and Spin Densities of [6]•–

Supplementary Table 32: Calculated charge distributions of [**6**]•– at TPSS/def2-TZVP/SMD(THF) level of theory.

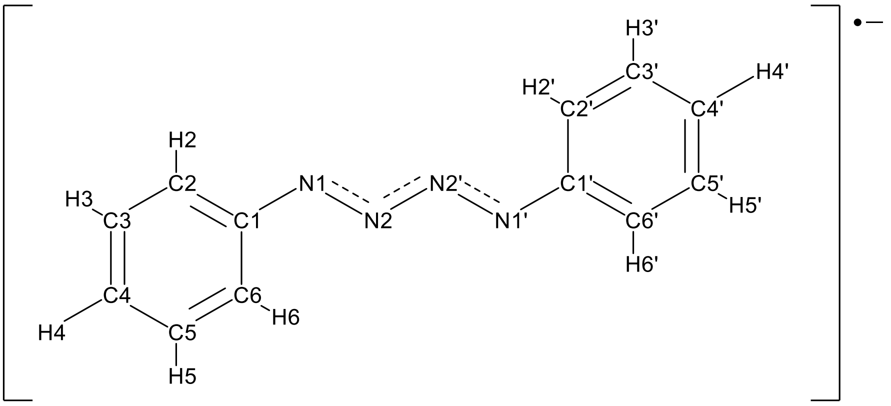

Atom	Charge Distribution		
	NPA	Hirshfeld	Mulliken
N1	–0.314	–0.204	–0.087
N2	–0.164	–0.131	–0.162
C1	0.072	–0.005	0.089
C2	–0.221	–0.068	–0.229
H2	0.214	0.031	0.086
C3	–0.225	–0.060	–0.137
H3	0.212	0.039	0.124
C4	–0.249	–0.070	–0.147
H4	0.210	0.036	0.122
C5	–0.216	–0.058	–0.144
H5	0.210	0.039	0.127
C6	–0.251	–0.072	–0.228
H6	0.222	0.022	0.086
N1’	–0.314	–0.204	–0.087
N2’	–0.164	–0.131	–0.162
C1’	0.072	–0.005	0.089
C2’	–0.221	–0.068	–0.229
H2’	0.214	0.031	0.086
C3’	–0.225	–0.060	–0.137
H3’	0.212	0.039	0.124
C4’	–0.249	–0.070	–0.147
H4’	0.210	0.036	0.122
C5’	–0.216	–0.058	–0.144

H5’	0.210	0.039	0.127
C6’	–0.251	–0.072	–0.228
H6’	0.222	0.022	0.086
Total	–1.000	–1.000	–1.000

Supplementary Table 33: Calculated spin densities of [**6**]$^{\bullet -}$ at TPSS/def2-TZVP/SMD(THF) level of theory.

**Atom**	**Spin Densities**		
	**NPA**	**Hirshfeld**	**Mulliken**
N1	0.272	0.232	0.271
N2	0.054	0.067	0.049
C1	–0.043	0.005	–0.041
C2	0.086	0.060	0.093
H2	–0.003	0.003	–0.005
C3	–0.035	–0.009	–0.044
H3	0.001	–0.001	0.002
C4	0.112	0.077	0.121
H4	–0.004	0.006	–0.007
C5	–0.039	–0.010	–0.046
H5	0.001	–0.001	0.002
C6	0.101	0.066	0.113
H6	–0.003	0.005	–0.007
N1’	0.272	0.232	0.270
N2’	0.054	0.067	0.049
C1’	–0.043	0.005	–0.041
C2’	0.086	0.060	0.093
H2’	–0.003	0.003	–0.005
C3’	–0.035	–0.009	–0.044
H3’	0.001	–0.001	0.002
C4’	0.112	0.077	0.121
H4’	–0.004	0.006	–0.007
C5’	–0.039	–0.010	–0.046
H5’	0.001	–0.001	0.002
C6’	0.101	0.066	0.113
H6’	–0.003	0.005	–0.007
Total	1.000	1.000	1.000

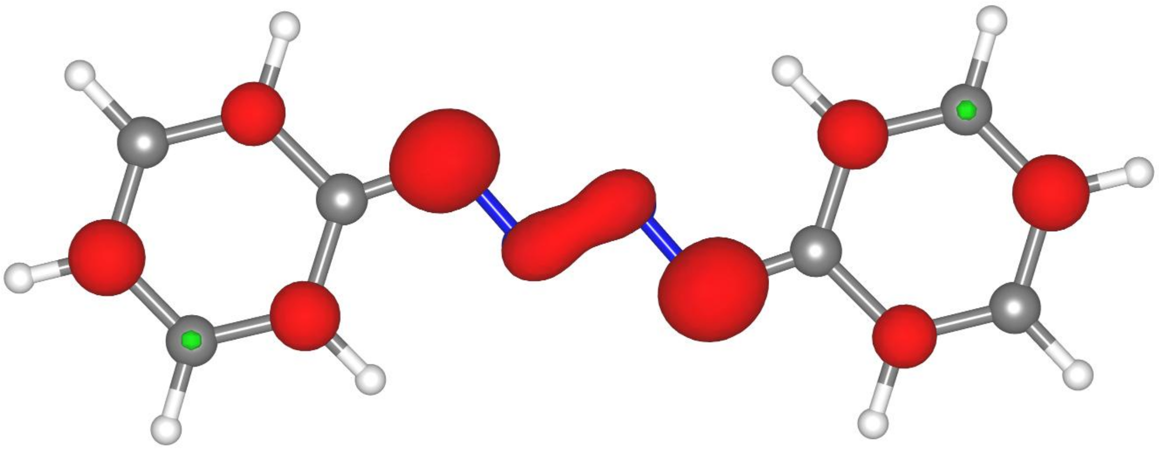

Supplementary Figure 53: Spin density plot (front-on) of [**6**]$^{•-}$ with isovalue = 0.005.

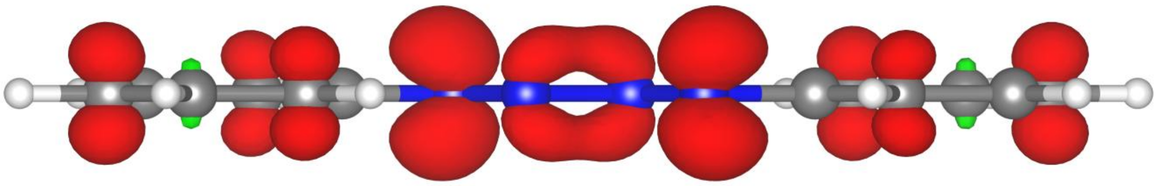

Supplementary Figure 54: Spin density plot (side-on) of [**6**]$^{•-}$ with isovalue = 0.005.

6.3 EPR data of [K(crypt)][6]

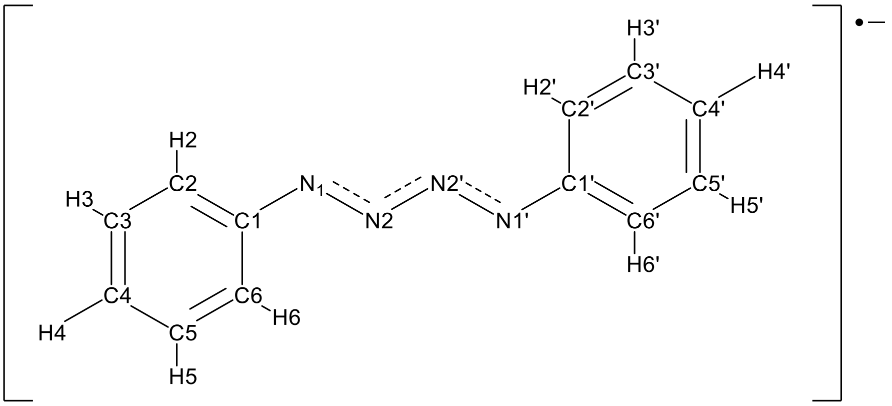

Supplementary Table 34: Calculated A_{iso} values of [**6**]$^{\bullet-}$ at EPR-III /B3LYP/SMD (THF) level of theory.

Atom	A_{iso} (MHz)	Atom	A_{iso} (MHz)
N1	13.389583	N1’	13.389374
N2	0.102385	N2’	0.102745
H2	–6.903429	H2’	–6.903548
H3	2.875280	H3’	2.875202
H5	2.958286	H5’	2.958234
H6	–7.839407	H6’	–7.839353
H4	–8.521670	H4’	–8.522039

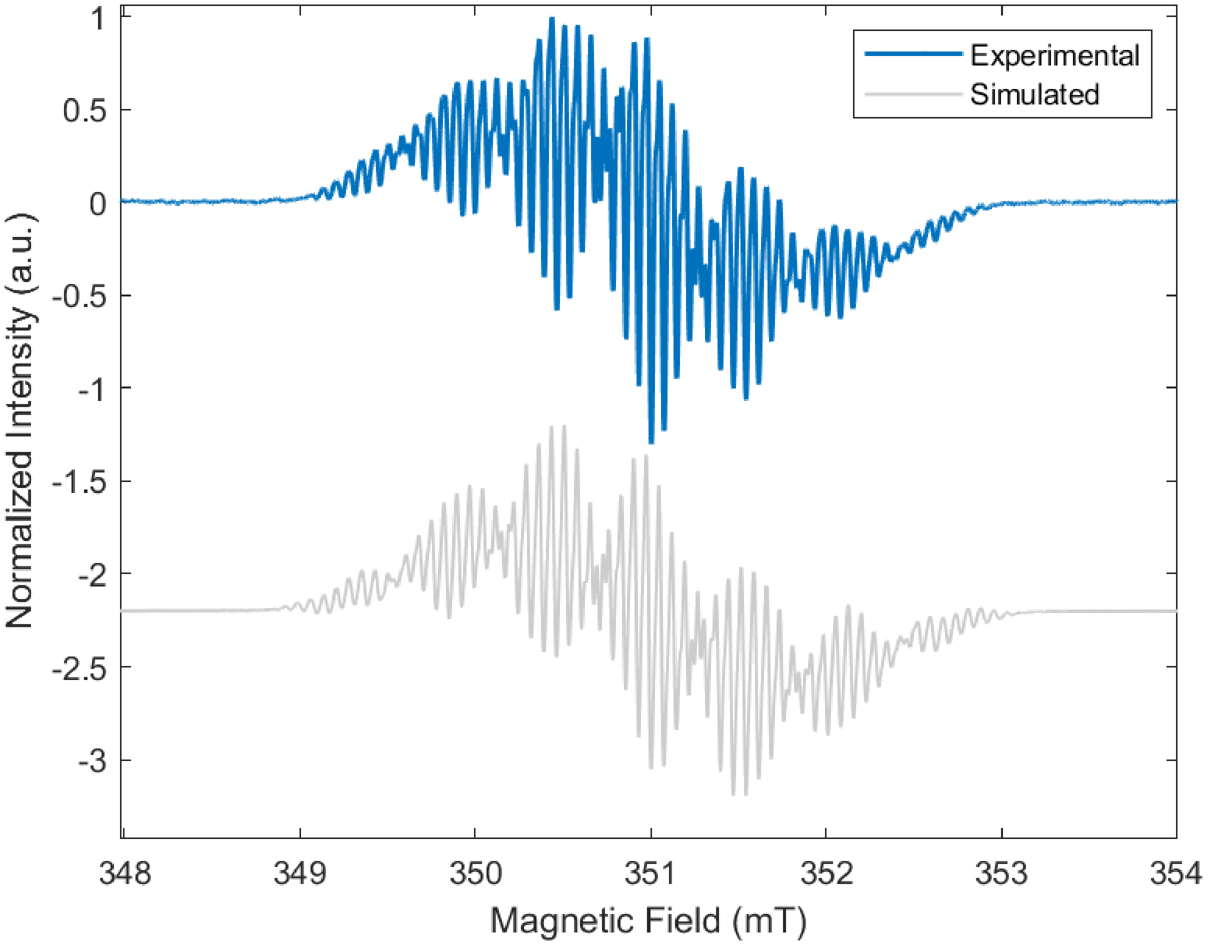

Supplementary Figure 55: Continuous wave EPR spectrum of [K(crypt)][**6**] (blue) stacked above the simulated spectrum (grey, ×2 A_N = 13.13 MHz, 2 A_N = 2.23, MHz, ×4 A_H = 8.06 MHz, ×4 A_H = 1.84 MHz, ×2 A_H = 9.09 MHz, g = 2.004, lw = 0.001, 0.056). Experimental parameters: solvent: THF, frequency: 9.844 GHz, temperature: 298 K, modulation amplitude: 0.2 G, scans: 20, gain: 30 dB.

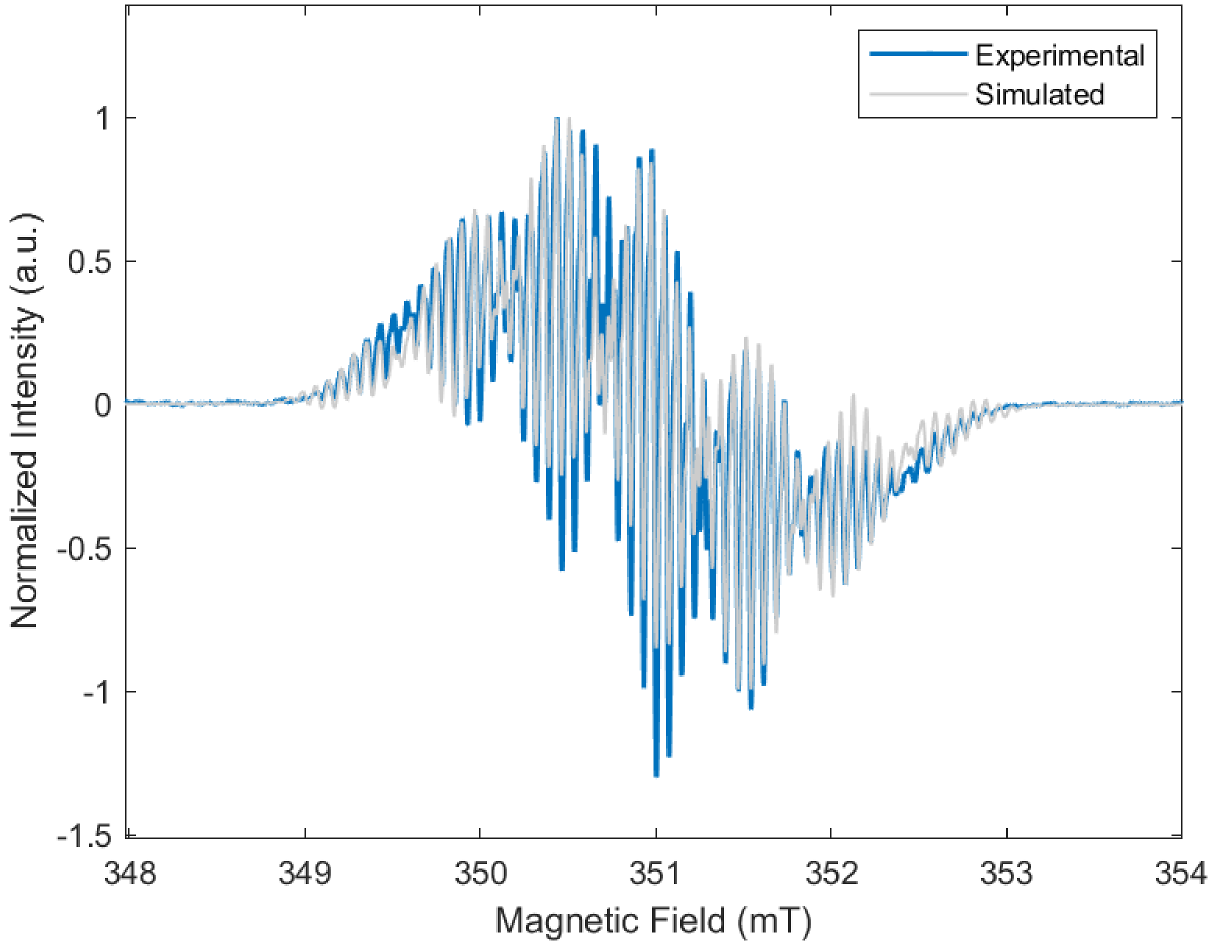

Supplementary Figure 56: Continuous wave EPR spectrum of [K(crypt)][**6**] (blue) overlayed with the simulated spectrum (grey, ×2 A_N = 13.13 MHz, 2 A_N = 2.23, MHz, ×4 A_H = 8.06 MHz, ×4 A_H = 1.84 MHz, ×2 A_H = 9.09 MHz, g = 2.004, lw = 0.001, 0.056). Experimental parameters: solvent: THF, frequency: 9.844 GHz, temperature: 298 K, modulation amplitude: 0.2 G, scans: 20, gain: 30 dB.

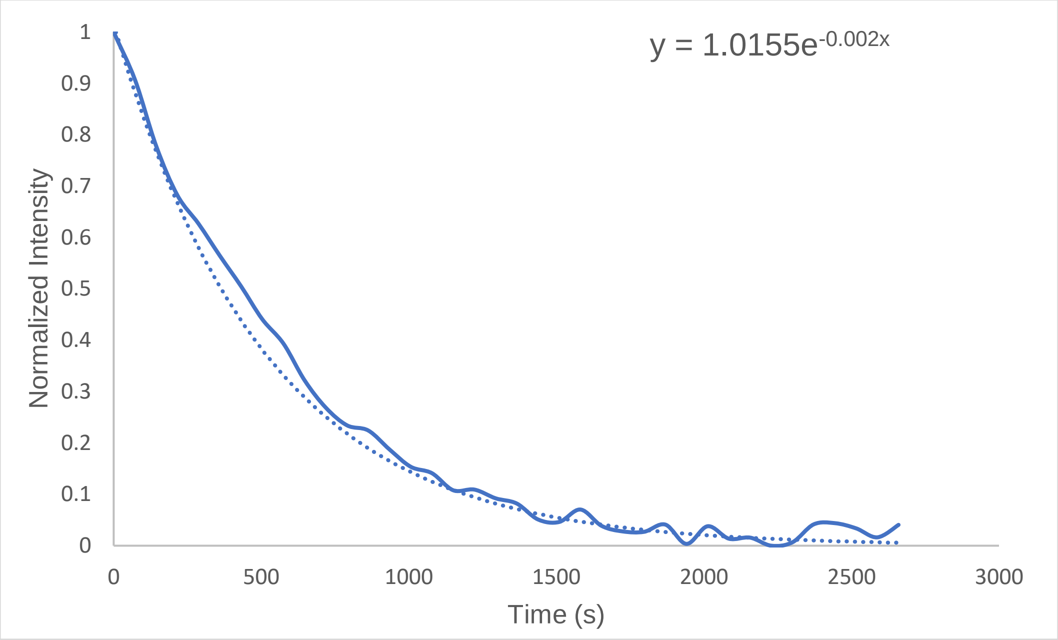

Supplementary Figure 57: Decay curve of the EPR signal Intensity for [K(crypt)][**6**] in THF with the exponential equation shown.

Supplementary Table 35: Decay curve equation and corresponding half-life of [K(crypt)][**6**]

Compound	Equation	Half-life (s)
[K(crypt)][6]	$y = 1.0155e^{-0.002x}$	354.3 (5.91 mins)

Supplementary Table 36: Spin counting for compound [K(crypt][**6**]

Compound	Actual Concentration (mM)	Number of Spins ($x10^{15}$)	Measured Concentration (mM)
[K(crypt)][**6**]	0.56	28.58	0.55

6.4 Cyclic Voltammetry Data of [K(crypt)][6]

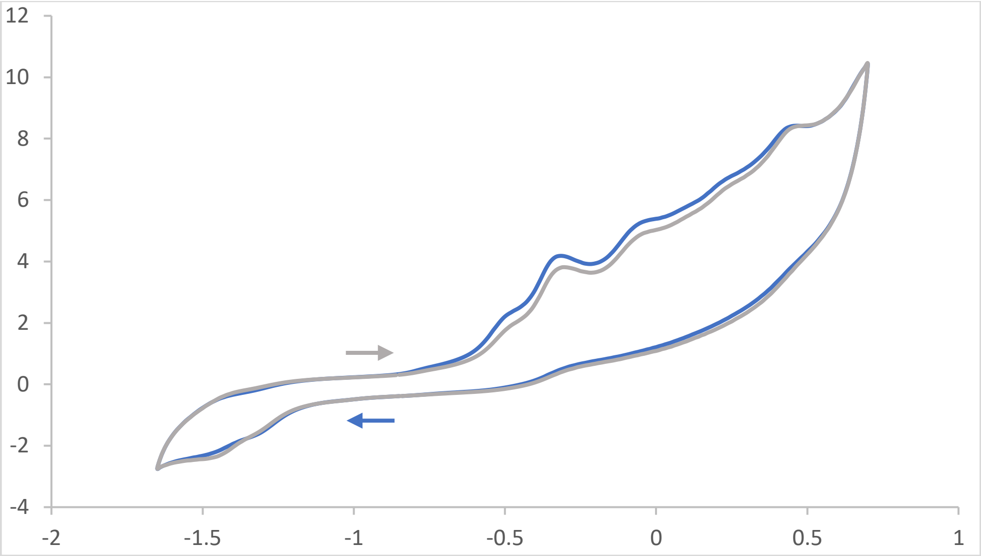

Supplementary Figure 58: Cyclic Voltammetry of [K(crypt)][**6**] 3 mM in THF with [nBu_4N][PF_6] electrolyte at 0.1 V/s starting at –0.8 V and scanning independently in the positive direction first (grey trace) and the negative direction first (blue trace). Glassy carbon working electrode, platinum wire counter electrode, and leak-proof Ag/AgCl reference electrode were used.

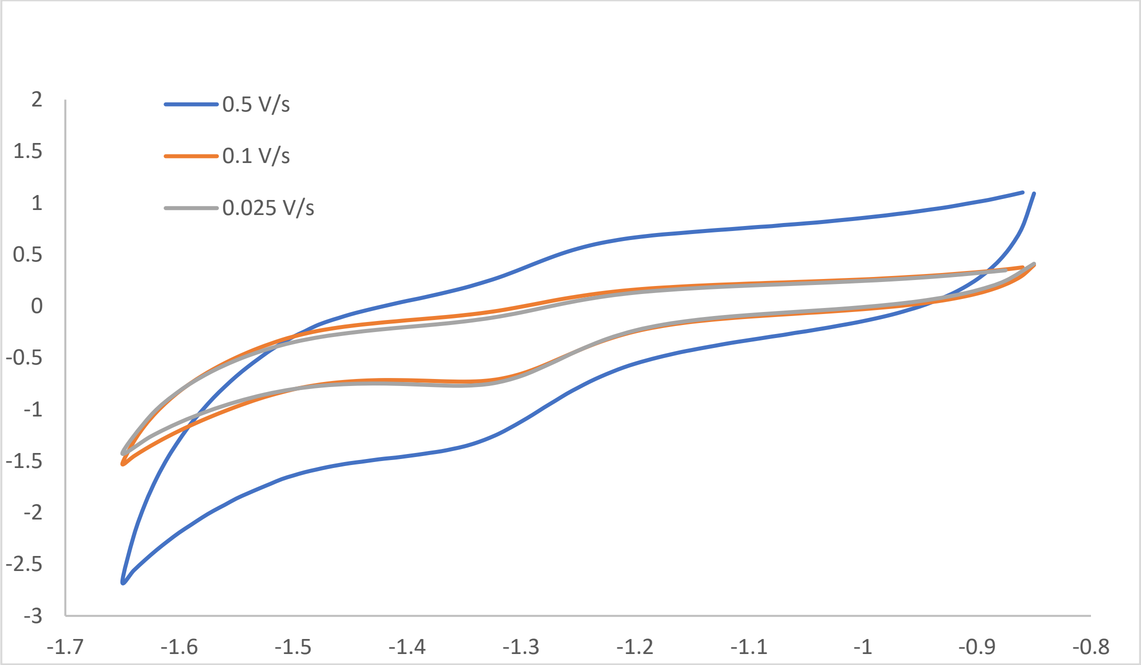

Supplementary Figure 59: Cyclic Voltammetry of [K(crypt)][**6**] 0.3 M in THF with [nBu_4N][PF_6] electrolyte at varying scan rates on first reduction wave using glassy carbon working electrode, platinum wire counter electrode, and leak-proof Ag/AgCl reference electrode.

6.5 UV-Vis spectrum and NTOs of [K(crypt)][6]

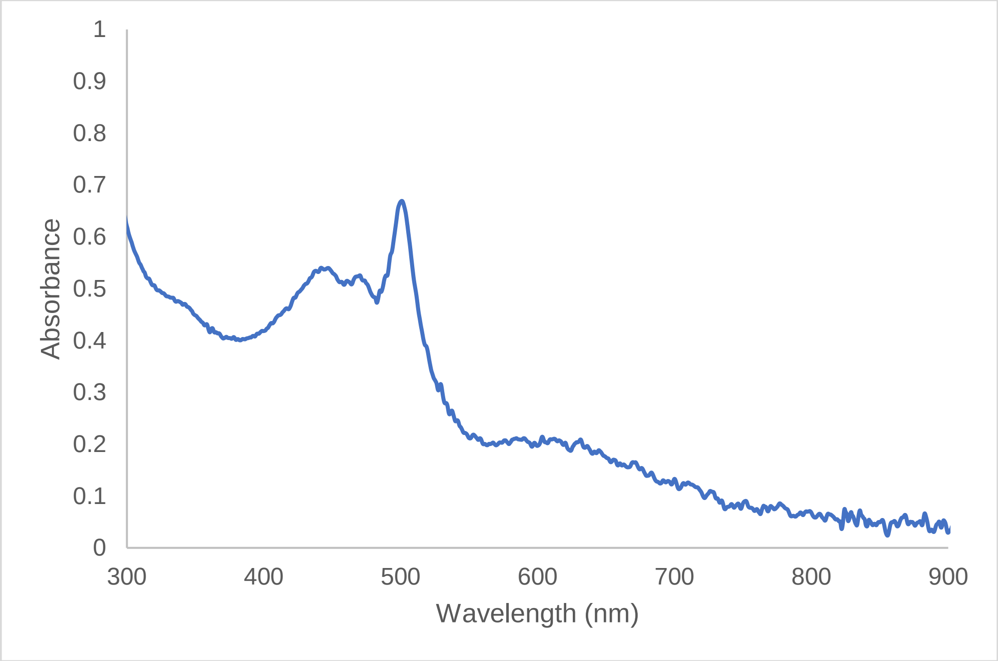

Supplementary Figure 60: UV-Vis spectrum of [K(crypt)][**6**] at an initial concentration of 0.05 mM in *o*DFB.

Supplementary Table 37: TD-DFT calculated UV wavelengths, oscillator strengths, natural transition orbitals (NTOs), and their occupations with blue and grey dots for selected excited states of [**6**]$^{\bullet-}$ (calculated at TPSSh/def2-TZVP/SMD(cyclopentanone) level of theory).

Excited State	Wavelength	Oscillator Strength	NTO Occupation Number		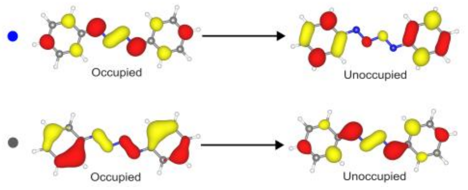
1	348.58	0.0490	0.116	1.778	
2	418.61	0.2237	1.796	0.144	
3	472.01	1.1316	1.065	0.890	
4	595.18	0.0077	0.961	1.010	

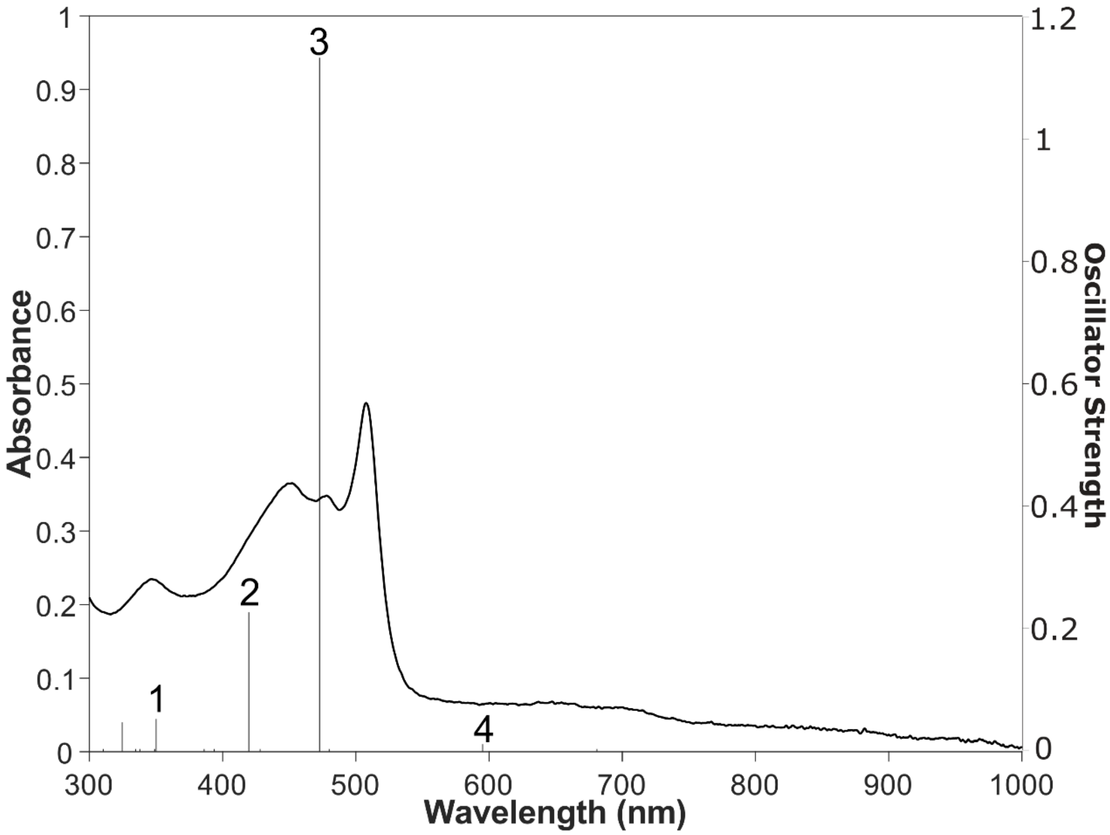

Supplementary Figure 61: Overlayed experimental UV-Vis spectrum of [K(crypt)][**6**] and calculated oscillator strengths of [**6**]$^{\bullet-}$ at TPSSh/def2-TZVP/SMD(cyclopentanone) level of theory.

7. Comparison of Derivatives

The solid-state structures of [K(crypt)][**1**], [K(crypt)][**3**], [K(crypt)][**4**], and [K(crypt)][**5**] showed similar bond lengths and bond angles around the {N_4} unit, with the only outlier being a slightly shorter N1–N2 bond in [K(crypt)][**5**]. The DFT computed bond metrics of these compounds are in good agreement with the calculated bond lengths, varying by less than 0.01 Å, and calculated bond angles, varying by less than 0.2°**.** The Wiberg bond index values and charge density distributions are also consistent throughout the series.

The Mulliken spin density at N1 decreases as the *para* substituent descends group 17 with [K(crypt)][**3**] having 58% of the spin at N1 compared to 54% in [K(crypt)][**4**] and 53% in [K(crypt)][**1**]. However, the spin densities at N1 of [K(crypt)][**5**] (55%) and [K(crypt)][**6**] (54%) are almost the same. The spin density at N2 also remains consistently small throughout the series. Continuous wave EPR studies and subsequent simulations of the series are in good agreement with the calculated spin densities. Each derivative shows the highest coupling to the terminal nitrogens (N1 and N1') followed by the *ortho* aromatic carbons. The similarity to benzylic radicals, as discussed in the manuscript, can be seen throughout the coupling constants with [K(crypt)][**3**] having a notably large A_F = 13.25 MHz compared to the smaller A_{Cl} = 0.83 MHz and A_{Br} = 2.05 MHz of [K(crypt)][**4**] and [K(crypt)][**1**], respectively.[40] Coupling at the N2 positions remains small across the series, with inclusion of the coupling in the simulation always being within the linewidth of the spectra. The half-life of the radicals decreases as the substituent changes in the order: [K(crypt)][**1**] > [K(crypt)][**4**] > [K(crypt)][**3**] > [K(crypt)][**6**] > [K(crypt)][**5**]. It is worth noting that [K(crypt)][**5**] has a half-life so short (156 seconds in THF) that it decomposes too quickly for an accurate application of the spin-counting experiment.

Analysis of the cyclic voltammograms shows that only [K(crypt)][**1**] and [K(crypt)][**4**] demonstrate electrochemically reversible reductions, with the other derivatives decomposing upon reduction. These data suggests that the corresponding dianions of [K(crypt)][**3**], [K(crypt)][**5**], and [K(crypt)][**6**] are likely unstable. The minimum point in the reduction waves (used instead of redox potential, as this cannot be obtained for the irreversible reduction events) becomes less negative as the group 17 substituent is changed from F to Cl to Br, and [K(crypt)][**5**] gives the most negative reduction potential.

The UV-Vis spectra of the series are largely consistent with the same general spectral shape, although in [K(crypt)][**3**] the first two absorption bands are larger and more defined. The λ_{max} of the four excited states are also similar across the series, and the NTO computed transitions show for each compound these excitations are governed by two transitions, similar to those observed for [K(crypt)][**1**]. In the case of [K(crypt)][**3**], there are slightly different occupations

for these transitions in the first and second excited states, in line with the slightly different appearance of the spectrum.

Supplementary Table 38: Comparison of key data discussed between derivatives [K(crypt)][**1**], [K(crypt)][**3**], [K(crypt)][**4**], [K(crypt)][**5**], and [K(crypt)][**6**].

**Compound**	**N1–N2 bond length (Å)**	**N2–N2' bond length (Å)**	**N1 Computed Spin (%, Mulliken)**	**A_N of N1 (MHz)**	**A_X of *para* substituent (MHz)**	**Half-life (s, THF)**	**Reduction wave minima (v)**
[K(crypt)][**1**]	1.316	1.322	52.6	14.61	2.05	2540.3	–1.21
[K(crypt)][**3**]	1.308	1.339	57.6	15.33	13.25	546.9	–1.48
[K(crypt)][**4**]	1.314	1.334	53.6	14.64	0.83	727.6	–1.34
[K(crypt)][**5**]	1.311	1.316	55.4	15.38	7.92	155.9	–1.59
[K(crypt)][**6**]	-	-	54.2	13.13	9.09	354.3	–1.35

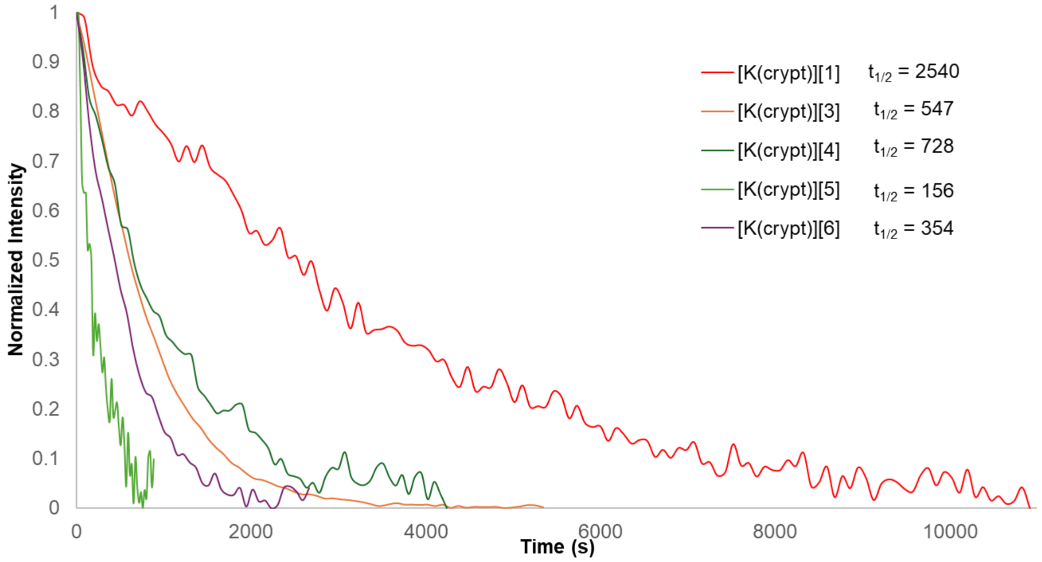

Supplementary Figure 62: Overlayed decay curves of the EPR signal intensity in THF of [K(crypt)][**1**] (red), [K(crypt)][**3**] (orange), [K(crypt)][**4**] (dark green), [K(crypt)][**5**] (light green), and [K(crypt)][**6**] (purple) with the half-life given in seconds. Note: decay curves were collected in THF, as tuning the spectrometer takes longer when oDFB is used as the solvent, and there was significant loss of material for the shorter lifetime radical anions.

8. Reactivity Studies

8.1 [K(THF)$_2$]$_2$[2]

8.1.1 Synthesis of [K(THF)$_2$]$_2$[2]

In a vial in the glovebox, 4-BrC$_6$H$_4$N$_3$ (29.5 mg, 0.235 mmol, 1 equiv.) and KC$_8$ (31.5 mg, 0.235 mmol, 1 equiv.) were suspended in THF resulting in a red solution. The reaction mixture was filtered and hexane added to the filtrate yielding a red solid. Recrystallization from vapor diffusion of hexane into a THF solution at –40 °C yielded crystals of [K(THF)$_2$]$_2$[**2**] suitable for XRD studies.

Author's Note: The crystal consists of two isomers (*cis*- and *trans*-) with the single crystal XRD occupancy of the *cis*- isomer being 47% and the *trans*- isomer being 53%. NMR spectroscopy studies were conducted on the crude material, and further characterization and purification could not be obtained as [K(THF)$_2$]$_2$[**2**] explodes when shocked under an inert atmosphere or when exposed to air. Further, crystals of [K(THF)$_2$]$_2$[**2**] were found to decompose on the slide during single crystal mounting, with bubbles forming in the oil (see Supplementary Figure 67).

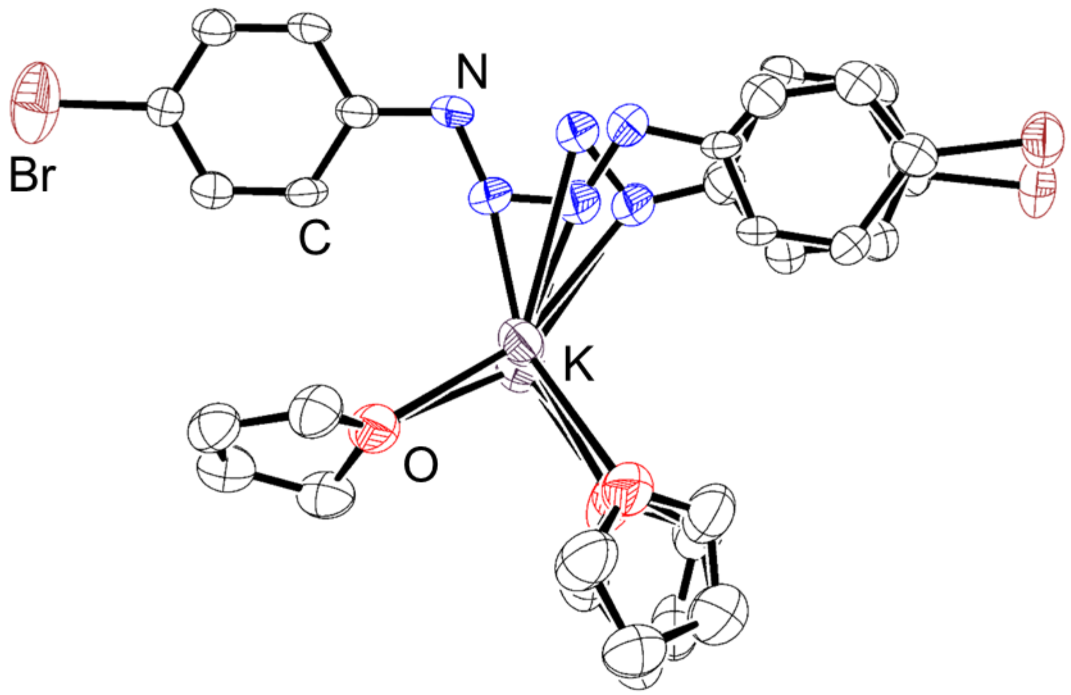

Supplementary Figure 63: Molecular structure of the asymmetric unit cell of [K(THF)$_2$]$_2$[**2**] showing anisotropic displacement ellipsoids at 50% probability with hydrogen atoms omitted for clarity. Disorder in bromines omitted for clarity. Nitrogen: blue; carbon: white; bromine: brown; potassium: violet; oxygen: red.

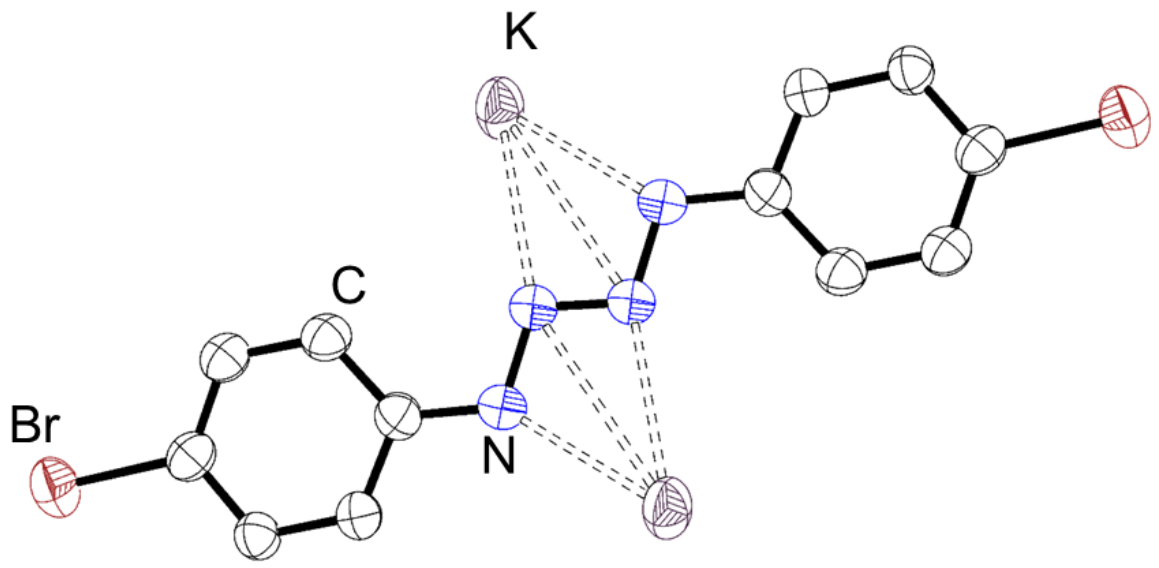

Supplementary Figure 64: Molecular structure of the *trans*- isomer of [K(THF)$_2$]$_2$[**2**] showing anisotropic displacement ellipsoids at 50% probability with hydrogen atoms and solvent molecules omitted for clarity. Nitrogen: blue; carbon: white; bromine: brown; potassium: violet; oxygen: red.

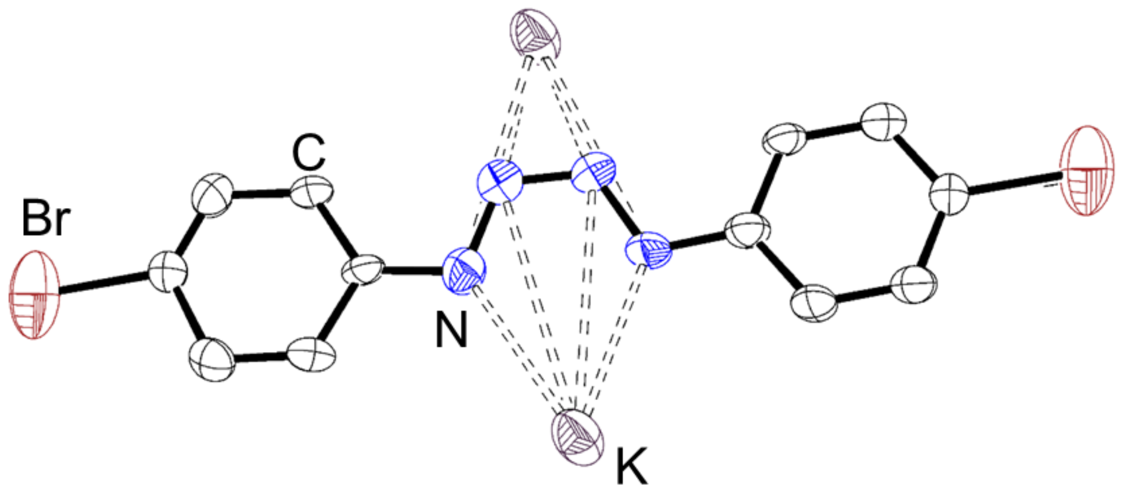

Supplementary Figure 65: Molecular structure of the *cis*- isomer of [K(THF)$_2$]$_2$[**2**] showing anisotropic displacement ellipsoids at 50% probability with hydrogen atoms and solvent molecules omitted for clarity. Nitrogen: blue; carbon: white; bromine: brown; potassium: violet; oxygen: red.

8.1.2 Investigation of *cis-* and *trans-* isomer interconversion

By allowing the occupancies in the crystal structure of $[K(THF)_2]_2$[**2**] to freely refine, a *cis*: *trans* ratio of 47: 53 could be found. Variable temperature NMR studies were conducted to understand the interconversion between the isomers in solution. At room temperature, we can see only one very broad hump in the aromatic region showing the interconversion of the 2 aromatic signals of each isomer. At –60°C, the signals begin to decoalesce with one large resonance and two smaller resonances being observed. This large resonance also begins to decoalesce at –80°C where we can identify the 4 signals that we expect for the presence of both a *cis*- and *trans*- isomer aromatic resonances. As the signals do not fully decoalesce at –80°C, energy barriers associated with the conversion cannot be determined.

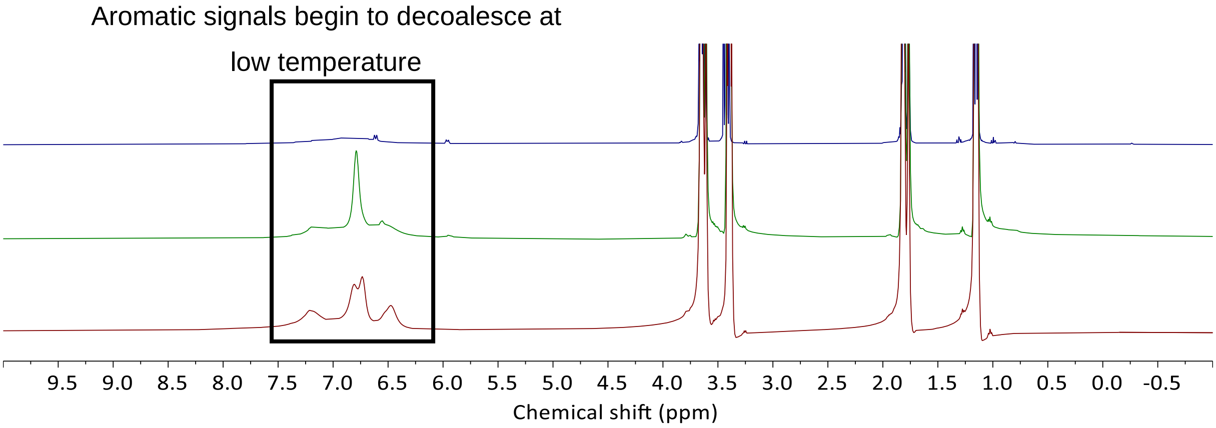

Supplementary Figure 66: Crude ^{1}H NMR(THF-d_8, 400 MHz) spectra of $[K(THF)_2]_2$[**2**] at 25°C (top), –60°C (middle) and –80°C (bottom).

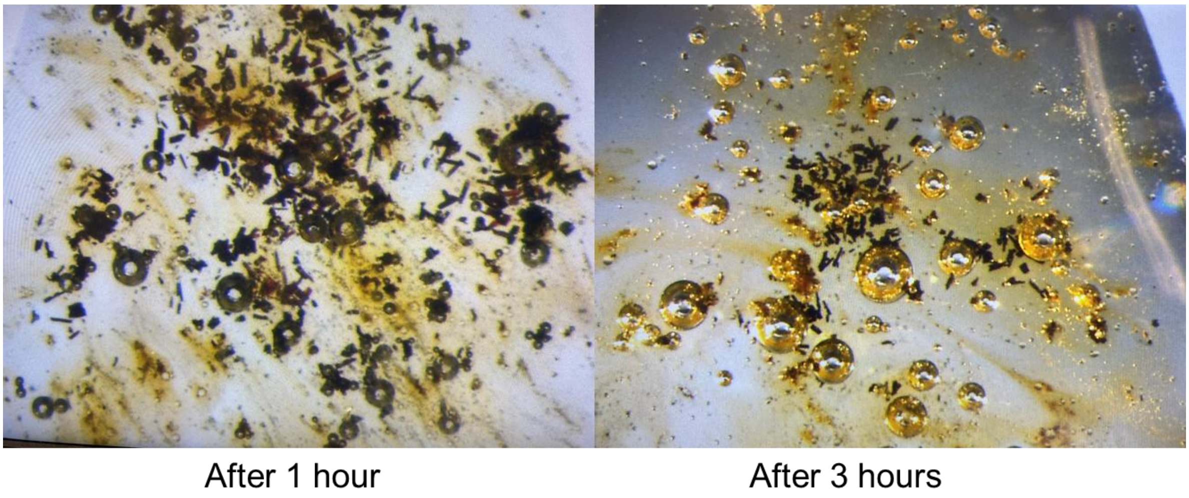

Supplementary Figure 67: Crystals of $[K(THF)_2]_2$[**2**] decomposing and releasing gas bubbles (presumably N_2) on a glass slide in air.

Supplementary Table 39: Electronic (E) and Gibbs (G) energetic differences between *cis*- and *trans*-isomers of $[K(THF)_2]_2$[**2**], calculated at the TPSS/def2-TZVP level of theory (THF omitted).

	**E (kcal/mol)**	**G (kcal/mol)**
Energetic difference (*trans* – *cis*)	–4.34	–3.81

8.2 Addition of Ph_3SnH to [K(crypt)][1]

8.2.1 Addition of Ph_3SnH to [K(crypt)][1] with crude NMR

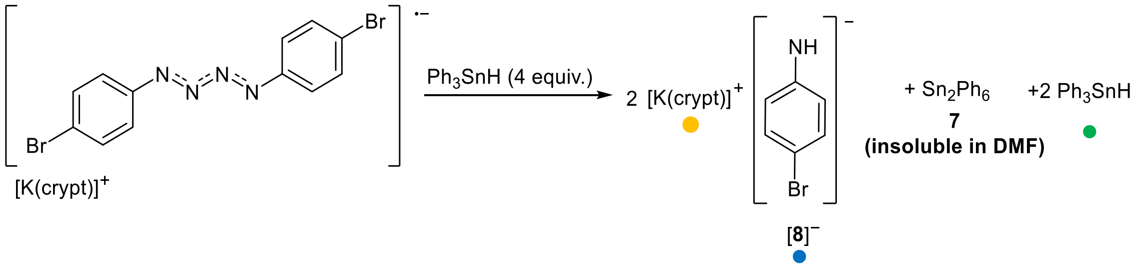

In the glovebox, a solution of Ph_3SnH (12.7 mg, 0.102 mmol, 4 equiv.) in DMF-d_7 (0.5 mL) was added to a vial containing [K(crypt)][**1**] (30.0 mg, 0.039 mmol, 1 equiv.). The solution was shaken for 1 minute before transferring to a J Young NMR tube for analysis by NMR spectroscopy.

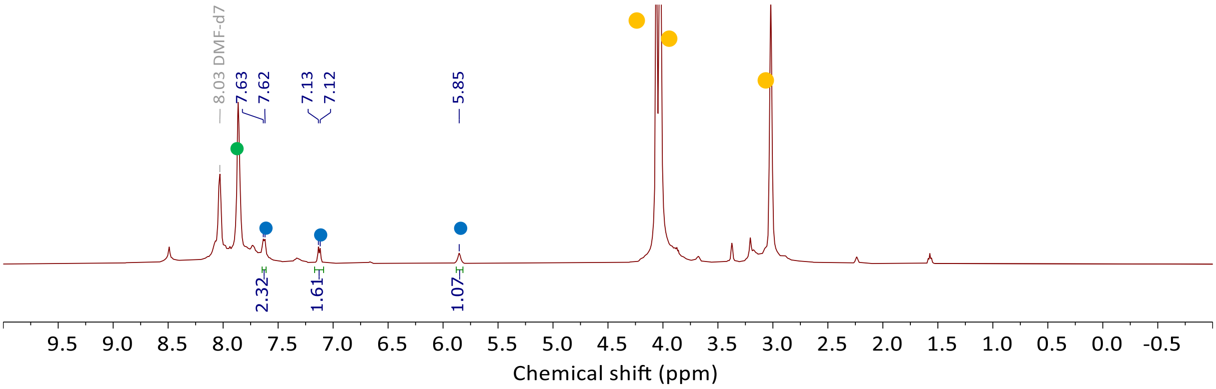

Supplementary Figure 68: Crude ^1H NMR spectrum (400 MHz, DMF-d_7) of reaction 8.2.1.

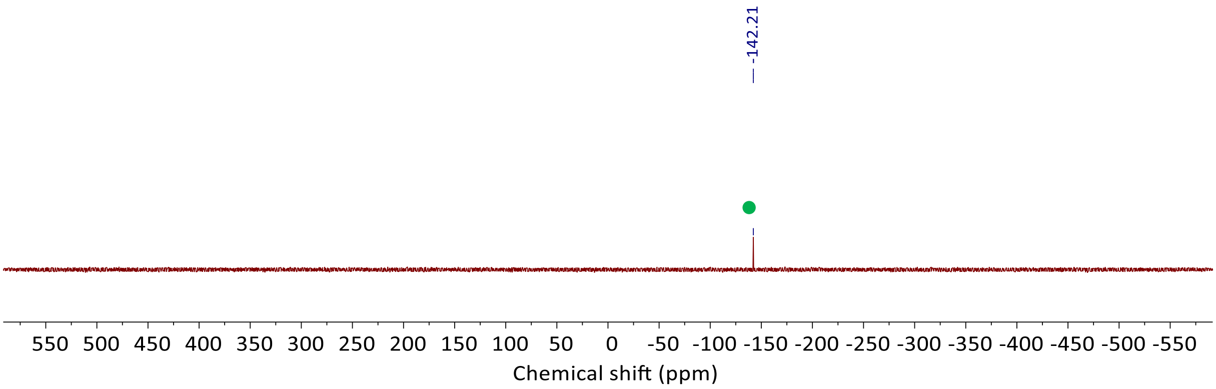

Supplementary Figure 69: ^{119}Sn NMR spectrum (149 MHz, DMF-d_7) of reaction 8.2.1 showing unreacted Ph_3SnH.

8.2.2 Addition of Ph_3SnH to [K(crypt)][1] with aqueous workup

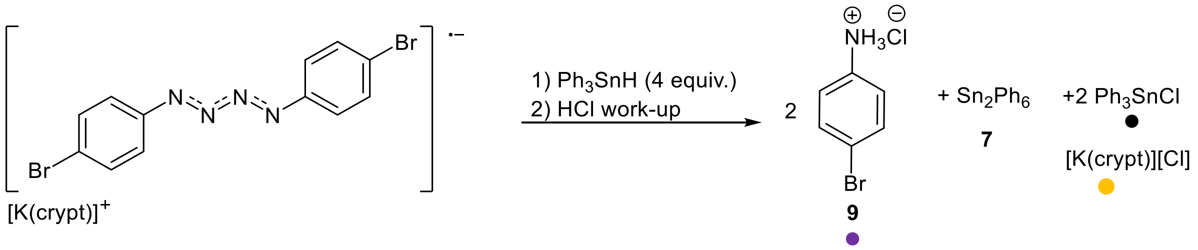

In the glovebox, a solution of Ph_3SnH (12.7 mg, 0.102 mmol, 4 equiv.) in DMF (1 mL) was added to a vial containing [K(crypt)][**1**] (30.0 mg, 0.039 mmol, 1 equiv.). The solution was stirred for 30 minutes yielding Sn_2Ph_6 (**7**) as a white solid which was filtered and recrystallized from benzene.[41] HCl in diethyl ether was added to the filtrate and the precipitate filtered and dried yielding 4-bromoanilinium chloride (**9**) which was analyzed by NMR spectroscopy. NMR yields was obtained using Si_2Me_6 (10 µL) as internal standard (^{1}H δ = 0.08 ppm).

NMR conversion of 4-bromophenylammonium chloride (9): 57% (vs Si_2Me_6 internal standard).

Isolated Yield of Sn_2Ph_6 (7): 5.4 mg (85%).

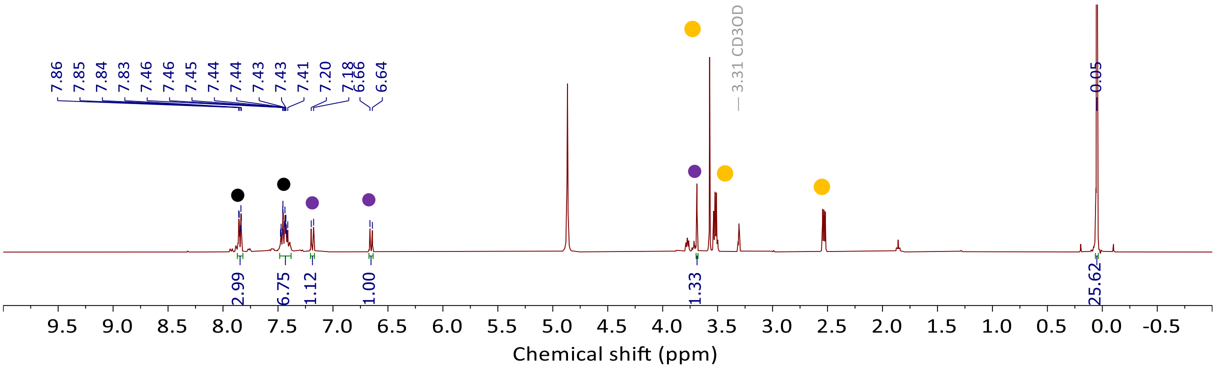

Supplementary Figure 70: ^{1}H NMR spectrum (400 MHz, MeOD-d_4) of worked up reaction mixture 8.2.2 containing 4-bromoanilinium chloride (**9**). Circles above signals identify the products.

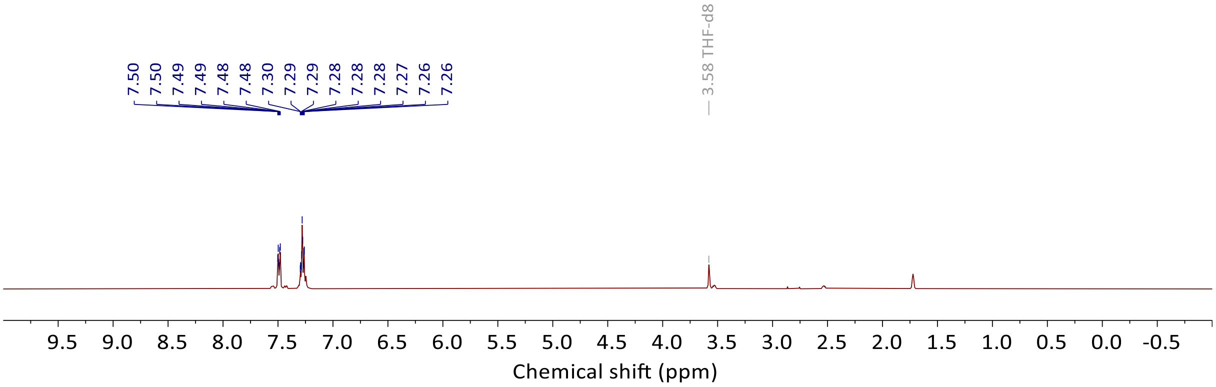

Supplementary Figure 71: ^{1}H NMR spectrum (400MHz, THF-d_8) of isolated Sn_2Ph_6 (**7**).

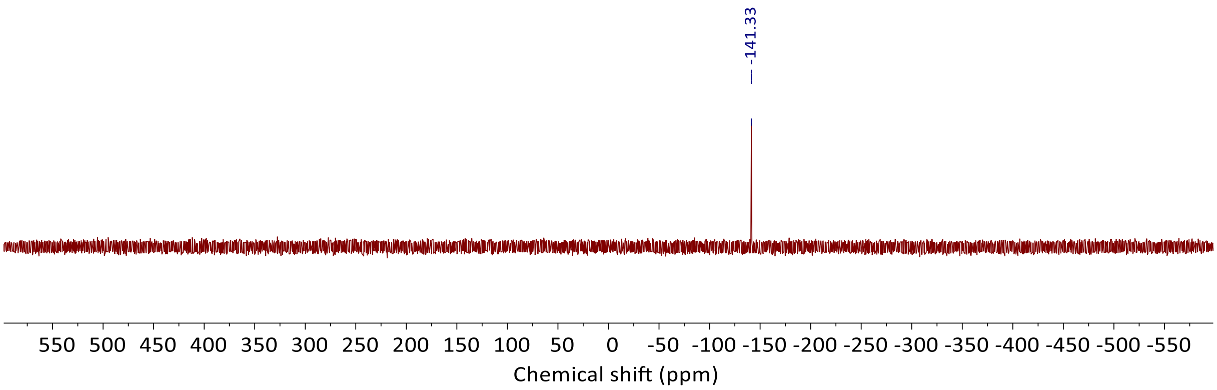

Supplementary Figure 72: ^{119}Sn NMR spectrum (149 MHz, C_6D_6) of isolated Sn_2Ph_6 (**7**).

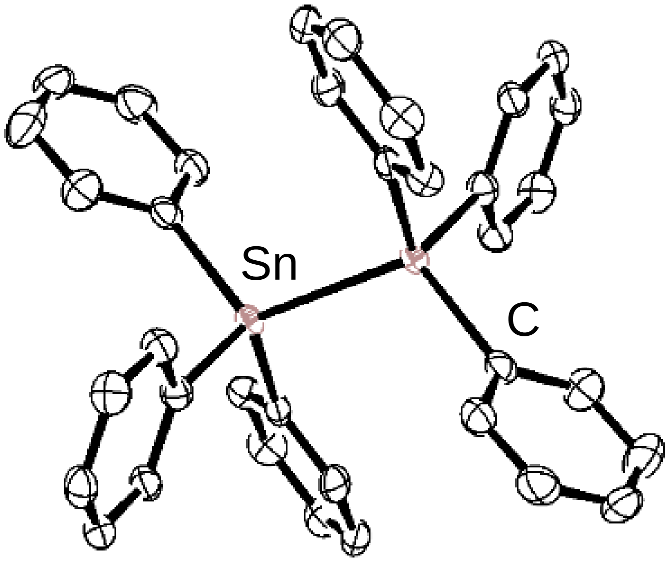

Supplementary Figure 73: Molecular structure of Sn_2Ph_6 (**7**) obtained from reaction 3.2.2 showing anisotropic displacement ellipsoids at 50% probability with hydrogen atoms omitted for clarity. Tin: pink; carbon: white.

8.3. Addition of TolSH to [K(crypt)][1]

8.3.1. Addition of 1 equivalent of TolSH

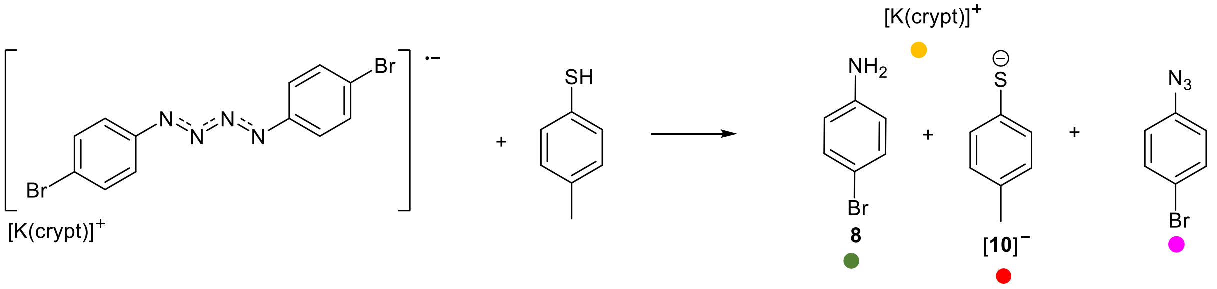

In the glovebox, a solution of TolSH (6.4 mg, 0.026 mmol, 1 equiv.) in THF-d_8 (0.5 mL) was added to a vial containing [K(crypt)][**1**] (20 mg, 0.026 mmol, 1 equiv.). The solution was shaken for 1 minute and filtered into a J Young NMR tube and analyzed by NMR spectroscopy. Single crystals from the reaction were obtained via slow diffusion of hexane into the reaction mixture. XRD studies confirmed these crystals to be of **8** + [K(crypt)][**10**]. EPR spectroscopy was conducted on the reaction mixture and no resonances were observed, confirming the presence of only diamagnetic products. The presence of 4-$BrC_6H_4N_3$ was confirmed by comparing NMR data from the reaction mixture with that independently acquired for 4-$BrC_6H_4N_3$.

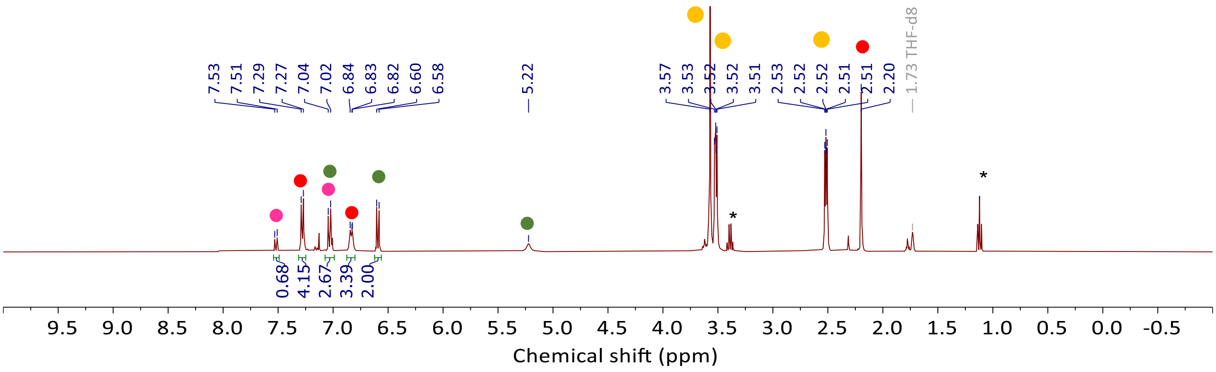

Supplementary Figure 74: ^{1}H NMR spectrum (400 MHz, THF-d_8) of crude reaction mixture of [K(crypt)][**1**] + TolSH 1:1. Circles above signals identify the products and diethyl ether identified with *.

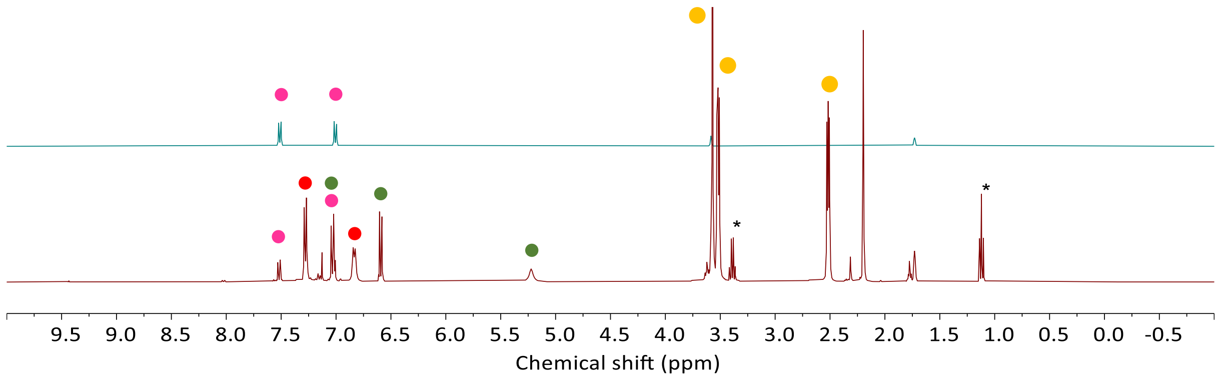

Supplementary Figure 75: Stacked ^{1}H NMR spectra (400 MHz, THF-d_8) of 4-$BrC_6H_4N_3$ (top) and the reaction mixture 8.3.1 (bottom).

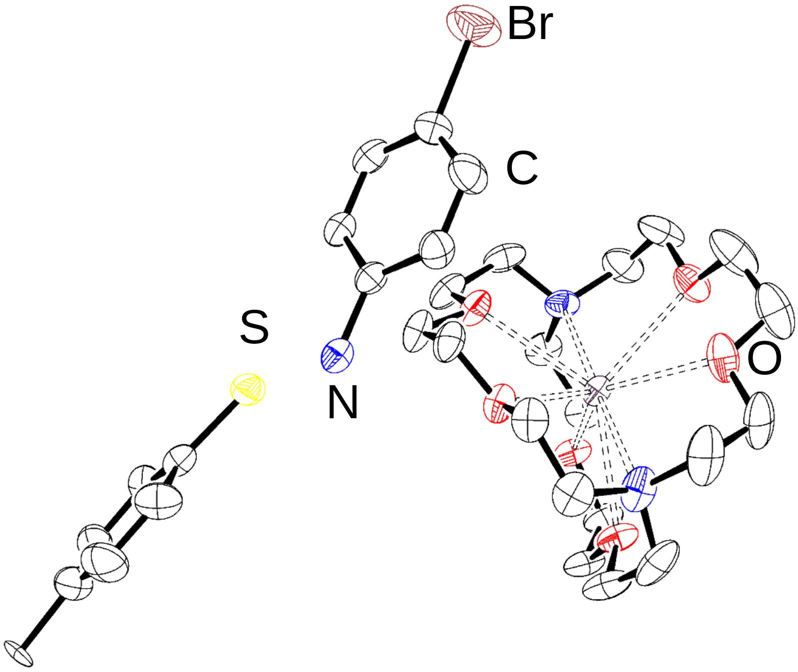

Supplementary Figure 76: Molecular structure of **8** + [K(crypt)][**10**] showing anisotropic displacement ellipsoids at 50% probability with hydrogen atoms omitted for clarity. Nitrogen: blue; carbon: white; bromine: brown; sulfur: yellow; oxygen: red; potassium: violet.

8.3.2 Addition of 4 equivalents of TolSH

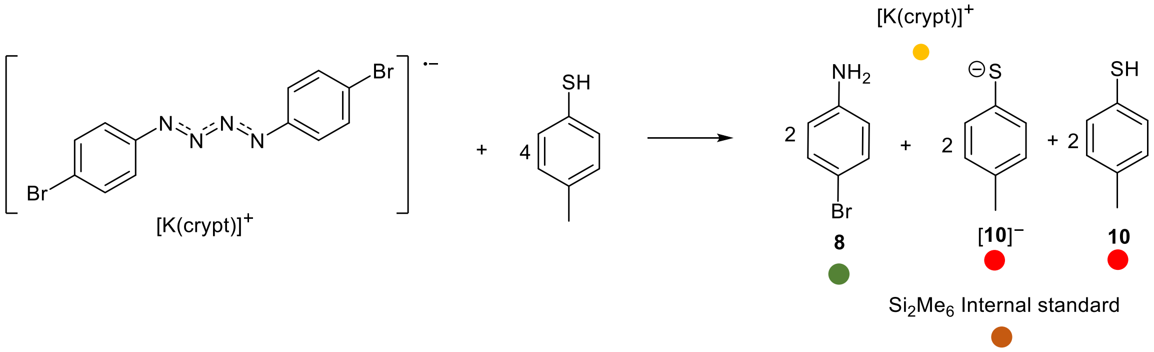

In the glovebox, a solution of TolSH (12.7 mg, 0.102 mmol, 4 equiv.) in THF (1 mL) was added to a vial containing [K(crypt)][**1**] (18.8 mg, 0.023 mmol, 1 equiv.). The solution was shaken for 1 minute before removing the solvent under vacuum. The remaining solid was washed with diethyl ether and dried under vacuum before analyzing by NMR spectroscopy in THF-d_8 (0.5 mL). Single crystals from the reaction were obtained via slow diffusion of hexane into the reaction mixture and **8** + [K(crypt)][**10**] was again observed by XRD studies. EPR spectroscopy was conducted on the reaction mixture and no resonances were observed, confirming the presence of only diamagnetic products.

NMR conversion: 64% (amine integration vs. Si_2Me_6 internal standard)

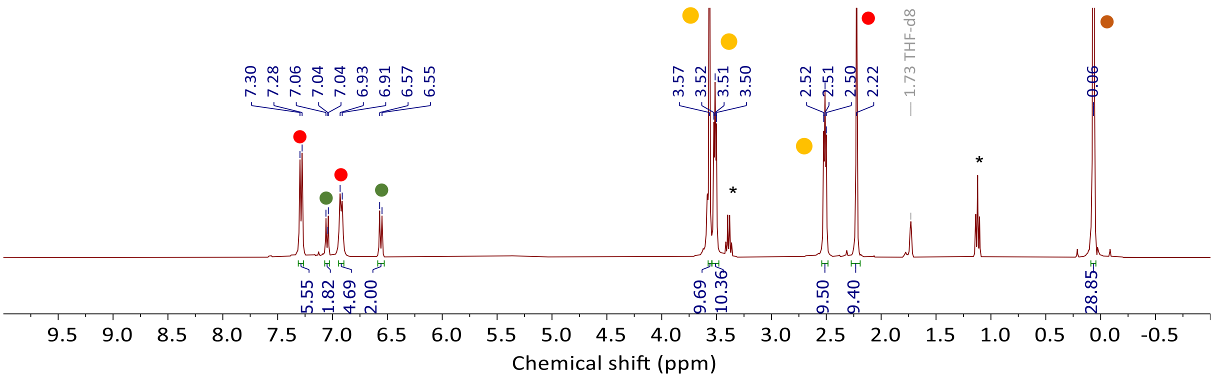

Supplementary Figure 77: ^{1}H NMR spectrum (400 MHz, THF-d_8) of **8** + [K(crypt)][**10**] after work-up. Circles above signals identify the products and diethyl ether identified with *.

8.3.3 Independent preparation of 1:2 mixture of 8 + [K(crypt)][10].

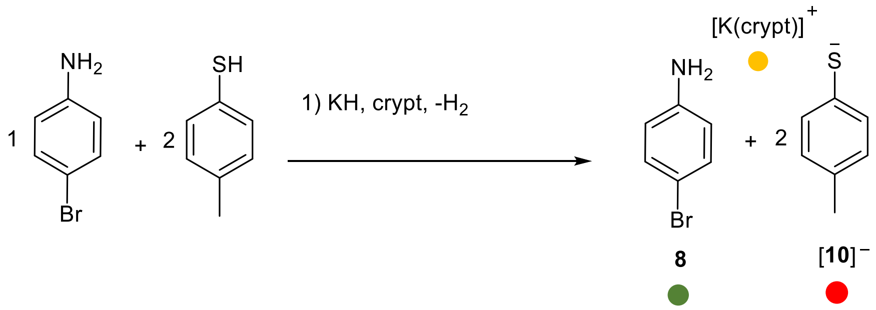

In order to confirm the presence of **8** + [K(crypt)][**10**] in the reactions with TolSH, a mixture of **8** + K(crypt)][**10**] was independently prepared and used to compare reaction mixture NMR data. In a vial, 4-bromoaniline (20.0 mg, 0.012 mmol, 1 equiv.), 2.2.2-cryptand (43.6 mg, 0.012 mmol, 1 eqv.) and TolSH (28.8 mg, 0.024 mmol, 2 equiv.) were dissolved in THF-d_8 (0.5mL). Potassium hydride (4.7 mg, 0.012 mmol, 1 equiv.) was added and shaken for 1 minute with gas evolution being observed. The mixture was then filtered into a J Young NMR tube for analysis. Broadening of the aromatic signals and changing of their chemical shift depends on the ratio **8**:[**10**]$^-$ is consistent with a proton on the thiol shuttling between the sulfur and the nitrogen of the amide. This is consistent with XRD data for **8** + [K(crypt)][**10**], where the potassium is found between the two compounds and the nitrogen and sulfur atoms point towards one another.

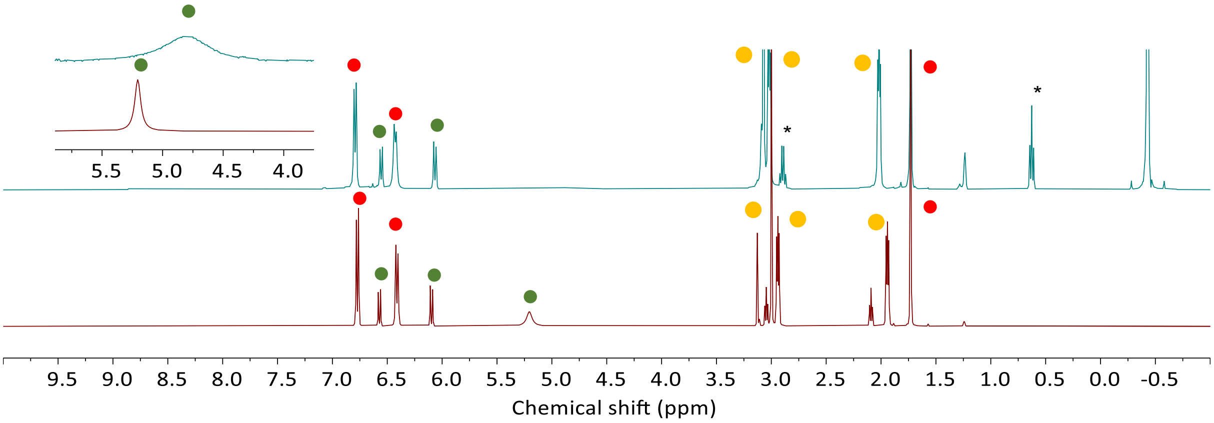

Supplementary Figure 78: Stacked ^{1}H NMR spectra (400 MHz, THF-d_8) of reaction mixture 8.3.2 (top) and independently prepared 1:2 mixture **8** + [K(crypt)][**10**] (bottom) with an inset showing the labile proton. Circles above signals identify the products and diethyl ether identified with *.

8.4 Reaction with 4-IC_6H_4CHO

8.4.1. Reaction in THF-d_8 and crude NMR spectra

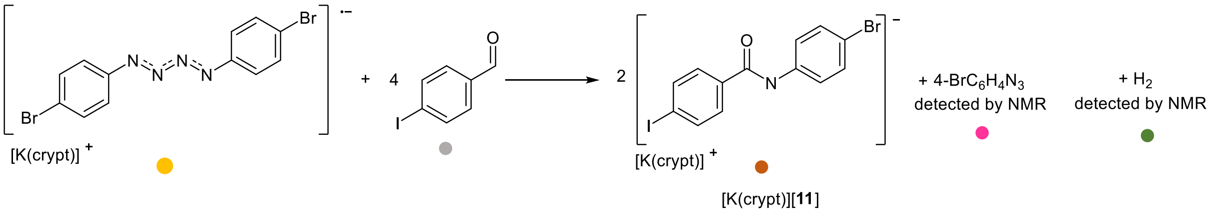

In a glovebox, [K(crypt)][**1**] (60 mg, 0.0765 mmol, 1 equiv.) and 4-iodobenzaldehyde (72 mg, 0.306 mmol, 4 equiv.) were dissolved in THF-d_8 and a crude NMR taken immediately to identify the fate of the remaining atoms in [K(crypt)][**1**] that were not observed in **11**. 4-$BrC_6H_4N_3$ and H_2 gas could be observed in the crude reaction mixture NMR spectrum.[42] McDonald has reported that in the gas phase reactivity of $[PhN]^{\bullet-}$ with aldehydes, the products formed are $[PhNC(O)R]^-$ (the deprotonated amide) and $H^\bullet$,[43] and $H^\bullet$ radicals are known to lead to the formation of H_2 gas.[44, 45]

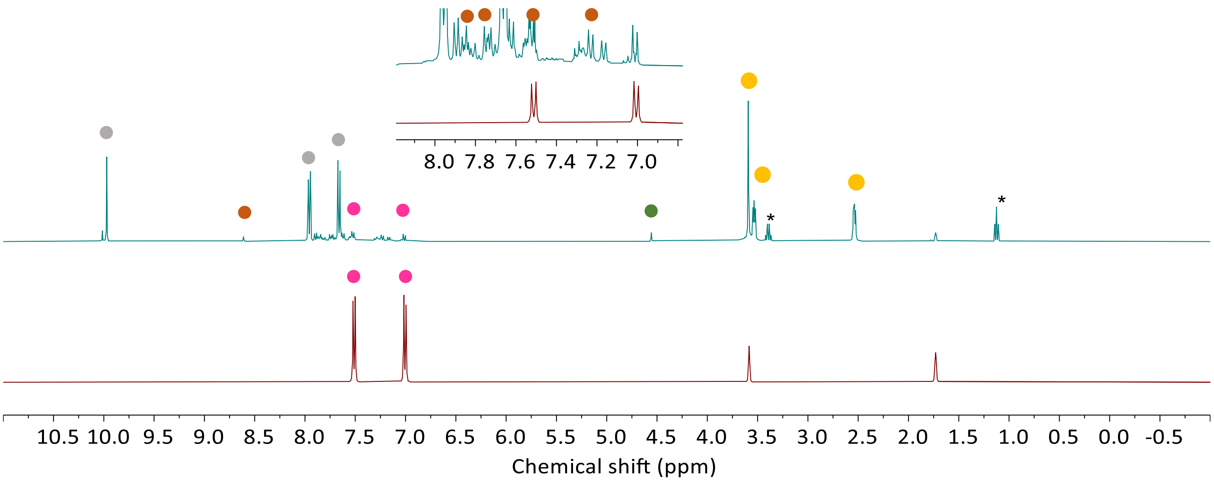

Supplementary Figure 79: ^1H NMR spectra (400 MHz, THF-d_8) of the crude reaction mixture between [K(crypt)][**1**] and 4 equivalents of 4-iodobenzaldehyde (top) stacked with 4-$BrC_6H_4N_3$ (bottom) with an inset zoomed into the aromatic region. Circles above signals identify the products and diethyl ether identified with *.

8.4.2. Reaction in *o*DFB and isolation of 11

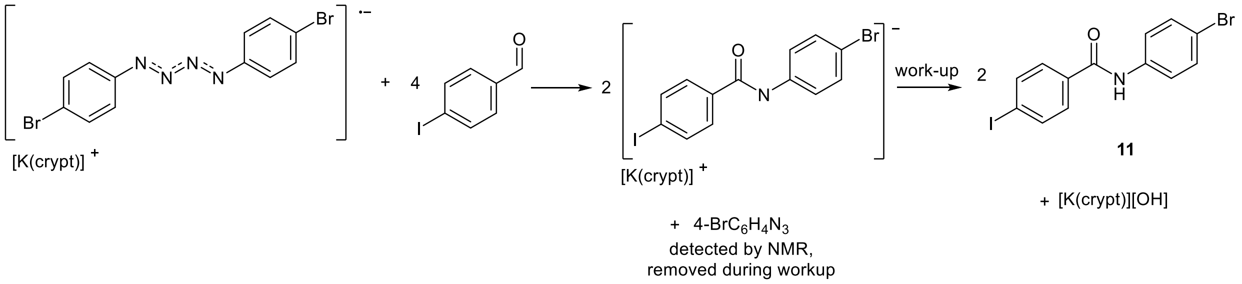

In a glovebox, [K(crypt)][**1**] (60 mg, 0.0765 mmol, 1 equiv.) and 4-iodobenzaldehyde (72 mg, 0.306 mmol, 4 equiv.) were dissolved in *o*DFB (2 mL) and stirred for 2 hours. The solvent was removed in vacuo yielding a dark red oil which was extracted with diethyl ether (wet) and passed through a silica plug using chloroform. The solvent was then removed and excess 4-iodobenzaldehyde removed by vacuum distillation at 45 °C yielding compound **11**.

Isolated Yield: 20.2 mg (66%)

Mass Spectrometry: $[M-H]^-$ Found 399.8843 Calculated 399.8839

^{1}H NMR (400 MHz, 298 K, CDCl$_3$): δ = 8.36 (s, 1H, N*H*), 7.83 (d, 2H, $^3J_{H-H}$ = 8.4 Hz, Ar), 7.62 (d, 2H, $^3J_{H-H}$ = 8.4 Hz, Ar), 7.51 (d, 2H, $^3J_{H-H}$ = 8.7 Hz, Ar), 7.09 (d, 2H, $^3J_{H-H}$ = 8.7f Hz, Ar) ppm.

^{13}C{^{1}H} NMR (101 MHz, 298 K, CDCl$_3$): δ = 159.53 (s, *C*=O), δ = 138.10 (s, Ar), δ = 137.72 (s, Ar), δ = 135.40 (s, Ar), δ = 132.28 (s, Ar), δ = 130.24 (s, Ar), δ = 129.25 (s, Ar), δ = 122.57 (s, Ar), δ = 98.59 (s, Ar) ppm.

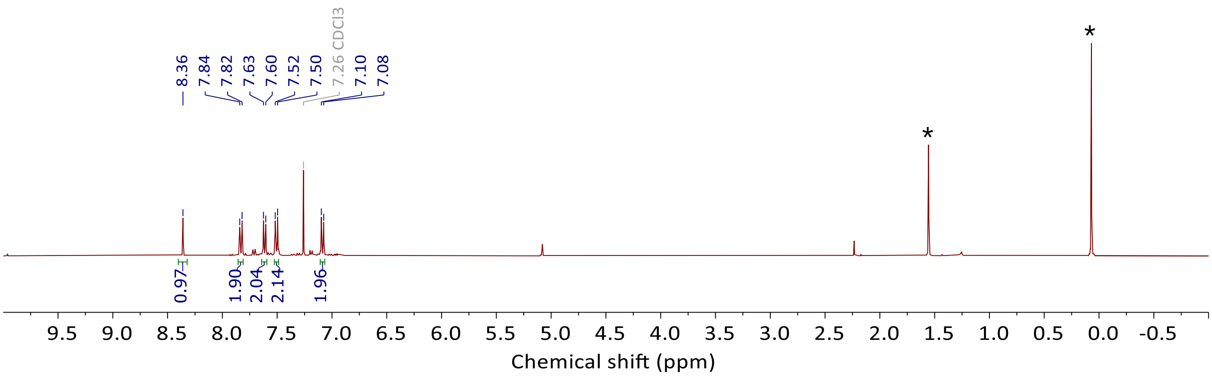

Supplementary Figure 80: ^{1}H NMR spectrum (400MHz, CDCl$_3$) of compound **11** (* indicates water and TMS in bench stored CDCl$_3$).

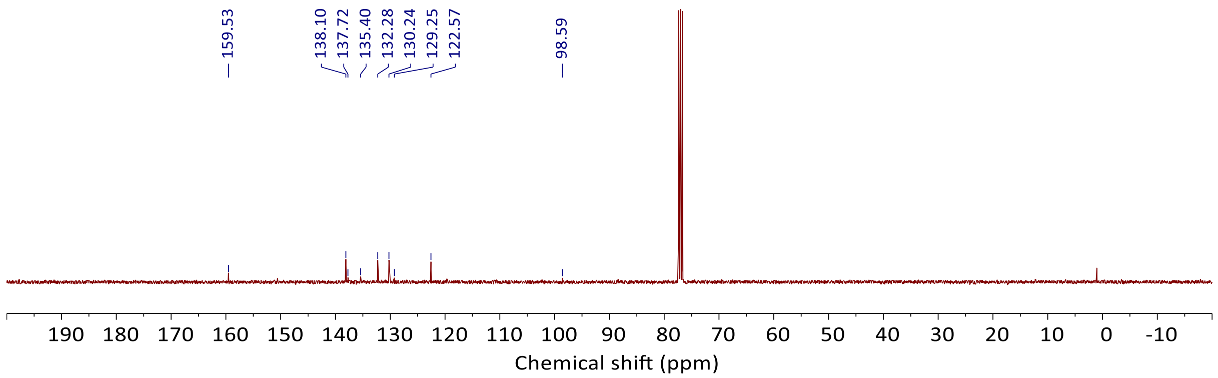

Supplementary Figure 81: $^{13}C\{1H\}$ NMR spectrum (101 MHz, $CDCl_3$) of compound **11**.

8.4.3. Control reaction of 4-BrPhNHK with 4-IC_6H_4CHO

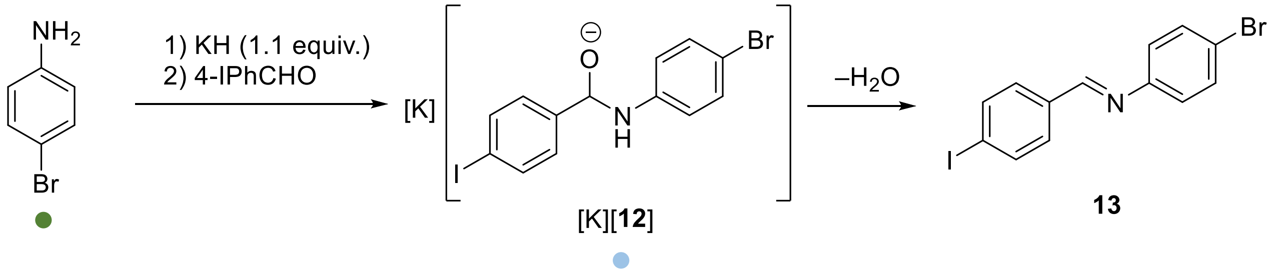

In a glovebox, 4-bromoaniline (15.2mg, 0.125 mmol, 1 equiv.) and potassium hydride (5.5mg, 0.138, 1.1 equiv.) were stirred in THF for 30 minutes. The reaction mixture was filtered and 4-IC_6H_4CHO (29 mg, 0.125 mmol, 1 equiv.) was added. The reaction mixture was filtered into a J Young NMR tube and analyzed by NMR spectroscopy yielding compound [**12**]$^{-}$. Mass spectrometry was conducted on [**12**]$^{-}$ with the major peak being of **13**, the expected product of the dehydration of [**12**]$^{-}$. Water was added to [**12**]$^{-}$, filtered and dried in vacuo. The resultant yellow solid was analyzed by NMR spectroscopy and the product was determined to be **13**. It is worth noting that intermediates related to [**12**]$^{-}$ have been previously reported when aldehydes are converted to imines.[46, 47]

Isolated Yield (13): 75% (36.1 mg)

NMR data of compound [**12**]$^{-}$:

^{1}H NMR (400 MHz, 298 K, THF): δ= 8.44 (s, 1H, *H*CON), 7.82 (d, 2H, $^{2}J_{H-H}$ = 8.2 Hz Ar), 7.64 (d, 2H, $^{2}J_{H-H}$ = 8.2 Hz Ar), 7.48 (d, 2H, $^{2}J_{H-H}$ = 8.8 Hz Ar), 7.12 (d, 2H, $^{2}J_{H-H}$ = 8.8 Hz Ar), 4.71 (s, 1H, N*H*) ppm.

NMR data of compound **13**:

^{1}H NMR (400 MHz, 298 K, $CDCl_3$): δ= 8.36 (s, 1H, *H*CN), 7.83 (d, 2H, $^{2}J_{H-H}$ = 8.2 Hz Ar), 7.62 (d, 2H, $^{2}J_{H-H}$ = 8.2 Hz Ar), 7.51 (d, 2H, $^{2}J_{H-H}$ = 8.8 Hz Ar), 7.09 (d, 2H, $^{2}J_{H-H}$ = 8.8 Hz Ar) ppm.

Mass Spectrometry: [**13**+H]$^{+}$ Found 385.9035 Calculated 385.9036

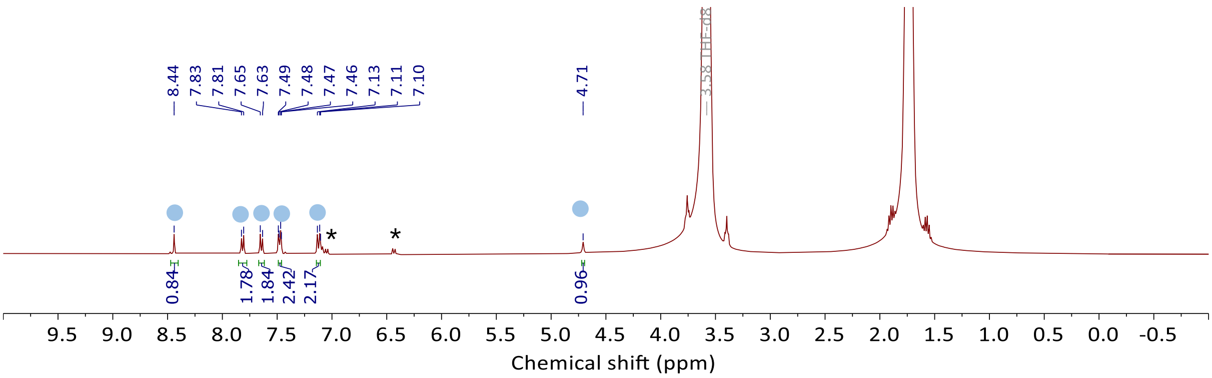

Supplementary Figure 82: ^{1}H NMR spectrum (400MHz, THF) of compound **12** with unreacted 4-BrPhNHK marked with *.

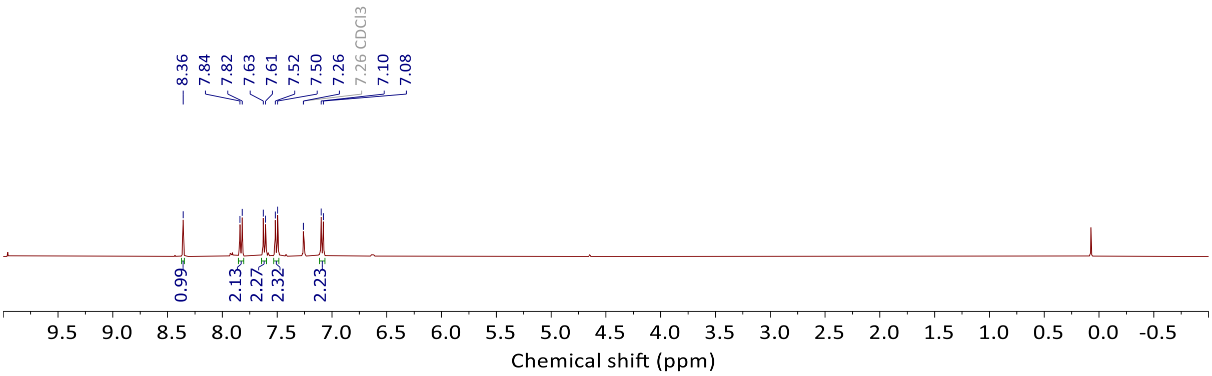

Supplementary Figure 83: ^{1}H NMR spectrum (400MHz, $CDCl_3$) of isolated compound **13.**

8.5 Calculated Energy of Azide Loss

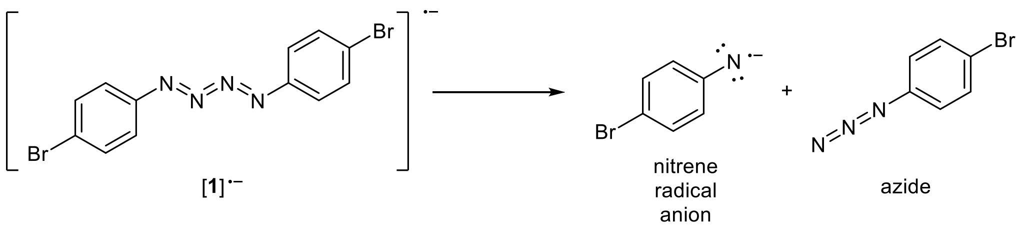

Supplementary Table 40: Calculated electronic and Gibbs energies for the production of a radical anion and azide from [**1**]$^{\bullet -}$ at TPSS/def2-TZVP level of theory.

Solvent	ΔE (kcal/mol)	ΔG (kcal/mol)
THF	36.7	19.2
Cyclopentanone	36.3	19.3

9. Crystallography Tables

**Identification code**	[K(crypt)][**1**]	[K(THF$_2$)]$_2$[**2**]
Empirical formula	$C_{30}H_{44}Br_2KN_6O_6$	$C_{14}H_{20}BrKN_2O_2$
Formula weight	783.626	367.33
Temperature/K	100.00(10)	100.15
Crystal system	monoclinic	monoclinic
Space group	C2/c	$P2_1/c$
a/Å	25.6607(6)	5.6855(2)
b/Å	8.1860(2)	14.6607(4)
c/Å	20.2572(4)	19.6744(5)
α/°	90	90
β/°	98.331(2)	95.860(3)
γ/°	90	90
Volume/Å^3	4210.29(17)	1631.36(8)
Z	4	4
ρ_{calc}g/cm^3	1.236	1.496
μ/mm^{-1}	3.670	5.744
F(000)	1612.9	752.0
Crystal size/mm^3	0.38 × 0.13 × 0.07	0.2 × 0.15 × 0.05
Radiation	Cu Kα (λ = 1.54184)	CuKα (λ = 1.54184)
2Θ range for data collection/°	6.96 to 152.1	7.534 to 151.968
Index ranges	-31 ≤ h ≤ 32, -8 ≤ k ≤ 10, -25 ≤ l ≤ 24	-7 ≤ h ≤ 6, -17 ≤ k ≤ 18, -22 ≤ l ≤ 24
Reflections collected	21319	14805
Independent reflections	4306 [R_{int} = 0.0306, R_{sigma} = 0.0244]	3337 [R_{int} = 0.0323, R_{sigma} = 0.0247]
Data/restraints/parameters	4306/0/229	3337/1266/398
Goodness-of-fit on F^2	1.029	1.064
Final R indexes [I>=2σ (I)]	R_1 = 0.0576, wR_2 = 0.1607	R_1 = 0.0408, wR_2 = 0.1169
Final R indexes [all data]	R_1 = 0.0644, wR_2 = 0.1646	R_1 = 0.0533, wR_2 = 0.1236
Largest diff. peak/hole / e Å^{-3}	0.47/-0.49	0.30/-0.42
CCDC	2423978	2423981

Identification code	[K(crypt)][**3**]	[K(THF$_2$)]$_2$[**4**]
Empirical formula	$C_{38}H_{60}F_2KN_6O_8$	$C_{30}H_{44}Cl_2KN_6O_6$
Formula weight	806.02	694.71
Temperature/K	100.15	100.15
Crystal system	monoclinic	monoclinic
Space group	I2/a	C2/c
a/Å	20.3742(3)	25.4601(4)
b/Å	7.86820(10)	8.17710(10)
c/Å	26.1165(4)	20.3593(3)
α/°	90	90
β/°	102.6360(10)	99.218(2)
γ/°	90	90
Volume/Å^3	4085.29(10)	4183.86(11)
Z	4	4
ρ_{calc}g/cm^3	1.310	1.103
μ/mm^{-1}	1.696	2.626
F(000)	1724.0	1468.0
Crystal size/mm^3	0.261 × 0.097 × 0.063	0.16 × 0.13 × 0.1
Radiation	CuKα (λ = 1.54184)	CuKα (λ = 1.54184)
2Θ range for data collection/°	6.938 to 151.55	7.034 to 151.87
Index ranges	-25 ≤ h ≤ 22, -9 ≤ k ≤ 9, -32 ≤ l ≤ 31	-31 ≤ h ≤ 31, -8 ≤ k ≤ 9, -25 ≤ l ≤ 25
Reflections collected	41231	41300
Independent reflections	4202 [R_{int} = 0.0386, R_{sigma} = 0.0195]	4269 [R_{int} = 0.0401, R_{sigma} = 0.0241]
Data/restraints/parameters	4202/62/289	4269/0/204
Goodness-of-fit on F^2	1.069	1.048
Final R indexes [I>=2σ (I)]	R_1 = 0.0808, wR_2 = 0.2295	R_1 = 0.0437, wR_2 = 0.1264
Final R indexes [all data]	R_1 = 0.0887, wR_2 = 0.2383	R_1 = 0.0536, wR_2 = 0.1324
Largest diff. peak/hole / e Å^{-3}	0.38/-0.57	0.43/-0.79
CCDC	2481373	2481374

**Identification code**	[K(crypt)][**5**]	**7**
Empirical formula	$C_{32}H_{50}KN_6O_6$	$C_{36}H_{30}Sn_2$
Formula weight	653.88	699.98
Temperature/K	150.15	150.00(10)
Crystal system	monoclinic	monoclinic
Space group	C2/c	$P2_1/n$
a/Å	25.8121(10)	17.0537(3)
b/Å	8.1362(2)	9.27280(10)
c/Å	20.5032(6)	20.2706(3)
α/°	90	90
β/°	99.099(3)	111.898(2)
γ/°	90	90
Volume/Å^3	4251.7(2)	2974.22(8)
Z	4	4
ρ_{calc}g/cm^3	1.022	1.563
μ/mm^{-1}	1.427	13.510
F(000)	1404.0	1384.0
Crystal size/mm^3	0.39 × 0.15 × 0.15	0.266 × 0.207 × 0.121
Radiation	CuKα (λ = 1.54184)	Cu Kα (λ = 1.54184)
2Θ range for data collection/°	6.936 to 152.572	8.54 to 152.302
Index ranges	-31 ≤ h ≤ 32, -9 ≤ k ≤ 10, -25 ≤ l ≤ 22	-21 ≤ h ≤ 21, -10 ≤ k ≤ 11, -25 ≤ l ≤ 21
Reflections collected	18940	15884
Independent reflections	4387 [R_{int} = 0.0466, R_{sigma} = 0.0333]	6166 [R_{int} = 0.0327, R_{sigma} = 0.0322]
Data/restraints/parameters	4387/0/205	6166/0/343
Goodness-of-fit on F^2	1.007	1.035
Final R indexes [I>=2σ (I)]	R_1 = 0.0464, wR_2 = 0.1307	R_1 = 0.0370, wR_2 = 0.0984
Final R indexes [all data]	R_1 = 0.0601, wR_2 = 0.1419	R_1 = 0.0390, wR_2 = 0.1003
Largest diff. peak/hole / e Å^{-3}	0.25/-0.25	3.48/-1.35
CCDC	2481375	2423980

Justification for B alert in **7**.

PLAT971_ALERT_2_B Check Calcd Resid. Dens. 1.70Ang From Sn02 3.33 eA-3

Author Response: Residual electron density is observed around the heavy Sn atom.

**Identification code**	**8** + [K(crypt)][**10**]	**8** + [K(crypt)][**10**] NoSpherA2
Empirical formula	$C_{31}H_{49}BrKN_3O_6S$	$C_{31}H_{49}BrKN_3O_6S$
Formula weight	710.80	710.819
Temperature/K	150.00(10)	150.00(10)
Crystal system	orthorhombic	orthorhombic
Space group	Pbca	Pbca
a/Å	23.3381(2)	23.3381(2)
b/Å	27.1976(3)	27.1976(3)
c/Å	11.35040(10)	11.3504(1)
α/°	90	90
β/°	90	90
γ/°	90	90
Volume/Å^3	7204.56(12)	7204.56(12)
Z	8	8
ρ_{calc}g/cm^3	1.311	1.311
μ/mm^{-1}	3.479	3.483
F(000)	2992.0	3001.2
Crystal size/mm^3	0.328 × 0.277 × 0.105	0.328 × 0.277 × 0.105
Radiation	Cu Kα (λ = 1.54184)	Cu Kα (λ = 1.54184)
2Θ range for data collection/°	7.576 to 152.642	7.58 to 134.14
Index ranges	-29 ≤ h ≤ 29, -34 ≤ k ≤ 31, -14 ≤ l ≤ 14	-29 ≤ h ≤ 29, -34 ≤ k ≤ 31, -14 ≤ l ≤ 14
Reflections collected	154596	154596
Independent reflections	7536 [R_{int} = 0.0604, R_{sigma} = 0.0150]	6420 [R_{int} = 0.0604, R_{sigma} = 0.0150]
Data/restraints/parameters	7536/0/389	6420/89/397
Goodness-of-fit on F^2	1.161	1.029
Final R indexes [I>=2σ (I)]	R_1 = 0.0871, wR_2 = 0.2171	R_1 = 0.0893, wR_2 = 0.2217
Final R indexes [all data]	R_1 = 0.0884, wR_2 = 0.2177	R_1 = 0.0902, wR_2 = 0.2221
Largest diff. peak/hole / e Å^{-3}	1.04/-1.47	1.43/-1.48
CCDC	2423979	NA

10. References

1. Heurich T, Nesterov V, Schnakenburg G, Qu Z-W, Grimme S, Hazin K*, et al.* Strong Evidence of a Phosphanoxyl Complex: Formation, Bonding, and Reactivity of Ligated Phosphorus Analogues of Nitroxides. *Angew. Chem. Int. Ed.* 2016, **55**(46)**:** 14439–14443.

2. Maier TM, Coburger P, van Leest NP, Hey-Hawkins E, Wolf R. Direct Synthesis of an Anionic 13-Vertex Closo-Cobaltacarborane Cluster. *Dalton Trans.* 2019, **48**(42)**:** 15772–15777.

3. Mamidyala SK, Cooper MA. Probing the Reactivity of o-Phthalaldehydic Acid/Methyl Ester: Synthesis of N-Isoindolinones and 3-Arylaminophthalides. *Chem. Commun.* 2013, **49**(75)**:** 8407–8409.

4. Stoll S, Schweiger A. EasySpin, A Comprehensive Software Package for Spectral Simulation and Analysis in EPR. *J. Magn. Reson.* 2006, **178**(1)**:** 42–55.

5. CrysAlis PRO. Agilent Technologies Ltd: Yarnton O, England 2014.

6. Sheldrick G. SHELXT - Integrated Space-Group and Crystal-Structure Determination. *Acta Crystallogr. A* 2015, **71**(1)**:** 3–8.

7. Dolomanov OV, Bourhis LJ, Gildea RJ, Howard JAK, Puschmann H. OLEX2: A Complete Structure Solution, Refinement and Analysis Program. *J. Appl. Cryst.* 2009, **42:** 339–341.

8. Kleemiss F, Dolomanov OV, Bodensteiner M, Peyerimhoff N, Midgley L, Bourhis LJ*, et al.* Accurate Crystal Structures and Chemical Properties from NoSpherA2. *Chem. Sci.* 2021, **12**(5)**:** 1675–1692.

9. Neese F. Software update: The ORCA program system—Version 5.0. *WIREs Comput. Mol. Sci.* 2022, **12**(5)**:** e1606.

10. Furness JW, Kaplan AD, Ning J, Perdew JP, Sun J. Accurate and Numerically Efficient r2SCAN Meta-Generalized Gradient Approximation. *J. Phys. Chem. Lett.* 2020, **11**(19)**:** 8208–8215.

11. Binkley JS, Pople JA, Hehre WJ. Self-Consistent Molecular Orbital Methods. 21. Small Split-Valence Basis Sets for First-Row Elements. *J. Am. Chem. Soc.* 1980, **102**(3)**:** 939–947.

12. Weigend F. Accurate Coulomb-Fitting Basis Sets for H to Rn. *Phys. Chem. Chem. Phys.* 2006, **8**(9)**:** 1057–1065.

13. Kabova EA, Blundell CD, Muryn CA, Whitehead GFS, Vitorica-Yrezabal IJ, Ross MJ*, et al.* SDPD-SX: Combining a Single Crystal X-ray Diffraction Setup with Advanced

Powder Data Structure Determination for Use in Early Stage Drug Discovery. *CrystEngComm* 2022, **24**(24)**:** 4337–4340.

14. Coelho A. An Indexing Algorithm Independent of Peak Position Extraction for X-ray Powder Diffraction Patterns. *J. Appl. Crystallogr.* 2017, **50**(5)**:** 1323–1330.

15. Petříček V, Dušek M, Palatinus L. Crystallographic Computing System JANA2006: General Features. *Z. Kristallogr. Cryst. Mater.* 2014, **229**(5)**:** 345–352.

16. Frisch MJ, Trucks GW, Schlegel HB, Scuseria GE, Robb MA, Cheeseman JR*, et al.* Gaussian 16 Rev. C.01. Wallingford, CT; 2016.

17. Tao J, Perdew JP, Staroverov VN, Scuseria GE. Climbing the Density Functional Ladder: Nonempirical Meta--Generalized Gradient Approximation Designed for Molecules and Solids. *Phys. Rev. Lett.* 2003, **91**(14)**:** 146401.

18. Grimme S. Supramolecular Binding Thermodynamics by Dispersion-Corrected Density Functional Theory. *Chem. Eur. J.* 2012, **18**(32)**:** 9955–9964.

19. Luchini G, Alegre-Requena J, Funes-Ardoiz I, Paton R. GoodVibes: Automated Thermochemistry for Heterogeneous Computational Chemistry Data. *F1000Research* 2020, **9**(291).

20. Weigend F, Ahlrichs R. Balanced Basis Sets of Split Valence, Triple Zeta Valence and Quadruple Zeta Valence Quality for H to Rn: Design and Assessment of Accuracy. *Phys. Chem. Chem. Phys.* 2005, **7**(18)**:** 3297–3305.

21. Marenich AV, Cramer CJ, Truhlar DG. Universal Solvation Model Based on Solute Electron Density and on a Continuum Model of the Solvent Defined by the Bulk Dielectric Constant and Atomic Surface Tensions. *J. Phys. Chem. B* 2009, **113**(18)**:** 6378–6396.

22. NBO 7.0. E. D. Glendening J, K. Badenhoop, A. E. Reed, J. E. Carpenter, J. A. Bohmann, C. M. Morales, P. Karafiloglou, C. R. Landis, and F. Weinhold, Theoretical Chemistry Institute, University of Wisconsin, Madison (2018).

23. Zhao L, Pan S, Holzmann N, Schwerdtfeger P, Frenking G. Chemical Bonding and Bonding Models of Main-Group Compounds. *Chem. Rev.* 2019, **119**(14)**:** 8781–8845.

24. Staroverov VN, Scuseria GE, Tao J, Perdew JP. Comparative Assessment of a New Nonempirical Density Functional: Molecules and Hydrogen-Bonded Complexes. *J. Chem. Phys.* 2003, **119**(23)**:** 12129–12137.

25. Neese F. The ORCA program system. *WIREs Comput. Mol. Sci.* 2012, **2**(1)**:** 73–78.

26. Becke AD. Density-functional thermochemistry. III. The Role of Exact Exchange. *J. Chem. Phys.* 1993, **98**(7)**:** 5648–5652.

27. Lee C, Yang W, Parr RG. Development of the Colle-Salvetti Correlation-Energy Formula into a Functional of the Electron Density. *Phys. Rev. B* 1988, **37**(2)**:** 785–789.

28. Kendall RA, Dunning TH, Jr., Harrison RJ. Electron Affinities of the First-row Atoms Revisited. Systematic Basis Sets and Wave Functions. *J. Chem. Phys.* 1992, **96**(9)**:** 6796–6806.

29. Barone V. Structure, Magnetic Properties and Reactivities of Open-Shell Species From Density Functional and Self-Consistent Hybrid Methods. *Recent Advances in Density Functional Methods*, pp 287–334.

30. Lu T, Chen F. Multiwfn: A Multifunctional Wavefunction Analyzer. *J. Comput. Chem.* 2012, **33**(5)**:** 580–592.

31. Momma K, Izumi F. VESTA: A Three-Dimensional Visualization System for Electronic and Structural Analysis. *Appl. Crystallogr.* 2008, **41**(3)**:** 653–658.

32. Hassan I, Pavlov J, Errabelli R, Attygalle AB. Oxidative Ionization Under Certain Negative-Ion Mass Spectrometric Conditions. *J. Am. Soc. Mass Spectrom.* 2017, **28**(2)**:** 270–277.

33. Perdew JP, Burke K, Ernzerhof M. Generalized Gradient Approximation Made Simple. *Phys. Rev. Lett.* 1996, **77**(18)**:** 3865–3868.

34. Adamo C, Barone V. Toward Reliable Density Functional Methods Without Adjustable Parameters: The PBE0 Model. *J. Chem. Phys.* 1999, **110**(13)**:** 6158–6170.

35. Chai J-D, Head-Gordon M. Long-range Corrected Hybrid Density Functionals with Damped Atom–Atom Dispersion Corrections. *Phys. Chem. Chem. Phys.* 2008, **10**(44)**:** 6615–6620.

36. Gulaczyk I, Kręglewski M, Valentin A. The N–N Stretching Band of Hydrazine. *J. Mol. Spectros.* 2003, **220**(1)**:** 132–136.

37. Fujino T, Tahara T. Picosecond Time-Resolved Raman Study of trans-Azobenzene. *J. Phys. Chem. A* 2000, **104**(18)**:** 4203–4210.

38. Yanai T, Tew DP, Handy NC. A New Hybrid Exchange–Correlation Functional Using the Coulomb-Attenuating Method (CAM-B3LYP). *Chem. Phys. Lett.* 2004, **393**(1)**:** 51–57.

39. Wang Y, Verma P, Jin X, Truhlar DG, He X. Revised M06 Density Functional for Main-Group and Transition-Metal Chemistry. *Proc. Natl. Acad. Sci.* 2018, **115**(41)**:** 10257–10262.

40. Dust JM, Arnold DR. Substituent Effects on Benzyl Radical ESR Hyperfine Coupling Constants. The σ_α• Scale Based Upon Spin Delocalization. *J. Am. Chem. Soc.* 1983, **105**(5)**:** 1221–1227.

41. Dainis Dakternieks, Fong Sheen Kuan, Andrew Duthie, Edward R. T. Tiekink. The Crystal Structure of the Triclinic Polymorph of Hexaphenyldistannane. *Main Group Met. Chem.* 2001, **24**(1)**:** 65–66.

42. Fulmer GR, Miller AJM, Sherden NH, Gottlieb HE, Nudelman A, Stoltz BM*, et al.* NMR Chemical Shifts of Trace Impurities: Common Laboratory Solvents, Organics, and Gases in Deuterated Solvents Relevant to the Organometallic Chemist. *Organometallics* 2010, **29**(9)**:** 2176–2179.

43. McDonald RN, Chowdhury AK. Hypovalent Radicals. 13. Gas-Phase Nucleophilic Reactivities of Phenylnitrene (PhN- •) and Sulfur Anion Radicals (S-•) at sp3 and Carbonyl Carbon. *J. Am. Chem. Soc.* 1983, **105**(2)**:** 198–207.

44. Dixon-Lewis G, Sutton MM, Williams A. The Kinetics of Hydrogen Atom Recombination. *Disc. Faraday Soc.* 1962, **33**(0)**:** 205–212.

45. Wang S, Dames EE, Davidson DF, Hanson RK. Reaction Rate Constant of CH2O + H = HCO + H2 Revisited: A Combined Study of Direct Shock Tube Measurement and Transition State Theory Calculation. *J. Phys. Chem. A* 2014, **118**(44)**:** 10201–10209.

46. Ding Y-Q, Cui Y-Z, Li T-D. New Views on the Reaction of Primary Amine and Aldehyde from DFT Study. *J. Phys. Chem. A* 2015, **119**(18)**:** 4252–4260.

47. Kwiecień A, Ciunik Z. Stable Hemiaminals: 2-Aminopyrimidine Derivatives. *Molecules*; 2015. pp. 14365–14376.
