# Supplementary material for: Crystalline nitrogen chain radical anions
Source: Nat Chem. 2026 Feb 10;18(4):686–94. doi: 10.1038/s41557-025-02040-2 (PMC13061614; doi:10.1038/s41557-025-02040-2)
Supplement: Supplementary file 2 — Raw data associated with [K(crypt)][1]. [file 41557_2025_2040_MOESM2_ESM.zip › Supplementary_Data_1/Folder 5 XRD/Cif_check.pdf]

checkCIF (basic structural check) running

Checking for embedded fcf data in CIF ...
No extractable fcf data in found in CIF

checkCIF/PLATON (basic structural check)

Structure factors have been supplied for datablock(s) 025_r_rlr018

THIS REPORT IS FOR GUIDANCE ONLY. IF USED AS PART OF A REVIEW PROCEDURE FOR PUBLICATION, IT SHOULD NOT REPLACE THE EXPERTISE OF AN EXPERIENCED CRYSTALLOGRAPHIC REFEREE.

No syntax errors found. CIF dictionary
Please wait while processing .... Interpreting this report

Structure factor report

Datablock: 025_r_rlr018

Bond precision:	C-C = 0.0054 A		Wavelength=1.54184
Cell:	a=25.6607(6)	b=8.1860(2)	c=20.2572(4)
	alpha=90	beta=98.331(2)	gamma=90
Temperature:	100 K		

	Calculated	Reported
Volume	4210.29(17)	4210.29(17)
Space group	C 2/c	C 1 2/c 1
Hall group	-C 2yc	-C 2yc
Moiety formula	C18 H36 K N2 O6, C12 H8 Br2 N4 [+ solvent]	C18 H36 K N2 O6, C12 H8 Br2 N4
Sum formula	C30 H44 Br2 K N6 O6 [+ solvent]	C30 H44 Br2 K1 N6 O6
Mr	783.61	783.63
Dx,g cm-3	1.236	1.236
Z	4	4
Mu (mm-1)	3.666	3.670
F000	1612.0	1612.9
F000'	1612.02	
h,k,lmax	32,10,25	32,10,25
Nref	4408	4306
Tmin,Tmax	0.592,0.773	0.677,1.000
Tmin'	0.236	

Correction method= # Reported T Limits: Tmin=0.677 Tmax=1.000
AbsCorr = MULTI-SCAN

Data completeness= 0.977 Theta(max)= 76.050

R(reflections)= 0.0576(3694) wR2(reflections)= 0.1646(4306)

S = 1.029 Npar= 229

The following ALERTS were generated. Each ALERT has the format
test-name_ALERT_alert-type_alert-level.
Click on the hyperlinks for more details of the test.

Alert level C

```
PLAT242_ALERT_2_C Low    'MainMol' Ueq as Compared to Neighbors of        C4 Check
PLAT906_ALERT_3_C Large K Value in the Analysis of Variance ......     3.259 Check
PLAT911_ALERT_3_C Missing FCF Refl Between Thmin & STh/L=    0.600        10 Report
                1  1  2,  21  5  2, -12  4  3, -10  8  3, -10  8  4,   2  0  4,
               26  0  4,  -2  0  8, -19  5 11, -12  0 20,
PLAT918_ALERT_3_C Reflection(s) with I(obs) much Smaller I(calc) .         1 Check
```

```
PLAT934_ALERT_3_C Number of (Iobs-Icalc)/Sigma(W) > 10 Outliers ..          1 Check
               -2   2   3,
PLAT975_ALERT_2_C Check Calcd Resid. Dens.  1.01Ang From O1      .       0.48 eA-3
```

Alert level G

```
PLAT041_ALERT_1_G Calc. and Reported SumFormula    Strings  Differ     Please Check
              Calc: C30 H44 Br2 K N6 O6
              Rep.: C30 H44 Br2 K1 N6 O6
PLAT073_ALERT_1_G H-atoms ref., but hydrogen treatment Reported as     constr Check
PLAT083_ALERT_2_G SHELXL Second Parameter in WGHT  Unusually Large      13.67 Why ?
PLAT605_ALERT_4_G Largest Solvent Accessible VOID in the Structure        253 A**3
PLAT769_ALERT_4_G CIF Embedded Explicitly Supplied Scattering Data     Please Note
PLAT868_ALERT_4_G ALERTS Due to the Use of _smtbx_masks Suppressed          ! Info
PLAT872_ALERT_4_G ALERTS Related to Anharmonic Refine   Suppressed          ! Info
PLAT912_ALERT_4_G Missing # of FCF Reflections Above STh/L=  0.600         92 Note
PLAT933_ALERT_2_G Number of HKL-OMIT Records in Embedded .res File          2 Note
               -2   0   8,    1   1   2,
PLAT969_ALERT_5_G The 'Henn et al.' R-Factor-gap value ..........       6.089 Note
              Predicted wR2: Based on SigI**2  2.70 or SHELX Weight 15.99
PLAT978_ALERT_2_G Number C-C Bonds with Positive Residual Density.          0 Info
PLAT982_ALERT_1_G The Br-f'=    -0.6696 Deviates from  IT-Value =     -0.6763 Check
PLAT983_ALERT_1_G The Br-f"=     1.2830 Deviates from  IT-Value =      1.2805 Check
PLAT983_ALERT_1_G The  K-f"=     1.0675 Deviates from  IT-Value =      1.0657 Check
```

```
  0 ALERT level A = Most likely a serious problem - resolve or explain
  0 ALERT level B = A potentially serious problem, consider carefully
  6 ALERT level C = Check. Ensure it is not caused by an omission or oversight
 14 ALERT level G = General information/check it is not something unexpected

  5 ALERT type 1 CIF construction/syntax error, inconsistent or missing data
  5 ALERT type 2 Indicator that the structure model may be wrong or deficient
  4 ALERT type 3 Indicator that the structure quality may be low
  5 ALERT type 4 Improvement, methodology, query or suggestion
  1 ALERT type 5 Informative message, check
```

It is advisable to attempt to resolve as many as possible of the alerts in all categories. Often the minor alerts point to easily fixed oversights, errors and omissions in your CIF or refinement strategy, so attention to these fine details can be worthwhile. In order to resolve some of the more serious problems it may be necessary to carry out additional measurements or structure refinements. However, the purpose of your study may justify the reported deviations and the more serious of these should normally be commented upon in the discussion or experimental section of a paper or in the "special_details" fields of the CIF. checkCIF was carefully designed to identify outliers and unusual parameters, but every test has its limitations and alerts that are not important in a particular case may appear. Conversely, the absence of alerts does not guarantee there are no aspects of the results needing attention. It is up to the individual to critically assess their own results and, if necessary, seek expert advice.

Publication of your CIF in IUCr journals

A basic structural check has been run on your CIF. These basic checks will be run on all CIFs submitted for publication in IUCr journals (*Acta Crystallographica*, *Journal of Applied Crystallography*, *Journal of Synchrotron Radiation*); however, if you intend to submit to *Acta Crystallographica Section C* or *E* or *IUCrData*, you should make sure that full publication checks are run on the final version of your CIF prior to submission.

Publication of your CIF in other journals

Please refer to the *Notes for Authors* of the relevant journal for any special instructions relating to CIF submission.

PLATON version of 02/02/2025; check.def file version of 02/02/2025

Datablock 025_r_rlr018 - ellipsoid plot

Download CIF editor (publCIF) from the IUCr
Download CIF editor (enCIFer) from the CCDC
Test a new CIF entry
