# Supplementary material for: Crystalline nitrogen chain radical anions
Source: Nat Chem. 2026 Feb 10;18(4):686–94. doi: 10.1038/s41557-025-02040-2 (PMC13061614; doi:10.1038/s41557-025-02040-2)
Supplement: Supplementary file 3 — Raw data associated with [K(crypt)][3]. [file 41557_2025_2040_MOESM3_ESM.zip › Supplementary_Data_2/Description of Additional Supplementary Files.docx]

**Description of Additional Supplementary Files**

**Supplementary Data Folder 1:** UV-Vis data.

In here all the data associated with ultraviolet-visible spectroscopy are given.

**Supplementary Data Folder 2:** Cyclic voltammetry data.

In here all the data associated with cyclic voltammetry studies are given.

**Supplementary Data Folder 3:** EPR data.

In here all the data associated with the continuous wave EPR studies are given.

**Supplementary Data Folder 4:** XRD data.

In here all the cif and cif reports for the X-ray diffraction studies are given.
