# Supplementary material for: Crystalline nitrogen chain radical anions
Source: Nat Chem. 2026 Feb 10;18(4):686–94. doi: 10.1038/s41557-025-02040-2 (PMC13061614; doi:10.1038/s41557-025-02040-2)
Supplement: Supplementary file 3 — Raw data associated with [K(crypt)][3]. [file 41557_2025_2040_MOESM3_ESM.zip › Supplementary_Data_2/Folder 4 XRD/cifcheck.pdf]

checkCIF (basic structural check) running

Checking for embedded fcf data in CIF ...
Found embedded fcf data in CIF. Extracting fcf data from uploaded CIF, please wait . . .

checkCIF/PLATON (basic structural check)

Structure factors have been supplied for datablock(s) 025_r_rlr011

THIS REPORT IS FOR GUIDANCE ONLY. IF USED AS PART OF A REVIEW PROCEDURE FOR PUBLICATION, IT SHOULD NOT REPLACE THE EXPERTISE OF AN EXPERIENCED CRYSTALLOGRAPHIC REFEREE.

No syntax errors found. CIF dictionary
Please wait while processing .... Interpreting this report

Structure factor report

Datablock: 025_r_rlr011

Bond precision:	C-C = 0.0064 A		Wavelength=1.54184
Cell:	a=20.3742(3)	b=7.8682(1)	c=26.1165(4)
	alpha=90	beta=102.636(1)	gamma=90
Temperature:	100 K		

	Calculated	Reported
Volume	4085.29(10)	4085.29(10)
Space group	I 2/a	I 1 2/a 1
Hall group	-I 2ya	-I 2ya
Moiety formula	C18 H36 K N2 O6, C12 H8 F2 N4, 2(C4 H8 O)	C18 H36 K N2 O6, C12 H8 F2 N4, 2(C4 H8 O)
Sum formula	C38 H60 F2 K N6 O8	C38 H60 F2 K N6 O8
Mr	806.02	806.02
Dx,g cm-3	1.311	1.310
Z	4	4
Mu (mm-1)	1.696	1.696
F000	1724.0	1724.0
F000'	1730.93	
h,k,lmax	25,9,32	25,9,32
Nref	4258	4202
Tmin,Tmax	0.821,0.899	0.867,1.000
Tmin'	0.642	

Correction method= # Reported T Limits: Tmin=0.867 Tmax=1.000
AbsCorr = MULTI-SCAN

Data completeness= 0.987 Theta(max)= 75.775

R(reflections)= 0.0808(3616) wR2(reflections)= 0.2383(4202)

S = 1.069 Npar= 289

The following ALERTS were generated. Each ALERT has the format
test-name_ALERT_alert-type_alert-level.
Click on the hyperlinks for more details of the test.

Alert level C

CRYSC01_ALERT_1_C The word below has not been recognised as a standard identifier.
dull
PLAT241_ALERT_2_C High 'MainMol' Ueq as Compared to Neighbors of C13 Check
And 3 other PLAT241 Alerts

```
PLAT241_ALERT_2_C High   'MainMol' Ueq as Compared to Neighbors of        C17 Check
PLAT241_ALERT_2_C High   'MainMol' Ueq as Compared to Neighbors of        C23 Check
PLAT241_ALERT_2_C High   'MainMol' Ueq as Compared to Neighbors of        C27 Check
PLAT242_ALERT_2_C Low    'MainMol' Ueq as Compared to Neighbors of        K15 Check
PLAT243_ALERT_4_C High   'Solvent' Ueq as Compared to Neighbors of        O11 Check
PLAT244_ALERT_4_C Low    'Solvent' Ueq as Compared to Neighbors of         C7 Check
PLAT244_ALERT_4_C Low    'Solvent' Ueq as Compared to Neighbors of        C10 Check
PLAT260_ALERT_2_C Large Average Ueq of Residue Including      O11      0.111 Check
PLAT340_ALERT_3_C Low Bond Precision on  C-C Bonds ...............    0.00636 Ang.
PLAT360_ALERT_2_C Short  C(sp3)-C(sp3) Bond  C23      - C23_b    .       1.42 Ang.
And 2 other PLAT360 Alerts

PLAT360_ALERT_2_C Short  C(sp3)-C(sp3) Bond  C27      - C28      .       1.36 Ang.
PLAT360_ALERT_2_C Short  C(sp3)-C(sp3) Bond  C9       - C10      .       1.40 Ang.

PLAT410_ALERT_2_C Short Intra H...H Contact  H27A     ..H28B     .       1.94 Ang.
                                                      x,y,z  =      1_555 Check
PLAT410_ALERT_2_C Short Intra H...H Contact  H27B     ..H28A     .       1.94 Ang.
                                                      x,y,z  =      1_555 Check
PLAT906_ALERT_3_C Large K Value in the Analysis of Variance ......      2.845 Check
PLAT976_ALERT_2_C Check Calcd Resid. Dens.  0.84Ang From O11     .      -0.53 eA-3
```

Alert level G

```
PLAT002_ALERT_2_G Number of Distance or Angle Restraints on AtSite         14 Note
PLAT003_ALERT_2_G Number of Uiso or U(i,j)  Restrained non-H-Atoms         10 Report
PLAT072_ALERT_2_G SHELXL First  Parameter in WGHT  Unusually Large       0.14 Report
PLAT083_ALERT_2_G SHELXL Second Parameter in WGHT  Unusually Large       7.06 Why ?
PLAT176_ALERT_4_G The CIF-Embedded .res File Contains SADI Records          9 Report
PLAT178_ALERT_4_G The CIF-Embedded .res File Contains SIMU Records          1 Report
PLAT230_ALERT_2_G Hirshfeld Test Diff for    N19      --C18      .        6.0 s.u.
And 3 other PLAT230 Alerts

PLAT230_ALERT_2_G Hirshfeld Test Diff for    C13      --C29      .        9.4 s.u.
PLAT230_ALERT_2_G Hirshfeld Test Diff for    C17      --C18      .        6.3 s.u.
PLAT230_ALERT_2_G Hirshfeld Test Diff for    C17      --C26      .        9.0 s.u.

PLAT301_ALERT_3_G Main Residue  Disorder ..............(Resd    1)        30% Note
PLAT398_ALERT_2_G Deviating  C-O-C   Angle From 120 for O11      .      106.2 Degree
PLAT860_ALERT_3_G Number of Least-Squares Restraints .............         62 Note
PLAT912_ALERT_4_G Missing # of FCF Reflections Above STh/L=  0.600         54 Note
PLAT969_ALERT_5_G The 'Henn et al.' R-Factor-gap value ...........      9.860 Note
              Predicted wR2: Based on SigI**2  2.42 or SHELX Weight 22.30
PLAT978_ALERT_2_G Number C-C Bonds with Positive Residual Density.          0 Info
```

```
   0 ALERT level A = Most likely a serious problem - resolve or explain
   0 ALERT level B = A potentially serious problem, consider carefully
  18 ALERT level C = Check. Ensure it is not caused by an omission or oversight
  16 ALERT level G = General information/check it is not something unexpected

   1 ALERT type 1 CIF construction/syntax error, inconsistent or missing data
  22 ALERT type 2 Indicator that the structure model may be wrong or deficient
   4 ALERT type 3 Indicator that the structure quality may be low
   6 ALERT type 4 Improvement, methodology, query or suggestion
   1 ALERT type 5 Informative message, check
```

It is advisable to attempt to resolve as many as possible of the alerts in all categories. Often the minor alerts point to easily fixed oversights, errors and omissions in your CIF or refinement strategy, so attention to these fine details can be worthwhile. In order to resolve some of the more serious problems it may be necessary to carry out additional measurements or structure refinements. However, the purpose of your study may justify the reported deviations and the more serious of these should normally be commented upon in the discussion or experimental section of a paper or in the "special_details" fields of the CIF. checkCIF was carefully designed to identify outliers and unusual parameters, but every test has its limitations and alerts that are not important in a particular case may appear. Conversely, the absence of alerts does not guarantee there are no aspects of the results needing attention. It is up to the individual to critically assess their own results and, if necessary, seek expert advice.

Publication of your CIF in IUCr journals

A basic structural check has been run on your CIF. These basic checks will be run on all CIFs submitted for publication in IUCr journals (*Acta Crystallographica*, *Journal of Applied Crystallography*, *Journal of Synchrotron Radiation*); however, if you intend to submit to *Acta*

Crystallographica Section C or *E* or *IUCrData*, you should make sure that full publication checks are run on the final version of your CIF prior to submission.

Publication of your CIF in other journals

Please refer to the *Notes for Authors* of the relevant journal for any special instructions relating to CIF submission.

PLATON version of 02/02/2025; check.def file version of 02/02/2025

Datablock 025_r_rlr011 - ellipsoid plot

Download CIF editor (publCIF) from the IUCr
Download CIF editor (enCIFer) from the CCDC
Test a new CIF entry
