# Supplementary material for: Crystalline nitrogen chain radical anions
Source: Nat Chem. 2026 Feb 10;18(4):686–94. doi: 10.1038/s41557-025-02040-2 (PMC13061614; doi:10.1038/s41557-025-02040-2)
Supplement: Supplementary file 4 — Raw data associated with [K(crypt)][4]. [file 41557_2025_2040_MOESM4_ESM.zip › Supplementary_Data_3/Folder 4 XRD/check_cif.pdf]

checkCIF (basic structural check) running

Checking for embedded fcf data in CIF ...
Found embedded fcf data in CIF. Extracting fcf data from uploaded CIF, please wait . . . . .

checkCIF/PLATON (basic structural check)

Structure factors have been supplied for datablock(s) 025_r_rlr003

THIS REPORT IS FOR GUIDANCE ONLY. IF USED AS PART OF A REVIEW PROCEDURE FOR PUBLICATION, IT SHOULD NOT REPLACE THE EXPERTISE OF AN EXPERIENCED CRYSTALLOGRAPHIC REFEREE.

No syntax errors found. CIF dictionary
Please wait while processing .... Interpreting this report

Structure factor report

Datablock: 025_r_rlr003

Bond precision:	C-C = 0.0030 A		Wavelength=1.54184
Cell:	a=25.4601(4)	b=8.1771(1)	c=20.3593(3)
	alpha=90	beta=99.218(2)	gamma=90
Temperature:	100 K		

	Calculated	Reported
Volume	4183.86(11)	4183.86(11)
Space group	C 2/c	C 1 2/c 1
Hall group	-C 2yc	-C 2yc
Moiety formula	C18 H36 K N2 O6, C12 H8 Cl2 N4 [+ solvent]	C18 H36 K N2 O6, C12 H8 Cl2 N4
Sum formula	C30 H44 Cl2 K N6 O6 [+ solvent]	C30 H44 Cl2 K N6 O6
Mr	694.71	694.71
Dx,g cm-3	1.103	1.103
Z	4	4
Mu (mm-1)	2.626	2.626
F000	1468.0	1468.0
F000'	1476.36	
h,k,lmax	32,10,25	31,9,25
Nref	4364	4269
Tmin,Tmax	0.697,0.769	0.828,1.000
Tmin'	0.626	

Correction method= # Reported T Limits: Tmin=0.828 Tmax=1.000
AbsCorr = MULTI-SCAN

Data completeness= 0.978 Theta(max)= 75.935

R(reflections)= 0.0437(3494) wR2(reflections)= 0.1324(4269)

S = 1.048 Npar= 204

The following ALERTS were generated. Each ALERT has the format
test-name_ALERT_alert-type_alert-level.
Click on the hyperlinks for more details of the test.

Alert level C

CRYSC01_ALERT_1_C The word below has not been recognised as a standard identifier.
dull
PLAT918_ALERT_3_C Reflection(s) with I(obs) much Smaller I(calc) . 1 Check
PLAT934_ALERT_3_C Number of (Iobs-Icalc)/Sigma(W) > 10 Outliers .. 1 Check
-2 0 2,

Alert level G

```
PLAT605_ALERT_4_G Largest Solvent Accessible VOID in the Structure        251 A**3
PLAT868_ALERT_4_G ALERTS Due to the Use of _smtbx_masks Suppressed          ! Info
PLAT912_ALERT_4_G Missing # of FCF Reflections Above STh/L=  0.600         60 Note
PLAT969_ALERT_5_G The 'Henn et al.' R-Factor-gap value ...........      4.999 Note
              Predicted wR2: Based on SigI**2  2.65 or SHELX Weight 12.63
PLAT978_ALERT_2_G Number C-C Bonds with Positive Residual Density.          2 Info
```

```
  0 ALERT level A = Most likely a serious problem - resolve or explain
  0 ALERT level B = A potentially serious problem, consider carefully
  3 ALERT level C = Check. Ensure it is not caused by an omission or oversight
  5 ALERT level G = General information/check it is not something unexpected

  1 ALERT type 1 CIF construction/syntax error, inconsistent or missing data
  1 ALERT type 2 Indicator that the structure model may be wrong or deficient
  2 ALERT type 3 Indicator that the structure quality may be low
  3 ALERT type 4 Improvement, methodology, query or suggestion
  1 ALERT type 5 Informative message, check
```

It is advisable to attempt to resolve as many as possible of the alerts in all categories. Often the minor alerts point to easily fixed oversights, errors and omissions in your CIF or refinement strategy, so attention to these fine details can be worthwhile. In order to resolve some of the more serious problems it may be necessary to carry out additional measurements or structure refinements. However, the purpose of your study may justify the reported deviations and the more serious of these should normally be commented upon in the discussion or experimental section of a paper or in the "special_details" fields of the CIF. checkCIF was carefully designed to identify outliers and unusual parameters, but every test has its limitations and alerts that are not important in a particular case may appear. Conversely, the absence of alerts does not guarantee there are no aspects of the results needing attention. It is up to the individual to critically assess their own results and, if necessary, seek expert advice.

Publication of your CIF in IUCr journals

A basic structural check has been run on your CIF. These basic checks will be run on all CIFs submitted for publication in IUCr journals (*Acta Crystallographica*, *Journal of Applied Crystallography*, *Journal of Synchrotron Radiation*); however, if you intend to submit to *Acta Crystallographica Section C* or *E* or *IUCrData*, you should make sure that full publication checks are run on the final version of your CIF prior to submission.

Publication of your CIF in other journals

Please refer to the *Notes for Authors* of the relevant journal for any special instructions relating to CIF submission.

PLATON version of 02/02/2025; check.def file version of 02/02/2025

Datablock 025_r_rlr003 - ellipsoid plot

Download CIF editor (publCIF) from the IUCr
Download CIF editor (enCIFer) from the CCDC
Test a new CIF entry
