# Supplementary material for: Crystalline nitrogen chain radical anions
Source: Nat Chem. 2026 Feb 10;18(4):686–94. doi: 10.1038/s41557-025-02040-2 (PMC13061614; doi:10.1038/s41557-025-02040-2)

checkCIF/PLATON report

Structure factors have been supplied for datablock(s) 025_r_rlr017_1

THIS REPORT IS FOR GUIDANCE ONLY. IF USED AS PART OF A REVIEW PROCEDURE FOR PUBLICATION, IT SHOULD NOT REPLACE THE EXPERTISE OF AN EXPERIENCED CRYSTALLOGRAPHIC REFEREE.

No syntax errors found.   CIF dictionary   Interpreting this report

Datablock: 025_r_rlr017_1

Bond precision:	C-C = 0.0040 A		Wavelength=1.54184
Cell:	a=5.6855(2)	b=14.6607(4)	c=19.6744(5)
	alpha=90	beta=95.860(3)	gamma=90
Temperature:	100 K		

	Calculated	Reported
Volume	1631.36(8)	1631.36(8)
Space group	P 21/c	P 1 21/c 1
Hall group	-P 2ybc	-P 2ybc
Moiety formula	C14 H20 Br K N2 O2	C14 H20 Br K N2 O2
Sum formula	C14 H20 Br K N2 O2	C14 H20 Br K N2 O2
Mr	367.32	367.33
Dx,g cm-3	1.496	1.496
Z	4	4
Mu (mm-1)	5.744	5.744
F000	752.0	752.0
F000'	752.44	
h,k,lmax	7,18,24	7,18,24
Nref	3402	3337
Tmin,Tmax	0.409,0.750	0.634,1.000
Tmin'	0.276	

Correction method= # Reported T Limits: Tmin=0.634 Tmax=1.000
AbsCorr = MULTI-SCAN

Data completeness= 0.981   Theta(max)= 75.984

R(reflections)= 0.0408(2616)   wR2(reflections)= 0.1236(3337)

S = 1.064   Npar= 398

The following ALERTS were generated. Each ALERT has the format
test-name_ALERT_alert-type_alert-level.
Click on the hyperlinks for more details of the test.

Alert level C

```
PLAT088_ALERT_3_C Poor Data / Parameter Ratio ....................       8.38 Note
PLAT220_ALERT_2_C NonSolvent   Resd 1  C   Ueq(max)/Ueq(min) Range        3.2 Ratio
PLAT906_ALERT_3_C Large K Value in the Analysis of Variance ......      4.781 Check
PLAT911_ALERT_3_C Missing FCF Refl Between Thmin & STh/L=    0.600          4 Report
                6  0  0,   3  6  3,   4 10  6,   2 12 10,
```

Alert level G

```
PLAT002_ALERT_2_G Number of Distance or Angle Restraints on AtSite         37 Note
PLAT003_ALERT_2_G Number of Uiso or U(i,j)  Restrained non-H-Atoms         44 Report
PLAT004_ALERT_5_G Polymeric Structure Found with Maximum Dimension          1 Info
PLAT174_ALERT_4_G The CIF-Embedded .res File Contains FLAT Records          3 Report
PLAT176_ALERT_4_G The CIF-Embedded .res File Contains SADI Records         13 Report
PLAT178_ALERT_4_G The CIF-Embedded .res File Contains SIMU Records          2 Report
PLAT187_ALERT_4_G The CIF-Embedded .res File Contains RIGU Records          2 Report
PLAT188_ALERT_3_G A Non-default SIMU Restraint Value has been used     0.0200 Report
PLAT190_ALERT_3_G A Non-default RIGU Restraint Value for First Par     0.0020 Report
PLAT190_ALERT_3_G A Non-default RIGU Restraint Value for SecondPar     0.0020 Report
PLAT191_ALERT_3_G A Non-default SADI Restraint Value has been used     0.0400 Report
PLAT191_ALERT_3_G A Non-default SADI Restraint Value has been used     0.0400 Report
PLAT191_ALERT_3_G A Non-default SADI Restraint Value has been used     0.0400 Report
PLAT191_ALERT_3_G A Non-default SADI Restraint Value has been used     0.0400 Report
PLAT191_ALERT_3_G A Non-default SADI Restraint Value has been used     0.0400 Report
PLAT301_ALERT_3_G Main Residue  Disorder .............(Resd    1)         75% Note
PLAT720_ALERT_4_G Number of Unusual/Non-Standard Labels ..........          2 Note
              H8AA    H9AA
PLAT764_ALERT_4_G Overcomplete CIF Bond List Detected (Rep/Expd) .       1.87 Ratio
PLAT773_ALERT_2_G Check long C-C Bond in CIF: C2       --C6A             2.00 Ang.
PLAT773_ALERT_2_G Check long C-C Bond in CIF: C1       --C2A             1.83 Ang.
PLAT773_ALERT_2_G Check long C-C Bond in CIF: C5       --C6A             1.82 Ang.
PLAT773_ALERT_2_G Check long C-C Bond in CIF: C6       --C1A             1.89 Ang.
PLAT773_ALERT_2_G Check long C-C Bond in CIF: C7       --C15A            1.79 Ang.
PLAT773_ALERT_2_G Check long C-C Bond in CIF: C9       --C13A            1.72 Ang.
PLAT779_ALERT_4_G Suspect or Irrelevant (Bond) Angle(s) in CIF ...       8.00 Deg.
              C4   -BR1  -C4A     1_555   1_555   1_555 ......     #    1 Check
PLAT779_ALERT_4_G Suspect or Irrelevant (Bond) Angle(s) in CIF ...       7.90 Deg.
              K1A  -O2   -K1      1_555   1_555   1_555 ......     #   54 Check
PLAT779_ALERT_4_G Suspect or Irrelevant (Bond) Angle(s) in CIF ...       6.30 Deg.
              K1A  -O1   -K1      1_555   1_555   1_555 ......     #   61 Check
PLAT779_ALERT_4_G Suspect or Irrelevant (Bond) Angle(s) in CIF ...      15.50 Deg.
              C16A -O1   -C7      1_555   1_555   1_555 ......     #   63 Check
PLAT779_ALERT_4_G Suspect or Irrelevant (Bond) Angle(s) in CIF ...      13.00 Deg.
              C13A -O1   -C10     1_555   1_555   1_555 ......     #   67 Check
PLAT779_ALERT_4_G Suspect or Irrelevant (Bond) Angle(s) in CIF ...      22.10 Deg.
              BR1  -C4   -BR1A    1_555   1_555   1_555 ......     #   82 Check
PLAT779_ALERT_4_G Suspect or Irrelevant (Bond) Angle(s) in CIF ...      14.40 Deg.
              C5   -C4   -C5A     1_555   1_555   1_555 ......     #   89 Check
PLAT779_ALERT_4_G Suspect or Irrelevant (Bond) Angle(s) in CIF ...      15.00 Deg.
              C3A  -C4   -C3      1_555   1_555   1_555 ......     #   91 Check
PLAT779_ALERT_4_G Suspect or Irrelevant (Bond) Angle(s) in CIF ...       9.90 Deg.
```

```
              C3   -C2   -C3A     1_555   1_555   1_555 ......     #   99 Check
PLAT779_ALERT_4_G Suspect or Irrelevant (Bond) Angle(s) in CIF ...      21.70 Deg.
              C1   -C2   -C6A     1_555   1_555   1_555 ......     #  103 Check
PLAT779_ALERT_4_G Suspect or Irrelevant (Bond) Angle(s) in CIF ...      16.30 Deg.
              C1A  -C2   -C1      1_555   1_555   1_555 ......     #  108 Check
PLAT779_ALERT_4_G Suspect or Irrelevant (Bond) Angle(s) in CIF ...      37.90 Deg.
              C1A  -C2   -C6A     1_555   1_555   1_555 ......     #  110 Check
PLAT779_ALERT_4_G Suspect or Irrelevant (Bond) Angle(s) in CIF ...       8.10 Deg.
              C4   -C3   -C4A     1_555   1_555   1_555 ......     #  113 Check
PLAT779_ALERT_4_G Suspect or Irrelevant (Bond) Angle(s) in CIF ...      18.90 Deg.
              C2A  -C3   -C2      1_555   1_555   1_555 ......     #  119 Check
PLAT779_ALERT_4_G Suspect or Irrelevant (Bond) Angle(s) in CIF ...       9.30 Deg.
              C2   -C1   -C2A     1_555   1_555   1_555 ......     #  123 Check
PLAT779_ALERT_4_G Suspect or Irrelevant (Bond) Angle(s) in CIF ...      28.80 Deg.
              C1A  -C1   -C2      1_555   1_555   1_555 ......     #  136 Check
PLAT779_ALERT_4_G Suspect or Irrelevant (Bond) Angle(s) in CIF ...      33.40 Deg.
              C1A  -C1   -C2A     1_555   1_555   1_555 ......     #  139 Check
PLAT779_ALERT_4_G Suspect or Irrelevant (Bond) Angle(s) in CIF ...      11.60 Deg.
              C6A  -C1   -C6      1_555   1_555   1_555 ......     #  145 Check
PLAT779_ALERT_4_G Suspect or Irrelevant (Bond) Angle(s) in CIF ...       4.70 Deg.
              K1   -N1   -K1A     1_555   1_555   1_555 ......     #  158 Check
PLAT779_ALERT_4_G Suspect or Irrelevant (Bond) Angle(s) in CIF ...      20.60 Deg.
              C1   -N1   -C1A     1_555   1_555   1_555 ......     #  161 Check
PLAT779_ALERT_4_G Suspect or Irrelevant (Bond) Angle(s) in CIF ...      25.70 Deg.
              C1   -N1   -C6A     1_555   1_555   1_555 ......     #  162 Check
PLAT779_ALERT_4_G Suspect or Irrelevant (Bond) Angle(s) in CIF ...      32.70 Deg.
              N1A  -N1   -N2      1_555   1_555   1_555 ......     #  180 Check
PLAT779_ALERT_4_G Suspect or Irrelevant (Bond) Angle(s) in CIF ...      14.30 Deg.
              C6   -C5   -C6A     1_555   1_555   1_555 ......     #  196 Check
PLAT779_ALERT_4_G Suspect or Irrelevant (Bond) Angle(s) in CIF ...       3.60 Deg.
              C4A  -C5   -C4      1_555   1_555   1_555 ......     #  197 Check
PLAT779_ALERT_4_G Suspect or Irrelevant (Bond) Angle(s) in CIF ...       9.90 Deg.
              C1   -C6   -C1A     1_555   1_555   1_555 ......     #  211 Check
PLAT779_ALERT_4_G Suspect or Irrelevant (Bond) Angle(s) in CIF ...       8.50 Deg.
              C5A  -C6   -C5      1_555   1_555   1_555 ......     #  220 Check
PLAT779_ALERT_4_G Suspect or Irrelevant (Bond) Angle(s) in CIF ...      17.20 Deg.
              C6A  -C6   -C1      1_555   1_555   1_555 ......     #  224 Check
PLAT779_ALERT_4_G Suspect or Irrelevant (Bond) Angle(s) in CIF ...      24.10 Deg.
              C6A  -C6   -C1A     1_555   1_555   1_555 ......     #  227 Check
PLAT779_ALERT_4_G Suspect or Irrelevant (Bond) Angle(s) in CIF ...      25.70 Deg.
              N2   -N2   -N4A     3_566   1_555   1_555 ......     #  237 Check
PLAT779_ALERT_4_G Suspect or Irrelevant (Bond) Angle(s) in CIF ...      20.40 Deg.
              N2   -N2   -N3A     3_566   1_555   1_555 ......     #  238 Check
PLAT779_ALERT_4_G Suspect or Irrelevant (Bond) Angle(s) in CIF ...      17.00 Deg.
              N2A  -N2   -K1      1_555   1_555   1_555 ......     #  247 Check
PLAT779_ALERT_4_G Suspect or Irrelevant (Bond) Angle(s) in CIF ...      36.10 Deg.
              N2A  -N2   -N1      1_555   1_555   1_555 ......     #  248 Check
PLAT779_ALERT_4_G Suspect or Irrelevant (Bond) Angle(s) in CIF ...      38.70 Deg.
              N3A  -N2   -K1      1_555   1_555   1_555 ......     #  255 Check
PLAT779_ALERT_4_G Suspect or Irrelevant (Bond) Angle(s) in CIF ...      23.50 Deg.
              C8   -C7   -C15A    1_555   1_555   1_555 ......     #  264 Check
PLAT779_ALERT_4_G Suspect or Irrelevant (Bond) Angle(s) in CIF ...      14.40 Deg.
              O1A  -C7   -O1      1_555   1_555   1_555 ......     #  265 Check
PLAT779_ALERT_4_G Suspect or Irrelevant (Bond) Angle(s) in CIF ...      13.90 Deg.
              O1   -C10  -O1A     1_555   1_555   1_555 ......     #  275 Check
PLAT779_ALERT_4_G Suspect or Irrelevant (Bond) Angle(s) in CIF ...      15.30 Deg.
              C14A -C10  -C9      1_555   1_555   1_555 ......     #  285 Check
```

```
PLAT779_ALERT_4_G Suspect or Irrelevant (Bond) Angle(s) in CIF ...       7.00 Deg.
             C10  -C9   -C13A    1_555   1_555   1_555 ......     #  290 Check
PLAT779_ALERT_4_G Suspect or Irrelevant (Bond) Angle(s) in CIF ...      29.10 Deg.
             C15A -C9   -C8      1_555   1_555   1_555 ......     #  300 Check
PLAT779_ALERT_4_G Suspect or Irrelevant (Bond) Angle(s) in CIF ...      13.10 Deg.
             C9   -C8   -C14A    1_555   1_555   1_555 ......     #  308 Check
PLAT779_ALERT_4_G Suspect or Irrelevant (Bond) Angle(s) in CIF ...      14.10 Deg.
             C16A -C8   -C7      1_555   1_555   1_555 ......     #  310 Check
PLAT779_ALERT_4_G Suspect or Irrelevant (Bond) Angle(s) in CIF ...       8.80 Deg.
             C4A  -BR1A -C4      1_555   1_555   1_555 ......     #  325 Check
PLAT779_ALERT_4_G Suspect or Irrelevant (Bond) Angle(s) in CIF ...      16.60 Deg.
             C5   -C4A  -C5A     1_555   1_555   1_555 ......     #  332 Check
PLAT779_ALERT_4_G Suspect or Irrelevant (Bond) Angle(s) in CIF ...      21.30 Deg.
             BR1A -C4A  -BR1     1_555   1_555   1_555 ......     #  333 Check
PLAT779_ALERT_4_G Suspect or Irrelevant (Bond) Angle(s) in CIF ...      13.20 Deg.
             C3A  -C4A  -C3      1_555   1_555   1_555 ......     #  335 Check
PLAT779_ALERT_4_G Suspect or Irrelevant (Bond) Angle(s) in CIF ...      13.50 Deg.
             C3   -C2A  -C3A     1_555   1_555   1_555 ......     #  343 Check
PLAT779_ALERT_4_G Suspect or Irrelevant (Bond) Angle(s) in CIF ...      12.80 Deg.
             C1A  -C2A  -C1      1_555   1_555   1_555 ......     #  349 Check
PLAT779_ALERT_4_G Suspect or Irrelevant (Bond) Angle(s) in CIF ...       8.60 Deg.
             C4   -C3A  -C4A     1_555   1_555   1_555 ......     #  352 Check
PLAT779_ALERT_4_G Suspect or Irrelevant (Bond) Angle(s) in CIF ...      14.60 Deg.
             C2A  -C3A  -C2      1_555   1_555   1_555 ......     #  358 Check
PLAT779_ALERT_4_G Suspect or Irrelevant (Bond) Angle(s) in CIF ...      11.30 Deg.
             C2   -C1A  -C2A     1_555   1_555   1_555 ......     #  363 Check
PLAT779_ALERT_4_G Suspect or Irrelevant (Bond) Angle(s) in CIF ...      25.70 Deg.
             C1   -C1A  -C6      1_555   1_555   1_555 ......     #  368 Check
PLAT779_ALERT_4_G Suspect or Irrelevant (Bond) Angle(s) in CIF ...      18.60 Deg.
             C1   -C1A  -C6A     1_555   1_555   1_555 ......     #  370 Check
PLAT779_ALERT_4_G Suspect or Irrelevant (Bond) Angle(s) in CIF ...      10.00 Deg.
             C6A  -C1A  -C6      1_555   1_555   1_555 ......     #  377 Check
PLAT779_ALERT_4_G Suspect or Irrelevant (Bond) Angle(s) in CIF ...      19.80 Deg.
             C6   -C5A  -C6A     1_555   1_555   1_555 ......     #  386 Check
PLAT779_ALERT_4_G Suspect or Irrelevant (Bond) Angle(s) in CIF ...       5.60 Deg.
             C4A  -C5A  -C4      1_555   1_555   1_555 ......     #  387 Check
PLAT779_ALERT_4_G Suspect or Irrelevant (Bond) Angle(s) in CIF ...      37.40 Deg.
             C1   -C6A  -C2      1_555   1_555   1_555 ......     #  393 Check
PLAT779_ALERT_4_G Suspect or Irrelevant (Bond) Angle(s) in CIF ...      44.10 Deg.
             C1   -C6A  -N1      1_555   1_555   1_555 ......     #  394 Check
PLAT779_ALERT_4_G Suspect or Irrelevant (Bond) Angle(s) in CIF ...      12.00 Deg.
             C1   -C6A  -C1A     1_555   1_555   1_555 ......     #  396 Check
PLAT779_ALERT_4_G Suspect or Irrelevant (Bond) Angle(s) in CIF ...      35.60 Deg.
             C6   -C6A  -C5      1_555   1_555   1_555 ......     #  407 Check
PLAT779_ALERT_4_G Suspect or Irrelevant (Bond) Angle(s) in CIF ...      33.90 Deg.
             C6   -C6A  -C5A     1_555   1_555   1_555 ......     #  409 Check
PLAT779_ALERT_4_G Suspect or Irrelevant (Bond) Angle(s) in CIF ...      25.50 Deg.
             C1A  -C6A  -C2      1_555   1_555   1_555 ......     #  411 Check
PLAT779_ALERT_4_G Suspect or Irrelevant (Bond) Angle(s) in CIF ...       5.70 Deg.
             C5A  -C6A  -C5      1_555   1_555   1_555 ......     #  418 Check
PLAT779_ALERT_4_G Suspect or Irrelevant (Bond) Angle(s) in CIF ...      36.40 Deg.
             N1   -N1A  -N2A     1_555   1_555   1_555 ......     #  423 Check
PLAT779_ALERT_4_G Suspect or Irrelevant (Bond) Angle(s) in CIF ...      18.80 Deg.
             C1A  -N1A  -C1      1_555   1_555   1_555 ......     #  430 Check
PLAT779_ALERT_4_G Suspect or Irrelevant (Bond) Angle(s) in CIF ...       3.40 Deg.
             K1   -N2A  -K1A     1_555   1_555   1_555 ......     #  435 Check
PLAT779_ALERT_4_G Suspect or Irrelevant (Bond) Angle(s) in CIF ...      32.90 Deg.
```

```
             N2   -N2A  -N1A     1_555   1_555   1_555 ......     #  442 Check
PLAT779_ALERT_4_G Suspect or Irrelevant (Bond) Angle(s) in CIF ...       2.50 Deg.
             K1   -N3A  -K1A     1_555   1_555   1_555 ......     #  453 Check
PLAT779_ALERT_4_G Suspect or Irrelevant (Bond) Angle(s) in CIF ...      41.10 Deg.
             N2A  -N3A  -N2      1_555   1_555   1_555 ......     #  457 Check
PLAT779_ALERT_4_G Suspect or Irrelevant (Bond) Angle(s) in CIF ...      15.50 Deg.
             C7   -O1A  -C16A    1_555   1_555   1_555 ......     #  505 Check
PLAT779_ALERT_4_G Suspect or Irrelevant (Bond) Angle(s) in CIF ...       6.70 Deg.
             K1A  -O1A  -K1      1_555   1_555   1_555 ......     #  509 Check
PLAT779_ALERT_4_G Suspect or Irrelevant (Bond) Angle(s) in CIF ...      12.20 Deg.
             C13A -O1A  -C10     1_555   1_555   1_555 ......     #  514 Check
PLAT779_ALERT_4_G Suspect or Irrelevant (Bond) Angle(s) in CIF ...      29.40 Deg.
             C8   -C16A -C15A    1_555   1_555   1_555 ......     #  524 Check
PLAT779_ALERT_4_G Suspect or Irrelevant (Bond) Angle(s) in CIF ...      14.40 Deg.
             O1A  -C16A -O1      1_555   1_555   1_555 ......     #  525 Check
PLAT779_ALERT_4_G Suspect or Irrelevant (Bond) Angle(s) in CIF ...      14.70 Deg.
             O1   -C13A -O1A     1_555   1_555   1_555 ......     #  533 Check
PLAT779_ALERT_4_G Suspect or Irrelevant (Bond) Angle(s) in CIF ...      12.20 Deg.
             C14A -C13A -C9      1_555   1_555   1_555 ......     #  544 Check
PLAT779_ALERT_4_G Suspect or Irrelevant (Bond) Angle(s) in CIF ...      10.00 Deg.
             C10  -C14A -C13A    1_555   1_555   1_555 ......     #  548 Check
PLAT779_ALERT_4_G Suspect or Irrelevant (Bond) Angle(s) in CIF ...      25.90 Deg.
             C15A -C14A -C8      1_555   1_555   1_555 ......     #  559 Check
PLAT779_ALERT_4_G Suspect or Irrelevant (Bond) Angle(s) in CIF ...      10.50 Deg.
             C9   -C15A -C14A    1_555   1_555   1_555 ......     #  566 Check
PLAT779_ALERT_4_G Suspect or Irrelevant (Bond) Angle(s) in CIF ...       8.70 Deg.
             C16A -C15A -C7      1_555   1_555   1_555 ......     #  575 Check
PLAT789_ALERT_4_G Atoms with Negative _atom_site_disorder_group  #         40 Check
PLAT811_ALERT_5_G No ADDSYM Analysis: Too Many Excluded Atoms ....          ! Info
PLAT822_ALERT_4_G CIF-embedded .res Contains Negative PART Numbers          1 Check
PLAT860_ALERT_3_G Number of Least-Squares Restraints ............        1266 Note
PLAT883_ALERT_1_G Absent Datum for _atom_sites_solution_primary ..     Please Do !
PLAT912_ALERT_4_G Missing # of FCF Reflections Above STh/L=  0.600         60 Note
PLAT941_ALERT_3_G Average HKL Measurement Multiplicity ..........         4.4 Low
PLAT969_ALERT_5_G The 'Henn et al.' R-Factor-gap value ..........       4.066 Note
             Predicted wR2: Based on SigI**2  3.04 or SHELX Weight 11.62
PLAT978_ALERT_2_G Number C-C Bonds with Positive Residual Density.          5 Info
```

---

  0 **ALERT level A** = Most likely a serious problem - resolve or explain
  0 **ALERT level B** = A potentially serious problem, consider carefully
  4 **ALERT level C** = Check. Ensure it is not caused by an omission or oversight
112 **ALERT level G** = General information/check it is not something unexpected

  1 ALERT type 1 CIF construction/syntax error, inconsistent or missing data
 10 ALERT type 2 Indicator that the structure model may be wrong or deficient
 14 ALERT type 3 Indicator that the structure quality may be low
 88 ALERT type 4 Improvement, methodology, query or suggestion
  3 ALERT type 5 Informative message, check

It is advisable to attempt to resolve as many as possible of the alerts in all categories. Often the minor alerts point to easily fixed oversights, errors and omissions in your CIF or refinement strategy, so attention to these fine details can be worthwhile. In order to resolve some of the more serious problems it may be necessary to carry out additional measurements or structure refinements. However, the purpose of your study may justify the reported deviations and the more serious of these should normally be commented upon in the discussion or experimental section of a paper or in the "special_details" fields of the CIF. checkCIF was carefully designed to identify outliers and unusual parameters, but every test has its limitations and alerts that are not important in a particular case may appear. Conversely, the absence of alerts does not guarantee there are no aspects of the results needing attention. It is up to the individual to critically assess their own results and, if necessary, seek expert advice.

Publication of your CIF in IUCr journals

A basic structural check has been run on your CIF. These basic checks will be run on all CIFs submitted for publication in IUCr journals (*Acta Crystallographica*, *Journal of Applied Crystallography*, *Journal of Synchrotron Radiation*); however, if you intend to submit to *Acta Crystallographica Section C* or *E* or *IUCrData*, you should make sure that full publication checks are run on the final version of your CIF prior to submission.

Publication of your CIF in other journals

Please refer to the *Notes for Authors* of the relevant journal for any special instructions relating to CIF submission.

PLATON version of 04/06/2025; check.def file version of 30/05/2025

Datablock 025_r_rlr017_1 - ellipsoid plot

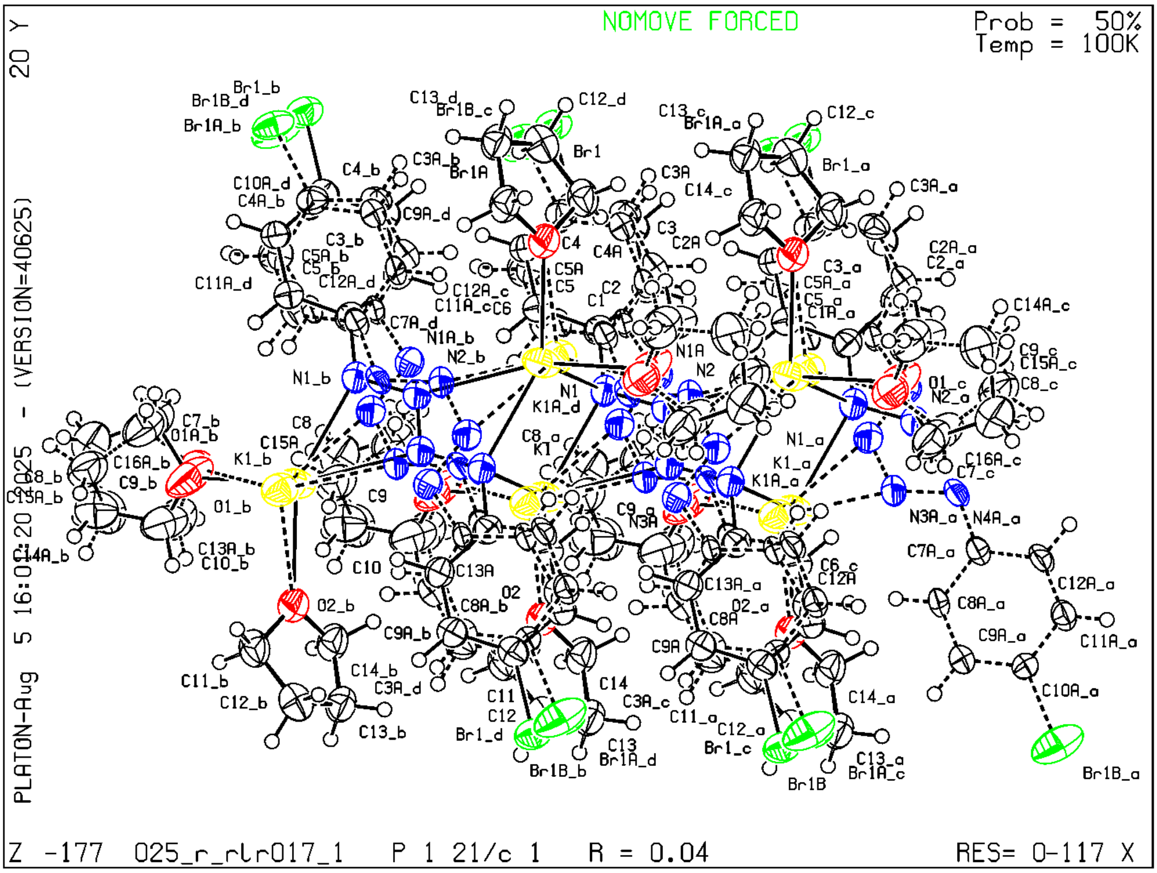

Supplement: Supplementary file 7 — Raw data associated with [K(THF)2]2[2]. [file 41557_2025_2040_MOESM7_ESM.zip › Supplementary_Data_6/Folder 1 XRD/cifreport.pdf]
