# Supplementary material for: Crystalline nitrogen chain radical anions
Source: Nat Chem. 2026 Feb 10;18(4):686–94. doi: 10.1038/s41557-025-02040-2 (PMC13061614; doi:10.1038/s41557-025-02040-2)

checkCIF/PLATON report

Structure factors have been supplied for datablock(s) 012rlr24

THIS REPORT IS FOR GUIDANCE ONLY. IF USED AS PART OF A REVIEW PROCEDURE FOR PUBLICATION, IT SHOULD NOT REPLACE THE EXPERTISE OF AN EXPERIENCED CRYSTALLOGRAPHIC REFEREE.

No syntax errors found.   CIF dictionary   Interpreting this report

Datablock: 012rlr24

```
Bond precision:   C-C = 0.0096 A           Wavelength=1.54184

Cell:              a=23.3381(2)      b=27.1976(3)      c=11.3504(1)
                   alpha=90          beta=90           gamma=90
Temperature:       150 K
```

	Calculated	Reported
Volume	7204.56(12)	7204.56(12)
Space group	P b c a	P b c a
Hall group	-P 2ac 2ab	-P 2ac 2ab
Moiety formula	C18 H36 K N2 O6, C6 H6 Br N, C7 H7 S	C6 H6 Br N, C18 H36 K N2 O6, C7 H7 S
Sum formula	C31 H49 Br K N3 O6 S	C31 H49 Br K N3 O6 S
Mr	710.79	710.82
Dx,g cm-3	1.311	1.311
Z	8	8
Mu (mm-1)	3.479	3.483
F000	2992.0	3001.2
F000’	2999.63	
h,k,lmax	27,32,13	27,32,13
Nref	6420	6420
Tmin,Tmax	0.367,0.694	0.554,1.000
Tmin’	0.277	

```
Correction method= # Reported T Limits: Tmin=0.554 Tmax=1.000
AbsCorr = MULTI-SCAN

Data completeness= 1.000            Theta(max)= 67.070

R(reflections)= 0.0893( 6269)                         wR2(reflections)=
                                                      0.2221( 6420)
S = 1.029                  Npar= 397
```

The following ALERTS were generated. Each ALERT has the format
test-name_ALERT_alert-type_alert-level.
Click on the hyperlinks for more details of the test.

Alert level B

```
PLAT411_ALERT_2_B Short Inter H...H Contact  H13      ..H35A     .        1.94 Ang.
                                      1/2-x,2-y,1/2+z  =      4_575 Check
```

Alert level C

```
PLAT042_ALERT_1_C Calc. and Reported MoietyFormula Strings  Differ     Please Check
              Calc: C18 H36 K N2 O6, C6 H6 Br N, C7 H7 S
              Rep.: C6 H6 Br N, C18 H36 K N2 O6, C7 H7 S
PLAT068_ALERT_1_C Reported F000 Differs from Calcd (or Missing)...     Please Check
PLAT241_ALERT_2_C High   'MainMol' Ueq as Compared to Neighbors of        C34 Check
PLAT341_ALERT_3_C Low Bond Precision on  C-C Bonds ...............    0.00959 Ang.
PLAT351_ALERT_3_C Long   C-H (X0.96,N1.08A)  C31      - H31A     .        1.11 Ang.
PLAT351_ALERT_3_C Long   C-H (X0.96,N1.08A)  C31      - H31B     .        1.11 Ang.
PLAT351_ALERT_3_C Long   C-H (X0.96,N1.08A)  C32      - H32A     .        1.11 Ang.
PLAT351_ALERT_3_C Long   C-H (X0.96,N1.08A)  C32      - H32B     .        1.11 Ang.
PLAT351_ALERT_3_C Long   C-H (X0.96,N1.08A)  C33      - H33A     .        1.11 Ang.
PLAT351_ALERT_3_C Long   C-H (X0.96,N1.08A)  C33      - H33B     .        1.11 Ang.
PLAT351_ALERT_3_C Long   C-H (X0.96,N1.08A)  C34      - H34A     .        1.11 Ang.
PLAT351_ALERT_3_C Long   C-H (X0.96,N1.08A)  C34      - H34B     .        1.11 Ang.
PLAT351_ALERT_3_C Long   C-H (X0.96,N1.08A)  C35      - H35A     .        1.11 Ang.
PLAT351_ALERT_3_C Long   C-H (X0.96,N1.08A)  C35      - H35B     .        1.11 Ang.
PLAT351_ALERT_3_C Long   C-H (X0.96,N1.08A)  C36      - H36A     .        1.11 Ang.
PLAT351_ALERT_3_C Long   C-H (X0.96,N1.08A)  C36      - H36B     .        1.11 Ang.
PLAT351_ALERT_3_C Long   C-H (X0.96,N1.08A)  C41      - H41A     .        1.11 Ang.
PLAT351_ALERT_3_C Long   C-H (X0.96,N1.08A)  C41      - H41B     .        1.11 Ang.
PLAT351_ALERT_3_C Long   C-H (X0.96,N1.08A)  C42      - H42A     .        1.11 Ang.
PLAT351_ALERT_3_C Long   C-H (X0.96,N1.08A)  C42      - H42B     .        1.11 Ang.
PLAT351_ALERT_3_C Long   C-H (X0.96,N1.08A)  C43      - H43A     .        1.11 Ang.
PLAT351_ALERT_3_C Long   C-H (X0.96,N1.08A)  C43      - H43B     .        1.11 Ang.
PLAT351_ALERT_3_C Long   C-H (X0.96,N1.08A)  C44      - H44A     .        1.11 Ang.
PLAT351_ALERT_3_C Long   C-H (X0.96,N1.08A)  C44      - H44B     .        1.11 Ang.
PLAT351_ALERT_3_C Long   C-H (X0.96,N1.08A)  C45      - H45A     .        1.11 Ang.
PLAT351_ALERT_3_C Long   C-H (X0.96,N1.08A)  C45      - H45B     .        1.11 Ang.
PLAT351_ALERT_3_C Long   C-H (X0.96,N1.08A)  C46      - H46A     .        1.11 Ang.
PLAT351_ALERT_3_C Long   C-H (X0.96,N1.08A)  C46      - H46B     .        1.11 Ang.
PLAT351_ALERT_3_C Long   C-H (X0.96,N1.08A)  C51      - H51A     .        1.11 Ang.
PLAT351_ALERT_3_C Long   C-H (X0.96,N1.08A)  C51      - H51B     .        1.11 Ang.
PLAT351_ALERT_3_C Long   C-H (X0.96,N1.08A)  C52      - H52A     .        1.11 Ang.
PLAT351_ALERT_3_C Long   C-H (X0.96,N1.08A)  C52      - H52B     .        1.11 Ang.
PLAT351_ALERT_3_C Long   C-H (X0.96,N1.08A)  C53      - H53A     .        1.11 Ang.
PLAT351_ALERT_3_C Long   C-H (X0.96,N1.08A)  C53      - H53B     .        1.11 Ang.
PLAT351_ALERT_3_C Long   C-H (X0.96,N1.08A)  C54      - H54A     .        1.11 Ang.
PLAT351_ALERT_3_C Long   C-H (X0.96,N1.08A)  C54      - H54B     .        1.11 Ang.
PLAT351_ALERT_3_C Long   C-H (X0.96,N1.08A)  C55      - H55A     .        1.11 Ang.
PLAT351_ALERT_3_C Long   C-H (X0.96,N1.08A)  C55      - H55B     .        1.11 Ang.
PLAT351_ALERT_3_C Long   C-H (X0.96,N1.08A)  C56      - H56A     .        1.11 Ang.
PLAT351_ALERT_3_C Long   C-H (X0.96,N1.08A)  C56      - H56B     .        1.11 Ang.
PLAT410_ALERT_2_C Short Intra H...H Contact  H35B     ..H46B     .        1.99 Ang.
                                                      x,y,z  =      1_555 Check
PLAT411_ALERT_2_C Short Inter H...H Contact  H15      ..H55B     .        2.12 Ang.
```

```
                                               -1/2+x,y,1/2-z  =      8_556 Check
PLAT906_ALERT_3_C Large K Value in the Analysis of Variance ......     10.702 Check
PLAT906_ALERT_3_C Large K Value in the Analysis of Variance ......      2.695 Check
PLAT975_ALERT_2_C Check Calcd Resid. Dens.  0.41Ang From N1      .       0.40 eA-3
PLAT977_ALERT_2_C Check Negative Difference Density on H13       .      -0.31 eA-3
```

Alert level G

```
PLAT002_ALERT_2_G Number of Distance or Angle Restraints on AtSite          3 Note
PLAT003_ALERT_2_G Number of Uiso or U(i,j)  Restrained non-H-Atoms          7 Report
PLAT083_ALERT_2_G SHELXL Second Parameter in WGHT  Unusually Large      63.34 Why ?
PLAT143_ALERT_4_G s.u. on c - Axis Small or Missing .............    0.00010 Ang.
PLAT172_ALERT_4_G The CIF-Embedded .res File Contains DFIX Records          2 Report
PLAT178_ALERT_4_G The CIF-Embedded .res File Contains SIMU Records          1 Report
PLAT187_ALERT_4_G The CIF-Embedded .res File Contains RIGU Records          1 Report
PLAT188_ALERT_3_G A Non-default SIMU Restraint Value has been used     0.0200 Report
PLAT190_ALERT_3_G A Non-default RIGU Restraint Value for First Par     0.0020 Report
PLAT190_ALERT_3_G A Non-default RIGU Restraint Value for SecondPar     0.0020 Report
PLAT769_ALERT_4_G CIF Embedded Explicitly Supplied Scattering Data          7 Note
PLAT860_ALERT_3_G Number of Least-Squares Restraints ............          89 Note
PLAT883_ALERT_1_G Absent Datum for _atom_sites_solution_primary ..     Please Do !
PLAT909_ALERT_3_G Percentage of I>2sig(I) Data at Theta(Max) Still        94% Note
PLAT910_ALERT_3_G Missing FCF Reflection(s) Below Theta(Min)[Deg]=       3.79 Note
                0  2  0,
PLAT948_ALERT_5_G Externally Supplied Scattering Factors       CIF          7 Note
PLAT969_ALERT_5_G The 'Henn et al.' R-Factor-gap value ..........      10.855 Note
              Predicted wR2: Based on SigI**2  2.05 or SHELX Weight 21.58
PLAT978_ALERT_2_G Number C-C Bonds with Positive Residual Density.          2 Info
PLAT982_ALERT_1_G The Br-f'=   -0.6696 Deviates from  IT-Value =      -0.6763 Check
PLAT983_ALERT_1_G The Br-f"=    1.2830 Deviates from  IT-Value =       1.2805 Check
PLAT983_ALERT_1_G The  K-f"=    1.0675 Deviates from  IT-Value =       1.0657 Check
PLAT983_ALERT_1_G The  S-f"=    0.5584 Deviates from  IT-Value =       0.5567 Check
```

```
   0 ALERT level A = Most likely a serious problem - resolve or explain
   1 ALERT level B = A potentially serious problem, consider carefully
  46 ALERT level C = Check. Ensure it is not caused by an omission or oversight
  22 ALERT level G = General information/check it is not something unexpected

   7 ALERT type 1 CIF construction/syntax error, inconsistent or missing data
  10 ALERT type 2 Indicator that the structure model may be wrong or deficient
  45 ALERT type 3 Indicator that the structure quality may be low
   5 ALERT type 4 Improvement, methodology, query or suggestion
   2 ALERT type 5 Informative message, check
```

It is advisable to attempt to resolve as many as possible of the alerts in all categories. Often the minor alerts point to easily fixed oversights, errors and omissions in your CIF or refinement strategy, so attention to these fine details can be worthwhile. In order to resolve some of the more serious problems it may be necessary to carry out additional measurements or structure refinements. However, the purpose of your study may justify the reported deviations and the more serious of these should normally be commented upon in the discussion or experimental section of a paper or in the "special_details" fields of the CIF. checkCIF was carefully designed to identify outliers and unusual parameters, but every test has its limitations and alerts that are not important in a particular case may appear. Conversely, the absence of alerts does not guarantee there are no aspects of the results needing attention. It is up to the individual to critically assess their own results and, if necessary, seek expert advice.

Publication of your CIF in IUCr journals

A basic structural check has been run on your CIF. These basic checks will be run on all CIFs submitted for publication in IUCr journals (*Acta Crystallographica*, *Journal of Applied Crystallography*, *Journal of Synchrotron Radiation*); however, if you intend to submit to *Acta Crystallographica Section C* or *E* or *IUCrData*, you should make sure that full publication checks are run on the final version of your CIF prior to submission.

Publication of your CIF in other journals

Please refer to the *Notes for Authors* of the relevant journal for any special instructions relating to CIF submission.

PLATON version of 04/06/2025; check.def file version of 30/05/2025

Datablock 012rlr24 - ellipsoid plot

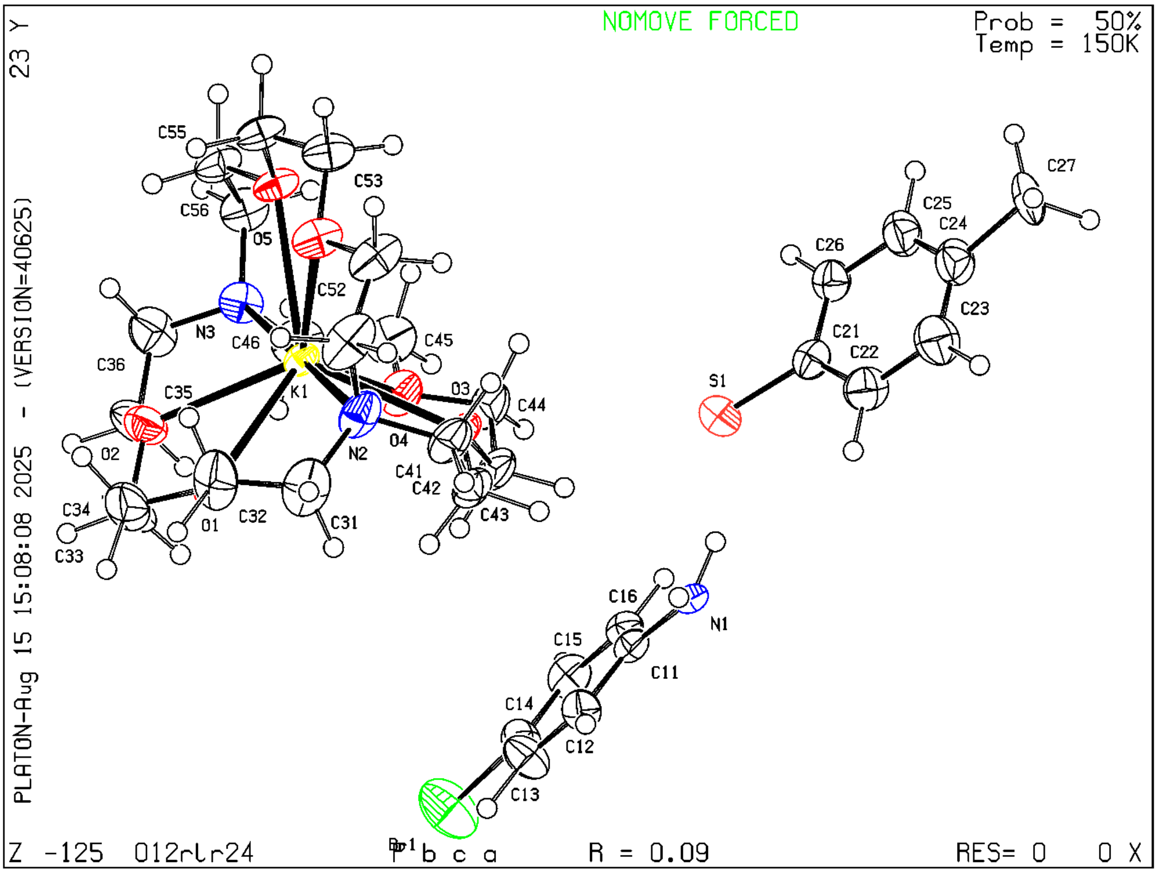

Supplement: Supplementary file 9 — Raw data associated with all other crystals. [file 41557_2025_2040_MOESM9_ESM.zip › Supplementary_Data_8/[K(crypt)][10]_[8]/NoSphereA2/012rlr24_cifreport.pdf]
