# Supplementary material for: Crystalline nitrogen chain radical anions
Source: Nat Chem. 2026 Feb 10;18(4):686–94. doi: 10.1038/s41557-025-02040-2 (PMC13061614; doi:10.1038/s41557-025-02040-2)

checkCIF/PLATON report

Structure factors have been supplied for datablock(s) 012rlr24

THIS REPORT IS FOR GUIDANCE ONLY. IF USED AS PART OF A REVIEW PROCEDURE FOR PUBLICATION, IT SHOULD NOT REPLACE THE EXPERTISE OF AN EXPERIENCED CRYSTALLOGRAPHIC REFEREE.

No syntax errors found. CIF dictionary Interpreting this report

Datablock: 012rlr24

Bond precision:	C-C = 0.0083 A		Wavelength=1.54184
Cell:	a=23.3381(2)	b=27.1976(3)	c=11.3504(1)
	alpha=90	beta=90	gamma=90
Temperature:	150 K		

	Calculated	Reported
Volume	7204.56(12)	7204.56(12)
Space group	P b c a	P b c a
Hall group	-P 2ac 2ab	-P 2ac 2ab
Moiety formula	C18 H36 K N2 O6, C6 H6 Br N, C7 H7 S	C6 H6 Br N, C18 H36 K N2 O6, C7 H7 S
Sum formula	C31 H49 Br K N3 O6 S	C31 H49 Br K N3 O6 S
Mr	710.79	710.80
Dx,g cm-3	1.311	1.311
Z	8	8
Mu (mm-1)	3.479	3.479
F000	2992.0	2992.0
F000'	2999.63	
h,k,lmax	29,34,14	29,34,14
Nref	7561	7536
Tmin,Tmax	0.367,0.694	0.552,1.000
Tmin'	0.278	

Correction method= # Reported T Limits: Tmin=0.552 Tmax=1.000
AbsCorr = MULTI-SCAN

Data completeness= 0.997 Theta(max)= 76.321

R(reflections)= 0.0871(7284) wR2(reflections)= 0.2177(7536)

S = 1.161 Npar= 389

The following ALERTS were generated. Each ALERT has the format
test-name_ALERT_alert-type_alert-level.
Click on the hyperlinks for more details of the test.

Alert level C

```
PLAT042_ALERT_1_C Calc. and Reported MoietyFormula Strings  Differ     Please Check
              Calc: C18 H36 K N2 O6, C6 H6 Br N, C7 H7 S
              Rep.: C6 H6 Br N, C18 H36 K N2 O6, C7 H7 S
PLAT214_ALERT_2_C Atom C13       (Anion/Solvent) ADP max/min Ratio        4.2 prolat
PLAT241_ALERT_2_C High   'MainMol' Ueq as Compared to Neighbors of        C23 Check
PLAT341_ALERT_3_C Low Bond Precision on  C-C Bonds ...............    0.00827 Ang.
PLAT906_ALERT_3_C Large K Value in the Analysis of Variance ......     10.343 Check
PLAT906_ALERT_3_C Large K Value in the Analysis of Variance ......      2.770 Check
PLAT972_ALERT_2_C Check Calcd Resid. Dens.  0.62Ang From Br1            -1.54 eA-3
PLAT972_ALERT_2_C Check Calcd Resid. Dens.  0.58Ang From Br1            -1.52 eA-3
PLAT975_ALERT_2_C Check Calcd Resid. Dens.  0.63Ang From N1      .       0.46 eA-3
PLAT975_ALERT_2_C Check Calcd Resid. Dens.  0.72Ang From O3      .       0.43 eA-3
PLAT976_ALERT_2_C Check Calcd Resid. Dens.  1.03Ang From N1      .      -0.45 eA-3
```

Alert level G

```
PLAT007_ALERT_5_G Number of Unrefined Donor-H Atoms ..............          2 Report
              H1A   H1B
PLAT083_ALERT_2_G SHELXL Second Parameter in WGHT  Unusually Large      40.28 Why ?
PLAT143_ALERT_4_G s.u. on c - Axis Small or Missing ..............    0.00010 Ang.
PLAT910_ALERT_3_G Missing # of FCF Reflection(s) Below Theta(Min).          1 Note
                0  2  0,
PLAT912_ALERT_4_G Missing # of FCF Reflections Above STh/L=  0.600         24 Note
PLAT969_ALERT_5_G The 'Henn et al.' R-Factor-gap value ...........      9.951 Note
              Predicted wR2: Based on SigI**2  2.19 or SHELX Weight 18.75
PLAT978_ALERT_2_G Number C-C Bonds with Positive Residual Density.          0 Info
```

```
   0 ALERT level A = Most likely a serious problem - resolve or explain
   0 ALERT level B = A potentially serious problem, consider carefully
  11 ALERT level C = Check. Ensure it is not caused by an omission or oversight
   7 ALERT level G = General information/check it is not something unexpected

   1 ALERT type 1 CIF construction/syntax error, inconsistent or missing data
   9 ALERT type 2 Indicator that the structure model may be wrong or deficient
   4 ALERT type 3 Indicator that the structure quality may be low
   2 ALERT type 4 Improvement, methodology, query or suggestion
   2 ALERT type 5 Informative message, check
```

It is advisable to attempt to resolve as many as possible of the alerts in all categories. Often the minor alerts point to easily fixed oversights, errors and omissions in your CIF or refinement strategy, so attention to these fine details can be worthwhile. In order to resolve some of the more serious problems it may be necessary to carry out additional measurements or structure refinements. However, the purpose of your study may justify the reported deviations and the more serious of these should normally be commented upon in the discussion or experimental section of a paper or in the "special_details" fields of the CIF. checkCIF was carefully designed to identify outliers and unusual parameters, but every test has its limitations and alerts that are not important in a particular case may appear. Conversely, the absence of alerts does not guarantee there are no aspects of the results needing attention. It is up to the individual to critically assess their own results and, if necessary, seek expert advice.

Publication of your CIF in IUCr journals

A basic structural check has been run on your CIF. These basic checks will be run on all CIFs submitted for publication in IUCr journals (*Acta Crystallographica*, *Journal of Applied Crystallography*, *Journal of Synchrotron Radiation*); however, if you intend to submit to *Acta Crystallographica Section C* or *E* or *IUCrData*, you should make sure that full publication checks are run on the final version of your CIF prior to submission.

Publication of your CIF in other journals

Please refer to the *Notes for Authors* of the relevant journal for any special instructions relating to CIF submission.

PLATON version of 22/08/2024; check.def file version of 21/08/2024

Datablock 012rlr24 - ellipsoid plot

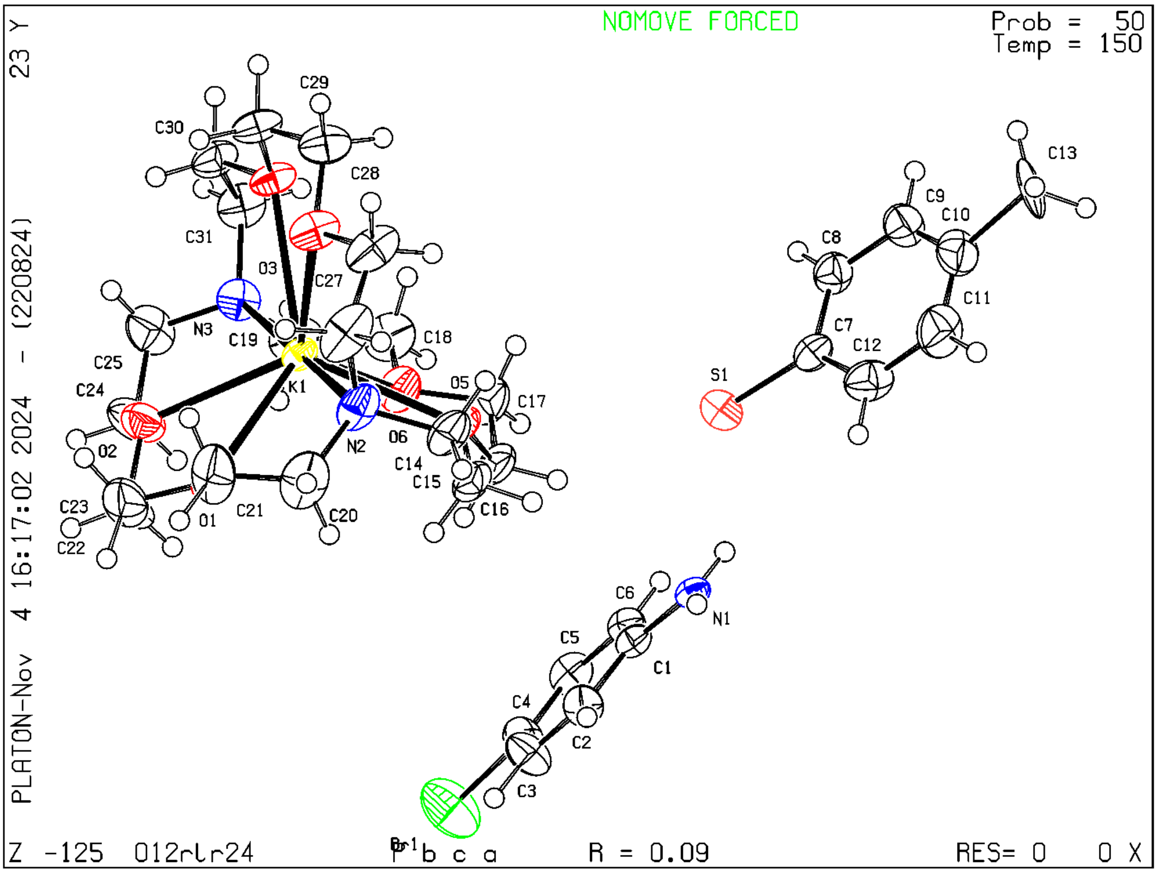

Supplement: Supplementary file 9 — Raw data associated with all other crystals. [file 41557_2025_2040_MOESM9_ESM.zip › Supplementary_Data_8/[K(crypt)][10]_[8]/[K(crypt)][4]_[6]_cifreport.pdf]
