# Supplementary material for: Crystalline nitrogen chain radical anions
Source: Nat Chem. 2026 Feb 10;18(4):686–94. doi: 10.1038/s41557-025-02040-2 (PMC13061614; doi:10.1038/s41557-025-02040-2)

checkCIF/PLATON report

Structure factors have been supplied for datablock(s) 021bvi24

THIS REPORT IS FOR GUIDANCE ONLY. IF USED AS PART OF A REVIEW PROCEDURE FOR PUBLICATION, IT SHOULD NOT REPLACE THE EXPERTISE OF AN EXPERIENCED CRYSTALLOGRAPHIC REFEREE.

No syntax errors found. CIF dictionary Interpreting this report

Datablock: 021bvi24

Bond precision:	C-C = 0.0060 A		Wavelength=1.54184
Cell:	a=17.0537(3)	b=9.2728(1)	c=20.2706(3)
	alpha=90	beta=111.898(2)	gamma=90
Temperature:	150 K		

	Calculated	Reported
Volume	2974.22(9)	2974.22(8)
Space group	P 21/n	P 1 21/n 1
Hall group	-P 2yn	-P 2yn
Moiety formula	C36 H30 Sn2	2(C18 H15 Sn)
Sum formula	C36 H30 Sn2	C36 H30 Sn2
Mr	700.02	699.98
Dx,g cm-3	1.563	1.563
Z	4	4
Mu (mm-1)	13.510	13.510
F000	1384.0	1384.0
F000’	1387.39	
h,k,lmax	21,11,25	21,11,25
Nref	6231	6166
Tmin,Tmax	0.087,0.195	0.965,0.982
Tmin’	0.011	

Correction method= # Reported T Limits: Tmin=0.965 Tmax=0.982
AbsCorr = ANALYTICAL

Data completeness= 0.990 Theta(max)= 76.151

R(reflections)= 0.0370(5819) wR2(reflections)= 0.1003(6166)

S = 1.035 Npar= 343

The following ALERTS were generated. Each ALERT has the format
test-name_ALERT_alert-type_alert-level.
Click on the hyperlinks for more details of the test.

Alert level B

```
PLAT971_ALERT_2_B Check Calcd Resid. Dens.  1.70Ang From Sn02          3.33 eA-3
```

Author Response: Residual electron density is observed around the heavy Sn atom.

Alert level C

```
PLAT042_ALERT_1_C Calc. and Reported MoietyFormula Strings  Differ     Please Check
              Calc: C36 H30 Sn2
              Rep.: 2(C18 H15 Sn)
PLAT094_ALERT_2_C Ratio of Maximum / Minimum Residual Density ....       2.58 Report
PLAT971_ALERT_2_C Check Calcd Resid. Dens.  1.39Ang From Sn01          1.67 eA-3
```

Author Response: Residual electron density is observed around the heavy Sn atom.

Alert level G

```
PLAT720_ALERT_4_G Number of Unusual/Non-Standard Labels .........         68 Note
              Sn01    Sn02    C003    C004    C005    H005    C006    C007
              C008    C009    H009    C00A    H00A    C00B    H00B    C00C
              H00C    C00D    C00E    H00E    C00F    H00F    C00G    H00G
              C00H    H00H    C00I    H00I    C00J    H00J    C00K    H00K
              C00L    H00L    C00M    H00M    C00N    H00N    C00O    H00O
              C00P    H00P    C00Q    H00Q    C00R    H00R    C00S    H00S
              C00T    H00T    C00U    H00U    C00V    H00V    C00W    H00W
              C00X    H00X    C00Y    H00Y    C00Z    H00Z    C010    H010
              C011    H011    C012    H012
PLAT790_ALERT_4_G Centre of Gravity not Within Unit Cell: Resd.  #          2 Note
              C36 H30 Sn2
PLAT883_ALERT_1_G Absent Datum for _atom_sites_solution_primary ..     Please Do !
PLAT910_ALERT_3_G Missing # of FCF Reflection(s) Below Theta(Min).          1 Note
               -1   0   1,
PLAT912_ALERT_4_G Missing # of FCF Reflections Above STh/L=  0.600         64 Note
PLAT941_ALERT_3_G Average HKL Measurement Multiplicity ..........         2.6 Low
PLAT969_ALERT_5_G The 'Henn et al.' R-Factor-gap value ..........       2.925 Note
              Predicted wR2: Based on SigI**2  3.43 or SHELX Weight  9.69
PLAT978_ALERT_2_G Number C-C Bonds with Positive Residual Density.          3 Info
```

0 **ALERT level A** = Most likely a serious problem - resolve or explain
1 **ALERT level B** = A potentially serious problem, consider carefully
3 **ALERT level C** = Check. Ensure it is not caused by an omission or oversight
8 **ALERT level G** = General information/check it is not something unexpected

2 ALERT type 1 CIF construction/syntax error, inconsistent or missing data

```
4 ALERT type 2 Indicator that the structure model may be wrong or deficient
2 ALERT type 3 Indicator that the structure quality may be low
3 ALERT type 4 Improvement, methodology, query or suggestion
1 ALERT type 5 Informative message, check
```

It is advisable to attempt to resolve as many as possible of the alerts in all categories. Often the minor alerts point to easily fixed oversights, errors and omissions in your CIF or refinement strategy, so attention to these fine details can be worthwhile. In order to resolve some of the more serious problems it may be necessary to carry out additional measurements or structure refinements. However, the purpose of your study may justify the reported deviations and the more serious of these should normally be commented upon in the discussion or experimental section of a paper or in the "special_details" fields of the CIF. checkCIF was carefully designed to identify outliers and unusual parameters, but every test has its limitations and alerts that are not important in a particular case may appear. Conversely, the absence of alerts does not guarantee there are no aspects of the results needing attention. It is up to the individual to critically assess their own results and, if necessary, seek expert advice.

Publication of your CIF in IUCr journals

A basic structural check has been run on your CIF. These basic checks will be run on all CIFs submitted for publication in IUCr journals (*Acta Crystallographica*, *Journal of Applied Crystallography*, *Journal of Synchrotron Radiation*); however, if you intend to submit to *Acta Crystallographica Section C* or *E* or *IUCrData*, you should make sure that full publication checks are run on the final version of your CIF prior to submission.

Publication of your CIF in other journals

Please refer to the *Notes for Authors* of the relevant journal for any special instructions relating to CIF submission.

PLATON version of 19/12/2024; check.def file version of 19/12/2024

Datablock 021bvi24 - ellipsoid plot

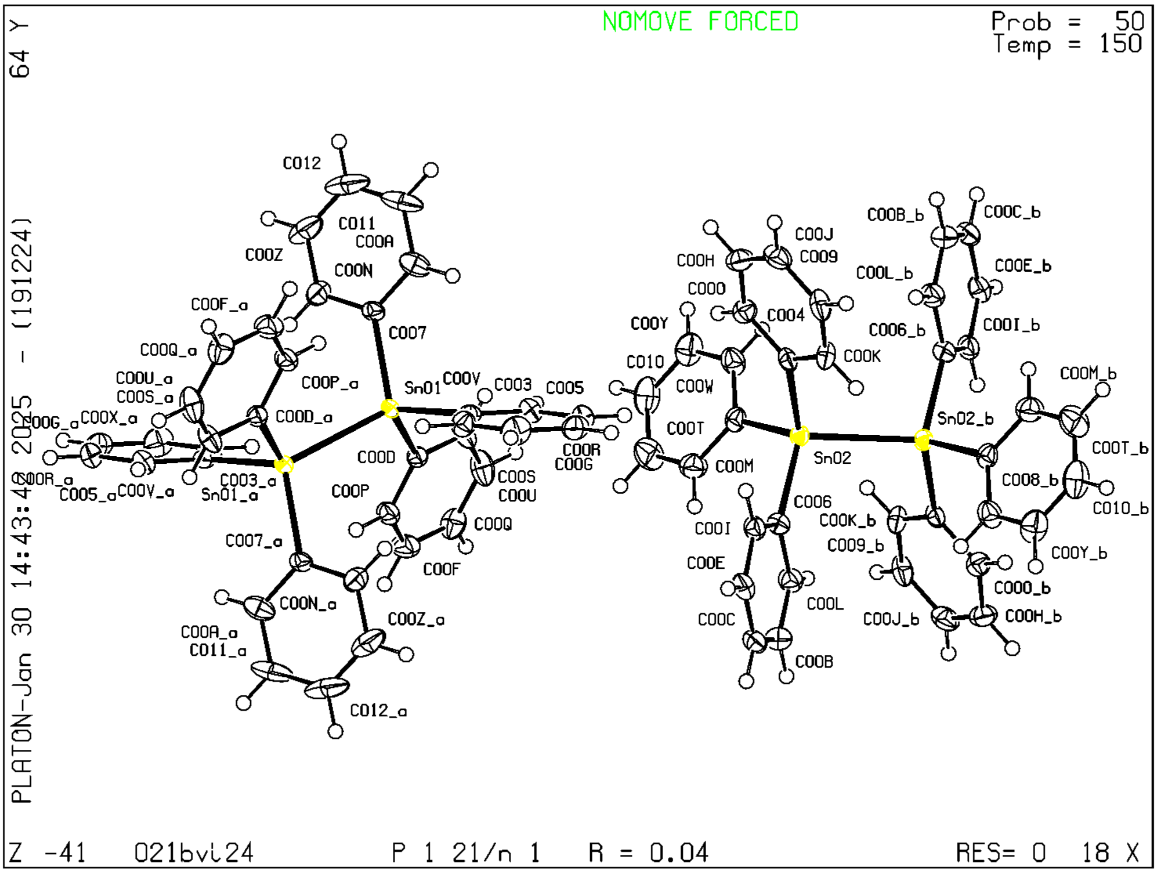

Supplement: Supplementary file 9 — Raw data associated with all other crystals. [file Sn2Ph6_cifreport.pdf › Supplementary_Data_8/Sn2Ph6/Sn2Ph6_cifreport.pdf]
